# Iminologous epoxide ring-closure†

**DOI:** 10.1039/d2sc04496j

**Published:** 2022-10-10

**Authors:** Chieh-Hung Tien, Alan J. Lough, Andrei K. Yudin

**Affiliations:** Davenport Research Laboratories, Department of Chemistry, University of Toronto Toronto ON M5S 3H6 Canada andrei.yudin@utoronto.ca; X-Ray Crystallography Laboratory, Department of Chemistry, University of Toronto Toronto ON M5S 3H6 Canada

## Abstract

The discovery of new reactions enables chemists to attain a better understanding of fundamental chemical reactivity and push the boundaries of organic synthesis. Our understanding and manipulation of high-energy states such as reactive conformations, intermediates, and transition structures contribute to this field. Herein we interrogate epoxide ring-closure by inserting the C=N functionality into a well-known precursor to nucleophilic epoxide ring-closure. The synthesis of tetrasubstituted, nitrile-tethered epoxides takes place *via* activation of iminologous diols followed by fragmentation. Mechanistic study reveals the transformation to be stereospecific, which is consistent with the concerted nature of the epoxide ring-closure.

## Abstract 2

The discovery of new reactions enables chemists to attain a better understanding of fundamental chemical reactivity and push the boundaries of organic synthesis.

## Graphical abstract

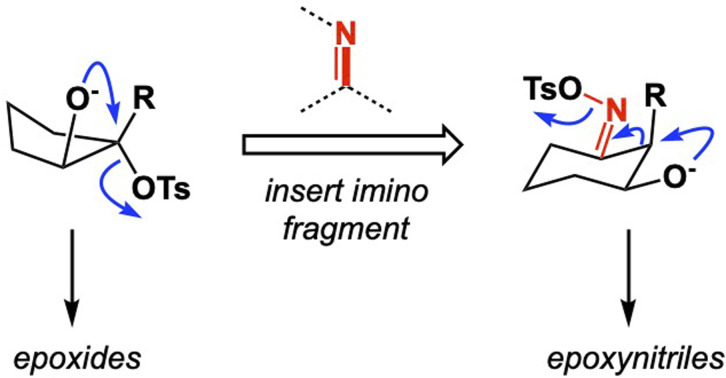

## Introduction

**Scheme 1:**
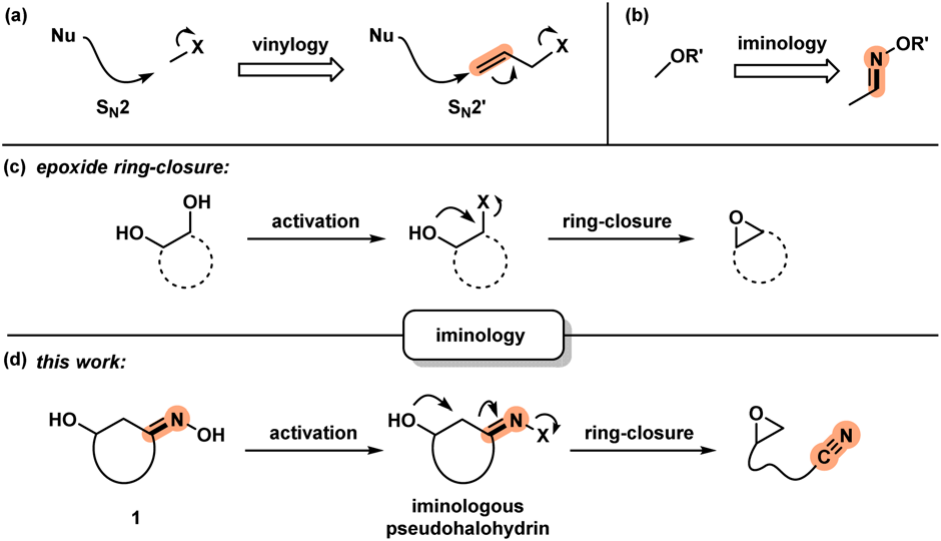
(a) S_N_2 and S_N_2′ reactions: a consideration of vinylogy; (b) application of iminology to alcohol derivatives to afford oximes; (c) ring-closure of 1,2-diols to obtain epoxides; and (d) iminologous epoxide ring-closure.

Development of new chemical transformations involves an interplay of mechanistic insights, serendipity, and unbiased screening.^1^ The *de novo* prediction of organic reactions is complicated by low-energy side processes that decrease reaction selectivity and result in low yields.^2^ Productive reaction path modification is evidenced in interrupted processes, which take place when a high-energy state such as a reactive intermediate or a strained conformation takes on an alternate path.^3^ While this appears logical, there is a lack of actionable steps that allow for the design of such transformations. Motivated by discovery of spatioenergetically-matched reaction paths, we recently initiated a program aimed at structural modification of well-established intermediates and transition state assemblies.^4^ Although the principle of vinylogy^5^ is restricted to consideration of ground states, an S_N_2′ pathway^6^ is effectively a vinylogous S_N_2 reaction (Scheme 1a). We felt that this way of looking at chemical reactivity might be applicable to other settings. Here we apply the principle of iminology^7^ to epoxide ring-closure^8^ and describe a hitherto unknown fragmentation reaction.^9,10^

Unlike the double bond in vinylogy, the utility of the imino-fragment (C=N) as a two-atom relay unit has not garnered attention in the synthetic community. Structurally speaking, oximes can be considered as the iminologues of alcohols and related ethers and esters (Scheme 1b).^11,12^ Driven by the ongoing interest in the energy-rich N–O bond of oximes,^12^ we considered the iminologue of 1,2-diols (1). Doing so appeared attractive because of the known migration, ring-closing and fragmentation transformations of 1,2-diols (Scheme 1c).^8,13^ Insertion of an imino fragment into the C–O bond of one of the hydroxy groups would afford structures such as 1, and subsequently the iminologous pseudohalohydrin upon oxime activation (Scheme 1d).^11*e*,14^

## Results and discussion

Iminologous diol 1 can be obtained from commercially available 1,3-diones by a simple 2- or 3-step sequence (see ESI†). Three initial substrates, 1a–c, were synthesized, and 1a was first reacted with 2 equivalents of KO*t*Bu and a variety of reagents to activate the N–O bond to access the desired iminologous pseudohalohydrin *in situ*. The crude mixture was then analyzed by ^1^H and ^13^C NMR spectroscopy (Table 1). The reaction of 1a with TsCl yielded a nitrile-containing species as indicated by ^13^C NMR spectroscopy, in addition to a multitude of side products, which were difficult to separate by chromatography (entry 4). Other activating agents gave rise to the iminologous pseudohalohydrin without further reaction in conjunction with traces of other species with the characteristic nitrile ^13^C signals (entries 1–3, 5). Treatment of 1a with DIPEA and TsCl, followed by KO*t*Bu provided a more manageable reaction mixture, which was subjected to column chromatography to provide ketonitrile 2a and epoxynitrile 3a in a 3 : 1 ratio with a 31% combined yield (entry 6).^15^ Iminologous diol 1b underwent a similar fragmentation process to afford a mixture of 2b and 3b in a 1 : 1 ratio with a 26% combined yield (entry 7). Ketonitriles 2a and 2b presumably arose from a semipinacol rearrangement pathway *via* a carbocation intermediate (*vide infra*). By switching to more electron-deficient 1c, the epoxynitrile 3c was synthesized as the sole product and isolated in 78% yield, suppressing the competing semipinacol rearrangement (entry 8). Increasing the equivalents of base and activating agent raised the yield to 88% (entry 9).

**Table 1:** Optimization of the reaction of 1a–c to afford ketonitriles 2a–c and/or epoxynitriles 3a–c

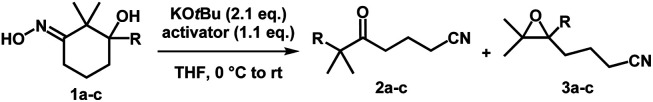				
Entry	Substrate	Activator	2 (%)	3 (%)
1^a^	1a (R = H)	Ph_2_POCl	n/a	n/a
2^a^	1a	TFAA	n/a	n/a
3^a^	1a	MsCl	n/a	n/a
4^b^	1a	TsCl	n/a	n/a
5^a^	1a	NsCl	n/a	n/a
6^c^	1a	TsCl	23	8
7	1b (R = Me)	TsCl	12	14
8	1c (R = CONB*n*_2_)	TsCl	n/a	78
9^d^	1c	TsCl	n/a	88

a The crude reaction was subjected to analysis by NMR spectroscopy, but no purification was attempted.

b Analysis by NMR spectroscopy indicated formation of nitrile-containing products, but no purification was attempted.

c DIPEA (1.1 eq.) was added to 1a, followed by TsCl (1.1 eq.). After 1 h, KO*t*Bu (2.1 eq.) was added.

d KO*t*Bu (2.4 eq.) and TsCl (1.4 eq.) were used. KO*t*Bu = potassium *tert*-butoxide, TFAA = trifluoroacetic anhydride, MsCl = methanesulfonyl chloride, TsCl = *p*-toluenesulfonyl chloride, NsCl = 2-nitrosulfonyl chloride, DIPEA = diisopropylethylamine.

**Scheme 2:**
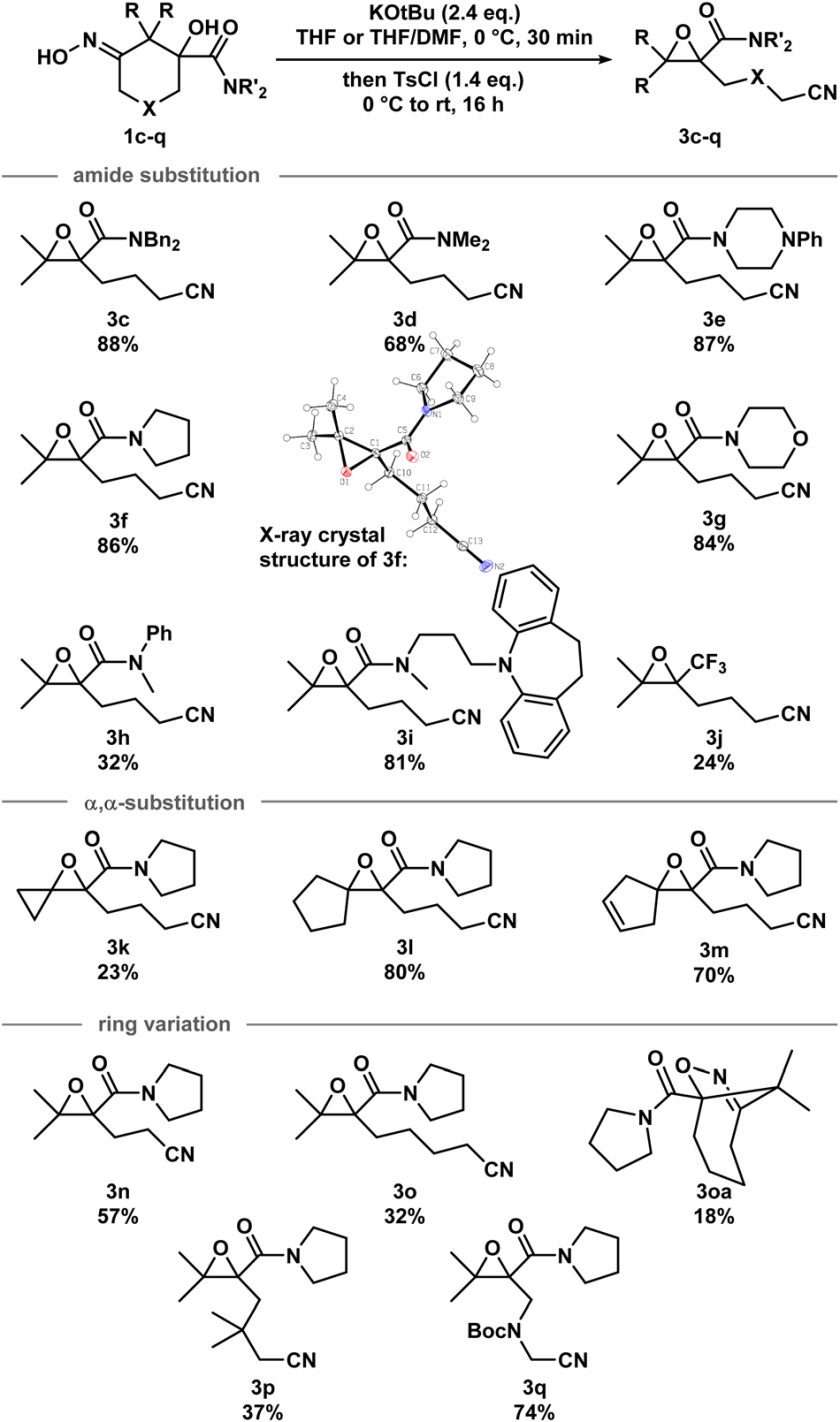
Scope of the iminologous ring-closure of iminologous diols 1c–q*via* iminologous pseudohalohydrins to afford epoxynitriles 3c–q. X-ray crystal structure of 3f (displacement ellipsoid set at 30% probability level).

With the optimized conditions in hand, a series of iminologous diols 1d–q were synthesized, and the scope of the reaction was investigated (Scheme 2). Switching the dibenzylamide moiety to dimethylamine, derived from DMF, slightly lowered the yield to 68% (3d). The presence of an aniline on the amide substituent did not hinder the reaction and 3e was obtained in high yield. Other amides derived from cyclic amines such as pyrrolidine (3f) and morpholine (3g) were also well tolerated. Single crystals of 3f were grown and analyzed by X-ray crystallography to confirm the identity of the molecule. By switching to a less electron-rich amide derived from *N*-methylaniline, the reaction became less efficient, but otherwise gave 3h in synthetically useful yields. A substrate containing the antidepressant desipramine fragment was isolated in 81% yield (3i). Changing the amide fragment to an equally electron-withdrawing CF_3_ group afforded epoxide 3j in 24% yield. Novel [2.2]- and [2.4]-spirocyclic epoxides 3k–m were synthesized in low to good yields. Notably, the olefin group in 3m was tolerated, which could be sensitive towards other oxidant-based epoxidation conditions. Reducing ring size of the precursor to five atoms gave propionitrile-tethered epoxide 3n in 57% yield. Increasing the ring size to seven atoms gave 3o in 32% yield. Interestingly, an isobaric side product (3oa) that does not contain a nitrile functional group was isolated. Based on the ^13^C NMR chemical shifts of the carbonyl species, and the coupling patterns of the methylene proton (see ESI†), the side product was proposed to be in a rigid, cyclic system. Thus, the structure of 3oa was assigned to be a bicyclo[4.2.1]-dihydroisoxazole bearing an exocyclic amide and a strained bridgehead carbonyl. This product was presumed to arise from the direct intramolecular substitution on the electrophilic nitrogen atom of the respective iminologous pseudohalohydrin by the negatively charged alkoxide. Lastly, substrates with substituents on the ring can also smoothly undergo the reaction to afford 3p and 3q in moderate to good yields.

**Scheme 3:**
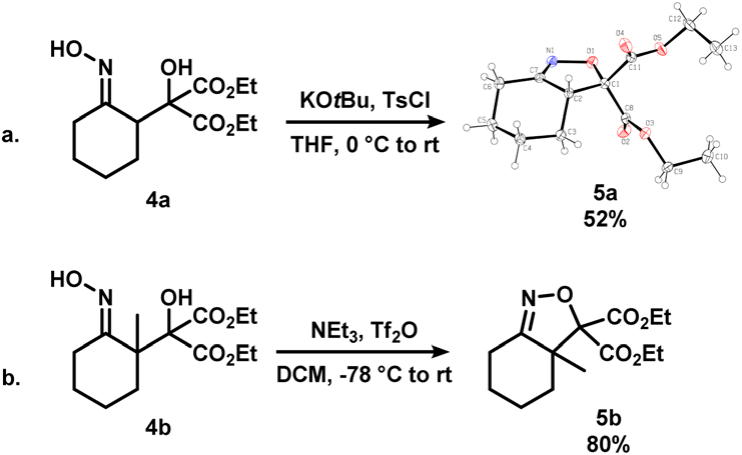
Synthesis of dihydroisoxazoles 5a (shown as X-ray crystal structure, displacement ellipsoid set at 30% probability level) and 5b*via* the activation of iminologous diols 4a and 4b.

To further expand the scope of this process, 4a and 4b, where the alcohol moiety is external to the ring system, were synthesized (Scheme 3). Treatment of 4a under standard conditions did not afford the desired epoxynitrile (Scheme 3a). To our surprise, similar to 3oa, a direct displacement of the pseudohalide by the alkoxide onto the nitrogen atom occurred to exclusively give dihydroisoxazole 5a. The identity of this species was further confirmed by single crystal X-ray crystallography. Analogous reactivity was observed when 4b was employed, and the methylated dihydrooxazines 5b was isolated in high yields (Scheme 3b). The presence of the diester substituent could lower the barrier of cyclization to favour dihydroisoxazole formation instead of the desired pathway to afford epoxides.^16^

**Scheme 4:**
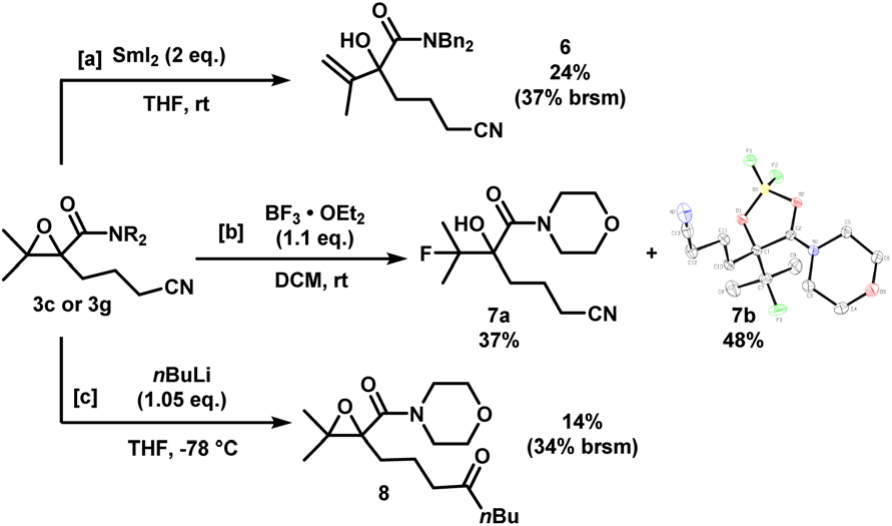
Downstream functionalization of epoxynitriles 3c and 3g. X-ray crystal structure of 7b (displacement ellipsoid set at 30% probability level).

The epoxynitriles can undergo further transformations to access other highly decorated molecules (Scheme 4). Treatment of 3c with SmI_2_ in THF effected the regioselective ring-opening of the epoxide to afford allylic alcohol 6. Lewis acid-mediated fluorination of 3g using BF_3_ gave fluorohydrin 7a in 37% yield. To our surprise, a significant amount of borylated heterocycle 7b did not undergo hydrolysis under reversed-phase chromatography conditions, and was isolated in 48% yield. The structure was identified by X-ray crystallography. Lastly, in the presence of a strong nucleophile such as *n*BuLi, the nitrile was reduced to the corresponding ketone, while maintaining the epoxide moiety, albeit in low yields.

**Scheme 5:**
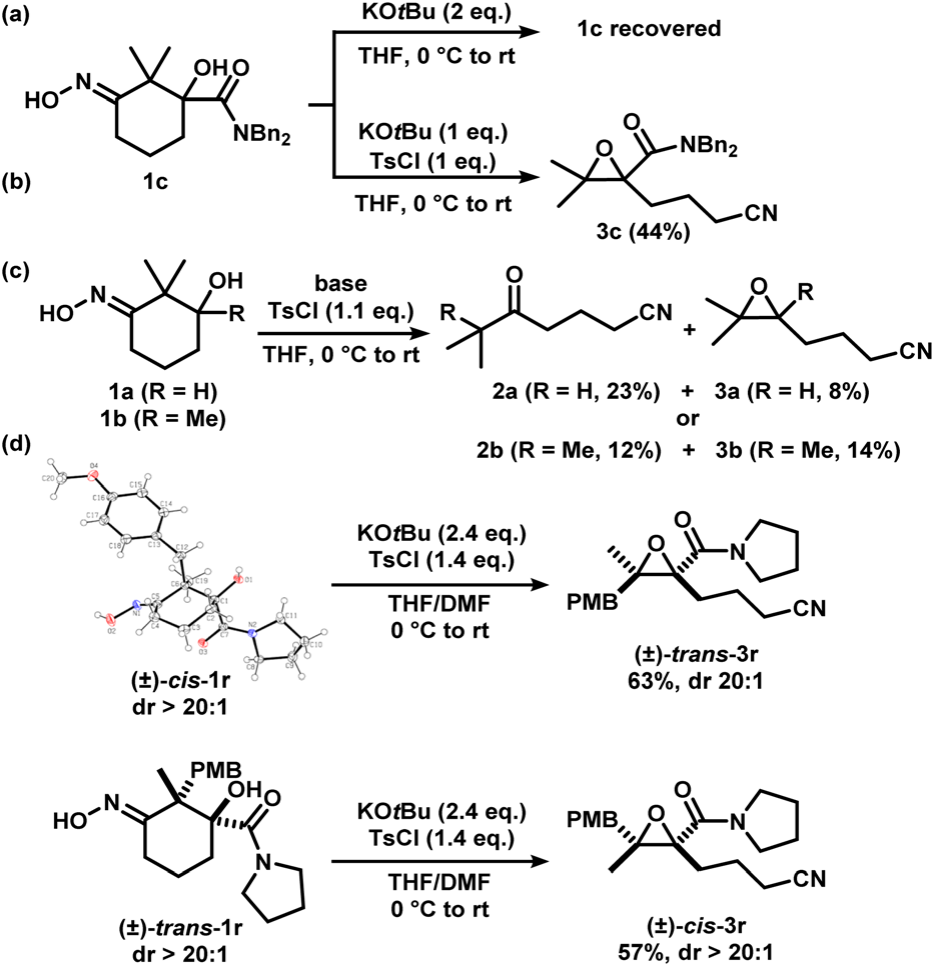
Mechanistic studies. X-ray crystal structure of (±)-*cis*-1r (displacement ellipsoid set at 30% probability level).

We considered the mechanism of this transformation (Scheme 5). Without TsCl, the reaction does not proceed, and 1c was recovered, indicating the intermediacy of the corresponding iminologous pseudohalohydrin (Scheme 5a). Using only one equivalent of KO*t*Bu and TsCl gave 3c in 44% isolated yield (Scheme 5b). It was previously established that when 1a and 1b were subjected to the reaction conditions, both the ring-closing products, 3a and 3b, and the H-migration products, 2a and 2b, were isolated (Scheme 5c). The ketones are presumed to arise from the [1,2]-H migration (semipinacol rearrangement) of the intermediates that contain adjacent carbocations. To further probe the presence of carbocations in amide-tethered substrates (since no migration was observed), oximes (±)-*cis*-1r and (±)-*trans*-1r were synthesized and isolated. The major isomer was crystallized, and a crystal structure was obtained to confirm the relative stereochemistry. Both isomers, (±)-*cis*-1r and (±)-*trans*-1r, were subsequently subjected to the standard conditions, and to our surprise, the epoxide products were afforded as diastereomerically pure species (±)-*trans*-3r and (±)-*cis*-3r, respectively (Scheme 5d). The relative configuration of the two diastereomers were assigned based on NOESY NMR assignment (see ESI†). This suggests the lack of planarization of the α-carbon atom (carbon in the α-position relative to the oxime), which is inconsistent with the formation of carbocations in this process. Therefore, C–O bond formation and C–C bond cleavage must occur in a concerted or otherwise stereospecific fashion.

**Scheme 6:**
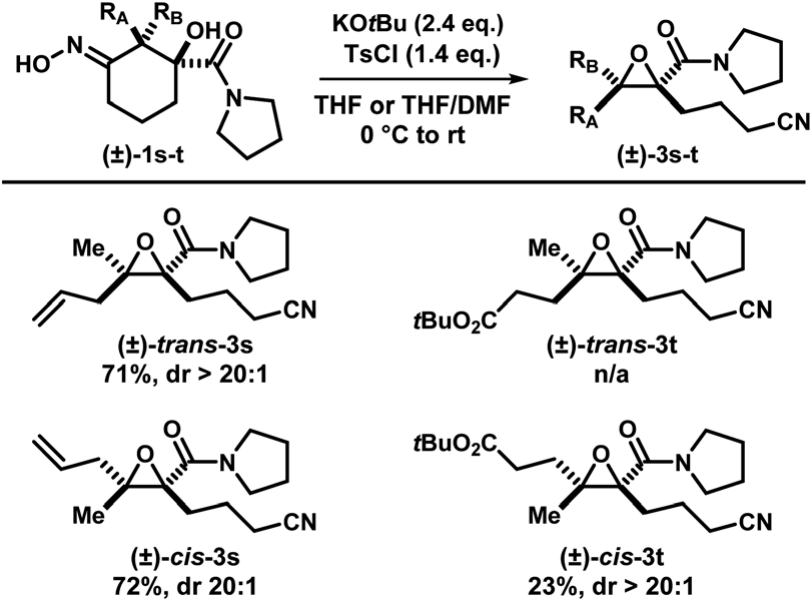
Iminologous ring-closure of diastereomerically enriched (±)-1s and (±)-1t.

Two other substrates (±)-1s and (±)-1t were synthesized and the diastereomers were separated (Scheme 6). Subjection of (±)-*trans*-1s and (±)-*cis*-1s to the optimized conditions provided epoxides (±)-*cis*-3s and (±)-*trans*-3s, respectively, with good yields in a stereospecific fashion. Ethylene ester-substituted (±)-*cis*-1t did not react under the reaction conditions, and the starting material was recovered upon hydrolysis during reversed-phase purification. Interestingly, diastereomeric (±)-*trans*-1t gave the desired epoxide (±)-*cis*-3t, albeit in low yields.

**Scheme 7:**
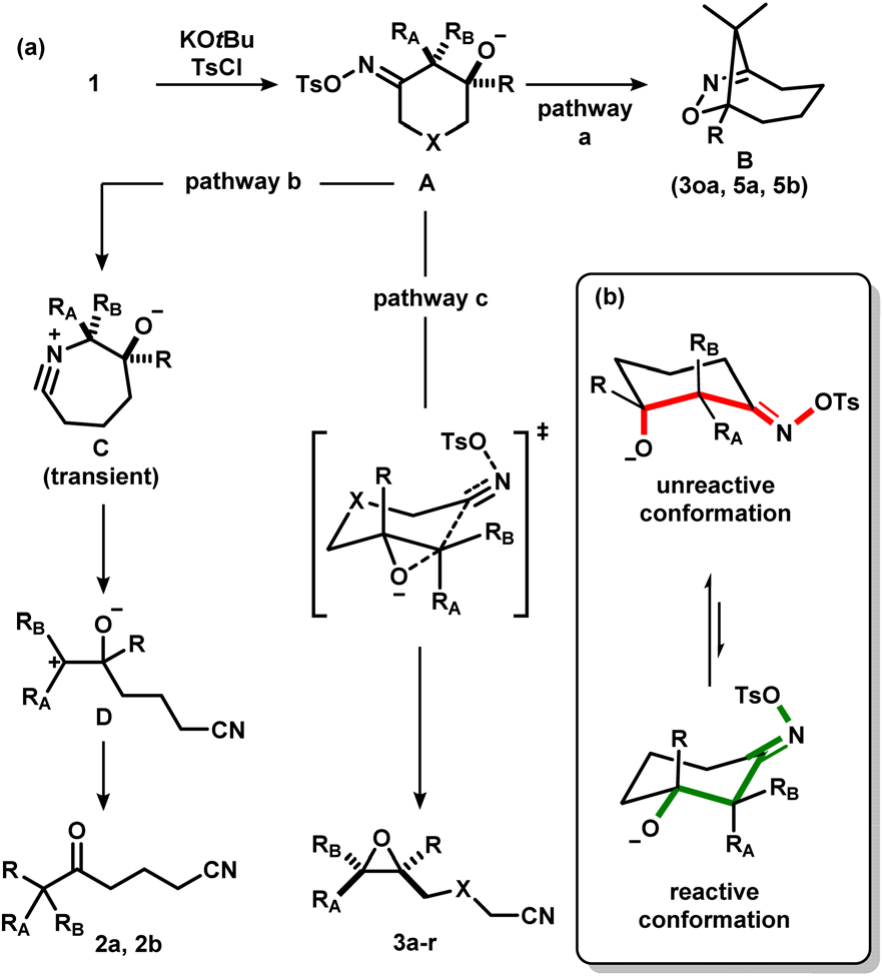
(a) Proposed mechanism for the formation of ketonitriles, epoxynitriles and dihydroisoxazoles *via* the activation of iminologous diols 1 with TsCl. (b) Analysis on the reactivity between different conformers.

Considering the aforementioned results, we put forth a potential mechanism of the epoxidation process (Scheme 7a). First, the oxime is activated to form the corresponding iminologous pseudohalohydrin A. If the ring size is sufficiently large, or if the alkoxide is spatially close to the electrophilic nitrogen, a transannular or intramolecular substitution could occur to give dihydroisoxazoles B (pathway a). Otherwise, A undergoes a stereospecific C–C fragmentation, where the group antiperiplanar to the tosylate migrates onto the nitrogen atom to generate nitrilium C as in the abnormal Beckmann rearrangement.^11*d*^ Facile fragmentation of C is expected to give the stabilized carbocation D, which could further undergo a semipinacol rearrangement to afford ketones 2 (pathway b). However, in amide-tethered substrates, the anionic oxygen atom in A likely undergoes direct intramolecular substitution to expel the oxime functional group by C–C cleavage in a concerted iminologous ring-closure to directly afford 3 (pathway c).^17^ This pathway is only possible if the oxygen atom is equatorial (Scheme 7b, green), which makes the C–O bond antiperiplanar to the breaking C–C bond as seen in the X-ray crystal structure of (±)-*cis*-1r. Since the equilibrium distribution of conformers is different between diastereomers, a change in reactivity would be expected, thus explaining the higher reactivity of (±)-*trans*-1t with respect to (±)-*cis*-1t.

## Conclusions

We have shown that insertion of the imino fragment into the key bond-breaking step of the well-established epoxide ring-closure process results in a transition state that delivers a novel iminologous fragmentation reaction to afford tetra-substituted epoxides. The mechanism has been examined, and it was proposed that the reaction is stereospecific and follows a concerted ring-closure pathway. We anticipate the application of the principle of iminology in other established processes to further demonstrate that structural modification of reactive intermediates can be used as a strategy in the development of new transformations.

## Author contributions

C.-H. T. and A. K. Y. conceived the project and wrote the manuscript. C.-H. T. designed and conducted the experiments. A. J. L. obtained and solved the crystal structures. All authors have given approval to the final version of the manuscript.

## Conflicts of interest

A. K. Y. is an associate editor of *Chemical Science*.

## Supplementary Material

SC-013-D2SC04496J-s001

SC-013-D2SC04496J-s002
