# Supplementary material for: Iminologous epoxide ring-closure
Source: Chem Sci. 2022 Oct 10;13(41):12175–9. doi: 10.1039/d2sc04496j (PMC9600474; doi:10.1039/d2sc04496j)
Supplement: SC-013-D2SC04496J-s001 [file SC-013-D2SC04496J-s001.pdf]

Electronic Supplementary Material (ESI) for Chemical Science.
This journal is © The Royal Society of Chemistry 2022

Iminologous Epoxide Ring-Closure

By

Chieh-Hung Tien,[a] Alan J. Lough,[b] Andrei K. Yudin[a,*]

[a] Davenport Research Laboratories, Department of Chemistry, University of Toronto, Toronto, ON M5S 3H6

[b] X-Ray Crystallography Laboratory, Department of Chemistry, University of Toronto, Toronto, ON M5S 3H6

Table of Contents

General considerations 3
Synthesis of precursors 4
General procedure for the synthesis of oximes: 5
Synthesis of products 15
2D NOESY NMR analysis of ***trans*-** and ***cis*-3r** 25
Figure S1. NOESY spectrum of ***trans*-3r** for diastereomer assignment. 26
Figure S2. NOESY spectrum of ***cis*-3r** for diastereomer assignment. 27
LCMS and TLC traces of ***trans*-** and ***cis*-3r** 27
Figure S3. LCMS trace of ***trans*-3r** with the MS spectrum of the product (4.03 min) shown. 27
Figure S4. LCMS trace of ***trans*-3r** with the MS spectrum of the column impurity (4.2 min) shown. 28
Figure S5. LCMS trace of ***cis*-3r** with the MS spectrum of the product (4.06 min) shown. 28
Figure S6. LCMS trace of ***cis*-3r** with the MS spectrum of the column impurity (4.2 min) shown. 28
Figure S7. LCMS trace of a blank run with the MS spectrum of the column impurity (4.2 min) shown. 29
Figure S8. LCMS trace of a mixture of ***trans*-3r** and ***cis*-3r** with the MS spectrum of the two products (4.03 min) shown. 29
Figure S9. LCMS trace of a mixture of ***trans*-3r** and ***cis*-3r** with the MS spectrum of the column impurity (4.2 min) shown. 29
Figure S10. TLC trace of ***trans*-3r** (left), ***cis*-3r** (right) and a co-spot (middle) eluted with 100% EtOAc visualized under UV light at 254 nm. 30
Figure S11. TLC trace of ***trans*-3r** (left), ***cis*-3r** (right) and a co-spot (middle) eluted with 100% EtOAc visualized with $KMnO_4$ stain. 31
NMR spectra 32
Crystallography data for **3f**, **5a**, and **7b** 94
References 127

General considerations

General: All solvents and reagents were purchased from commercial sources and used as received unless otherwise stated. Tetrahydrofuran (THF) and toluene were distilled from sodium benzophenone ketyl prior to use. Dichloromethane (DCM) was distilled from calcium hydride prior to use. Reactions were performed under inert atmosphere at room temperature (20 °C) unless otherwise stated. 2,2-Dimethyl-3-((triisopropylsilyl)oxy)cyclohex-3-en-1-one (**S1**)[1], 2,2,-disubstituted-1,3-diones[2-8] and formamides[9-10] were synthesized according to literature procedures.

Chromatography: Flash column chromatography was carried out using Silicycle 230-400 mesh silica gel eluted with hexanes/ethyl acetate. Thin-layer chromatography was performed on Merck Aluminum-backed TLC Silica gel 60 F^{254} and visualized using a UV lamp (254 nm) and $KMnO_4$ stain. Reverse-phase chromatography was carried out using iLOK SL series C18 20g 50Å on a Biotage® Isolera Prime Flash Purification System eluted with water/acetonitrile (0.1% formic acid) with the absorbance detector set to 214 nm.

Nuclear Magnetic Resonance spectroscopy: Rotamers and other conformers of several epoxynitriles were observed in ^{1}H and ^{13}C NMR spectroscopy and reported accordingly. ^{1}H, ^{13}C, and ^{19}F NMR spectra were recorded on Bruker 400 MHz and 500 MHz spectrometers at 23 °C unless otherwise stated. Spectral data is reported as follows: chemical shift, multiplicity (s = singlet, d = doublet, t = triplet, q = quartet, dd = doublet of doublets, dt = doublet of triplets, m = multiplet, br = broad), coupling constant (J) in Hertz (Hz), and integration. ^{1}H NMR chemical shifts (δ) are reported in parts per million (ppm) and referenced to residual protonated solvent peak ($CDCl_3$: δ = 7.26; DMSO-d_6: δ = 2.50). ^{13}C NMR chemical shifts (δ) are reported in ppm and referenced to the corresponding solvent peaks ($CDCl_3$: δ = 77.2; DMSO-d_6: δ = 39.5). ^{11}B NMR chemical shifts (δ) are reported in ppm and referenced to an external standard of $BF_3 \cdot OEt_2$ (δ = 0.0). ^{19}F NMR chemical shifts (δ) are reported in ppm and referenced to an external standard of $CFCl_3$ (δ = 0.0).

Mass spectroscopy: High resolution mass spectra were obtained on a VG 70-250S (double focusing) mass spectrometer at 70 eV or on an ABI/Sciex Qstar mass spectrometer with ESI or DART sources, MS/MS and accurate mass capabilities. Low-resolution mass spectra (ESI) were collected on an Agilent Technologies 1200 series HPLC paired to a 6130 Mass Spectrometer.

Infrared spectroscopy and melting point: FTIR analysis was carried out on a Bruker Alpha Platinum ATP spectrometer. Peaks are reported in wavenumbers (ν) in cm^{-1}. Melting point was

measured uncorrected utilizing a Mel-Temp capillary melting point apparatus.

RP-HPLC/MS: Low-resolution mass spectra (ESI) were collected on an Agilent Technologies 1200 series HPLC paired to a 6130 Mass Spectrometer. Compounds were resolved on Phenomenex's Kinetex 2.6u C18 50x4.6mm column at room temperature with a flow of 1 mL/min. The gradient consisted of eluents A (0.1% formic acid in Milli-Q water) and B (0.1% formic acid in HPLC-grade MeCN). Method: linear gradient starting from 5% of B to 95% over 4 min at a flow rate of 1.0 mL/min. Stays constant at 95% for 1 min and then returns to 5% over 0.5 min.

Synthesis of precursors

***tert*-Butyl 4,4-dimethyl-3,5-dioxopiperidine-1-carboxylate (S2):** *tert*-Butyl 3,5-dioxopiperidine-1-carboxylate (1.5 g, 7.0 mmol, 1 equiv.) and K_2CO_3 (2.92 g, 21.1 mmol, 3 equiv.) were added to a 100 mL round bottom flask equipped with a stir bar. MeCN (15 mL) was added, then the mixture was stirred. Iodomethane (1.3 mL, 21.1 mmol, 3 equiv.) was added, then the flask was sealed with a septum and heated in a 40 °C oil bath overnight. The mixture was cooled, then filtered over Celite, then loaded onto Celite. The volatiles were removed *in vacuo*, then the residue was subjected to normal phase column chromatography to afford the product as a colourless solid (0.197 g, 12%). ^{1}H NMR (400 MHz, $CDCl_3$) δ: 4.28 (br, 4H), 1.50 (s, 9H), 1.32 (s, 6H).

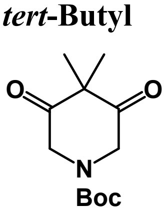

2,2-Dimethylcycloheptane-1,3-dione (S3): Tetrabutylammonium hydroxide (8.04 mL, 12.3 mmol, 1.1 equiv., 40 wt% in water) was added to a stirring solution of cycloheptane-1,3-dione (1.4 g, 11.2 mmol, 1 equiv.) dissolved in dioxane (8 mL). Iodomethane (1.58 mL, 24.6 mmol, 2.2 equiv.) was added dropwise to the stirring solution and stirred for 16 h. The mixture was washed with sat. NH_4Cl and extracted with a mixture of hexanes/ethyl acetate. The organic layers were combined and loaded onto Celite. The volatiles were removed *in vacuo*, then the residue was purified by normal phase column chromatography to afford **x** as a colourless solid (0.42 g, 2.72 mmol, 24%). ^{1}H NMR (400 MHz, $CDCl_3$) δ: 2.47-2.45 (m, 4H), 1.89-1.86 (m, 4H), 1.23 (s, 3H). $^{13}C\{^1H\}$ (101 MHz, $CDCl_3$) δ: 213.0, 41.1, 28.1, 20.8.

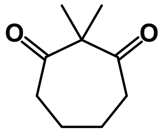

General procedure for the synthesis of iminologous diols 1:

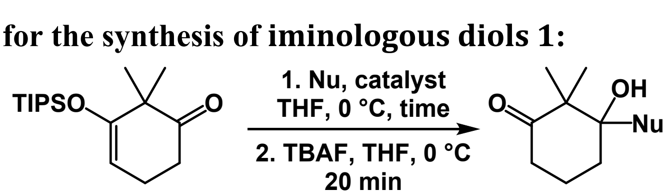

A: If the catalysts are weighable solids, then they were added to an oven-dried 50 mL round bottom flask equipped with a magnetic stir bar and purged of air with nitrogen. To an oven-dried 50 mL round bottom flask equipped with a magnetic stir bar was added a solution of **S1**

(1 equiv.) in THF (0.2 M). Then the solution was cooled with an ice water bath. The appropriate nucleophile (DIBAL-H, $TMSCF_3$, or MeMgBr) and catalyst (if liquid) were then added and stirred for the indicated time. Upon completion of reaction as indicated by TLC, the mixture was loaded onto Celite, then the volatiles were removed *in vacuo*. The residue was purified by normal phase column chromatography to afford the silyl enol ethers. These were then subjected to TBAF deprotection in THF to afford the β-hydroxy ketones, which were used without further purification.

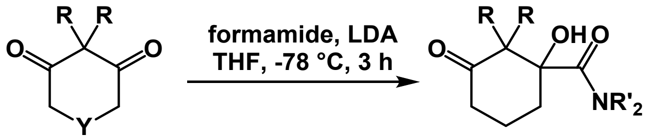

B: If the 2,2-disubstituted-1,3-diones and formamides were weighable solids, then they were added to an oven-dried 50 mL round bottom flask equipped with a magnetic stir bar and purged of air with nitrogen. If the 2,2-disubstituted-1,3-diones and formamides were liquids, then they were added to an oven-dried 50 mL round bottom flask equipped with a magnetic stir bar as solutions in THF (1 M), then the final concentration was adjusted to 0.23 M. The solution was cooled with an acetone/dry ice bath for 30 minutes. To a separate oven-dried 50 mL round bottom flask was added diisopropylamine (1 equiv. to 3.5 equiv.) and THF (0.9 M), then the mixture was cooled with an ice water bath for 10 minutes. *n*BuLi (1 equiv. or 3 equiv., 2.5 M in hexane) was added dropwise to the cooled diisopropylamine solution over 5 minutes, then the resulting mixture was stirred for an additional 10 minutes to obtain a solution of lithium diisopropylamide in THF/hexane. The freshly prepared LDA solution was added dropwise by cannula to the dione/formamide solution at -78 °C over 45 minutes. After stirring for an additional 2 h at -78 °C, formic acid (1 equiv. or 3 equiv.) dissolved in MeOH (3 mL) was added dropwise to the reaction mixture, then the mixture was loaded onto Celite. The volatiles were removed *in vacuo*, then the residue was purified by reversed-phase column chromatography to afford the β-hydroxy ketones, which were used without further characterization.

<u>General procedure for the synthesis of oximes:</u>

To the hydroxy ketone dissolved in MeOH (0.2 M) was added aniline or ammonium acetate (1 equiv.), sodium acetate (3 equiv.) and hydroxylamine hydrochloride (3 equiv.), then the mixture was stirred for 16 h at room temperature or 40 °C. The resulting mixture was diluted with MeOH and loaded onto Celite. The volatiles were removed *in vacuo*, then the residue was purified by reversed-phase column chromatography to afford the oximes.

(*E*)-3-Hydroxy-2,2-dimethylcyclohexan-1-one oxime (1a): The ketone was synthesized

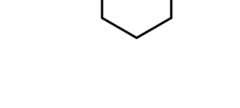

according to general procedure A from **S1** (0.537 g, 1.81 mmol, 1 equiv.) using DIBAL-H (1.45 mL, 2.17 mmol, 1.2 equiv.) as the nucleophile, and stirred for 1.5 h. The oxime was synthesized using aniline as the promoter, and obtained as a colourless solid (0.182 g, 64 % overall yield from **S1**). ^{1}H NMR (400 MHz, $CDCl_3$) δ: 9.19 (br s, 1H), 3.53-3.50 (m, 1H), 2.78-2.71 (m, 1H), 2.40-2.34 (m, 1H), 1.93-1.80 (m, 2H), 1.72-1.64 (m, 2H), 1.51-1.42 (m, 1H), 1.20 (s, 3H), 1.13 (s, 3H). ^{13}C{^{1}H} (101 MHz, $CDCl_3$) δ: 164.4, 77.3, 43.2, 29.5, 23.8, 20.9, 20.5, 20.0. Melting point: 127 – 129 °C.

(*E*)-3-Hydroxy-2,2,3-trimethylcyclohexan-1-one oxime (1b): The ketone was synthesized

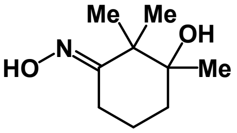

according to general procedure A from **S1** (0.400 g, 1.35 mmol, 1 equiv.) using MeMgBr (0.585 mL, 1.75 mmol, 1.3 equiv., 3M in diethyl ether) as the nucleophile and $ZnCl_2$ (0.018 g, 0.14 mmol, 10 mol %) as the catalyst, and stirred for 16 h from 0 °C to room temperature. The oxime was synthesized using aniline as the promoter and obtained as a colourless solid (0.053 g, 23 % overall yield from **S1**). ^{1}H NMR (400 MHz, $CDCl_3$) δ: 8.94 (br s, 1H), 2.75-2.68 (m, 1H), 2.50-2.43 (m, 1H), 1.80-1.71 (m, 3H), 1.63-1.55 (m, 2H), 1.43 (br s, 1H), 1.19 (s, 3H), 1.18 (s, 3H), 1.15 (s, 3H). ^{13}C{^{1}H} (101 MHz, $CDCl_3$) δ: 164.9, 75.9, 45.9, 35.7, 24.2, 22.4, 20.6, 20.4, 19.7. Melting point: 72 – 76 °C.

(*E*)-*N*,*N*-Dibenzyl-1-hydroxy-3-(hydroxyimino)-2,2-dimethylcyclohexane-1-carboxamide

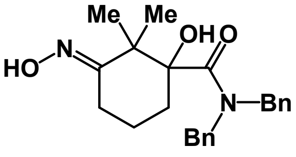

(1c): The ketone was synthesized according to general procedure B from 2,2-dimethylcyclohexane-1-one[2] (**S4**, 0.680 g, 4.8 mmol, 1 equiv.) with *N,N*-dibenzylformamide[9] (1.20 g, 5.3 mmol, 1.1 equiv.) and freshly prepared LDA solution (16.9 mmol, 3.5 equiv.), and stirred for 3 h at -78 °C. The oxime was synthesized using aniline as the promoter and heated for two nights and obtained as a colourless solid (0.437 g, 24 % overall yield from **S3**). ^{1}H NMR (400 MHz, DMSO-d_6) δ: 10.09 (s, 1H), 7.38-7.16 (m, 10H), 5.36 (s, 1H), 5.28 (d, J = 15.2 Hz, 1H), 4.80 (d, J = 15.6 Hz, 1H), 4.49 (d, J = 14.8 Hz, 1H), 4.14 (d, J = 14.8 Hz, 1H), 2.61-2.54 (m, 1H), 2.37-2.30 (m, 1H), 2.25-2.20 (m, 1H), 1.92-1.85 (m, 1H), 1.71-1.65 (m, 1H), 1.60-1.53 (m, 1H), 1.21 (s, 1H), 1.09 (s, 3H). ^{13}C{^{1}H} (101 MHz, DMSO-d_6) δ: 173.1, 162.0, 138.2, 137.9, 128.3, 127.5, 127.3, 126.9, 82.0, 50.8, 48.6, 45.6, 32.1, 23.4, 22.5, 19.1, 18.7. Melting point: 198 – 202 °C (decomp.).

(*E*)-1-Hydroxy-3-(hydroxyimino)-*N*,*N*,2,2-tetramethylcyclohexane-1-carboxamide (1d):

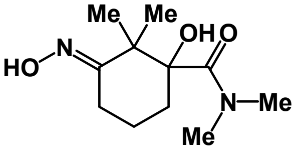

The ketone was synthesized according to general procedure B from **S4**[2] (0.210 g, 1.5 mmol, 1 equiv.) with *N-N*-dimethylformamide (0.115 mL, 1.5 mmol, 1 equiv.) and freshly prepared LDA solution (4.5 mmol, 3 equiv.), and stirred for 3 h at -78 °C. The oxime was

synthesized using aniline as the promoter and obtained as a colourless solid (0.084 g, 25 % overall yield from **S3**). ^{1}H NMR (500 MHz, DMSO-d_6) δ: 10.10 (s, 1H), 5.20 (s, 1H), 3.32 (s, 3H), 2.80 (br s, 3H), 2.61-2.56 (m, 1H), 2.25-2.16 (m, 2H), 1.64-1.60 (m, 1H), 1.56-1.52 (m, 1H), 1.47-1.42 (m, 1H), 1.07-1.06 (overlapping s, 6H). ^{13}C{^{1}H} (126 MHz, DMSO-d_6) δ: 172.7, 161.9, 81.8, 45.1, 31.7, 23.5, 22.7, 19.1, 18.8. FTIR (neat): ν = 3393, 3177, 2989, 2938, 2882, 2827, 1576, 1502, 1446, 1421, 1395, 1364, 1335, 1248, 1194, 1172, 1143, 1108, 918, 911, 802, 730, 670, 628. Melting point: 220 – 223 °C (decomp.).

(*E*)-(1-Hydroxy-3-(hydroxyimino)-2,2-dimethylcyclohexyl)(4-phenylpiperazin-1-yl)methanone (1e): The ketone was synthesized according to general procedure B from **S4**[2] (0.210 g, 1.5 mmol, 1 equiv.) with 4-phenylpiperazine-1-carbaldehyde[9] (0.285 g, 1.5 mmol, 1 equiv.) and freshly prepared LDA solution (4.5 mmol, 3 equiv.), and stirred for 3 h at -78 °C. The oxime was synthesized using aniline as the promoter and heated for two nights and obtained as a colourless solid (0.086 g, 17 % overall yield from **S3**). ^{1}H NMR (500 MHz, DMSO-d_6) δ: 10.14 (s, 1H), 7.23-7.20 (m, 2H), 6.95-6.94 (m, 2H), 6.80-6.77 (m, 1H), 5.35 (s, 3H), 4.38-3.58 (br, 4H), 3.13 (br, 4H), 2.62-2.56 (m, 1H), 2.28-2.21 (m, 1H), 1.72-1.68 (m, 1H), 1.59-1.54 (m, 1H), 1.49-1.44 (m, 1H), 1.10 (s, 6H). ^{13}C{^{1}H} (126 MHz, DMSO-d_6) δ: 171.6, 161.8, 150.9, 128.9, 119.1, 115.6, 83.0, 82.1, 52.9, 48.7, 44.9, 31.9, 23.4, 22.8, 19.0, 18.7. Melting point: 214 – 218 °C (decomp.).

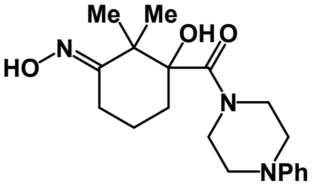

(*E*)-(1-Hydroxy-3-(hydroxyimino)-2,2-dimethylcyclohexyl)(pyrrolidin-1-yl)methanone (1f): The ketone was synthesized according to general procedure B from **S4**[2] (0.210 g, 1.5 mmol, 1 equiv.) with *N*-formylpyrrolidine[9] (0.150 g, 1.5 mmol, 1 equiv.) and freshly prepared LDA solution (4.5 mmol, 3 equiv.), and stirred for 3 h at -78 °C. The oxime was synthesized using aniline as the promoter and obtained as a colourless solid (0.116 g, 30 % overall yield from **S3**). ^{1}H NMR (500 MHz, DMSO-d_6) δ: 10.11 (s, 1H), 4.96 (s, 1H), 3.88-3.83 (m, 1H), 3.69-3.64 (m, 1H), 3.30-3.29 (m, 2H), 2.64-2.60 (m, 1H), 2.23-2.18 (m, 2H), 1.82-1.51 (m, 7H), 1.08 (s, 3H), 1.04 (s, 3H). ^{13}C{^{1}H} (126 MHz, DMSO-d_6) δ: 171.7, 161.8, 80.8, 47.9, 47.7, 44.9, 31.4, 26.9, 23.6, 22.5, 22.2, 19.0, 18.8. Melting point: 239 – 242 °C (decomp.).

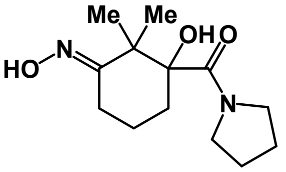

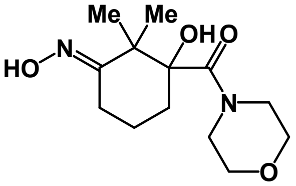

(*E*)-(1-Hydroxy-3-(hydroxyimino)-2,2-dimethylcyclohexyl)-(morpholino)methanone (1g): The ketone was synthesized according to general procedure B from **S4**[2] (0.210 g, 1.5 mmol, 1 equiv.) with *N*-formylmorpholine[9] (0.173 g, 1.5 mmol, 1 equiv.) and freshly prepared LDA solution (4.5 mmol, 3 equiv.), and stirred for 3 h at -78 °C. The oxime was synthesized using aniline as the promoter and obtained as a

colourless solid (0.204 g, 50 % overall yield from **S3**). ^{1}H NMR (500 MHz, DMSO-d_6) δ: 10.14 (s, 1H), 5.31 (s, 1H), 4.27-3.41 (overlapping br m, 8H), 2.62-2.59 (m, 1H), 2.25-2.17 (m, 2H), 1.69-1.64 (m, 1H), 1.59-1.53 (m, 1H), 1.50-1.44 (m, 1H), 1.08-1.07 (overlapping s, 6H). ^{13}C{^{1}H} (126 MHz, DMSO-d_6) δ: 171.6, 161.7, 82.2, 66.5, 44.9, 31.9, 23.5, 22.7, 19.0, 18.7. Melting point: 211 – 218 °C (decomp.).

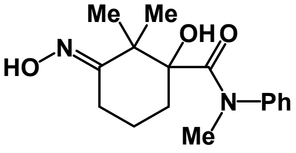

(*E*)-1-Hydroxy-3-(hydroxyimino)-*N*,2,2-trimethyl-*N*-phenylcyclohexane-1-carboxamide (1h): The ketone was synthesized according to general procedure B from **S4**[2] (0.210 g, 1.5 mmol, 1 equiv.) with *N*-methyl-*N*-phenylformamide[9] (0.203 g, 1.5 mmol, 1 equiv.) and freshly prepared LDA solution (4.5 mmol, 3 equiv.), and stirred for 3 h at -78 °C. The oxime was synthesized using aniline as the promoter and obtained as a colourless solid (0.121 g, 28 % overall yield from **S3**). ^{1}H NMR (500 MHz, DMSO-d_6) δ: 10.02 (s. 1H), 7.36-7.32 (m, 2H), 7.23-7.20 (m, 1H), 7.16-7.12 (m, 2H), 4.64 (br, 1H), 3.27 (br 3H), 2.53 (m, 1H), 2.30-2.25 (m, 1H), 2.09-2.01 (m, 1H), 1.74-1.69 (m, 1H), 1.59-1.56 (m, 2H), 1.11 (s, 3H), 1.03 (s, 3H). ^{13}C{^{1}H} (126 MHz, DMSO-d_6) δ: 173.0, 162.0, 146.9, 128.7, 126.9, 125.9, 82.9, 81.4, 45.3, 40.7, 32.2, 23.2, 19.1, 18.6. Melting point: 255 – 260 °C (decomp.).

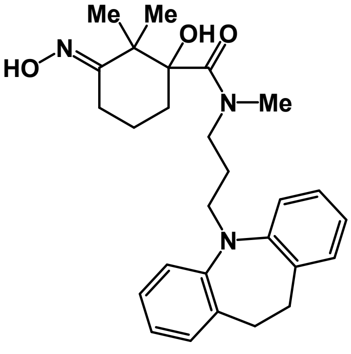

(*E*)-*N*-(3-(10,11-Dihydro-5*H*-dibenzo[*b*,*f*]azepin-5-yl)propyl)-1-hydroxy-3-(hydroxy-imino)-*N*,2,2-trimethylcyclohexane-1-carboxamide (1i): The ketone was synthesized according to general procedure B from **S4**[2] (0.143 g, 1.02 mmol, 1 equiv.) with *N*-(3-(10,11-dihydro-5*H*-dibenzo[*b*,*f*]azepin-5-yl)propyl)-*N*-methyl-formamide[10] (0.300 g, 1.02 mmol, 1 equiv.) and freshly prepared LDA solution (3.06 mmol, 3 equiv.), and stirred for 3 h at -78 °C. The oxime was synthesized using aniline as the promoter and obtained as a colourless solid (0.093 g, 20 % overall yield from **S3**). ^{1}H NMR (500 MHz, DMSO-d_6) δ: 10.07 (s, 1H), 7.12-7.09 (m, 6H), 6.92-6.89 (m, 2H), 5.14 (br, 1H), 3.68-3.65 (m, 2H), 3.17-3.09 (br, 8H), 2.56 (m, 1H), 2.19-2.07 (br, 3H), 1.67-1.39 (m, 5H), 1.04-1.02 (overlapping s, 6H). ^{13}C{^{1}H} (126 MHz, DMSO-d_6) δ: 162.0, 148.1, 133.7, 129.7, 126.3, 122.4, 119.8, 81.7, 47.7, 47.5, 45.1, 36.9, 31.6, 31.5, 25.4, 23.3, 22.8, 19.0, 18.8. Melting point: 195 – 202 °C (decomp.).

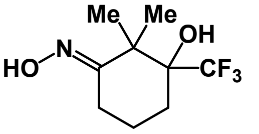

(*E*)-3-Hydroxy-2,2-dimethyl-3-(trifluoromethyl)cyclohexan-1-one oxime (1j): The ketone was synthesized according to general procedure A from **S1** (0.400 g, 1.35 mmol, 1 equiv.) using $TMSCF_3$ (0.300 mL, 2.02 mmol, 1.5 equiv.) as the nucleophile and TBAF (0.067 mL, 0.07 mmol, 5 mol %) as the catalyst, and stirred for 3 h at 0 °C. The oxime was synthesized using aniline as the promoter and

obtained as a colourless solid (0.150 g, 49 % overall yield from **S1**). ^{1}H NMR (500 MHz, DMSO-d_6) δ: 10.46 (s, 1H), 5.75 (s, 1H), 2.53-2.42 (m, 2H, overlap with solvent peak), 2.01-1.95 (m, 1H), 1.85-1.81 (m, 1H), 1.67-1.60 (m, 1H), 1.51-1.44 (m, 1H), 1.17-1.16 (overlapping s, 6H). ^{13}C{^{1}H} (126 MHz, DMSO-d_6) δ: 160.6, 127.1 (q, J = 291 Hz), 76.9 (q, J = 25 Hz), 43.7, 28.0 (d, J = 1.3 Hz), 22.2 (d, J = 2.5 Hz), 22.1 (d, J = 1.3 Hz), 18.6, 18.6. ^{19}F NMR (376 MHz, $CDCl_3$) δ: -72.98. Melting point: 175 – 181 °C.

(*E*)-(4-Hydroxy-8-(hydroxyimino)spiro[2.5]octan-4-yl)(pyrrolidin-1-yl)methanone (1k):

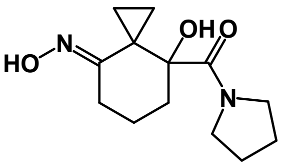

The ketone was synthesized according to general procedure B from spiro[2.5]octane-4,8-dione[3] (0.207 g, 1.5 mmol, 1 equiv.) with *N*-formylpyrrolidine[9] (0.150 g, 1.5 mmol, 1 equiv.) and freshly prepared LDA solution (4.5 mmol, 3 equiv.), and stirred for 3 h at -78 °C. The oxime was synthesized using aniline as the promoter and obtained as a colourless solid (0.134 g, 35 % overall yield from dione). ^{1}H NMR (400 MHz, DMSO-d_6) δ: 9.95 (br, 1H), 5.12 (br 1H), 3.81 (br, 1H), 3.57 (br, 1H), 3.27 (br, 2H), 2.45-2.41 (m, 1H), 2.29-2.11 (m, 2H), 1.85-1.51 (m, 7H), 0.87-0.85 (m, 1H), 0.67-0.66 (m, 2H), 0.53-0.51 (m, 1H). ^{13}C{^{1}H} (101 MHz, DMSO-d_6) δ: 171.2, 158.6, 77.2, 47.5, 47.3, 34.6, 28.7, 26.6, 22.6, 22.5, 19.2, 10.4, 7.1. Melting point: 198 – 203 °C (decomp.).

(*E*)-(6-Hydroxy-10-(hydroxyimino)spiro[4.5]decan-6-yl)(pyrrolidin-1-yl)methanone (1l):

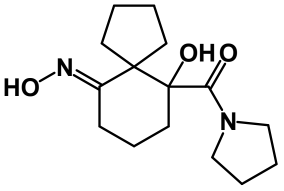

The ketone was synthesized according to general procedure B from spiro[4.5]decane-6,10-dione[4] (0.250 g, 1.5 mmol, 1 equiv.) with *N*-formylpyrrolidine[9] (0.150 g, 1.5 mmol, 1 equiv.) and freshly prepared LDA solution (4.5 mmol, 3 equiv.), and stirred for 3 h at -78 °C. The oxime was synthesized using aniline as the promoter and obtained as a colourless solid (0.081 g, 19 % overall yield from dione). ^{1}H NMR (400 MHz, DMSO-d_6) δ: 9.97 (s, 1H), 5.08 (br, 1H), 3.85-3.80 (m, 1H), 3.67-3.60 (m, 1H), 3.28-3.25 (m, 2H), 2.58-2.53 (m, 1H), 2.19-2.12 (m, 1H), 2.05-2.00 (m, 2H), 1.80-1.77 (m, 2H), 1.71-1.54 (m, 7H), 1.46-1.38 (m, 5H). ^{13}C{^{1}H} (101 MHz, DMSO-d_6) δ: 171.9, 160.8, 80.6, 57.2, 47.7, 47.5, 32.7, 32.1, 31.0, 26.8, 25.0, 25.0, 22.6, 19.4, 19.1. Melting point: 237 – 238 °C (decomp.).

(*E*)-(6-Hydroxy-10-(hydroxyimino)spiro[4.5]dec-2-en-6-yl)(pyrrolidin-1-yl)methanone (1m):

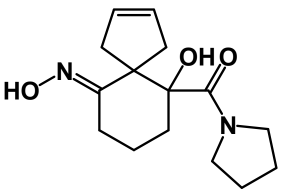

The ketone was synthesized according to general procedure B from spiro[4.5]dec-2-ene-6,10-dione[4] (0.222 g, 1.35 mmol, 1 equiv.) with *N*-formylpyrrolidine[9] (0.133 g, 1.35 mmol, 1 equiv.) and freshly prepared LDA solution (4.05 mmol, 3 equiv.), and stirred for 3 h at -78 °C. The oxime was synthesized using aniline as the promoter and obtained as a colourless solid (0.124 g, 33 % overall yield from dione). ^{1}H NMR (400 MHz,

DMSO-d_6) δ: 9.95 (s, 1H), 5.51 (d, J = 5.6 Hz, 1H), 5.39 (d, J = 5.6 Hz, 1H), 5.1 (s, 1H), 3.87-3.81 (m, 1H), 3.66-3.61 (m, 1H), 3.27-3.24 (m, 2H), 2.90-2.86 (m, 1H), 2.74-2.70 (m, 1H), 2.63-2.59 (m, 1H), 2.42-2.37 (m, 1H), 2.24-2.20 (m, 1H), 2.09-1.93 (m, 2H), 1.79-1.56 (m, 6H), 1.37-1.29 (m, 1H). $^{13}C\{^1H\}$ (101 MHz, DMSO-d_6) δ: 171.6, 160.1, 129.8, 126.8, 80.1, 56.1, 47.4, 39.7, 38.3, 31.7, 26.7, 22.6, 19.2, 18.9. Melting point: 247 – 250 °C (decomp.).

(*E*)-(1-Hydroxy-3-(hydroxyimino)-2,2-dimethylcyclopentyl)(pyrrolidin-1-yl)methanone (1n): The ketone was synthesized according to general procedure B from 2,2-dimethylcyclopentane-1,3-dione[5] (0.189 g, 1.5 mmol, 1 equiv.) with *N*-formylpyrrolidine[9] (0.150 g, 1.5 mmol, 1 equiv.) and freshly prepared LDA solution (4.5 mmol, 3 equiv.), and stirred for 3 h at -78 °C. The oxime was synthesized using aniline as the promoter and obtained as a colourless solid (0.099 g, 27 % overall yield from dione). ^{1}H NMR (500 MHz, DMSO-d_6) δ: 10.12 (s, 1H), 5.30 (br, 1H), 3.86-3.82 (m, 1H), 3.69-3.64 (m, 1H), 3.37-3.27 (m, 2H), 2.48-2.40 (m, 2H), 2.35-2.30 (m, 1H), 1.85-1.83 (m, 1H), 1.76-1.60 (m, 4H), 1.09 (s, 3H), 0.91 (s, 3H). $^{13}C\{^1H\}$ (126 MHz, DMSO-d_6) δ: 170.9, 167.6, 85.8, 49.0, 47.9, 47.4, 33.2, 26.8, 24.0, 23.4, 22.5, 21.5. Melting point: 207 – 211 °C (decomp.).

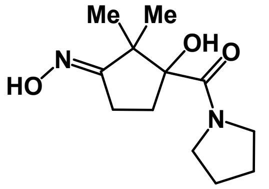

(*E*)-(1-Hydroxy-3-(hydroxyimino)-2,2-dimethylcycloheptyl)(pyrrolidin-1-yl)methanone (1o): The ketone was synthesized according to general procedure B from **S2** (0.417 g, 2.7 mmol, 1 equiv.) with *N*-formylpyrrolidine[9] (0.268 g, 2.7 mmol, equiv.) and freshly prepared LDA solution (0.3 mmol, 1.1 equiv.), and stirred for 3 h at -78 °C. The oxime was synthesized using ammonium acetate as the promoter with 4 equivalents of NH_2OH • HCl and sodium acetate at 60 °C for 5 days and obtained as a colourless solid (0.097 g, 13 % overall yield from dione). ^{1}H NMR (500 MHz, DMSO-d_6) δ: 10.27 (s, 1H), 4.79 (s, 1H), 3.86-3.82 (m, 1H), 3.71-3.66 (m, 1H), 3.31-3.29 (m, 2H, overlap with water), 2.55-2.2.44 (m, 2H, overlap with solvent peak), 2.13-2.08 (m, 1H), 1.87-1.45 (m, 9H), 1.18 (s, 3H), 1.10 (s, 3H). $^{13}C\{^1H\}$ (126 MHz, DMSO-d_6) δ: 173.4, 163.1, 80.8, 48.2, 47.9, 47.9, 36.2, 26.9, 25.1, 24.1, 23.7, 23.6, 22.5, 22.3. Melting point: 187 – 193 °C.

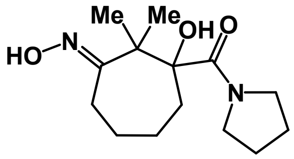

(*E*)-(1-Hydroxy-3-(hydroxyimino)-2,2,5,5-tetramethylcyclohexyl)(pyrrolidin-1-yl)methanone (1p): The ketone was synthesized according to general procedure B from 2,2,5,5-tetramethylcyclohexane-1,3-dione[6] (0.147 g, 0.87 mmol, 1 equiv.) with *N*-formylpyrrolidine[9] (0.087 g, 0.87 mmol, 1 equiv.) and freshly prepared LDA solution (2.62 mmol, 3 equiv.), and stirred for 3 h at -78 °C. The oxime was synthesized using aniline

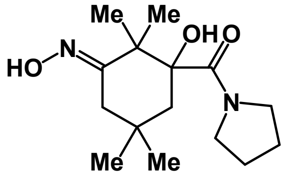

as the promoter and stirred for 2 nights and obtained as a colourless solid (0.081 g, 33 % overall yield from dione). ^{1}H NMR (400 MHz, DMSO-d_6) δ: 10.23 (s, 1H), 4.78 (s, 1H), 3.91-3.88 (m, 1H), 3.66-3.62 (m, 1H), 3.34-3.31 (overlap with water, 2H), 2.90 (d, J = 14 Hz, 1H), 2.44 (d, J = 14 Hz, 1H), 1.87-1.83 (m, 1H), 1.77-1.69 (m, 3H), 1.61-1.59 (m, 1H), 1.40 (d, J = 15.2 Hz, 1H), 1.07 (s, 3H), 1.03 (s, 3H), 0.96 (s, 6H). ^{13}C{^{1}H} (101 MHz, DMSO-d_6) δ:171.6, 160.4, 82.5, 48.2, 48.1, 44.1, 44.1, 33.3, 32.4, 31.9, 28.9, 27.0, 25.6, 22.4, 21.2. Melting point: 254 – 256 °C (decomp.).

***tert*-Butyl (*Z*)-3-hydroxy-5-(hydroxyimino)-4,4-dimethyl-3-(pyrrolidine-1-carbonyl)-piperidine-1-carboxylate (1q):**

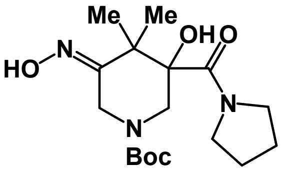

The ketone was synthesized according to general procedure B from **S2** (0.185 g, 0.77 mmol, 1 equiv.) with *N*-formylpyrrolidine[9] (0.076 g, 0.77 mmol, equiv.) and freshly prepared LDA solution (2.3 mmol, 3 equiv.), and stirred for 3 h at -78 °C. The oxime was synthesized using aniline as the promoter and obtained as a colourless solid (0.027 g, 10 % overall yield from dione). ^{1}H NMR (500 MHz, DMSO-d_6) δ: 10.48 (br, 1H), 5.58 (br, 1H), 4.51-4.35 (m, 1H), 4.09-3.81 (m, 3H), 3.72-3.65 (m, 1H), 3.35-3.29 (m, 3H), 1.83-1.65 (m, 4H), 1.39-1.38 (m, 9H), 1.12-1.10 (overlapping s, 6H). ^{13}C{^{1}H} (126 MHz, DMSO-d_6, mixture of rotamers) δ: 169.9, 169.9, 158.2, 154.2, 154.0, 78.9, 78.8, 78.0, 78.0, 47.9, 47.7, 47.2, 43.6, 38.8, 38.0, 28.1, 26.8, 26.7, 22.5, 22.5, 22.4, 22.0. Melting point: 212 – 219 °C (decomp.).

Diethyl (*E*)-2-hydroxy-2-(2-(hydroxyimino)cyclohexyl)malonate (4a):

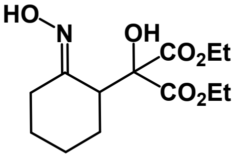

Cyclohexanone (0.104 mL, 1 mmol, equiv.) was added to an oven-dried 50 mL round bottom flask, equipped with a magnetic stir bar dissolved in THF (5 mL) and stirred in an acetone/dry ice bath for 30 minutes. A freshly prepared solution of LDA (1.05 mmol, 1.05 equiv.) was added dropwise to the solution over 5 minutes at -78 °C. Diethyl mesoxalate (0.153 mL, 1 mmol, 1 equiv.) was added dropwise at -78 °C and the mixture was stirred for 16 h while warming up to room temperature. The reaction was quenched with saturated NH_4Cl and extracted with a mixture of hexanes/ethyl acetate. The organic layers were combined and loaded onto Celite. The volatiles were removed *in vacuo* then the residue was subjected to normal phase column chromatography to afford the hydroxy ketone which was used without further characterization. The oxime was synthesized using aniline as the promoter with 1 equivalent of NH_2OH • HCl and sodium acetate and obtained as a colourless solid (0.148 g, 52 % overall yield from cyclohexanone). ^{1}H NMR (500 MHz, DMSO-d_6) δ: 10.46 (s, 1H), 5.25 (s, 1H), 4.15 (q, J = 7 Hz, 2H), 4.08 (q, J = 7.5 Hz, 2H), 3.13 (dd, J = 10.5, 4.5 Hz, 1H), 3.02-2.97 (m, 1H), 1.86-1.76 (m, 2H), 1.68-1.54 (m, 3H), 1.48-1.43 (m, 1H), 1.32-1.28 (m, 1H), 1.18 (t, J = 7.5 Hz, 3H), 1.14 (t, J = 7 Hz, 3H). ^{13}C{^{1}H} (101 MHz, DMSO-d_6) δ: 169.5, 169.3, 156.0, 80.8, 61.5, 61.1, 46.2, 27.6, 25.1, 24.4, 23.7,

13.9, 13.9. FTIR (neat): ν = 3540, 3362, 2990, 2944, 2916, 2861, 1745, 1730, 1663, 1474, 1441, 1398, 1276, 1227, 1149, 1115, 1061, 1018, 968, 929, 898, 862, 789, 699, 666, 635, 599. Melting point: 79 – 81 °C.

Diethyl (*E*)-2-hydroxy-2-(2-(hydroxyimino)-1-methylcyclohexyl)malonate (4b): To an oven-dried 50 mL round bottom flask equipped with a magnetic stir bar was added trimethyl((2-methylcyclohex-1-en-1-yl)oxy)silane[11] (1.00 g, 5.42 mmol, 1.1 equiv.) and diethyl mesoxalate (0.750 mL, 4.88 mmol, 1 equiv.) and DCM (20 mL). The mixture was cooled with an ice/water bath and stirred for 10 minutes. $BF_3 \bullet OEt_2$ (0.670 mL, 5.42 mmol, 1.1 equiv.) was added dropwise, then the mixture was stirred overnight while warming up to room temperature. The mixture was loaded onto Celite, then the volatiles were removed *in vacuo*. The residue was subjected to normal phase column chromatography to afford the hydroxy ketone which was used without further characterization. The oxime was synthesized using ammonium acetate as the promoter with 2 equivalents of $NH_2OH \bullet HCl$ and sodium acetate in ethanol at room temperature, and obtained as a colourless solid (0.047 g, 5 % overall yield from diethylmesoxalate). ^{1}H NMR (400 MHz, DMSO-d_6) δ: 10.50 (s, 1H), 5.44 (s, 1H), 4.16-4.05 (m, 4H), 2.96-2.91 (m, 1H), 2.27-2.19 (m, 1H), 2.00-1.92 (m, 1H), 1.69-1.62 (m, 2H), 1.53-1.45 (m, 1H), 1.38-1.29 (m, 5H), 1.20-1.14 (m, 6H). ^{13}C{^{1}H} (101 MHz, DMSO-d_6) δ: 169.8, 168.4, 161.1, 83.9, 60.9, 60.6, 45.6, 33.7, 23.0, 21.6, 21.1, 20.9, 13.8, 13.8. Melting point: 113 – 116 °C (decomp.).

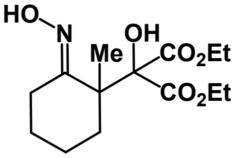

***rac*-((1*S*,2*R*,*E*)-1-hydroxy-3-(hydroxyimino)-2-(4-methoxybenzyl)-2-methylcyclohexyl)-(pyrrolidin-1-yl)methanone (*cis*-1r):** The ketone was synthesized according to general procedure B from 2-(4-methoxybenzyl)-2-methylcyclohexane-1,3-dione[7] (0.493 g, 2 mmol, 1 equiv.) with *N*-formylpyrrolidine[9] (0.200 g, 2 mmol, 1 equiv.) and freshly prepared LDA solution (6 mmol, 3 equiv.), and stirred for 3 h at -78 °C to afford the *cis*-isomer as the major product. The oxime was synthesized using ammonium acetate as the promoter with 4 equivalents of $NH_2OH \bullet HCl$ and sodium acetate at 60 °C for 5 days and obtained as a colourless solid (0.136 g, 19 % overall yield from dione, dr > 20:1). Crystals suitable for X-ray crystallography analysis were grown in a mixture of MeCN/DMSO using the slow evaporation method at room temperature. ^{1}H NMR (500 MHz, DMSO-d_6) δ: 9.90 (s, 1H), 6.84 (d, J = 8.2 Hz, 2H), 6.73 (d, J = 8.2 Hz, 2H), 5.32 (br, 1H), 3.83-3.73 (m overlap with s, 5H), 3.40-3.37 (m, 2H), 3.13-3.09 (m, 1H), 2.94 (d, J = 14.1 Hz, 1H), 2.70 (d, J = 14.1 Hz, 1H), 2.02-1.95 (m, 2H), 1.81-1.61 (m, 7H), 0.85 (s, 3H). ^{13}C{^{1}H} (126 MHz, DMSO-d_6) δ: 171.9, 158.8, 157.4, 131.7, 130.2, 112.7, 80.6, 54.8, 49.0, 47.8, 47.6, 37.6, 31.1, 26.8, 19.8, 19.3, 19.3, 16.5. Melting point: 205 – 206 °C.

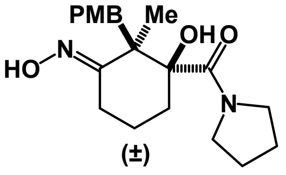

***rac*-((1*S*,2*S*,*E*)-1-hydroxy-3-(hydroxyimino)-2-(4-methoxybenzyl)-2-methylcyclohexyl)-(pyrrolidin-1-yl)methanone (*trans*-1r):** The ketone was synthesized in the same reaction as above and isolated as the minor isomer. The oxime was synthesized using ammonium acetate promoter with 4 equivalents of NH_2OH • HCl and sodium acetate at 60 °C for 5 days and obtained as a colourless solid (0.020 g, 3 % overall yield from dione, 4:1 rotamer, dr > 20:1). ^{1}H NMR (500 MHz, DMSO-d_6) δ: 10.36 (s, minor), 10.23 (s, 1H), 6.92 (d, J = 9 Hz, minor), 6.81 (d, J = 9 Hz, 2H), 6.75 (d, J = 8.5 Hz, minor), 6.71 (d, J = 8.5 Hz, 2H), 4.98 (s, 1H), 4.81 (d, J = 4.5 Hz, minor), 3.96-3.92 (m, 1H), 3.72-3.68 (overlap with minor, 4H), 3.44-3.37 (overlap with minor, 2H), 3.29-3.27 (m, minor), 3.19-3.16 (m, 1H), 3.15-3.12 (d, J = 14 Hz, 1H), 2.95-2.85 (m, minor), 2.87 (d, J = 13.5 Hz, minor), 2.72 (d, J = 13.5 Hz, minor), 2.66 (d, J = 14 Hz, 1H), 2.63-2.58 (m, 1H), 2.22-2.16 (m, minor), 2.11-2.04 (m, 1H), 1.79-1.55 (overlap with minor, 7H), 0.89-0.88 (overlapping s, 3H). ^{13}C{^{1}H} (126 MHz, DMSO-d_6) δ: 171.9, 160.0 (minor), 158.3, 157.4, 157.3 (minor), 131.2, 131.2 (minor), 130.4 (minor), 130.2, 81.8, 75.2 (minor), 54.9 (minor), 54.8, 48.1, 48.0, 46.9 (minor), 35.3 (minor), 31.4, 29.3, 26.9, 22.5, 21.3 (minor), 20.6 (minor), 19.8 (minor), 19.7, 19.3, 17.4. Melting point: 193 – 197 °C.

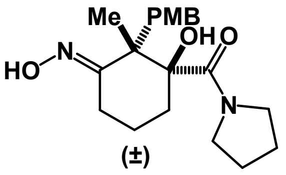

***rac*-((1*S*,2*R*,*E*)-2-Allyl-1-hydroxy-3-(hydroxyimino)-2-methylcyclohexyl)(pyrrolidin-1-yl)methanone (*cis*-1s):** The ketone was synthesized according to general procedure B from 2-allyl-2-methylcyclohexane-1,3-dione[7] (0.600 g, 3.61 mmol, 1 equiv.) with *N*-formylpyrrolidine[9] (0.358 g, 3.61 mmol, 1 equiv.) and freshly prepared LDA solution (10.83 mmol, 3 equiv.), and stirred for 3 h at -78 °C to afford the *cis*-isomer as the major product. The oxime was synthesized using ammonium acetate as the promoter with 3 equivalents of NH_2OH • HCl and sodium acetate at 50 °C for 4 days and obtained as a colourless solid upon trituration with methanol (1 mL) following column chromatography purification (0.128 g, 13 % overall yield from dione, dr > 20:1). ^{1}H NMR (500 MHz, DMSO-d_6) δ: 10.01 (s, 1H), 5.62-5.54 (m, 1H), 5.13 (s, 1H), 4.96-4.90 (m, 2H), 3.81-3.68 (m, 2H), 3.28-3.24 (m, 2H), 2.83 (dt, J = 14.5, 4.5 Hz, 1H), 2.52-2.48 (m, overlap with DMSO, 1H), 2.10-2.06 (m, 1H), 1.89-1.75 (m, 5H), 1.65-1.53 (m, 4H), 1.00 (s, 3H). ^{13}C{^{1}H} (126 MHz, DMSO-d_6) δ: 171.8, 159.7, 135.6, 116.3, 80.5, 44.7, 47.7, 47.6, 37.9, 31.4, 26.8, 22.5, 19.6, 19.1, 17.5. Melting point: 197 – 201 °C (decomp.).

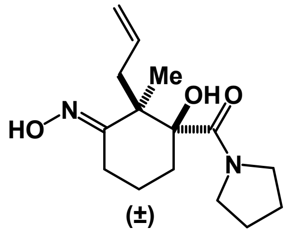

***rac*-((1*S*,2*S*,*E*)-2-Allyl-1-hydroxy-3-(hydroxyimino)-2-methylcyclohexyl)(pyrrolidin-1-yl)methanone (*trans*-1s):** The ketone was synthesized in the same reaction as above and isolated as the minor isomer. The oxime was synthesized using ammonium acetate promoter with 3 equivalents of NH_2OH • HCl and sodium acetate at 50 °C for 3 days and obtained as a colourless solid

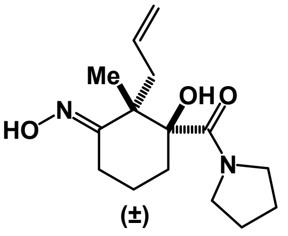

(0.029 g, 3 % overall yield from dione, dr 20:1). ^{1}H NMR (500 MHz, DMSO-d_6) δ: 10.27 (s, 1H), 5.50 (m, 1H), 4.94-4.88 (m, overlapped with OH, 3H), 3.93-3.89 (m, 1H), 3.72-3.64 (m, 1H), 3.32-3.27 (m, 2H), 3.05-3.01 (m, 1H), 2.66-2.62 (m, 1H), 2.47-2.44 (m, overlapped with DMSO, 1H), 2.08-2.03 (m, 1H), 1.86-1.51 (m, 8H), 0.89-0.88 (overlapping s, 3H). ^{13}C{^{1}H} (126 MHz, DMSO-d_6) δ: 171.6, 159.1, 135.1, 116.5, 81.5, 48.0, 48.0, 47.9, 39.7, 31.5, 26.9, 22.4, 19.4, 19.1, 17.6. Melting point: 179 – 182 °C (decomp.).

***rac-tert*-Butyl 3-((1*S*,2*R*,*E*)-2-hydroxy-6-(hydroxyimino)-1-methyl-2-(pyrrolidine-1-carbonyl)cyclohexyl)propanoate (*cis*-1t):** The ketone was synthesized according to general procedure B from *tert*-butyl 3-(1-methyl-2,6-dioxocyclohex-yl)propanoate[8] (0.485 g, 1.91 mmol, 1 equiv.) with *N*-formylpyrrolidine[9] (0.189 g, 1.91 mmol, 1 equiv.) and freshly prepared LDA solution (5.72 mmol, 3 equiv.), and stirred for 3 h at -78 °C to afford the *cis*-isomer as the major product. The oxime was synthesized using ammonium acetate as the promoter with 5 equivalents of NH_2OH • HCl and sodium acetate at 50 °C for 2 days and obtained as a colourless solid (0.133 g, 19 % overall yield from dione, dr > 20:1). ^{1}H NMR (500 MHz, DMSO-d_6) δ: 10.1 (s, 1H), 5.16 (s, 1H), 3.81-3.78 (m 1H), 3.71-3.66 (m, 1H), 3.27-3.24 (m, 2H), 2.75-2.70 (m, 1H), 2.02-1.74 (m, 8H), 1.66-1.52 (m, 5H), 1.37 (s, 9H), 0.97 (s, 3H). ^{13}C{^{1}H} (126 MHz, DMSO-d_6) δ: 172.9, 171.8, 159.9, 80.9, 79.3, 47.7, 47.6, 47.3, 31.2, 29.9, 27.9, 27.8, 26.8, 22.5, 19.4, 18.9, 17.2. Melting point: 202 – 204 °C (decomp.).

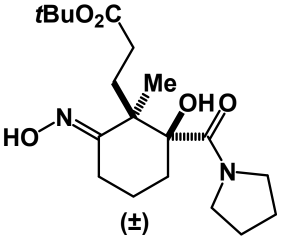

***rac-tert*-Butyl 3-((1*S*,2*S*,*E*)-2-hydroxy-6-(hydroxyimino)-1-methyl-2-(pyrrolidine-1-carbonyl)cyclohexyl)propanoate (*trans*-1t):** The ketone was synthesized in the same reaction as above and isolated as the minor isomer. The oxime was synthesized using ammonium acetate promoter with 5 equivalents of NH_2OH • HCl and sodium acetate at 50 °C for 5 days and obtained as a colourless solid (0.040 g, 6 % overall yield from dione, dr > 20:1). ^{1}H NMR (500 MHz, DMSO-d_6) δ: 10.38 (s, 1H), 5.00 (s, 1H), 3.91-3.87 (m, 1H), 3.70-3.64 (m, 1H), 3.31-3.27 (m, overlap with water, 2H), 3.00-2.98 (m, 1H), 2.46-2.41 (m, 1H), 2.03-1.92 (m, 2H), 1.83-1.54 (m, 10 H), 1.36 (s, 9H), 0.97 (s, 3H). ^{13}C{^{1}H} (126 MHz, DMSO-d_6) δ: 172.6, 171.6, 159.2, 81.5, 79.4, 48.0, 47.9, 47.9, 31.4, 30.2, 29.4, 27.7, 26.9, 22.4, 19.1, 19.0, 17.0. Melting point: 210 – 211 °C (decomp.).

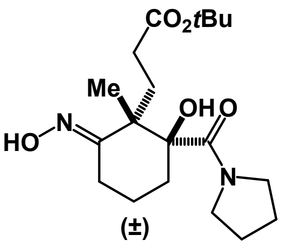

Synthesis of products

General procedure for the activation of iminologous diols:

C: To an oven-dried 4-dram vial equipped with a magnetic stir bar was added the

corresponding oxime (1 equiv.), then the vial was capped with a B24 septum and the vessel was purged of air with nitrogen. The vial was cooled with an ice/water bath, then THF (2 mL) was added, and the mixture was stirred for 5 minutes. *N*,*N*-Diisopropylethylamine (1.05 equiv., DIPEA) was added to the solution and stirred for 5 minutes at 0 °C. *p*-Toluenesulfonyl chloride (1.05 equiv, TsCl) was dissolved in THF (0.5 mL), then added dropwise to the stirring solution. The mixture was stirred for 1h, then potassium *tert*-butoxide (2.1 equiv., KO*t*Bu) was added, then the reaction mixture was stirred for 16 h while warming to room temperature. The mixture was loaded onto Celite, then the volatiles were evaporated *in vacuo*. The residue was subjected to normal phase column chromatography to afford the products.

D: To an oven-dried 4-dram vial equipped with a magnetic stir bar was added the corresponding oxime (1 equiv.) and KO*t*Bu (2.1 or 2.4 equiv.), then the vial was capped with a B24 septum and the vessel was purged of air with nitrogen. The vial was cooled with an ice/water bath, then THF or 1:1 THF/DMF (1-1.5 mL) was added, and the mixture was stirred for 15 minutes. TsCl (1.1 or 1.4 equiv.) was dissolved in THF (0.5 mL) then added dropwise to the stirring solution. The reaction mixture was stirred for 16 h while warming to room temperature. The mixture was loaded onto Celite, then the volatiles were evaporated *in vacuo*. The residue was subjected to normal phase (Me- or CF_3-bound) or reversed-phase (amide-bound) column chromatography to afford the products.

6-Methyl-5-oxoheptanenitrile (2a): The product was synthesized using general procedure C

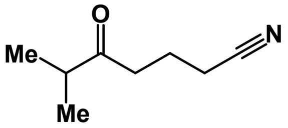

from **1a** (30 mg, 0.19 mmol, 1 equiv.) as the major product and obtained as a clear oil (6.2 mg, 23% yield). ^{1}H NMR (400 MHz, $CDCl_3$) δ: 2.67-2.57 (overlapping t and p, t: J = 7.2 Hz, p: J = 6.8 Hz, 3H), 2.41 (t, J = 6.8 Hz, 2H), 1.91 (p, J = 6.4 Hz, 2H), 1.11 (d, J = 6.8 Hz, 6H). $^{13}C\{^1H\}$ (101 MHz, $CDCl_3$) δ: 213.1, 119.4, 41.1, 38.0, 19.4, 18.3, 16.6. HRMS (DART) m/z calc'd for $C_8H_{14}NO$ $[M+H]^+$: 140.10699; found: 140.10632.

4-(3,3-Dimethyloxiran-2-yl)butanenitrile (3a): The product was synthesized using general

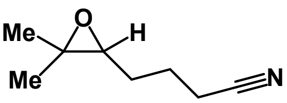

procedure C from **1a** (30 mg, 0.19 mmol, 1 equiv.) as the minor product and obtained as a clear oil (2.1 mg, 8% yield). ^{1}H NMR (400 MHz, $CDCl_3$) δ: 2.72 (dd, J = 8, 4.4 Hz, 1H), 2.46-2.41 (m, 2H), 1.91-1.77 (m, 3H), 1.62-1.55 (m, 2H), 1.32 (s, 3H), 1.28 (s, 3H). $^{13}C\{^1H\}$ (101 MHz, $CDCl_3$) δ: 119.5, 63.3, 58.4, 27.9, 24.8, 23.0, 18.9, 17.1. HRMS (DART) m/z calc'd for $C_8H_{17}N_2O$ $[M+NH_4]^+$: 157.13354; found: 157.13305.

6,6-Dimethyl-5-oxoheptanenitrile (2b): The product was synthesized using general

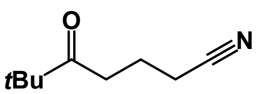

procedure D from **1b** (17 mg, 0.1 mmol, 1 equiv.) with KO*t*Bu (2.1 equiv.) and TsCl (1.1 equiv.) in THF (1 mL) as the minor product and

obtained as a clear oil (1.8 mg, 14% yield). ^{1}H NMR (400 MHz, $CDCl_3$) δ: 2.69 (t, J = 6.8 Hz, 2H), 2.41 (t, J = 6.4 Hz, 2H), 1.91 (p, J = 6.4 Hz, 2H), 1.16 (s, 9H). ^{13}C{^{1}H} (101 MHz, $CDCl_3$) δ: 214.5, 119.5, 44.3, 34.5, 26.5, 19.7, 16.6. The chemical shifts are in agreement with literature values.[12]

4-(2,3,3-Trimethyloxiran-2-yl)butanenitrile (3b): The product was synthesized using general procedure D from **1b** (17 mg, 0.1 mmol, 1 equiv.) with KO*t*Bu (2.1 equiv.) and TsCl (1.1 equiv.) in THF (1 mL) as the major product and obtained as a clear oil (2.2 mg, 14% yield). ^{1}H NMR (400 MHz, $CDCl_3$) δ: 2.45-2.35 (m, 2H), 1.88-1.61 (m, 6H), 1.34-1.31 (overlapping s, 9H). ^{13}C{^{1}H} (101 MHz, $CDCl_3$) δ: 119.5, 63.6, 62.3, 33.6, 21.9, 21.4, 21.1, 18.6, 17.5. HRMS (DART) m/z calc'd for $C_9H_{15}NO$ $[M+H]^+$: 154.12264; found: 154.12268.

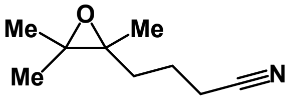

***N*,*N*-Dibenzyl-2-(3-cyanopropyl)-3,3-dimethyloxirane-2-carboxamide (3c):** The product was synthesized using general procedure D from **1c** (57.1 mg, 0.15 mmol, 1 equiv.) with KO*t*Bu (2.4 equiv.) and TsCl (1.4 equiv.) in THF (1.5 mL) and obtained as a yellow oil (47.7 mg, 88% yield). ^{1}H NMR (400 MHz, $CDCl_3$, exists as two rotamers, major conformer integrated) δ: 7.44-7.18 (m, 10H, overlap with $CHCl_3$ and minor rotamer), 5.10 (d, J = 13.6 Hz, minor), 4.88 (d, J = 15.6 Hz, 1H), 4.70 (d, J = 14.8 Hz, 1H), 4.50 (d, J = 16 Hz, minor), 4.44 (d, J = 15.6 Hz, 1H), 4.28-4.22 (overlapping d, J = 16.4, 14.4, 1H, overlap with minor rotamer), 4.01 (d, J = 14.8 Hz, minor), 2.58-2.51 (m, minor), 2.41-2.38 (m, 2H), 2.06-1.82 (m, 4H, overlap with minor rotamer), 1.42-1.41 (overlapping s, 6H), 1.35 (s, minor), 1.22 (s, minor). ^{13}C{^{1}H} (101 MHz, $CDCl_3$, exists as two rotamers) δ: 170.1 (minor), 169.6, 136.8, 136.5, 136.4 (minor), 135.1 (minor), 129.4, 128.9, 128.8, 128.7, 128.7 (minor), 128.3 (minor), 128.0 (minor), 127.9 (minor), 127.8, 127.8 (minor), 126.9 (minor), 119.6 (minor), 119.2, 67.7, 66.8 (minor), 63.6, 62.7 (minor), 49.2, 49.0 (minor), 47.2, 46.6 (minor), 31.1, 28.8 (minor), 23.4, 21.9 (minor), 21.9 (minor), 21.8, 19.9, 19.4 (minor), 17.2, 16.9 (minor). HRMS (ESI) m/z calc'd for $C_{23}H_{27}N_2O_2$ $[M+H]^+$: 363.2067; found: 363.2053.

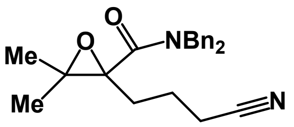

2-(3-Cyanopropyl)-*N*,*N*,3,3-tetramethyloxirane-2-carboxamide (3d): The product was synthesized using general procedure D from **1d** (34.2 mg, 0.15 mmol, 1 equiv.) with KO*t*Bu (2.4 equiv.) and TsCl (1.4 equiv.) in THF (1.5 mL) and obtained as a clear oil which solidifies to become a colourless solid upon sitting in a -20 °C freezer (21.6 mg, 68% yield). ^{1}H NMR (500 MHz, $CDCl_3$, two conformers reported as one species) δ: 3.11-2.91 (overlapping s, 6H), 2.53-2.50 (m, 1H), 2.39-2.38 (m, 1H), 2.09-2.03 (m, 1H), 1.94-1.66 (m, 3H), 1.39-1.36 (overlapping s, 3H), 1.24-1.22 (overlapping s, 3H). ^{13}C{^{1}H} (126 MHz, $CDCl_3$, exists as two rotamers) δ: 169.4 (minor),

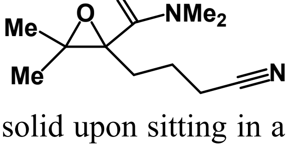

169.2, 119.6 (minor), 119.2, 67.8, 66.6 (minor), 62.9, 62.3 (minor), 36.9, 36.5 (minor), 35.6, 34.8 (minor), 30.8, 28.4 (minor), 23.0, 21.8, 21.7 (minor), 21.5 (minor), 19.8, 19.4 (minor), 17.3, 16.9 (minor). FTIR (neat): ν = 3580, 2971, 2949, 2938, 2238, 1639, 1499, 1452, 1399, 1379, 1274, 1154, 1108, 1090, 907, 879, 834, 768, 743, 696, 582. HRMS (DART) m/z calc'd for $C_{11}H_{19}N_2O_2$ $[M+H]^+$: 211.14410; found: 211.14447. Melting point: 49 – 50 °C.

4-(3,3-Dimethyl-2-(4-phenylpiperazine-1-carbonyl)oxiran-2-yl)butanenitrile (3e): The

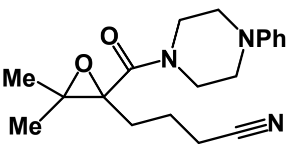

product was synthesized using general procedure D from **1e** (51.8 mg, 0.15 mmol, 1 equiv.) with KO*t*Bu (2.4 equiv.) and TsCl (1.4 equiv.) in THF/DMF (1.5 mL) and obtained as an orange oil (21.6 mg, 87% yield). ^{1}H NMR (500 MHz, $CDCl_3$, two conformers reported as one species) δ: 7.30-7.26 (m, 2H), 6.95-6.89 (m, 3H), 4.01-3.79 (m, 2H), 3.70-3.47 (m, 2H), 3.29-3.02 (m, 4H), 2.57-2.39 (m, 2H), 2.15-2.05 (m, 1H), 1.95-1.74 (m, 3H), 1.43-1.40 (overlapping s, 3H), 1.30-1.29 (s, 3H). ^{13}C{^{1}H} (126 MHz, $CDCl_3$, exists as two rotamers) δ: 168.1 (minor), 168.0, 150.9, 150.7 (minor), 129.4 (minor), 129.4, 121.2 (minor), 120.8, 119.5 (minor), 119.1, 117.1 (minor), 116.8, 67.7, 66.4 (minor), 63.2, 62.4 (minor), 50.3 (minor), 50.1, 49.8, 45.3 (minor), 45.2 (minor), 42.1, 41.6 (minor), 30.8, 28.9, 23.0, 22.1 (minor), 21.8, 21.6 (minor), 19.8, 19.5 (minor), 17.3, 16.9 (minor). HRMS (DART) m/z calc'd for $C_{19}H_{26}N_3O_2$ $[M+H]^+$: 328.20195; found: 328.20216.

4-(3,3-Dimethyl-2-(pyrrolidine-1-carbonyl)oxiran-2-yl)butanenitrile (3f): The product

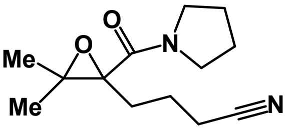

was synthesized using general procedure D from **1f** (38.1 mg, 0.15 mmol, 1 equiv.) with KO*t*Bu (2.4 equiv.) and TsCl (1.4 equiv.) in THF (1.5 mL) and obtained as a colourless solid (30.4 mg, 86% yield). Crystals suitable for X-ray crystallography analysis were grown in a mixture of hexanes/DCM using the slow evaporation method at room temperature. ^{1}H NMR (500 MHz, $CDCl_3$) δ: 3.72-3.32 (m, 4H), 2.54-2.34 (m, 2H), 2.18-2.13 (m, 1H), 1.98-1.70 (m, 7H), 1.36 (s, 3H), 1.23 (s, 3H). ^{13}C{^{1}H} (126 MHz, $CDCl_3$, exists as two rotamers) δ: 168.0, 119.6 (minor), 119.3, 68.4, 67.4 (minor), 62.6, 61.9 (minor), 46.7, 46.5 (minor), 46.3, 45.6 (minor), 30.4, 27.8 (minor), 26.5, 23.5 (minor), 22.5, 21.8, 21.7 (minor), 21.4 (minor), 19.9, 19.7 (minor), 17.3, 16.9 (minor). HRMS (DART) m/z calc'd for $C_{13}H_{21}N_2O_2$ $[M+H]^+$: 237.15975; found: 237.16013. Melting point: 74 – 75 °C (decomp.).

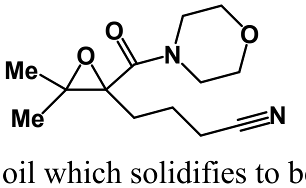

4-(3,3-Dimethyl-2-(morpholine-4-carbonyl)oxiran-2-yl)butanenitrile (3g): The product was synthesized using general procedure D from **1g** (40.5 mg, 0.15 mmol, 1 equiv.) with KO*t*Bu (2.4 equiv.) and TsCl (1.4 equiv.) in THF (1.5 mL) and obtained as a clear oil which solidifies to become a colourless solid upon sitting in a -20 °C freezer (31.7 mg, 84%

yield). ^{1}H NMR (500 MHz, $CDCl_3$, two conformers reported as one species) δ: 3.92-3.29 (m, 8H), 2.56-2.35 (m, 2H), 2.10-1.69 (m, 4H), 1.38-1.36 (overlapping s, 3H), 1.27-1.26 (overlapping s, 3H). $^{13}C\{^1H\}$ (126 MHz, $CDCl_3$, exists as two rotamers) δ: 168.1 (minor), 167.9, 119.5 (minor), 119.1, 67.6, 67.1, 67.0, 67.0 (minor), 66.7 (minor), 66.3 (minor), 63.1, 62.2 (minor), 46.0, 45.8 (minor), 42.4, 41.9 (minor), 30.8, 28.7 (minor), 23.0, 22.0 (minor), 21.8, 21.6 (minor), 19.7, 19.4 (minor), 17.3, 16.9 (minor). HRMS (DART) m/z calc'd for $C_{13}H_{21}N_2O_3$ $[M+H]^+$: 253.15467; found: 253.15507. Melting point: 81 – 82 °C.

2-(3-Cyanopropyl)-*N*,3,3-trimethyl-*N*-phenyloxirane-2-carboxamide (3h): The product was synthesized using general procedure D from **1h** (43.6 mg, 0.15 mmol, 1 equiv.) with KO*t*Bu (2.4 equiv.) and TsCl (1.4 equiv.) in THF (1.5 mL) and obtained as an orange oil (12.9 mg, 32% yield). ^{1}H NMR (500 MHz, $CDCl_3$, multiple conformers reported as one species) δ: 7.44-7.14 (m, overlap with $CHCl_3$, 5H), 3.47-3.22 (m, 3H), 2.48-2.32 (m, 2H), 1.97-1.75 (m, 3H), 1.47-1.17 (m, 7H). $^{13}C\{^1H\}$ (126 MHz, DMSO-d_6, exists as two rotamers) δ: 168.5, 168.5 (minor), 143.3 (minor), 142.6, 128.9 (minor), 128.9, 126.5 (minor), 126.5, 126.0, 120.4, 120.4 (minor), 67.3 (minor), 66.5, 62.7 (minor), 62.0, 37.5 (minor), 37.2, 29.9 (minor), 27.9, 23.0 (minor), 21.9, 21.6, 21.2 (minor), 19.3 (minor), 18.9, 16.2 (minor), 16.0. HRMS (DART) m/z calc'd for $C_{16}H_{21}N_2O_2$ $[M+H]^+$: 273.15975; found: 273.16057.

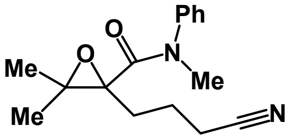

2-(3-Cyanopropyl)-*N*-(3-(10,11-dihydro-5*H*-dibenzo[*b*,*f*]azepin-5-yl)propyl)-*N*,3,3-trimethyloxirane-2-carboxamide (3i): The product was synthesized using general procedure D from **1i** (67.4 mg, 0.15 mmol, 1 equiv.) with KO*t*Bu (2.4 equiv.) and TsCl (1.4 equiv.) in THF/DMF (1.5 mL) and obtained as a yellow oil (52.2 mg, 81% yield). ^{1}H NMR (500 MHz, DMSO-d_6, multiple conformers reported as one species) δ: 7.15-7.08 (m, 6H), 6.95-6.89 (m, 2H), 3.87-3.10 (m, 9H), 2.90-2.65 (multiple s, 3H), 2.48-2.32 (m, 2H), 1.95-1.45 (m, 6H), 1.29-1.24 (overlapping s, 3H), 1.06-1.00 (overlapping s, 3H). $^{13}C\{^1H\}$ (126 MHz, DMSO-d_6, exists as four conformers) δ: 168.2, 168.1, 168.0, 167.8, 148.0, 148.0, 148.0, 147.9, 133.7, 133.7, 133.5, 129.8, 129.7, 129.7, 129.7, 126.4, 126.3, 126.3, 122.6, 122.4, 122.4, 122.4, 120.3, 120.3, 120.3, 120.2, 119.7, 119.7, 119.7, 67.1, 67.1, 66.3, 66.2, 62.1, 61.9, 61.5, 61.5, 47.2, 47.2, 47.1, 46.4, 46.3, 44.6, 43.9, 34.1, 34.0, 32.3, 31.5, 31.5, 31.5, 31.4, 30.2, 30.0, 28.4, 28.0, 26.2, 25.5, 24.9, 24.9, 22.7, 22.6, 21.5, 21.4, 21.3, 21.3, 21.3, 21.1, 19.2, 18.8, 18.8, 16.1, 16.1, 15.8, 15.7. HRMS (DART) m/z calc'd for $C_{26}H_{32}N_3O_2$ $[M+H]^+$: 418.24890; found: 418.24917.

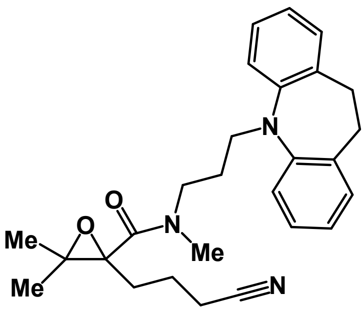

4-(3,3-Dimethyl-2-(trifluoromethyl)oxiran-2-yl)butanenitrile (3j): The product was

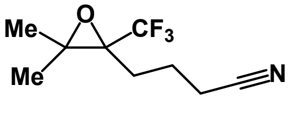

synthesized using general procedure D from **1j** (33.8 mg, 0.15 mmol, 1 equiv.) with KHMDS (2.2 equiv.) and TsCl (1.1 equiv.) in THF (1.5 mL) and obtained as a clear oil (7.5 mg, 24% yield). ^{1}H NMR (500 MHz, CDCl$_3$) δ: 2.42 (t, *J* = 6.6 Hz, 2H), 2.09-2.03 (m, 1H), 1.92-1.81 (m, 3H), 1.49 (s, 3H), 1.42 (s, 3H). ^{13}C{^{1}H} (126 MHz, CDCl$_3$) δ: 124.8 (q, *J* = 280 Hz), 119.0, 63.7 (q, *J* = 34 Hz), 62.3, 26.6 (q, *J* = 1.3 Hz), 22.0, 21.0, 20.0 (q, *J* = 2.5 Hz), 17.4. ^{19}F NMR (376 MHz, CDCl$_3$) δ: -67.12. HRMS (DART) m/z calc'd for $C_9H_{12}NOF_3$ [M+H]$^+$: 208.09438; found: 208.09431.

4-(2-(Pyrrolidine-1-carbonyl)-1-oxaspiro[2.2]pentan-2-yl)butanenitrile (3k): The product

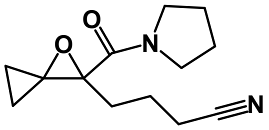

was synthesized using general procedure D from **1k** (37.8 mg, 0.15 mmol, 1 equiv.) with KO*t*Bu (2.4 equiv.) and TsCl (1.4 equiv.) in THF (1.5 mL) and obtained as a clear oil (8.2 mg, 23% yield). ^{1}H NMR (400 MHz, CDCl$_3$) δ: 3.70-3.64 (m, 1H), 3.57-3.46 (m, 3H), 2.43 (t, *J* = 7.1 Hz, 2H), 2.34-2.27 (m, 1H), 2.03-1.78 (m, 7H), 1.12-1.05 (m, 4H). ^{13}C{^{1}H} (101 MHz, CDCl$_3$) δ: 166.9, 119.2, 64.9, 62.8, 46.5, 46.4, 31.2, 26.4, 23.5, 20.9, 17.1, 2.6, 2.5. HRMS (DART) m/z calc'd for $C_{13}H_{19}N_2O_2$ [M+H]$^+$: 235.14410; found: 235.14420.

4-(2-(Pyrrolidine-1-carbonyl)-1-oxaspiro[2.4]heptan-2-yl)butanenitrile (3l): The product

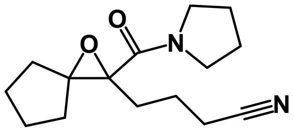

was synthesized using general procedure D from **1l** (42.1 mg, 0.15 mmol, 1 equiv.) with KO*t*Bu (2.4 equiv.) and TsCl (1.4 equiv.) in THF/DMF (1.5 mL) and obtained as a yellow oil (31.6 mg, 80% yield). ^{1}H NMR (400 MHz, CDCl$_3$) δ: 3.60-3.40 (m, 4H), 2.43-2.40 (m, 2H), 2.30-2.23 (m, 1H), 1.98-1.48 (m, 15H). ^{13}C{^{1}H} (101 MHz, CDCl$_3$) δ: 167.9, 119.3, 73.5, 66.6, 46.8, 46.3, 31.4, 30.3, 26.6, 25.2, 25.1, 23.6, 21.8, 17.2. HRMS (DART) m/z calc'd for $C_{15}H_{23}N_2O_2$ [M+H]$^+$: 263.17540; found: 263.17498.

4-(2-(Pyrrolidine-1-carbonyl)-1-oxaspiro[2.4]hept-5-en-2-yl)butanenitrile (3m): The

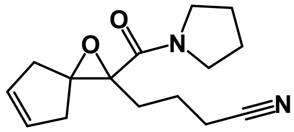

product was synthesized using general procedure D from **1m** (41.8 mg, 0.15 mmol, 1 equiv.) with KO*t*Bu (2.4 equiv.) and TsCl (1.4 equiv.) in THF/DMF (1.5 mL) and obtained as a yellow oil (27.2 mg, 70% yield). ^{1}H NMR (400 MHz, CDCl$_3$) δ: 5.76 (s, 2H), 3.61-3.45 (m, 4H), 2.78 (d, *J* = 18 Hz, 1H), 2.60 (br, 1H), 2.46-2.22 (m, 5H), 1.96-1.74 (m, 6H), 1.63-1.53 (m, 1H). ^{13}C{^{1}H} (101 MHz, CDCl$_3$) δ: 167.4, 128.7, 128.0, 119.2, 71.1, 66.2, 46.8, 46.2, 37.6, 37.0, 30.8, 26.5, 23.5, 21.7, 17.1. HRMS (DART) m/z calc'd for $C_{15}H_{21}N_2O_2$ [M+H]$^+$: 261.15975; found: 261.16008.

3-(3,3-Dimethyl-2-(pyrrolidine-1-carbonyl)oxiran-2-yl)propanenitrile (3n): The product

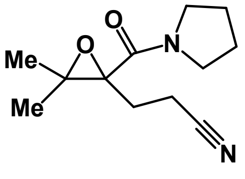

was synthesized using general procedure D from **1n** (25.0 mg, 0.104 mmol, 1 equiv.) with KO*t*Bu (2.4 equiv.) and TsCl (1.4 equiv.) in THF (1

mL) and obtained as a yellow oil (13.2 mg, 57% yield). ^{1}H NMR (500 MHz, $CDCl_3$) δ: 3.68-3.46 (m, 4H), 2.66-2.61 (m, 1H), 2.52-2.43 (m, 2H), 1.97-1.87 (m, 4H), 1.76-1.74 (br, 1H), 1.39 (s, 3H), 1.27 (s, 3H). ^{13}C{^{1}H} (126 MHz, $CDCl_3$) δ: 167.3, 119.0, 67.3, 62.9, 47.1, 46.6, 27.7, 26.5, 23.5, 22.1, 20.0, 13.9. HRMS (DART) m/z calc'd for $C_{12}H_{19}N_2O_2$ [M+H]$^+$: 223.14410; found: 223.14380.

5-(3,3-Dimethyl-2-(pyrrolidine-1-carbonyl)oxiran-2-yl)pentanenitrile (3o): The product was synthesized using general procedure D from **1o** (40.3 mg, 0.15 mmol, 1 equiv.) with KO*t*Bu (2.4 equiv.) and TsCl (1.4 equiv.) in THF (1.5 mL) and obtained as a clear oil (12.2 mg, 32% yield). ^{1}H NMR (500 MHz, $CDCl_3$) δ: 3.70-3.34 (m, 4H), 2.38-2.30 (m, 2H), 2.18-2.12 (m, 1H), 1.93-1.52 (m, 9H), 1.35 (s, 3H), 1.24 (s, 3H). ^{13}C{^{1}H} (126 MHz, $CDCl_3$) δ: 168.4, 119.5, 69.0, 62.5, 46.8, 46.3, 30.7, 26.6, 25.6, 24.7, 23.6, 22.5, 20.0, 17.2. HRMS (DART) m/z calc'd for $C_{14}H_{23}N_2O_2$ [M+H]$^+$: 251.17540; found: 251.17548.

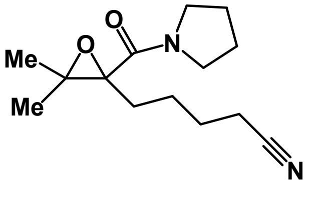

(9,9-Dimethyl-7-oxa-8-azabicyclo[4.2.1]non-1(8)-en-6-yl)(pyrrolidin-1-yl)methanone (3oa): The product was synthesized in the same reaction as **3o**, but further purified by normal phase column chromatography and obtained as a clear oil (6.4 mg, 18%). ^{1}H NMR (500 MHz, $CDCl_3$) δ: 3.88-3.78 (m, 2H), 3.53-3.45 (m, 2H), 2.66-2.62 (m, 1H), 2.52-2.45 (m, 1H), 1.99-1.75 (m, 9H), 1.41-1.37 (overlap with s, 4H), 1.21 (s, 3H). ^{13}C{^{1}H} (126 MHz, $CDCl_3$) δ: 171.7, 168.0, 96.5, 51.2, 47.8, 47.4, 33.6, 27.0, 26.8, 26.3, 24.7, 23.4, 22.0, 18.8. HRMS (DART) m/z calc'd for $C_{14}H_{23}N_2O_2$ [M+H]$^+$: 251.17540; found: 251.17614.

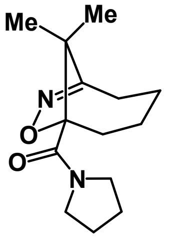

4-(3,3-Dimethyl-2-(pyrrolidine-1-carbonyl)oxiran-2-yl)-3,3-dimethylbutanenitrile (3p): The product was synthesized using general procedure D from **1p** (42.4 mg, 0.15 mmol, 1 equiv.) with KO*t*Bu (2.4 equiv.) and TsCl (1.4 equiv.) in THF/DMF (1.5 mL) and obtained as a clear oil which solidifies to become a colourless solid upon sitting in a -20 ˚C freezer (14.8 mg, 37% yield). ^{1}H NMR (400 MHz, $CDCl_3$, two conformers reported as one species) δ: 3.70-3.27 (m, 4H), 2.66-2.55 (m, 1H), 2.42-2.15 (m, 2H), 2.07-1.52 (m, 5H), 1.40-1.38 (two s, 3H), 1.23-1.20 (two s, 3H), 1.19-1.15 (two s, 3H), 1.13 (s, 3H). ^{13}C{^{1}H} (101 MHz, $CDCl_3$, exists as two rotamers in equal ratio) δ: 169.0, 168.8, 118.9, 118.6, 67.0, 65.8, 62.0, 60.4, 47.4, 47.0, 46.9, 45.8, 40.1, 39.0, 34.8, 33.8, 30.5, 29.5, 29.0, 28.6, 28.4, 26.6, 26.5, 23.9, 23.3, 21.6, 21.1, 20.8, 19.7. HRMS (DART) m/z calc'd for $C_{15}H_{25}N_2O_2$ [M+H]$^+$: 265.19105; found: 265.19116. Melting point: 74 – 76 ˚C.

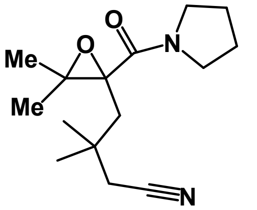

***tert*-Butyl (cyanomethyl)((3,3-dimethyl-2-(pyrrolidine-1-carbonyl)oxiran-2-yl)methyl)-**

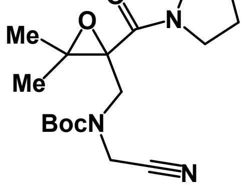

carbamate (3q): The product was synthesized using general procedure D from **1q** (21.3 mg, 0.060 mmol, 1 equiv.) with KO*t*Bu (2.4 equiv.) and TsCl (1.4 equiv.) in THF/DMF (1 mL) and obtained as a clear oil (15.0 mg, 74% yield). ^{1}H NMR (500 MHz, $CDCl_3$, multiple conformers reported as one species) δ: 3.68-3.46 (m, 4H), 2.66-2.61 (m, 1H), 2.52-2.43 (m, 2H), 1.97-1.87 (m, 4H), 1.76-1.74 (br, 1H), 1.39 (s, 3H), 1.27 (s, 3H). $^{13}C\{^{1}H\}$ (126 MHz, $CDCl_3$, exists as two rotamers) δ: 167.0, 165.6 (minor), 154.0, 116.2 (minor), 116.0, 82.4, 82.3 (minor) 67.6, 67.2 (minor), 62.3 (minor), 60.2, 48.4, 46.7, 46.4 (minor), 46.1, 45.7, 37.4 (minor), 36.4 (minor), 35.5 (minor), 28.2, 28.1 (minor), 26.4, 26.3 (minor), 23.9, 23.6 (minor), 22.3 (minor), 21.8, 20.0 (minor), 19.5. HRMS (DART) m/z calc'd for $C_{17}H_{28}N_3O_4$ $[M+H]^+$: 338.20743; found: 338.20760.

Diethyl 4,5,6,7-tetrahydrobenzo[*c*]isoxazole-3,3(3a*H*)-dicarboxylate (5a): The product was

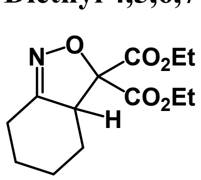

synthesized using general procedure C from **4a** (43.1 mg, 0.15 mmol, 1 equiv.) as the major product and obtained as a colourless solid (21.1 mg, 52% yield). Crystals suitable for X-ray crystallography analysis were grown in $CDCl_3$ using the slow evaporation method at room temperature. ^{1}H NMR (400 MHz, $CDCl_3$) δ: 4.35-4.18 (m, 4H), 3.93 (dd, J = 12.6, 6 Hz, 1H), 2.80-2.75 (m, 1H), 2.19-2.04 (m, 2H), 2.00-1.95 (m, 1H), 1.91-1.85 (m, 1H), 1.49-1.15 (m, 9H). $^{13}C\{^{1}H\}$ (101 MHz, $CDCl_3$) δ: 167.1, 166.5, 159.5, 89.5, 62.9, 62.3, 53.9, 28.8, 25.7, 25.1, 24.3, 14.3, 14.0. FTIR (neat): ν = 2975, 2938, 2865, 1761, 1729, 1464, 1449, 1304, 1269, 1240, 1203, 1134, 1062, 1026, 943, 856, 826, 767, 743, 689, 657. HRMS (DART) m/z calc'd for $C_{13}H_{20}NO_5$ $[M+H]^+$: 270.13383; found: 270.13393. Melting point: 61 – 63 °C (decomp.).

Diethyl 3a-methyl-4,5,6,7-tetrahydrobenzo[*c*]isoxazole-3,3(3a*H*)-dicarboxylate (5b): To

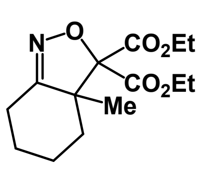

an oven-dried 4-dram vial equipped with a magnetic stir bar was added **4b** (41.9 mg, 0.14 mmol, 1 equiv.), then the vial was capped with a B24 septum and the vessel was purged of air with nitrogen. The vial was cooled with an ice/water bath, then DCM (1.3 mL) was added, and the mixture was stirred for 5 minutes. DIPEA (0.051 mL, 0.293 mmol, 2.1 equiv.) was added to the solution and stirred for 5 minutes at 0 °C. Trifluoromethanesulfonic anhydride (0.146 mL, 0.146 mmol, 1.1 equiv., 1 M in DCM) was added dropwise, then the reaction mixture was stirred for 16 h while warming to room temperature. The mixture was loaded onto Celite, then the volatiles were evaporated *in vacuo*. The residue was subjected to reversed-phase column chromatography to afford the product as a clear oil (31.5 mg, 80% yield). ^{1}H NMR (500 MHz, $CDCl_3$) δ:4.31-4.18 (m, 4H), 2.71-2.66 (m, 1H), 2.21-2.15 (m, 1H), 1.98-1.92 (m, 2H), 1.78-1.67 (m, 2H), 1.59-1.51 (m, 1H), 1.42-1.34 (m, 1H), 1.32-1.25 (overlapping t and s, J = 7.1, 5.8 Hz, 9H). $^{13}C\{^{1}H\}$ (126 MHz, $CDCl_3$) δ: 166.4, 165.8, 163.7, 93.3, 62.2, 62.2, 56.4, 34.1, 25.5, 23.3, 21.3, 18.9, 14.1, 14.1. HRMS (DART) m/z calc'd for $C_{14}H_{22}NO_5$ $[M+H]^+$: 284.14925; found: 284.14989.

***N*,*N*-Dibenzyl-5-cyano-2-hydroxy-2-(prop-1-en-2-yl)pentanamide (6):** To an oven-dried 1-dram vial equipped with a stir bar was added **3c** (18.1 mg, 0.05 mmol, 1 equiv.), then the vial was capped with a B19 septum and the vessel was purged of air with nitrogen. THF (0.5 mL) was added, then the solution was stirred at room temperature for 5 minutes before the dropwise addition of SmI_2 (0.2 mL, 0.10 mmol, 2 equiv., 0.5 M in THF). The mixture was stirred for 16 h at room temperature then loaded onto Celite. The volatiles were removed *in vacuo*, then the residue was subjected to reversed-phase column chromatography to afford the product as a clear oil (4.4 mg, 24%, 6 mg of **3c** recovered). ^{1}H NMR (500 MHz, $CDCl_3$) δ: 7.42-7.40 (m, 6H), 7.21-7.12 (m, 4H), 5.10 (s, 1H), 5.04-5.02 (m, 2H), 4.84 (d, *J* = 14.6 Hz, 1H), 4.76 (d, *J* = 15.8 Hz, 1H), 4.59 (d, *J* = 15.8 Hz, 1H), 4.33 (d, *J* = 14.6 Hz, 1H), 2.30-2.19 (m, 2H), 2.09-2.04 (m, H), 1.97-1.92 (m, 2H), 1.73 (s, 3H), 1.54-1.47 (m, 1H). ^{13}C{^{1}H} (126 MHz, $CDCl_3$) δ: 173.8, 146.1, 136.5, 135.8, 129.3, 129.1, 128.8, 128.0, 126.8, 119.5, 112.9, 78.0, 50.1, 49.6, 34.9, 20.5, 19.1, 17.4. HRMS (DART) m/z calc'd for $C_{23}H_{27}N_2O_2$ $[M+H]^+$: 363.20670; found: 363.20645.

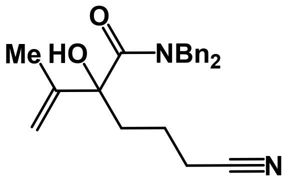

6-Fluoro-5-hydroxy-6-methyl-5-(morpholine-4-carbonyl)heptanenitrile (7a): To an oven-dried 1-dram vial equipped with a stir bar was added **3g** (25.2 mg, 0.10 mmol, 1 equiv.), then the vial was capped with a B19 septum and the vessel was purged of air with nitrogen. DCM (1 mL) was added, then the solution was stirred at room temperature for 5 minutes before the addition of $BF_3 \bullet OEt_2$ (14 µL, 0.11 mmol, 1.1 equiv.). The mixture was stirred for 2 h at room temperature then loaded onto Celite. The volatiles were removed *in vacuo*, then the residue was subjected to reversed-phase column chromatography to afford the product as a clear oil (10.1 mg, 37%). ^{1}H NMR (500 MHz, $CDCl_3$) δ: 5.36 (br, 1H), 4.16-3.37 (m, 8H), 2.57-2.25 (m, 3H), 1.93-1.81 (m, 2H), 1.49-1.27 (overlapping m, 7H). ^{13}C{^{1}H} (126 MHz, $CDCl_3$) δ: 171.7, 119.4, 98.2 (dd, *J* = 178, 4 Hz), 80.1 (d, *J* = 26 Hz), 67.0, 66.4, 47.7, 45.3, 31.8, 23.4 (d, *J* = 24 Hz), 23.0 (d, *J* = 25 Hz), 20.4, 17.6. ^{19}F NMR (376 MHz, $CDCl_3$) δ: -143.84 (m). FTIR (neat): ν = 3369, 2990, 2952, 2863, 2255, 1616, 1468, 1438, 1390, 1372, 1234, 1116, 1067, 1039, 919, 848, 734, 609. HRMS (DART) m/z calc'd for $C_{13}H_{22}N_2O_3F$ $[M+H]^+$: 273.16090; found: 273.16086.

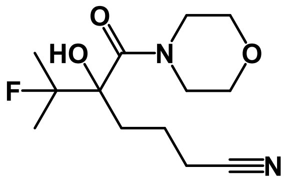

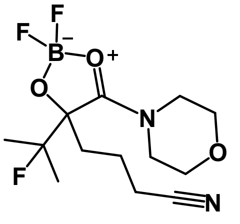

4-(2,2-Difluoro-4-(2-fluoropropan-2-yl)-5-morpholino-2,4-dihydro-$1\lambda^3,3,2\lambda^4$-dioxaborol-4-yl)butanenitrile (7b): The product was isolated in the same reaction as described above as a colourless solid (15.4 mg, 48%). Crystals suitable for x-ray crystallography analysis were grown in a mixture of $CDCl_3$ and hexanes using the slow evaporation method at room

temperature. ^{1}H NMR (500 MHz, $CDCl_3$) δ: 4.17-4.10 (m, 2H), 3.97-3.88 (m, 2H), 3.80-3.74 (m, 4H), 2.60 (dt, J = 17.2, 5.4 Hz, 1H), 2.40-2.34 (m, 1H), 2.25-2.19 (m, 1H), 2.13-2.02 (m, 2H), 1.53 (d, J = 23.2 Hz, 3H), 1.46 (d, J = 22.8 Hz, 3H), 1.38-1.30 (m, 1H). ^{13}C{^{1}H} (126 MHz, $CDCl_3$) δ: 179.1, 119.4, 98.5 (dd, J = 178, 4 Hz), 88.8 (d, J = 27 Hz), 66.4, 66.3 (rotamer), 66.3 (rotamer), 49.2 (rotamer), 49.1 (rotamer), 48.5, 31.3 (d, J = 2 Hz), 23.8-23.6 (rotamer d, J = 23 Hz), 22.8 (d, J = 24 Hz), 20.3, 17.4. ^{11}B NMR (128 MHz,$CDCl_3$) δ: 5.08. ^{19}F NMR (376 MHz, $CDCl_3$) δ: -142.11 - -142.35 (m), -145.17 (d, J = 70 Hz, ^{10}B-F), -145.23 (d, J = 70 Hz, ^{11}B-F), -147.06 (d, J = 70 Hz, ^{10}B-F), -147.14 (d, J = 70 Hz, ^{11}B-F). HRMS (DART) m/z calc'd for $C_{13}H_{24}BN_3O_3F_3$ $[M+NH_4]^+$: 338.18573; found: 338.18564. Melting point:135 – 136 °C.

1-(3,3-Dimethyl-2-(morpholine-4-carbonyl)oxiran-2-yl)octan-4-one (8): To an oven-dried 2-dram vial equipped with a stir bar was added **3g** (44.1 mg, 0.175 mmol, 1 equiv.), then the vial was capped with a B24 septum and the vessel was purged of air with nitrogen. THF (1.7 mL) was added, then the solution was cooled with a dry ice/acetone bath for 20 minutes before the addition of *n*BuLi (74 µL, 0.184 mmol, 1.05 equiv.). The mixture was stirred overnight while warming to room temperature then loaded onto Celite. The volatiles were removed *in vacuo*, then the residue was subjected to reversed-phase column chromatography to afford the product as a clear oil (7.4 mg, 14%, 26.6 mg of **3g** recovered). ^{1}H NMR (500 MHz, $CDCl_3$, multiple conformers reported as one species) δ: 3.92-3.29 (m, 8H), 2.62-2.34 (m, 4H), 2.03-1.89 (m, H), 1.82-1.75 (m, 1H), 1.71-1.63 (m, 1H), 1.58-1.50 (m, 3H), 1.35-1.34 (overlapping s, 3H), 1.31-1.26 (m, 5H), 0.91-0.87 (m, 3H). ^{13}C{^{1}H} (126 MHz, $CDCl_3$, exists as two rotamers) δ: 211.3 (minor), 210.5, 168.6, 168.5 (minor), 68.5, 67.2, 67.1, 67.1 (minor), 67.1 (minor), 66.8 (minor), 63.0, 61.7 (minor), 46.1, 45.9 (minor), 42.7 (minor), 42.7 (minor), 42.4, 42.4, 41.9 (minor), 41.4 (minor), 31.1, 29.6 (minor), 26.1, 23.1, 22.5 (minor), 22.5, 22.1 (minor), 19.8, 19.8, 19.6 (minor), 19.4, 14.0. HRMS (DART) m/z calc'd for $C_{17}H_{30}NO_4$ $[M+H]^+$: 312.21693; found: 312.21631.

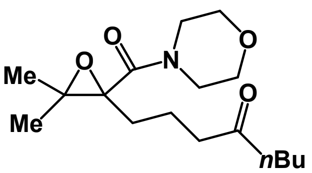

***rac*-4-((2*S*,3*R*)-3-(4-Methoxybenzyl)-3-methyl-2-(pyrrolidine-1-carbonyl)oxiran-2-yl)butanenitrile (*trans*-3r):** The product was synthesized using general procedure D from ***cis*-1r** (54.1 mg, 0.15 mmol, 1 equiv.) with KO*t*Bu (2.4 equiv.) and TsCl (1.4 equiv.) in THF/DMF (1.5 mL), and further purified by normal phase column chromatography to be obtained as a clear oil (32.5 mg, 63% yield, dr 20:1). ^{1}H NMR (500 MHz, $CDCl_3$, two conformers reported as one species) δ: 7.19 (m, 2H), 6.86 (m, 2H), 3.79 (s, 3H), 3.74-3.72 (m, 1H), 3.56-3.45 (m, 3H), 3.32-3.27 (m, minor), 3.15-3.09 (m, minor), 2.92-2.83 (q, J = 15 Hz, 2H), 2.62-2.59 (m, minor), 2.44-2.38 (m overlap with minor, 2H), 2.30-2.28 (m, minor), 2.00-1.72 (m, 8H), 1.14 (s, 3H). ^{13}C{^{1}H} (126 MHz, $CDCl_3$, exists as two rotamers) δ: 168.0, 167.8

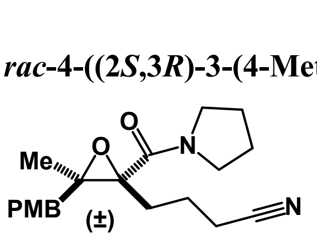

(minor), 158.7 (minor), 158.5, 130.7 (minor), 130.3, 128.6, 128.4 (minor), 119.7 (minor), 119.2, 114.2, 68.8, 67.9 (minor), 65.1, 65.0 (minor), 55.4, 46.9, 46.4, 45.7 (minor), 38.9 (minor), 38.6, 30.6, 28.0 (minor), 26.6, 26.5 (minor), 23.9 (minor), 23.5, 22.0, 21.9 (minor), 19.6, 18.6 (minor), 17.4, 17.0 (minor). HRMS (DART) m/z calc'd for $C_{20}H_{27}N_2O_3$ $[M+H]^+$: 343.20162; found: 343.20213.

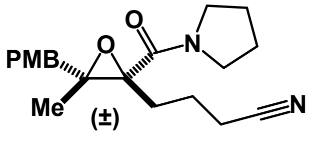

***rac*-4-((2*S*,3*S*)-3-(4-Methoxybenzyl)-3-methyl-2-(pyrrolidine-1-carbonyl)oxiran-2-yl)butanenitrile (*cis*-3r):** The product was synthesized using general procedure D from ***trans*-1r** (15.1 mg, 0.042 mmol, 1 equiv.) with KO*t*Bu (2.4 equiv.) and TsCl (1.4 equiv.) in THF/DMF (0.4 mL) and obtained as a clear oil (8.2 mg, 57% yield, dr > 20:1). ^{1}H NMR (500 MHz, $CDCl_3$, two conformers reported as one species) δ: 7.20 (d, J = 8.5 Hz, 2H), 6.83 (d, J = 8.5 Hz, 2H), 3.79-3.75 (m overlap with s, 4H), 3.61-3.43 (m, 3H), 2.81 (d, J = 14.8 Hz, 1H), 2.56 (d, J = 14.8 Hz, 1H), 2.46-2.41 (m, 2H), 2.22-2.17 (m, 1H), 1.99-1.71 (m, 8H), 1.25-1.21 (overlapping s, 3H). ^{13}C{^{1}H} (126 MHz, $CDCl_3$) δ: 167.9, 158.5, 130.9, 129.2, 119.3, 113.9, 68.4, 65.5, 55.4, 46.9, 46.4, 41.3, 30.7, 26.6, 23.5, 21.8, 17.4, 17.2. HRMS (DART) m/z calc'd for $C_{20}H_{27}N_2O_3$ $[M+H]^+$: 343.20162; found: 343.20182.

***rac*-4-((2*S*,3*R*)-3-Allyl-3-methyl-2-(pyrrolidine-1-carbonyl)oxiran-2-yl)butanenitrile**

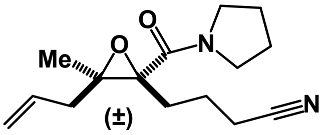

(*trans*-3s): The product was synthesized using general procedure D from ***cis*-1s** (42.1 mg, 0.15 mmol, 1 equiv.) with KO*t*Bu (2.4 equiv.) and TsCl (1.4 equiv.) in THF/DMF (1.5 mL) and obtained as a clear oil (27.8 mg, 71% yield, > dr 20:1). ^{1}H NMR (500 MHz, $CDCl_3$) δ: 5.83-5.75 (m, 1H), 5.22-5.14 (m, 2H), 3.71-3.66 (m, 1H), 3.53-3.35 (m, 3H), 2.56-2.17 (m, 5H), 1.95-1.67 (m, 7H), 1.21 (s, 3H). ^{13}C{^{1}H} (126 MHz, $CDCl_3$, exists as two rotamers) δ: 167.8, 167.8 (minor), 132.5, 132.1 (minor), 119.6 (minor), 119.4 (minor), 119.2, 118.6, 68.7, 67.6 (minor), 64.3, 63.7 (minor), 46.8, 46.5 (minor), 46.3, 45.7 (minor), 38.8 (minor), 38.2, 30.1, 27.5 (minor), 26.5, 26.5 (minor), 23.8 (minor), 23.5, 21.8, 21.7 (minor), 19.8, 18.7 (minor), 17.3, 16.9 (minor). HRMS (DART) m/z calc'd for $C_{15}H_{23}N_2O_2$ $[M+H]^+$: 263.17540; found: 263.17611.

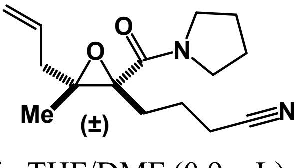

***rac*-4-((2*S*,3*S*)-3-Allyl-3-methyl-2-(pyrrolidine-1-carbonyl)oxiran-2-yl)butanenitrile (*cis*-3s):** The product was synthesized using general procedure D from ***trans*-1s** (25.0 mg, 0.089 mmol, 1 equiv.) with KO*t*Bu (2.4 equiv.) and TsCl (1.4 equiv.) in THF/DMF (0.9 mL) and obtained as a clear oil (16.9 mg, 72% yield, dr 20:1). ^{1}H NMR (500 MHz, $CDCl_3$, two conformers reported as one species) δ: 5.90-5.77 (m, 1H), 5.12-5.06 (m, 2H), 3.74-3.69 (m, 1H), 3.53-3.36 (m, 3H), 2.53-2.41 (m, 2H), 2.24-2.09 (m, 3H), 1.95-1.69

(m, 7H), 1.36-1.32 (overlapping s, 3H). $^{13}C\{^1H\}$ (126 MHz, $CDCl_3$) δ: 167.7, 133.1, 119.3, 118.7, 68.5, 64.4, 46.7, 46.4, 41.2, 30.5, 26.6, 23.5, 21.8, 17.3, 17.3. HRMS (DART) m/z calc'd for $C_{15}H_{23}N_2O_2$ $[M+H]^+$: 263.27540; found: 263.17569.

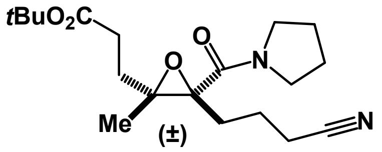

***rac-tert*-Butyl 3-((2*S*,3*S*)-3-(3-cyanopropyl)-2-methyl-3-(pyrrolidine-1-carbonyl)oxiran-2-yl)propanoate (*cis*-3t):** The product was synthesized using general procedure D from ***trans*-1t** (27.6 mg, 0.075 mmol, 1 equiv.) with KO*t*Bu (2.4 equiv.) and TsCl (1.4 equiv.) in THF (0.75 mL) and obtained as a clear oil (6.1 mg, 23% yield, dr > 20:1). ^1H NMR (500 MHz, $CDCl_3$) δ: 3.75-3.72 (m, 1H), 3.55-3.35 (m, 3H), 2.50-2.38 (m, 3H), 2.32-2.26 (m, 1H), 2.17-2.12 (m, 1H), 1.95-1.69 (m, 9H), 1.42 (s, 9H), 1.34 (s, 3H). $^{13}C\{^1H\}$ (126 MHz, $CDCl_3$) δ: 172.3, 167.5, 119.3, 80.5, 68.8, 64.3, 46.7, 46.4, 31.7, 31.1, 30.5, 28.2, 26.6, 23.5, 21.8, 17.3, 17.3. HRMS (DART) m/z calc'd for $C_{19}H_{31}N_2O_4$ $[M+H]^+$: 351.22783; found: 351.22761.

2D NOESY NMR analysis of *trans*- and *cis*-3r

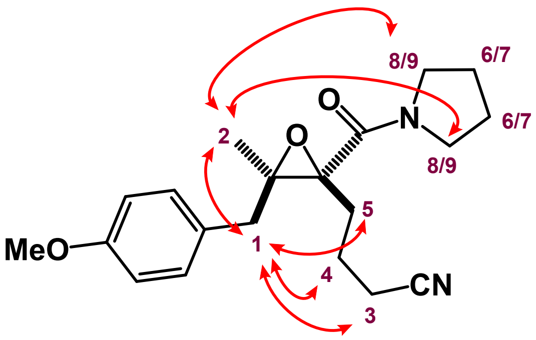

***trans*-3r**
(from *cis*-1r)

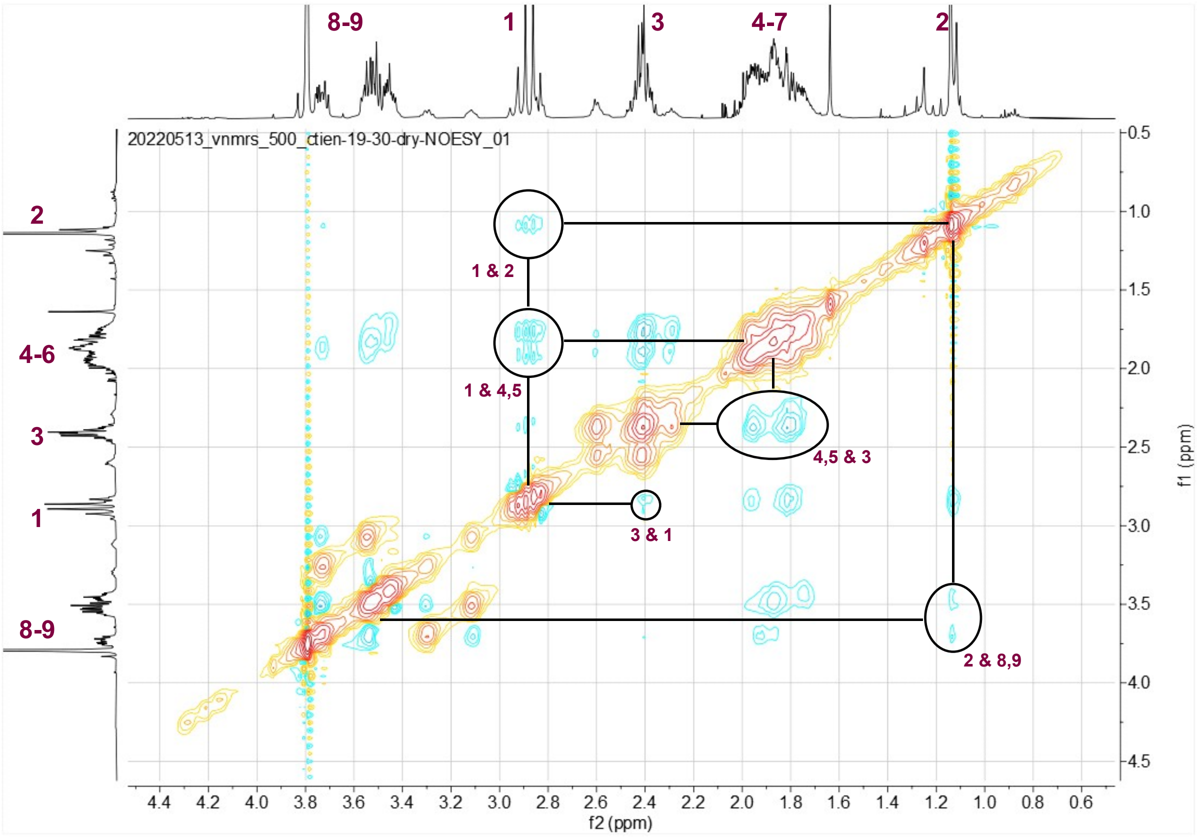

Figure S1. NOESY spectrum of ***trans*-3r** for diastereomer assignment.

Peaks were assigned based on ^{1}H, ^{13}C, COSY, HSQC and HMBC NMR spectroscopy.

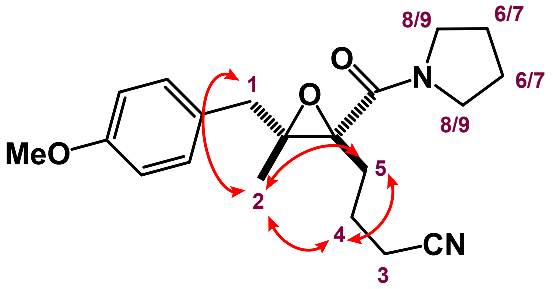

***cis*-3r**
(from *trans*-1r)

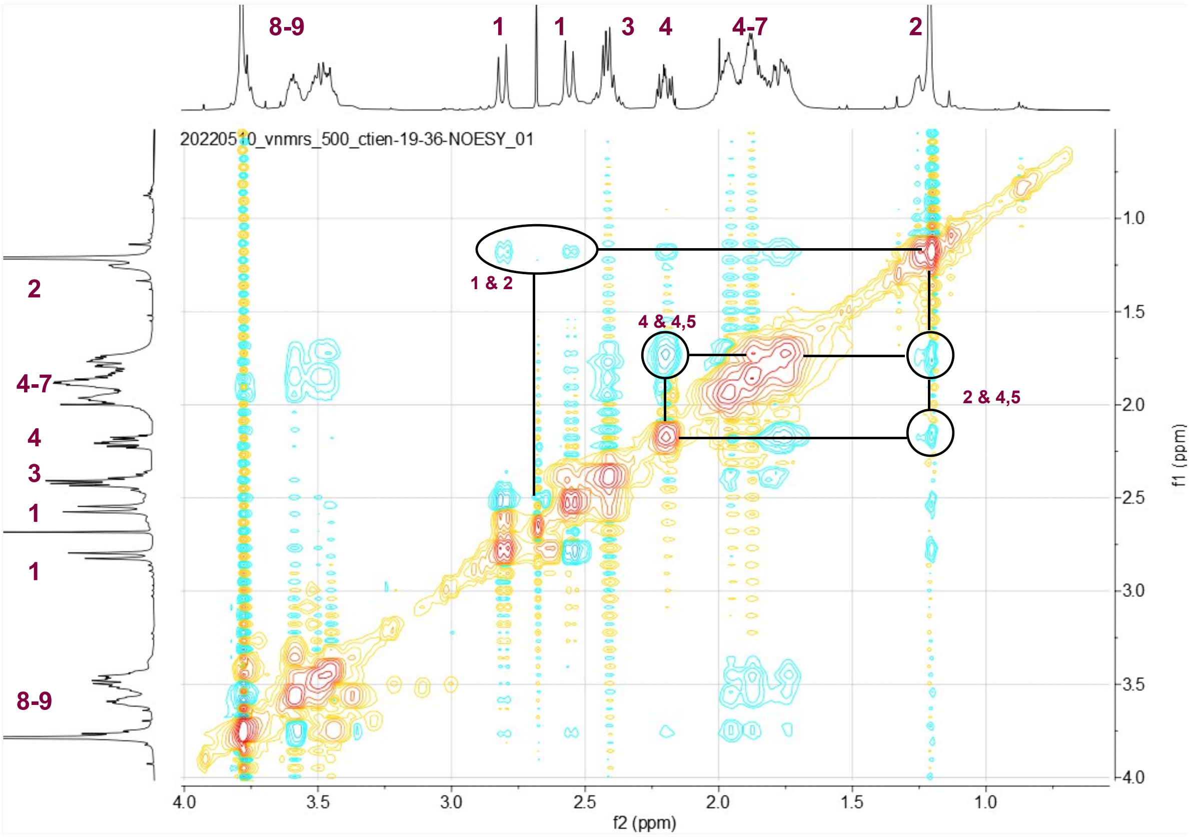

Figure S2. NOESY spectrum of ***cis*-3r** for diastereomer assignment.

Peaks were assigned based on ^{1}H, ^{13}C, COSY, HSQC and HMBC NMR spectroscopy.

<u>LCMS and TLC traces of *trans*- and *cis*-3r</u>

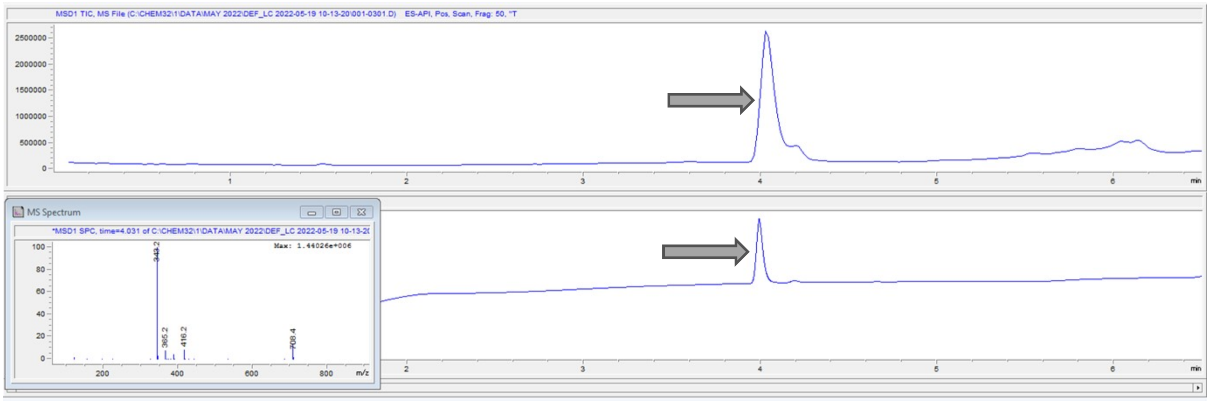

Figure S3. LCMS trace of ***trans*-3r** with the MS spectrum of the product (4.03 min) shown.

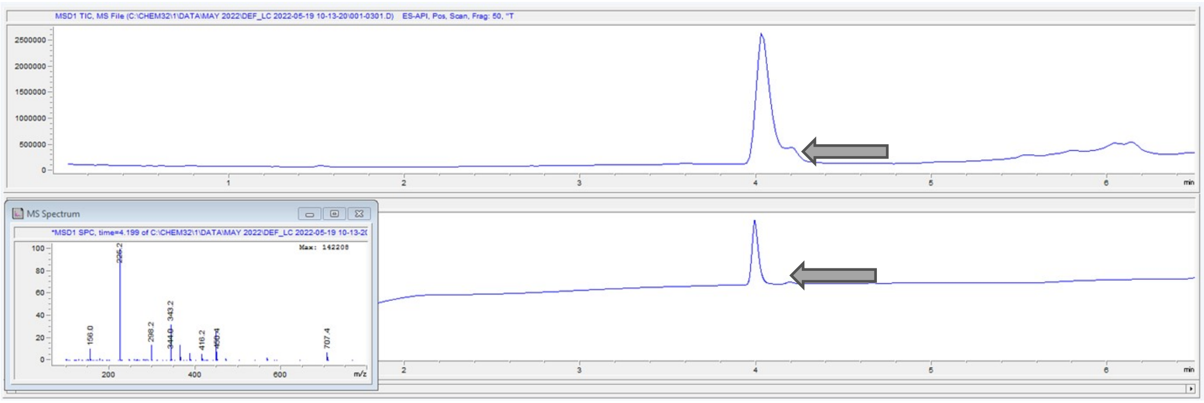

Figure S4. LCMS trace of ***trans*-3r** with the MS spectrum of the column impurity (4.2 min) shown.

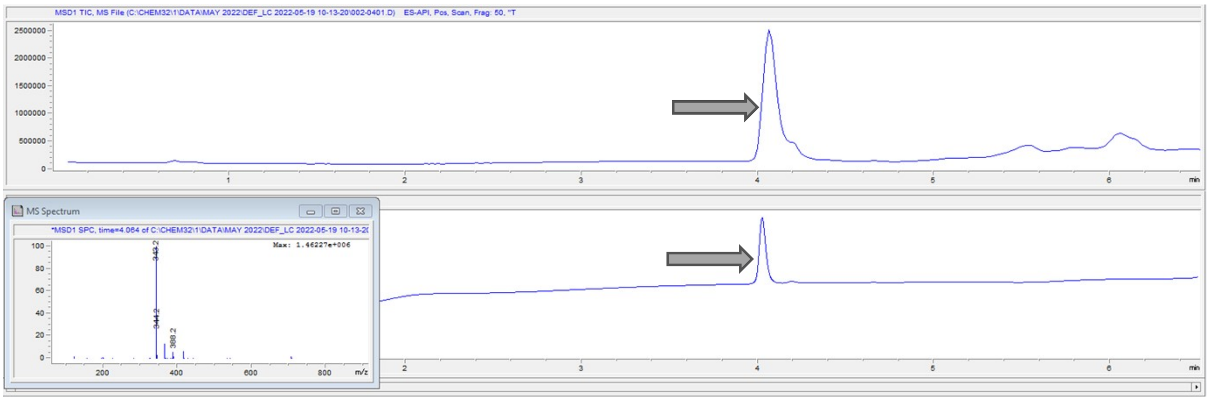

Figure S5. LCMS trace of ***cis*-3r** with the MS spectrum of the product (4.06 min) shown.

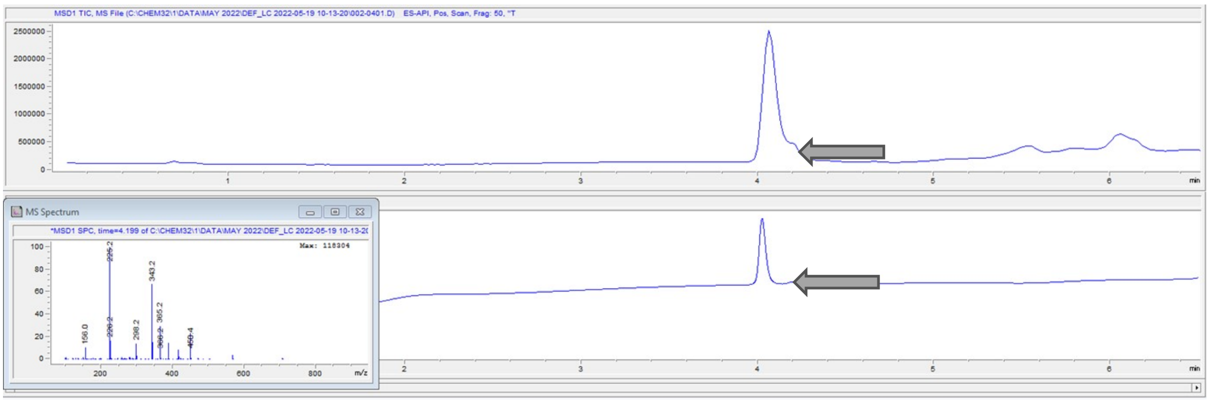

Figure S6. LCMS trace of ***cis*-3r** with the MS spectrum of the column impurity (4.2 min) shown.

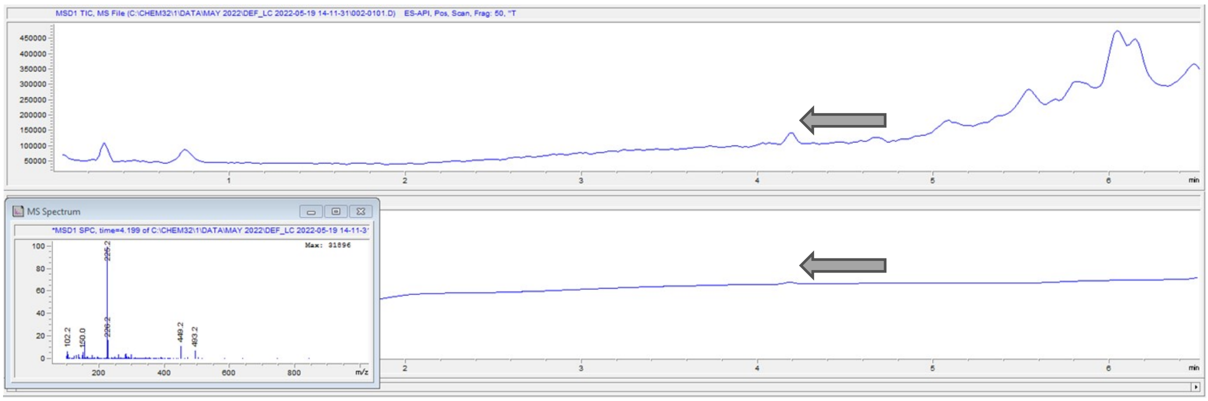

Figure S7. LCMS trace of a blank run with the MS spectrum of the column impurity (4.2 min) shown.

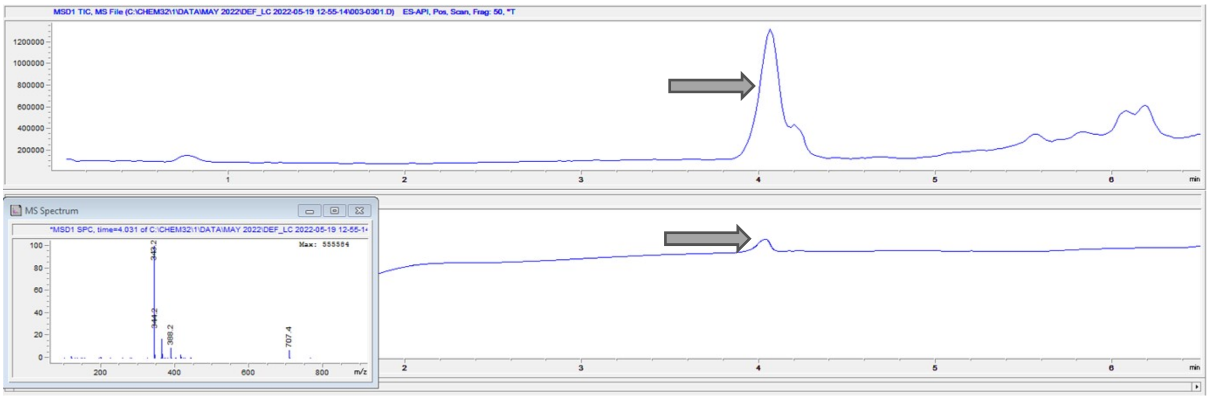

Figure S8. LCMS trace of a mixture of ***trans*-3r** and ***cis*-3r** with the MS spectrum of the two products (4.03 min) shown.

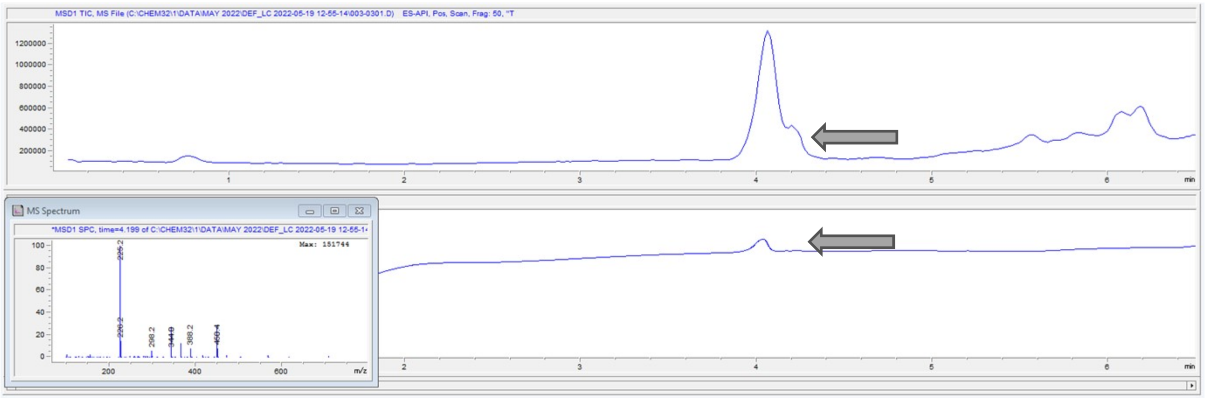

Figure S9. LCMS trace of a mixture of ***trans*-3r** and ***cis*-3r** with the MS spectrum of the column impurity (4.2 min) shown.

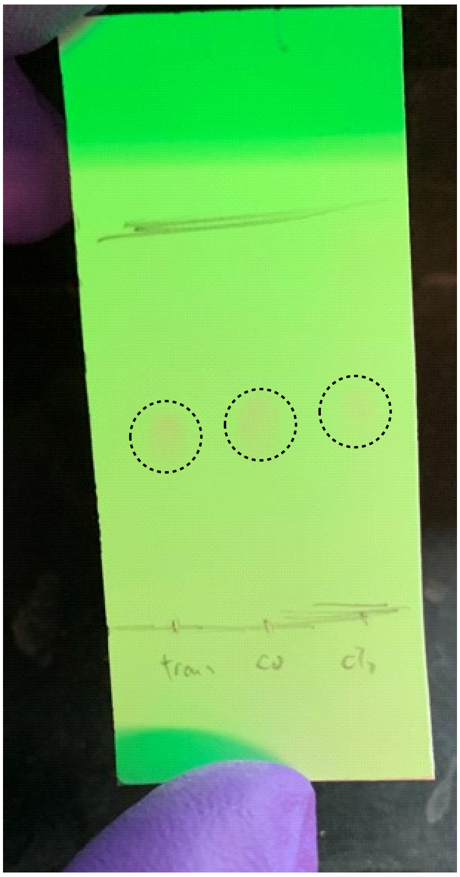

Figure S10. TLC trace of ***trans*-3r** (left), ***cis*-3r** (right) and a co-spot (middle) eluted with 100% EtOAc visualized under UV light at 254 nm.

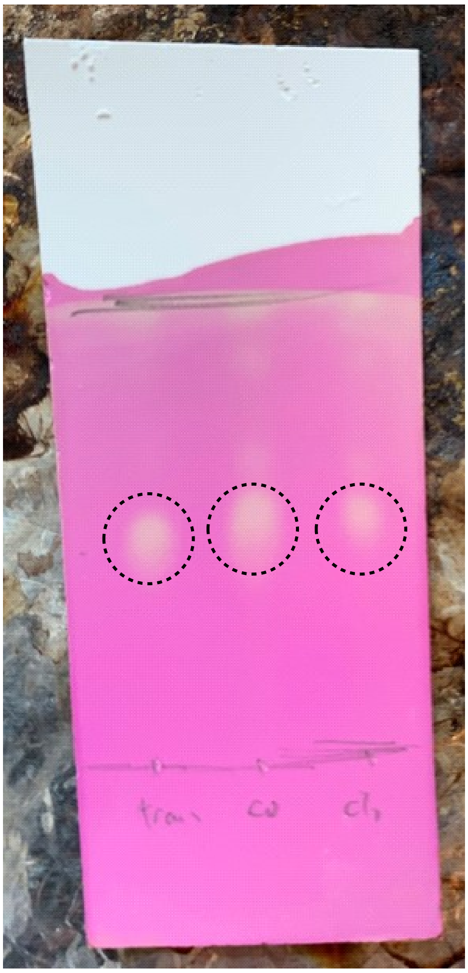

Figure S11. TLC trace of ***trans*-3r** (left), ***cis*-3r** (right) and a co-spot (middle) eluted with 100% EtOAc visualized with $KMnO_4$ stain.

The RP-LCMS and TLC traces are included to demonstrate that ***trans*-3r** and ***cis*-3r** are inseparable by both reversed-phase and normal phase column chromatography. Therefore, purification by column chromatography does not affect the *dr* of the reaction.

NMR spectra

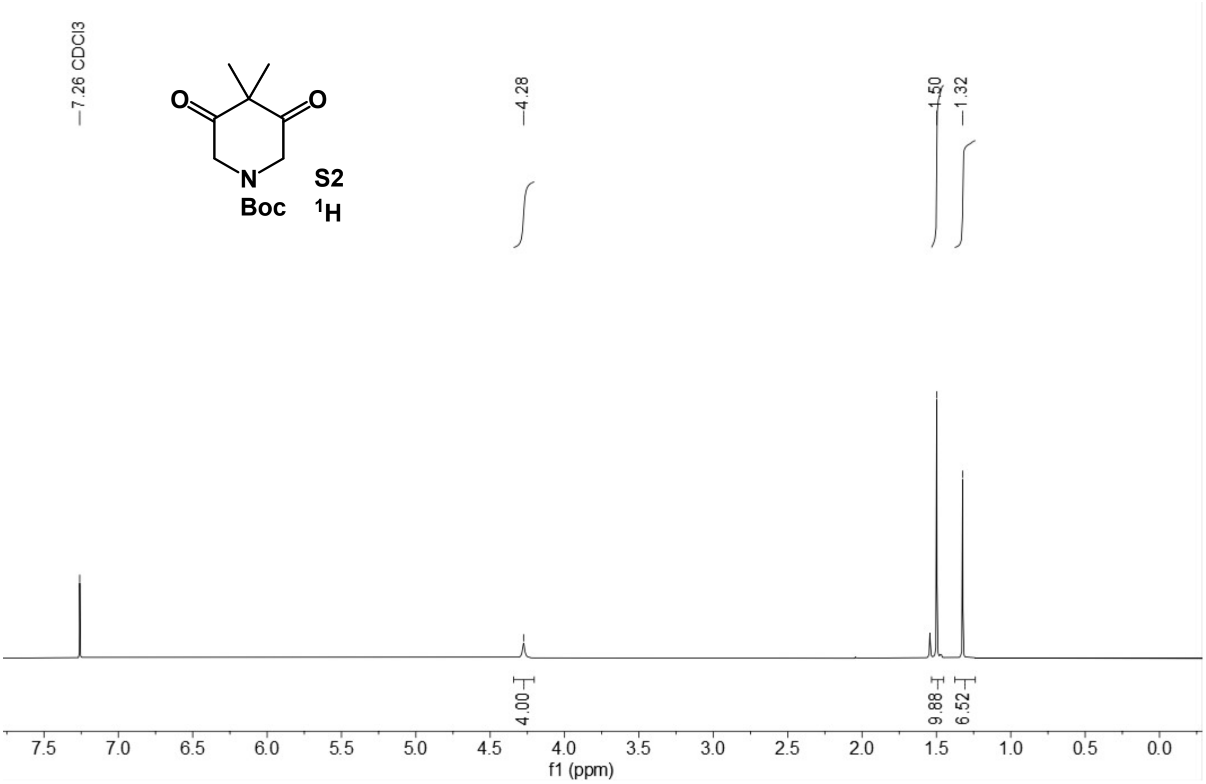
7.26 CDCl3
4.28
1.50
1.32
O
O
N
Boc
S2
1H
4.00
9.88
6.52
7.5
7.0
6.5
6.0
5.5
5.0
4.5
4.0
3.5
3.0
2.5
2.0
1.5
1.0
0.5
0.0
f1 (ppm)

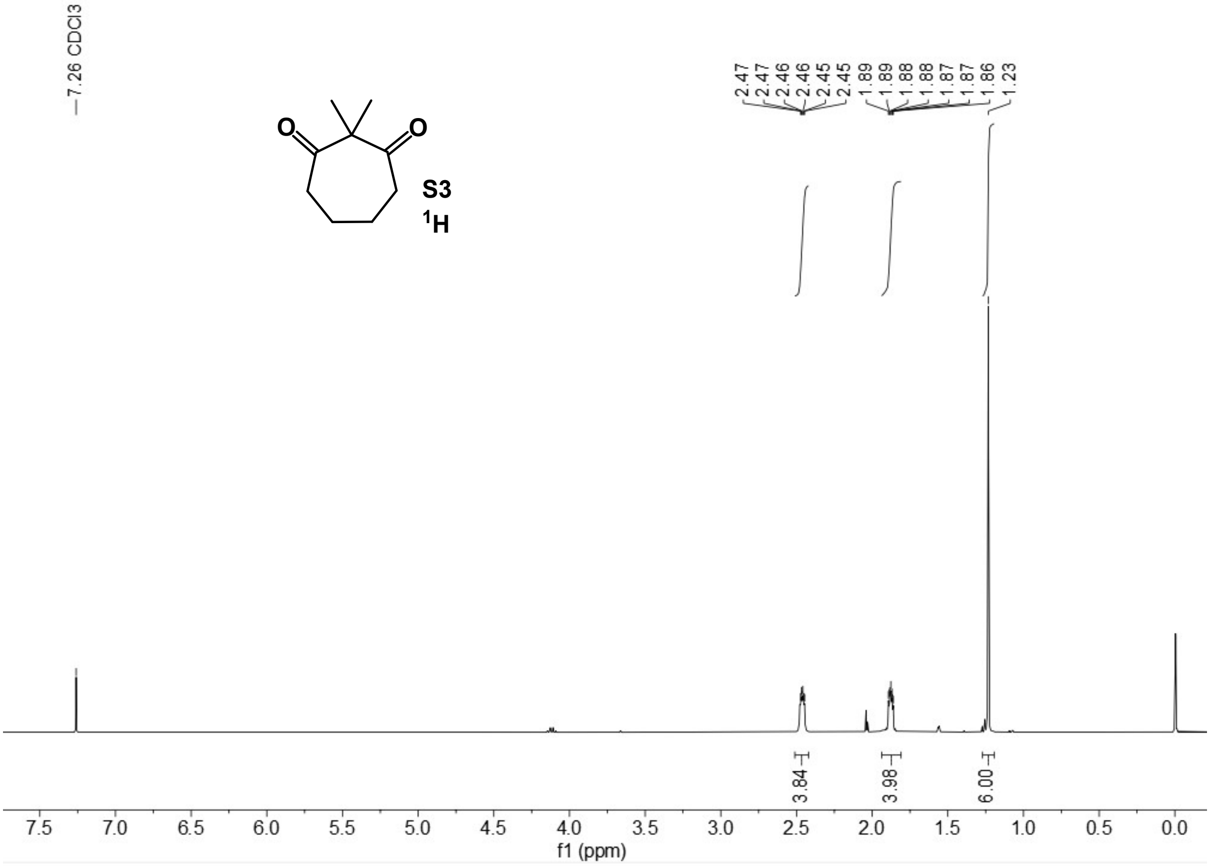
7.26 CDCl3
O
O
S3
1H
2.47
2.47
2.46
2.46
2.45
2.45
1.89
1.89
1.88
1.88
1.87
1.87
1.86
1.23
3.84
3.98
6.00
7.5 7.0 6.5 6.0 5.5 5.0 4.5 4.0 3.5 3.0 2.5 2.0 1.5 1.0 0.5 0.0
f1 (ppm)

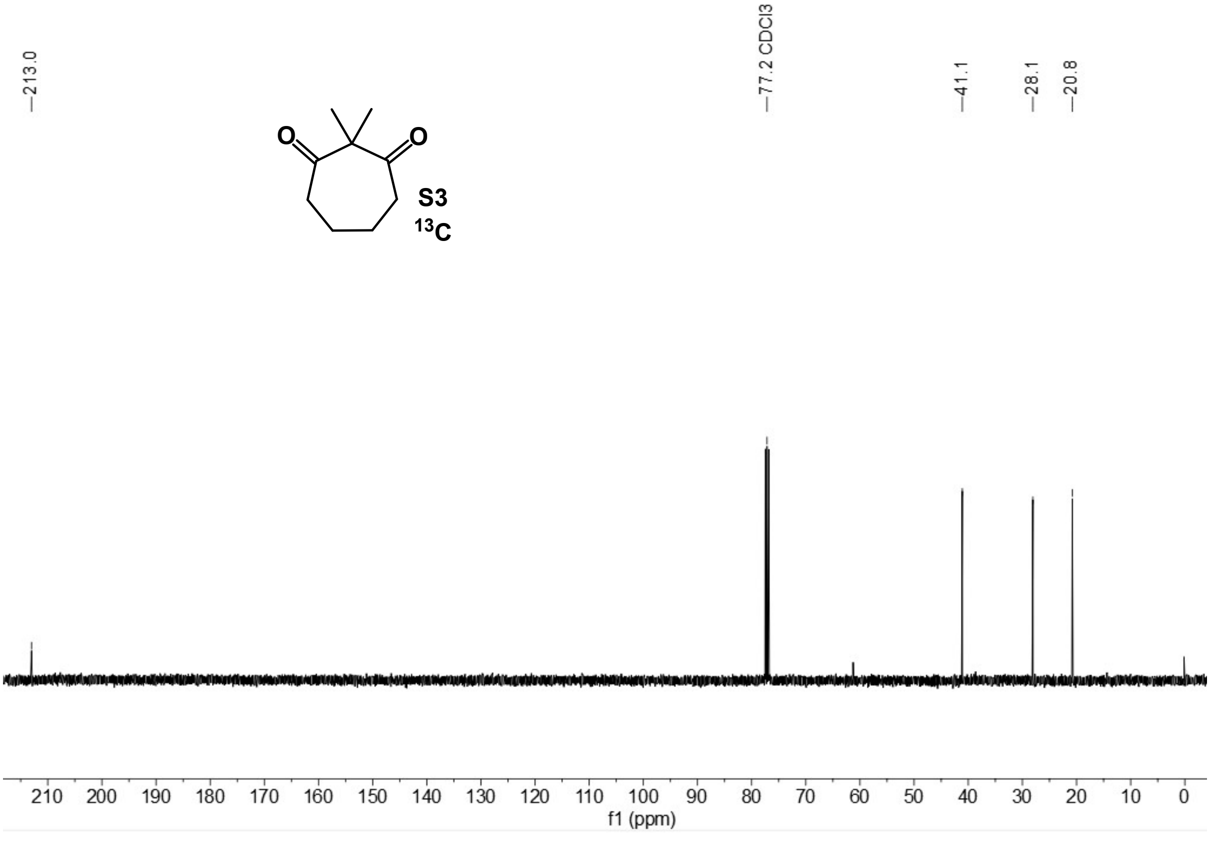
213.0
77.2 CDCl3
41.1
28.1
20.8
O
O
S3
13C
210 200 190 180 170 160 150 140 130 120 110 100 90 80 70 60 50 40 30 20 10 0
f1 (ppm)

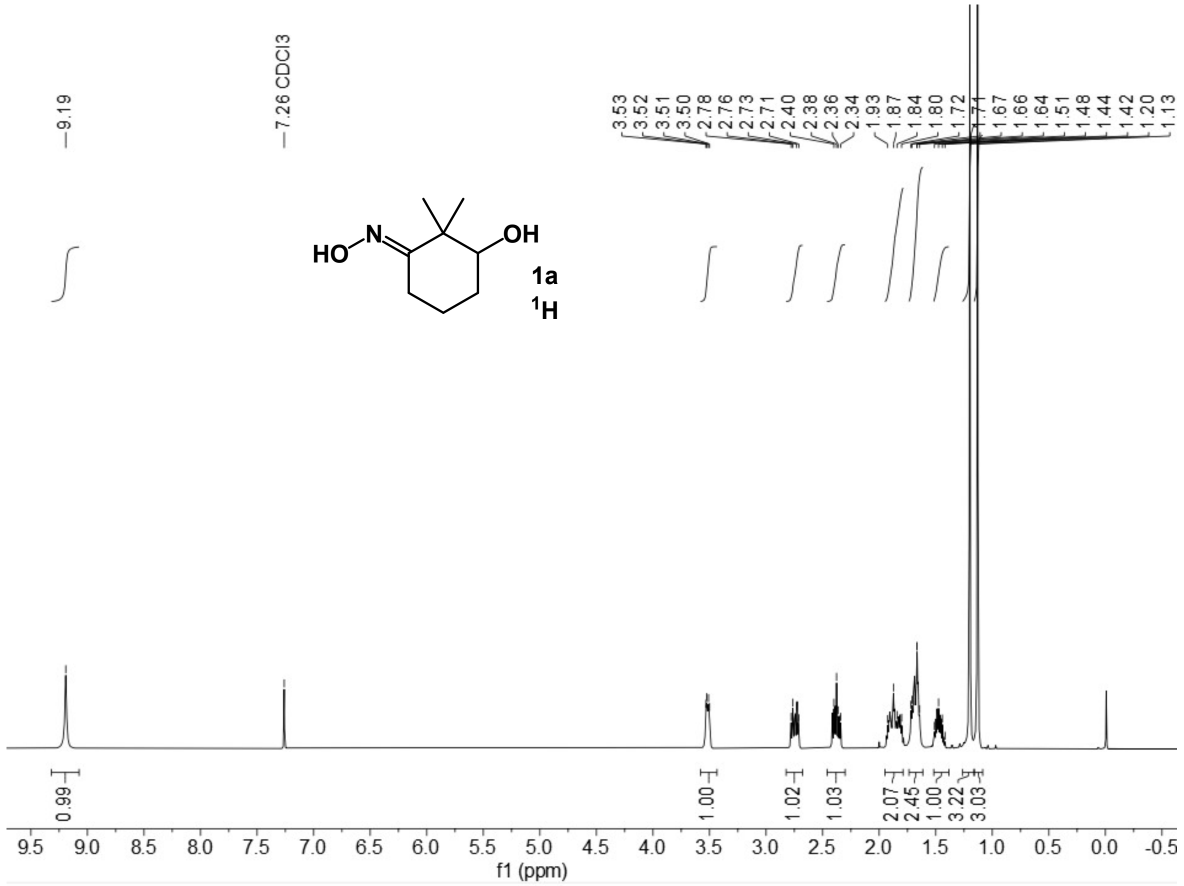
9.19
7.26 CDCl3
3.53 3.52 3.51 3.50 2.78 2.76 2.73 2.71 2.40 2.38 2.36 2.34 1.93 1.87 1.84 1.80 1.72 1.71 1.67 1.66 1.64 1.51 1.48 1.44 1.42 1.20 1.13
HO
N
OH
1a
1H
0.99
1.00
1.02
1.03
2.07
2.45
1.00
3.22
3.03
9.5 9.0 8.5 8.0 7.5 7.0 6.5 6.0 5.5 5.0 4.5 4.0 3.5 3.0 2.5 2.0 1.5 1.0 0.5 0.0 -0.5
f1 (ppm)

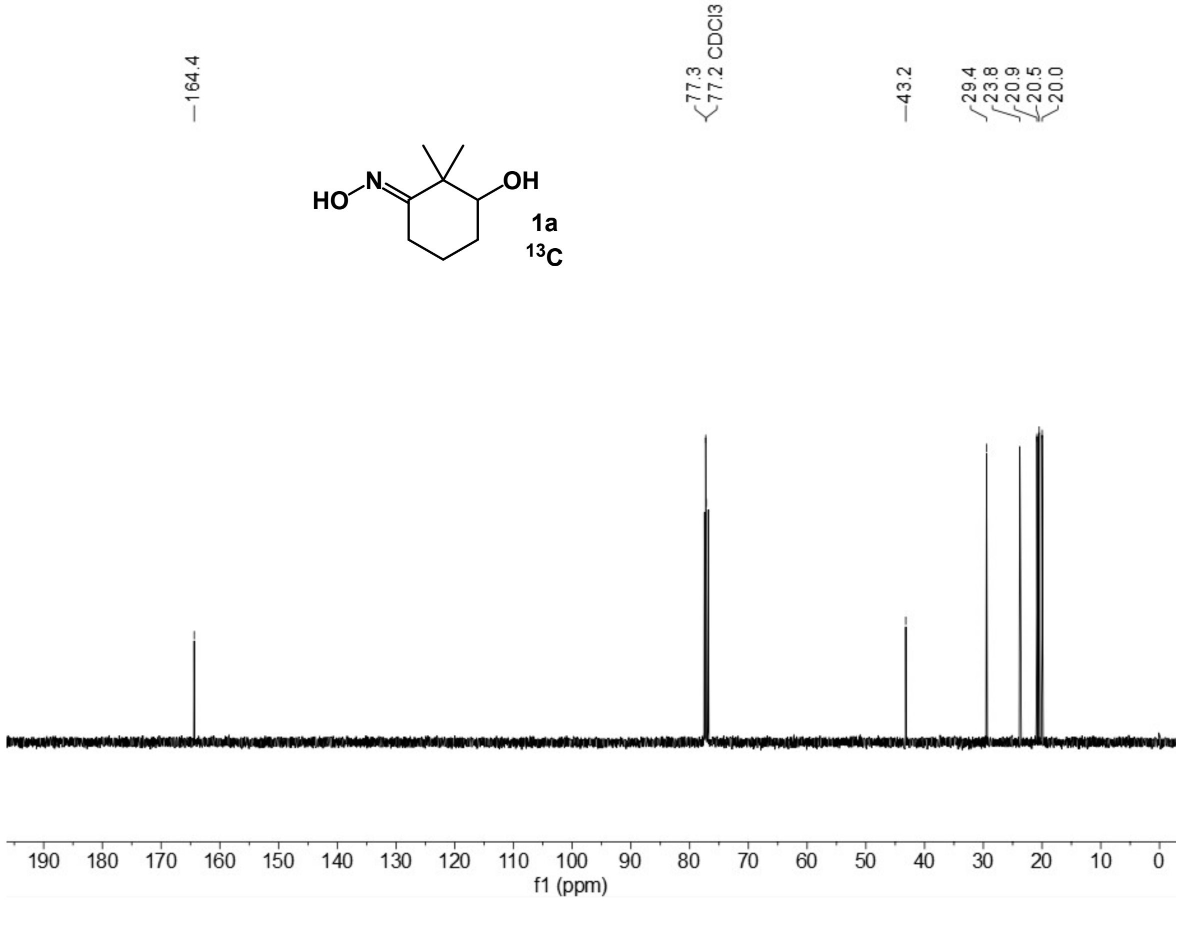
164.4
77.3
77.2 CDCl3
43.2
29.4 23.8 20.9 20.5 20.0
HO
N
OH
1a
13C
190 180 170 160 150 140 130 120 110 100 90 80 70 60 50 40 30 20 10 0
f1 (ppm)

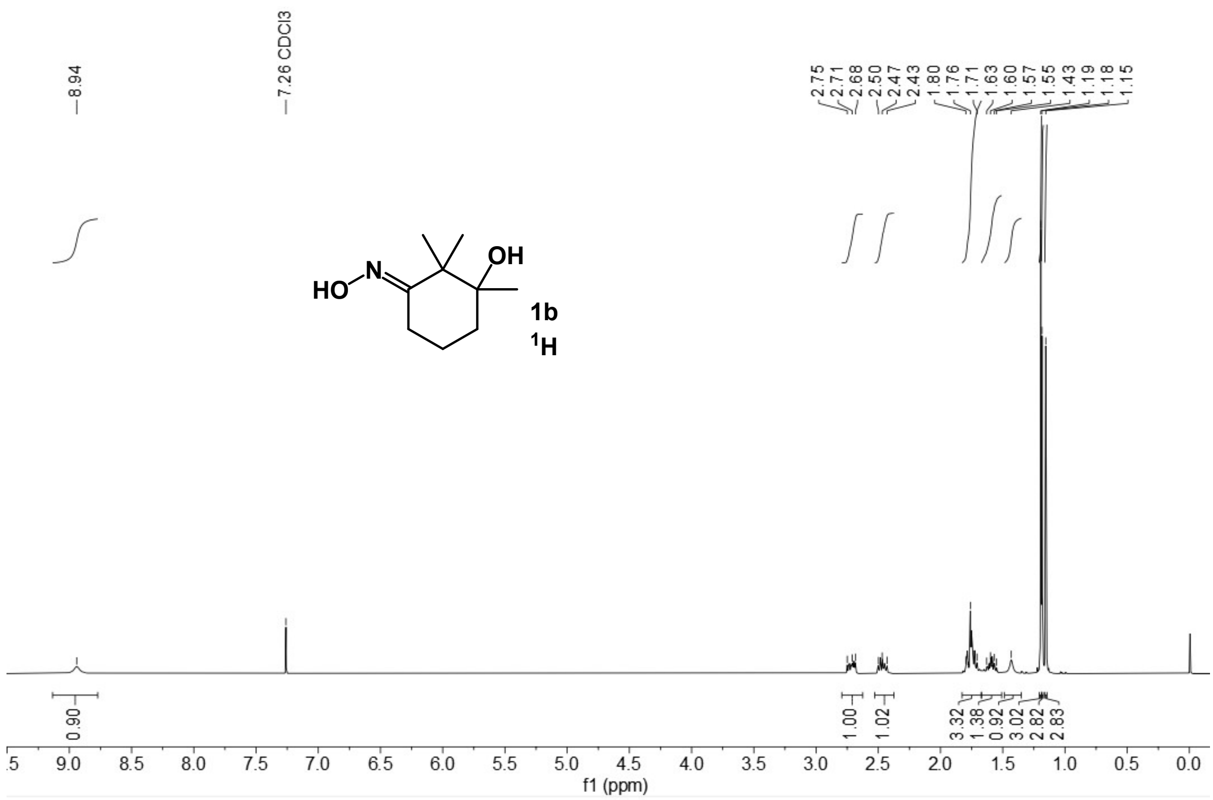
8.94
7.26 CDCl3
2.75
2.71
2.68
2.50
2.47
2.43
1.80
1.76
1.71
1.63
1.60
1.57
1.55
1.43
1.19
1.18
1.15
HO
N
OH
1b
1H
0.90
1.00
1.02
3.32
1.38
0.92
3.02
2.82
2.83
f1 (ppm)

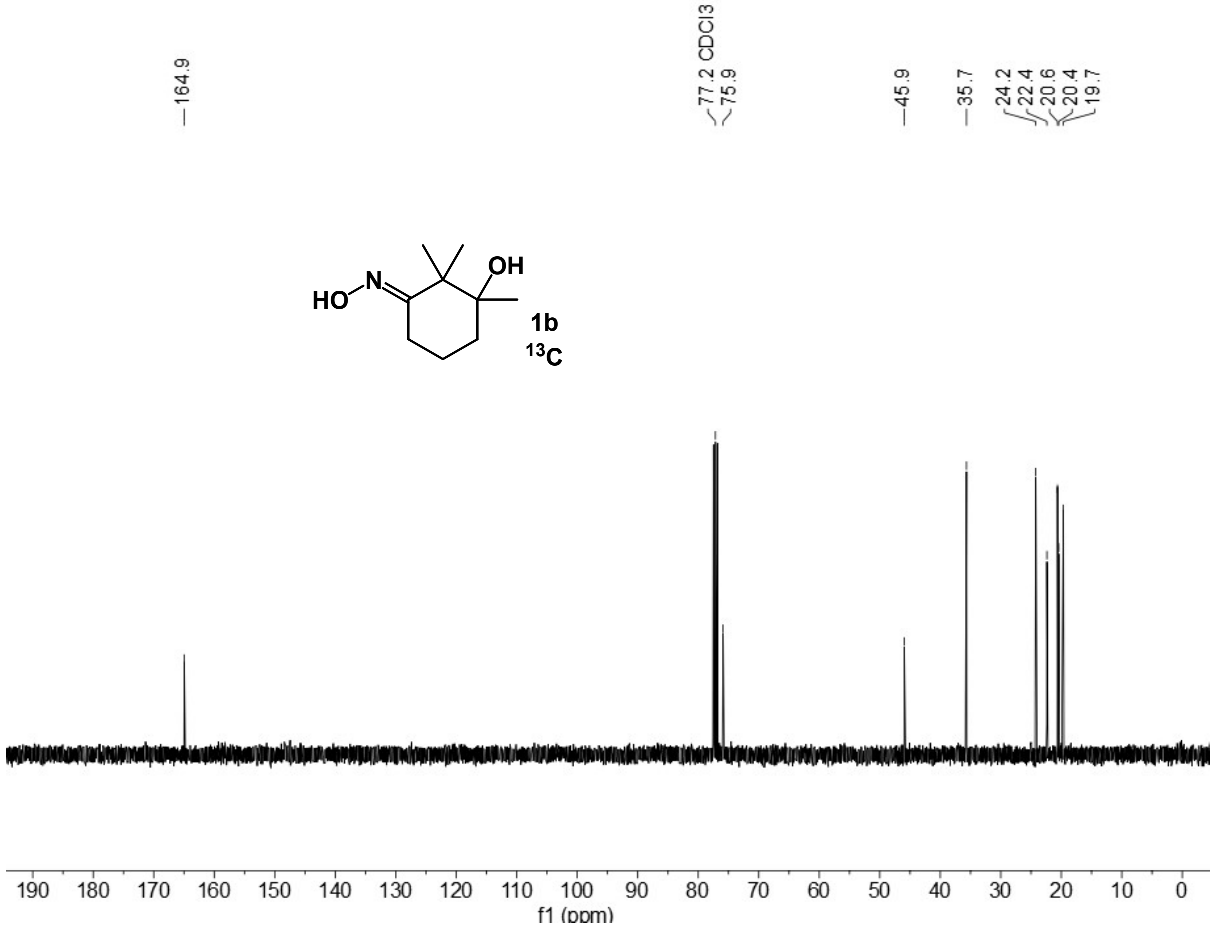
164.9
77.2 CDCl3
75.9
45.9
35.7
24.2
22.4
20.6
20.4
19.7
HO
N
OH
1b
13C
f1 (ppm)

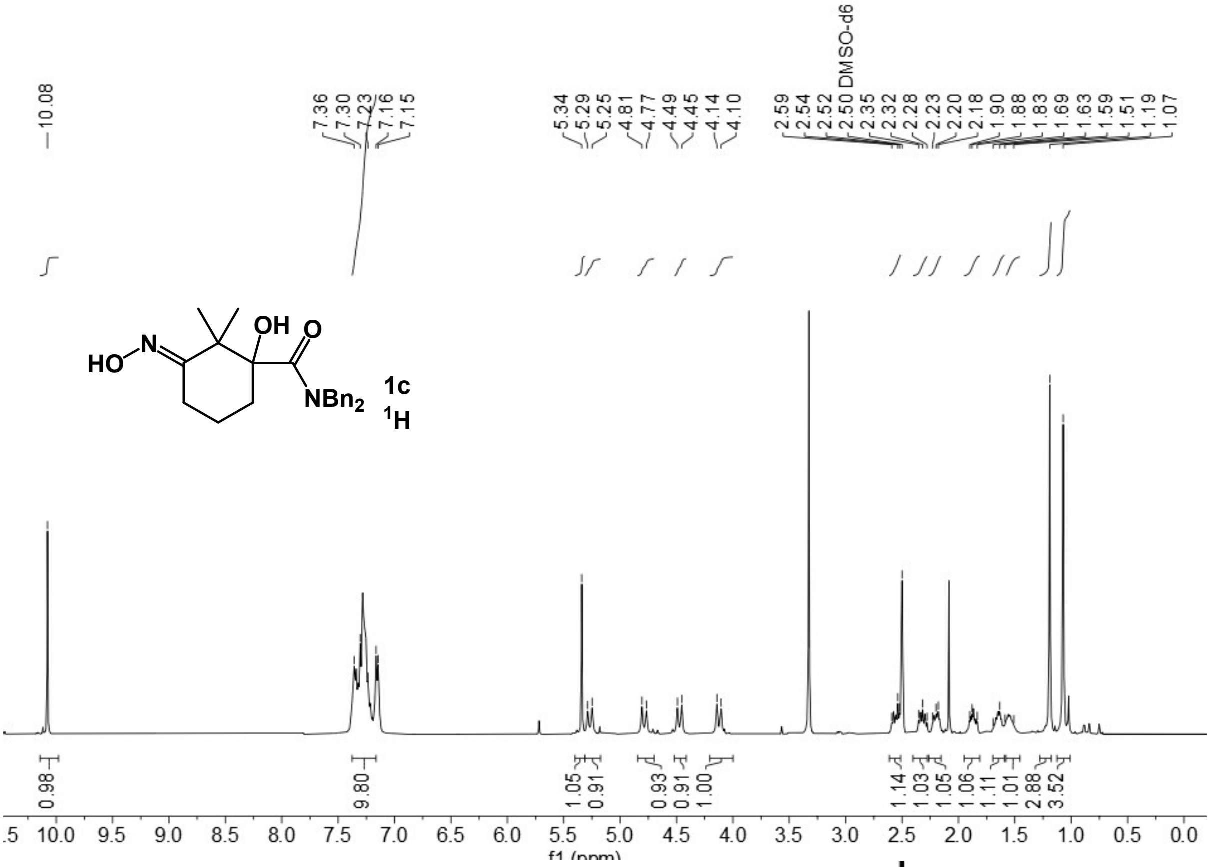

10.08
7.36 7.30 7.23 7.16 7.15
5.34 5.29 5.25 4.81 4.77 4.49 4.45 4.14 4.10
2.59 2.54 2.52 2.50 DMSO-d6 2.35 2.32 2.28 2.23 2.20 2.18 1.90 1.88 1.83 1.69 1.63 1.59 1.51 1.19 1.07
HO
N
OH
O
NBn2
1c
1H
0.98 9.80 1.05 0.91 0.93 0.91 1.00 1.14 1.03 1.05 1.06 1.11 1.01 2.88 3.52
f1 (ppm)

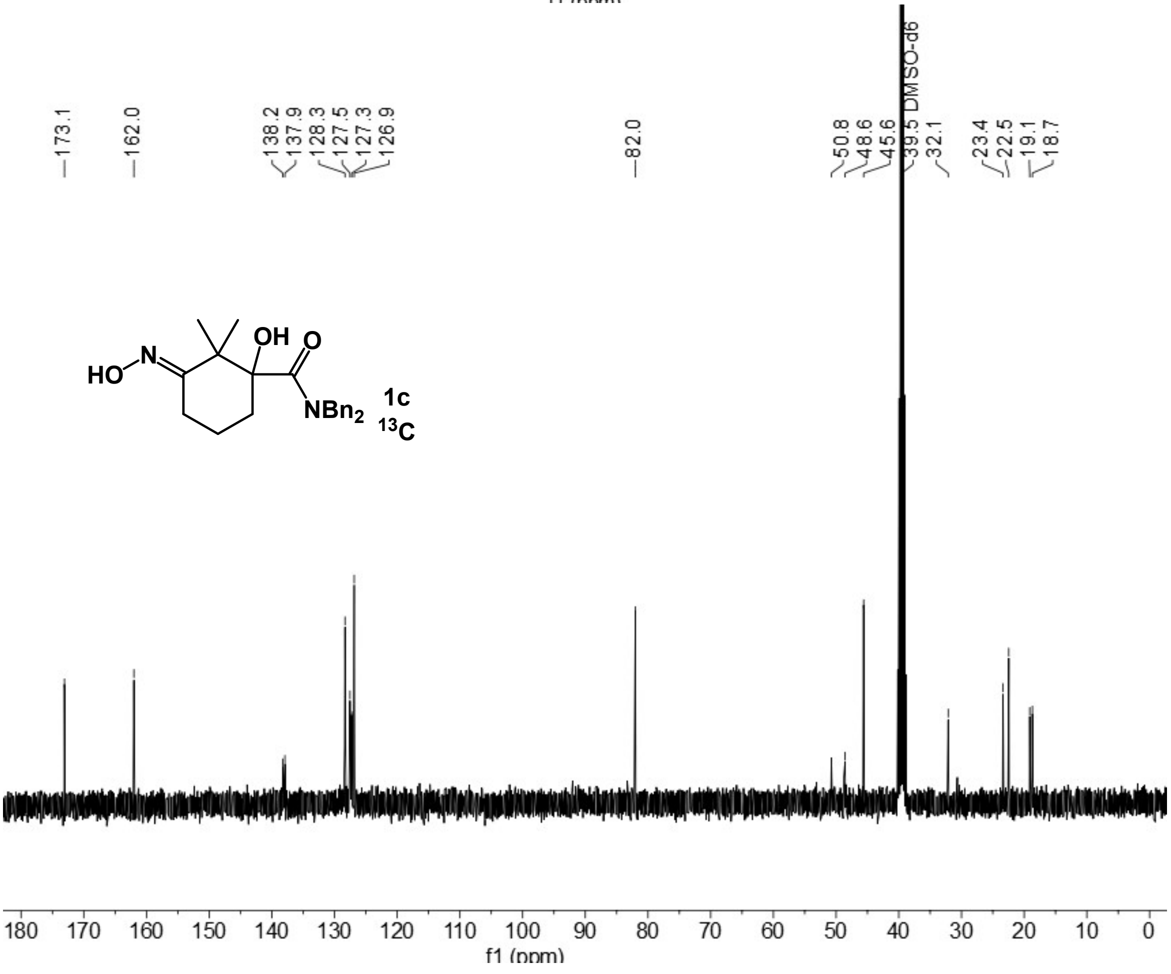

173.1
162.0
138.2 137.9 128.3 127.5 127.3 126.9
82.0
50.8 48.6 45.6 39.5 DMSO-d6 32.1
23.4 22.5 19.1 18.7
HO
N
OH
O
NBn2
1c
13C
f1 (ppm)

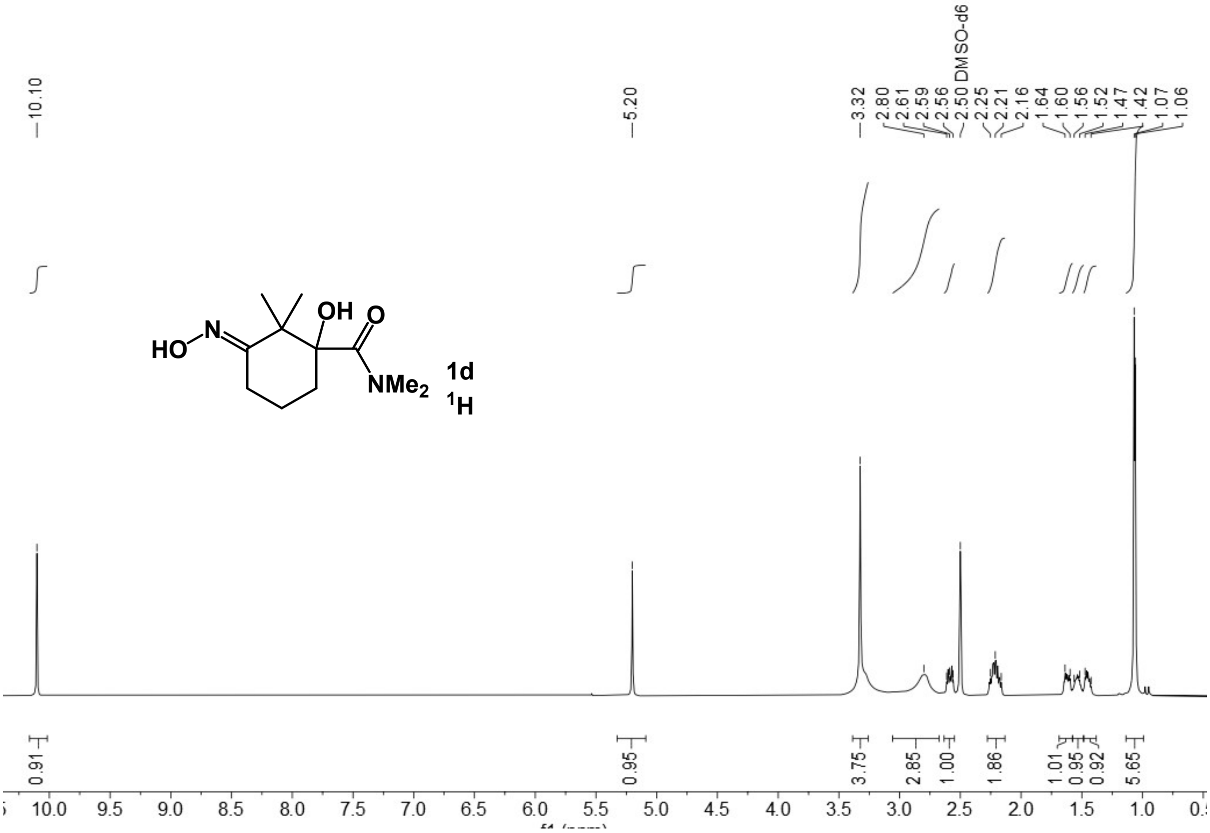
10.10
5.20
3.32
2.80
2.61
2.59
2.56
2.50 DMSO-d6
2.25
2.21
2.16
1.64
1.60
1.56
1.52
1.47
1.42
1.07
1.06
HO
N
OH
O
NMe2
1d
1H
0.91
0.95
3.75
2.85
1.00
1.86
1.01
0.95
0.92
5.65
f1 (ppm)

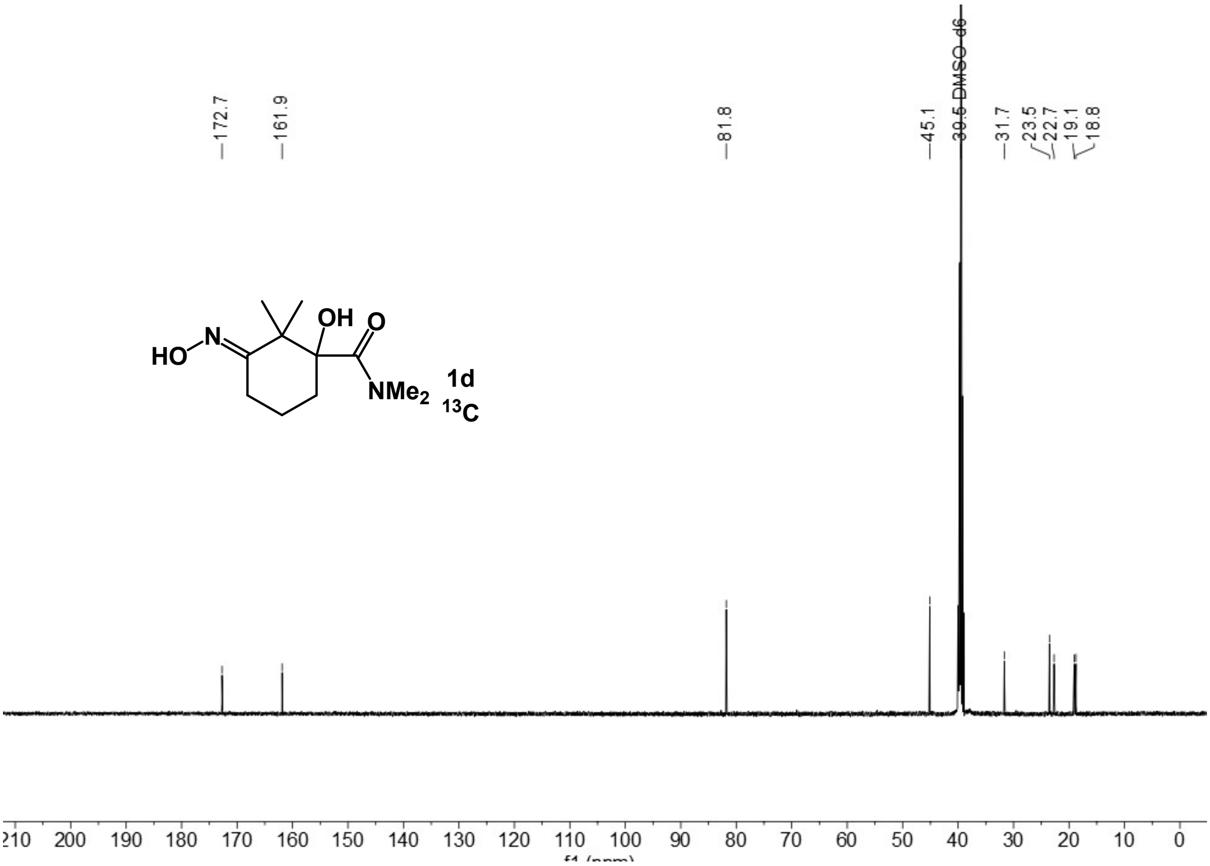
172.7
161.9
81.8
45.1
39.5 DMSO-d6
31.7
23.5
22.7
19.1
18.8
HO
N
OH
O
NMe2
1d
13C
f1 (ppm)

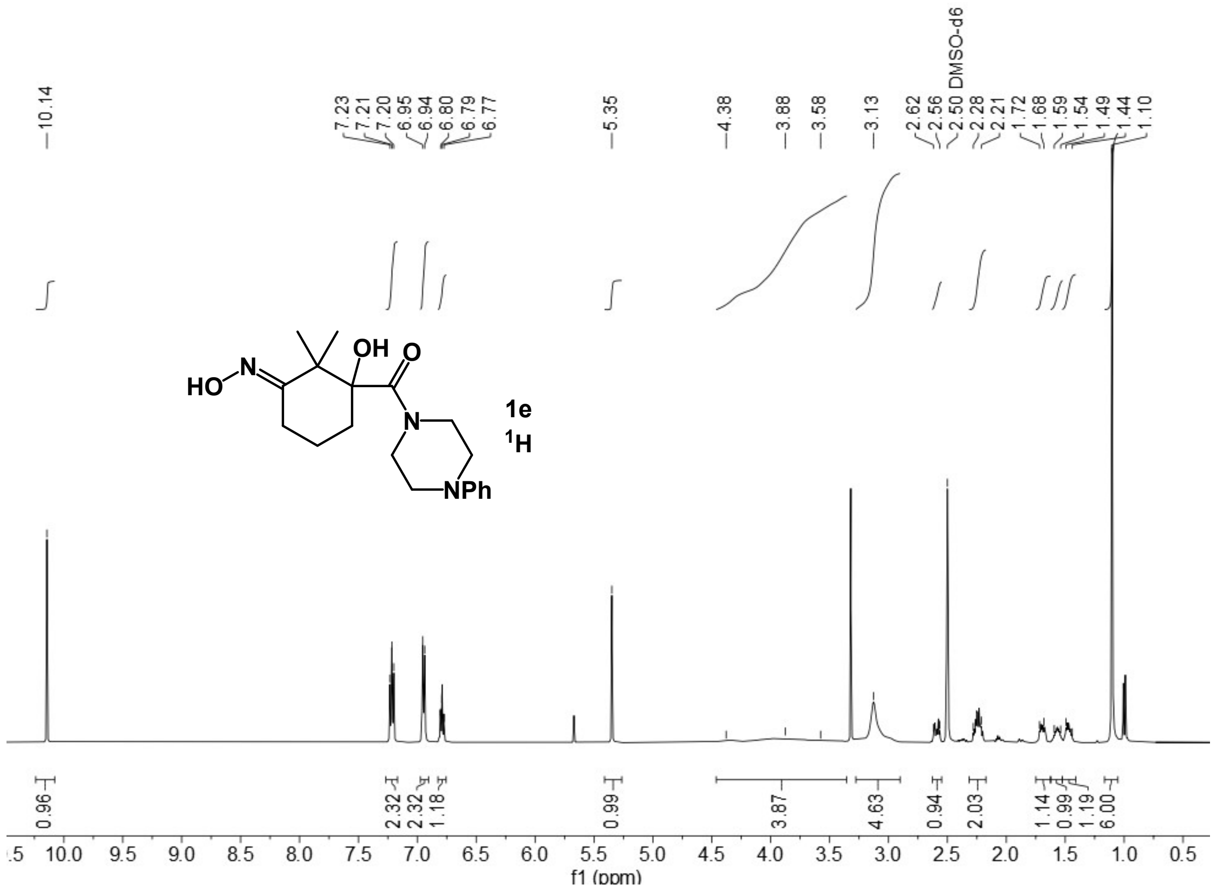
10.14
7.23 7.21 7.20 6.95 6.94 6.80 6.79 6.77
5.35
4.38
3.88
3.58
3.13
2.62 2.56 2.50 DMSO-d6 2.28 2.21 1.72 1.68 1.59 1.54 1.49 1.44 1.10
HO N OH O N NPh
1e
1H
0.96 2.32 2.32 1.18 0.99 3.87 4.63 0.94 2.03 1.14 0.99 1.19 6.00
f1 (ppm)

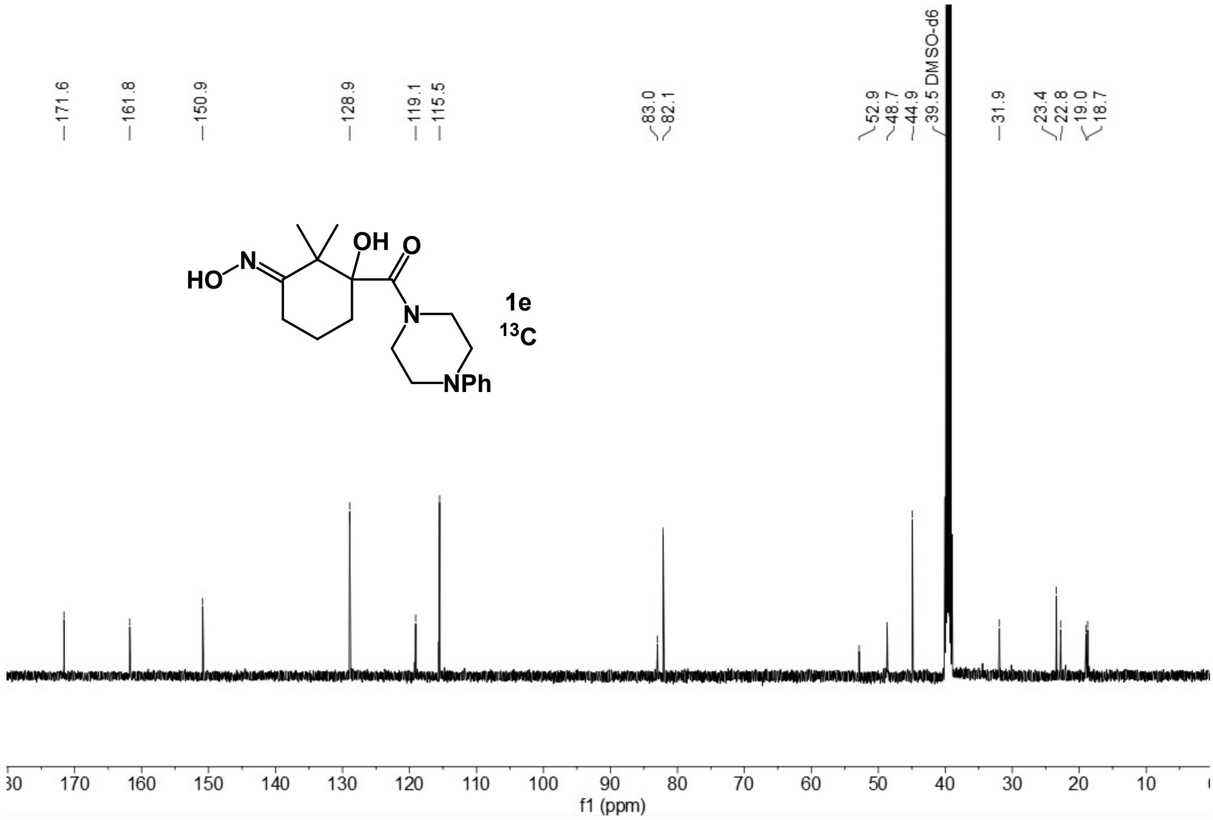
171.6
161.8
150.9
128.9
119.1
115.5
83.0 82.1
52.9 48.7 44.9 39.5 DMSO-d6
31.9
23.4 22.8 19.0 18.7
HO N OH O N NPh
1e
13C
f1 (ppm)

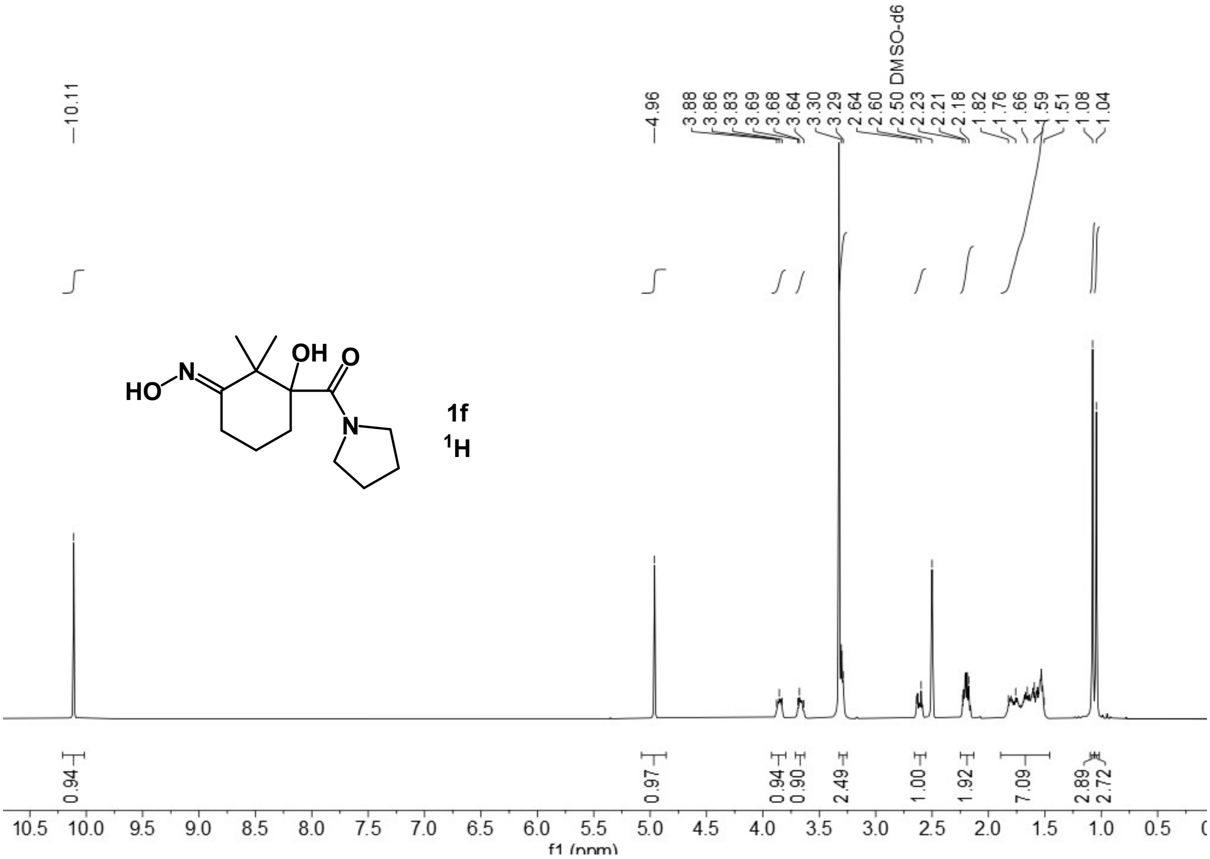

1f
1H
HO
N
OH
O
N
10.11
4.96
3.88 3.86 3.83 3.69 3.68 3.64 3.30 3.29 2.64 2.60 2.50 DMSO-d6 2.23 2.21 2.18 1.82 1.76 1.66 1.59 1.51 1.08 1.04
0.94 0.97 0.94 0.90 2.49 1.00 1.92 7.09 2.89 2.72
10.5 10.0 9.5 9.0 8.5 8.0 7.5 7.0 6.5 6.0 5.5 5.0 4.5 4.0 3.5 3.0 2.5 2.0 1.5 1.0 0.5
f1 (ppm)

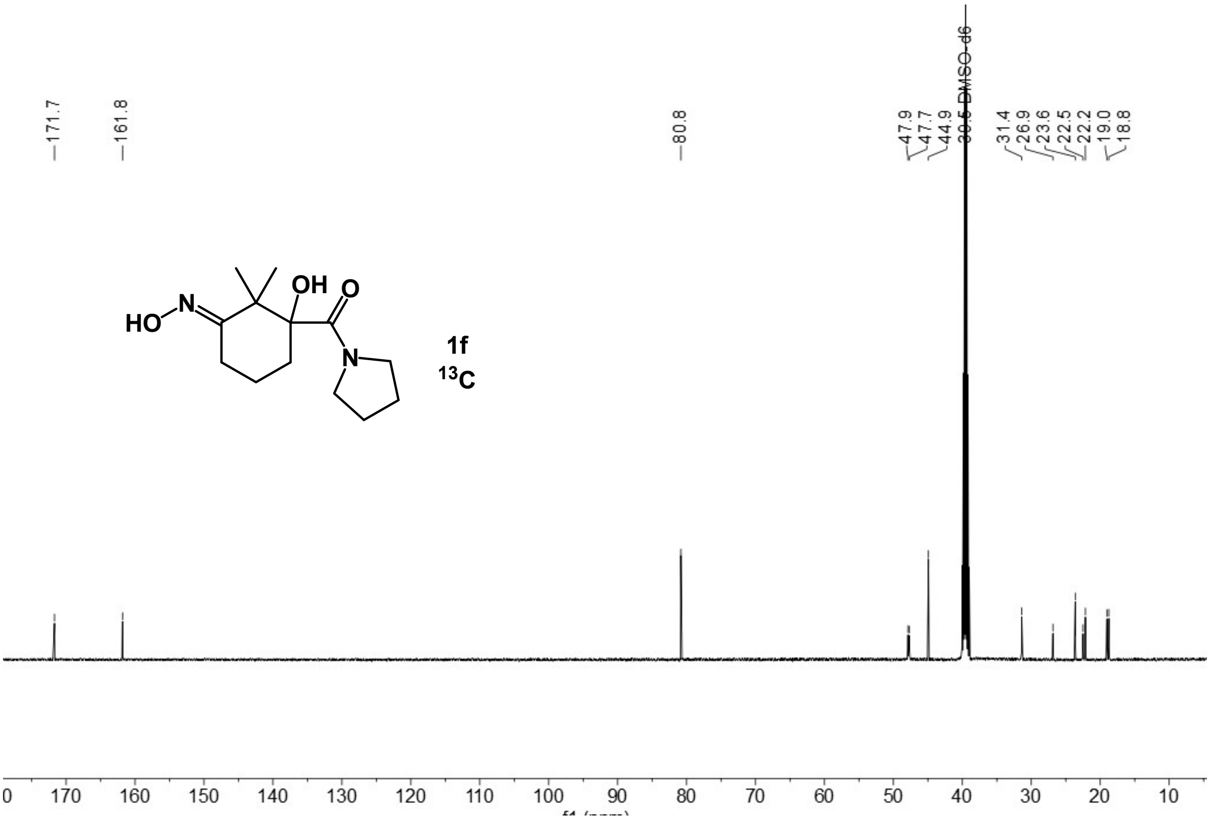

1f
13C
HO
N
OH
O
N
171.7
161.8
80.8
47.9 47.7 44.9 39.5 DMSO-d6
31.4 26.9 23.6 22.5 22.2 19.0 18.8
170 160 150 140 130 120 110 100 90 80 70 60 50 40 30 20 10

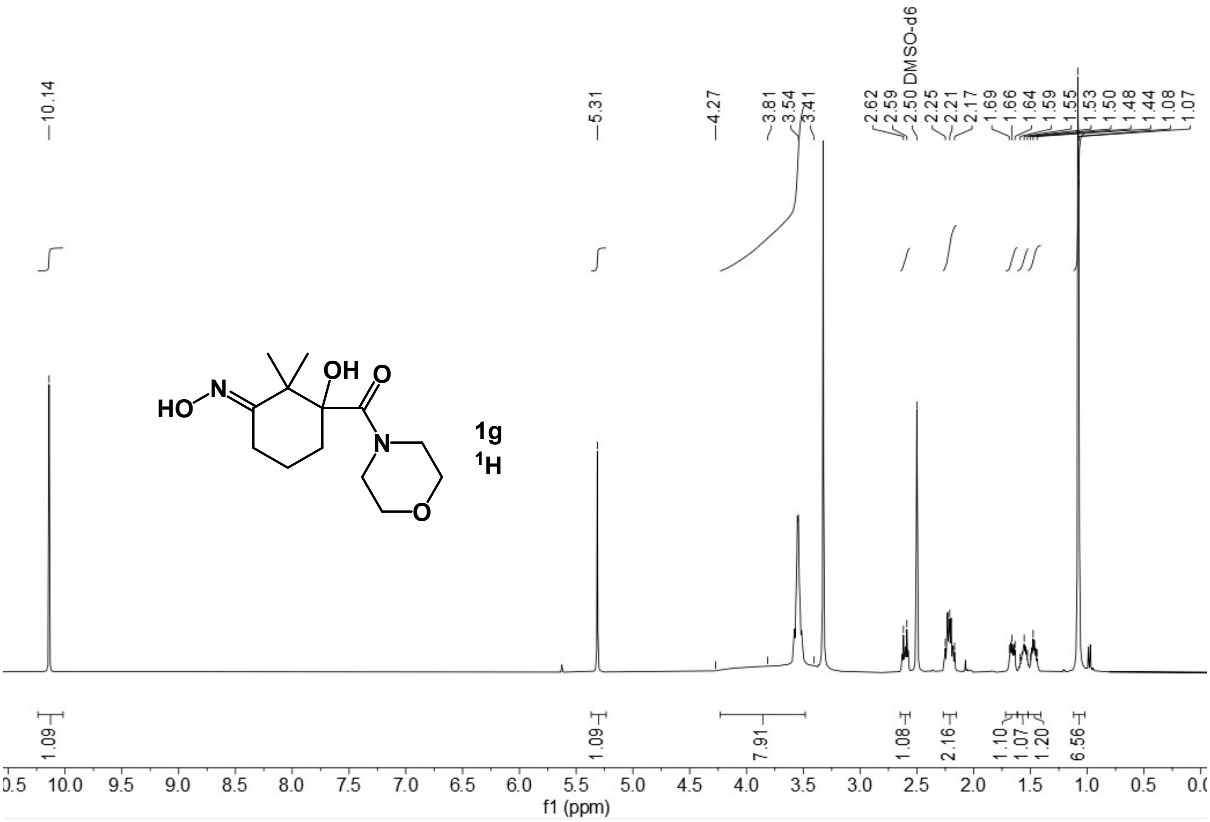

1g
^{1}H
10.14
5.31
4.27
3.81
3.54
3.41
2.62
2.59
2.50 DMSO-d6
2.25
2.21
2.17
1.69
1.66
1.64
1.59
1.55
1.53
1.50
1.48
1.44
1.08
1.07
1.09
1.09
7.91
1.08
2.16
1.10
1.07
1.20
6.56
f1 (ppm)

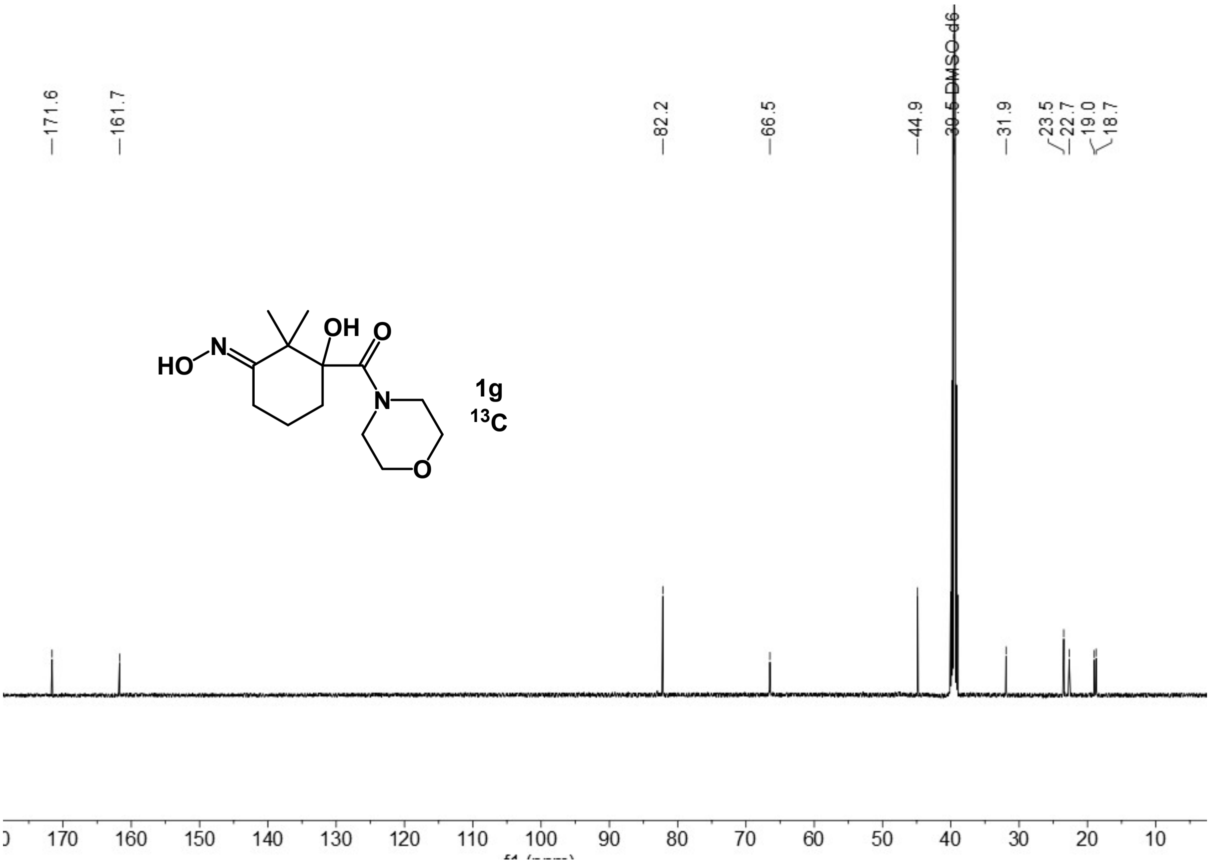

1g
^{13}C
171.6
161.7
82.2
66.5
44.9
39.5 DMSO-d6
31.9
23.5
22.7
19.0
18.7

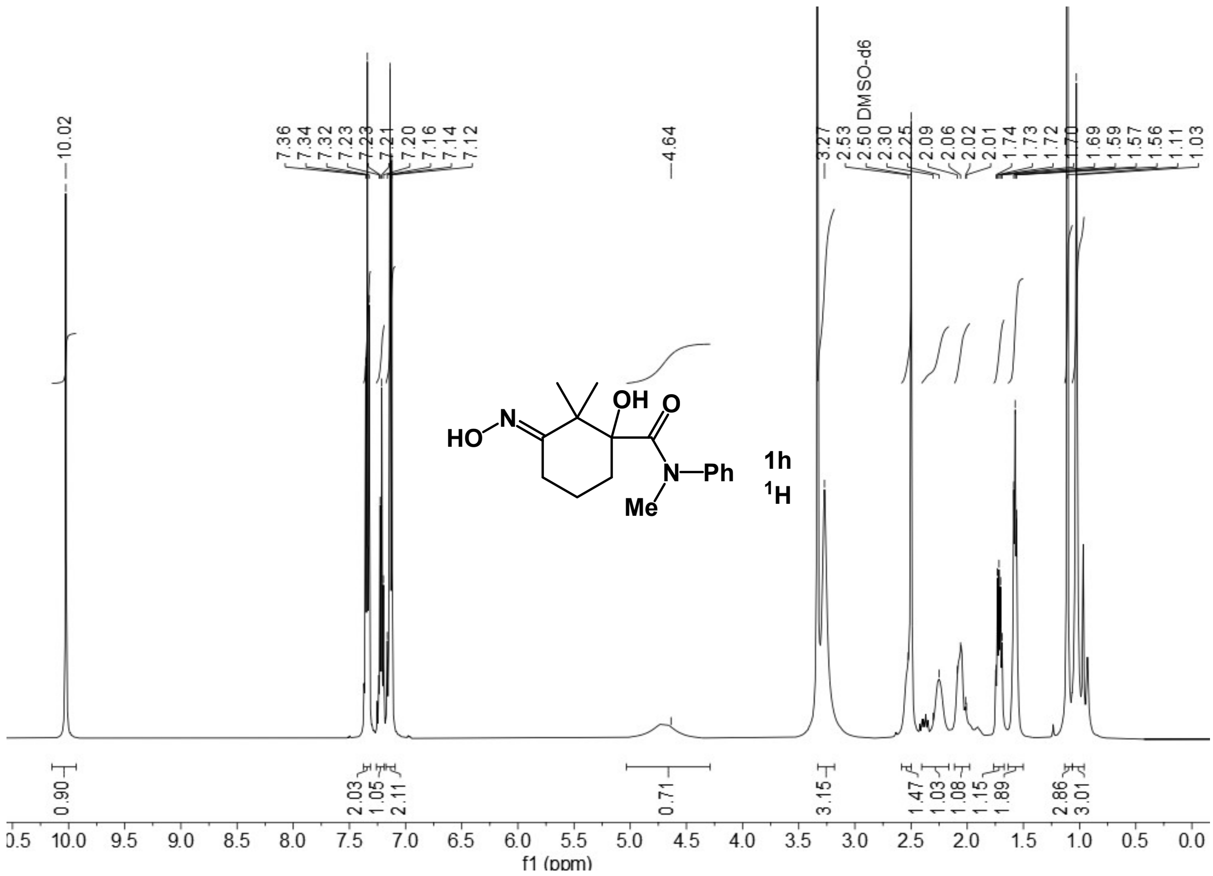
10.02
7.36 7.34 7.32 7.23 7.23 7.21 7.20 7.16 7.14 7.12
4.64
3.27
2.53
2.50 DMSO-d6
2.30 2.25 2.09 2.06 2.02 2.01 1.74 1.73 1.72 1.70 1.69 1.59 1.57 1.56 1.11 1.03
HO
N
OH
O
N—Ph
Me
1h
^{1}H
0.90
2.03 1.05 2.11
0.71
3.15
1.47 1.03 1.08 1.15 1.89
2.86 3.01
f1 (ppm)

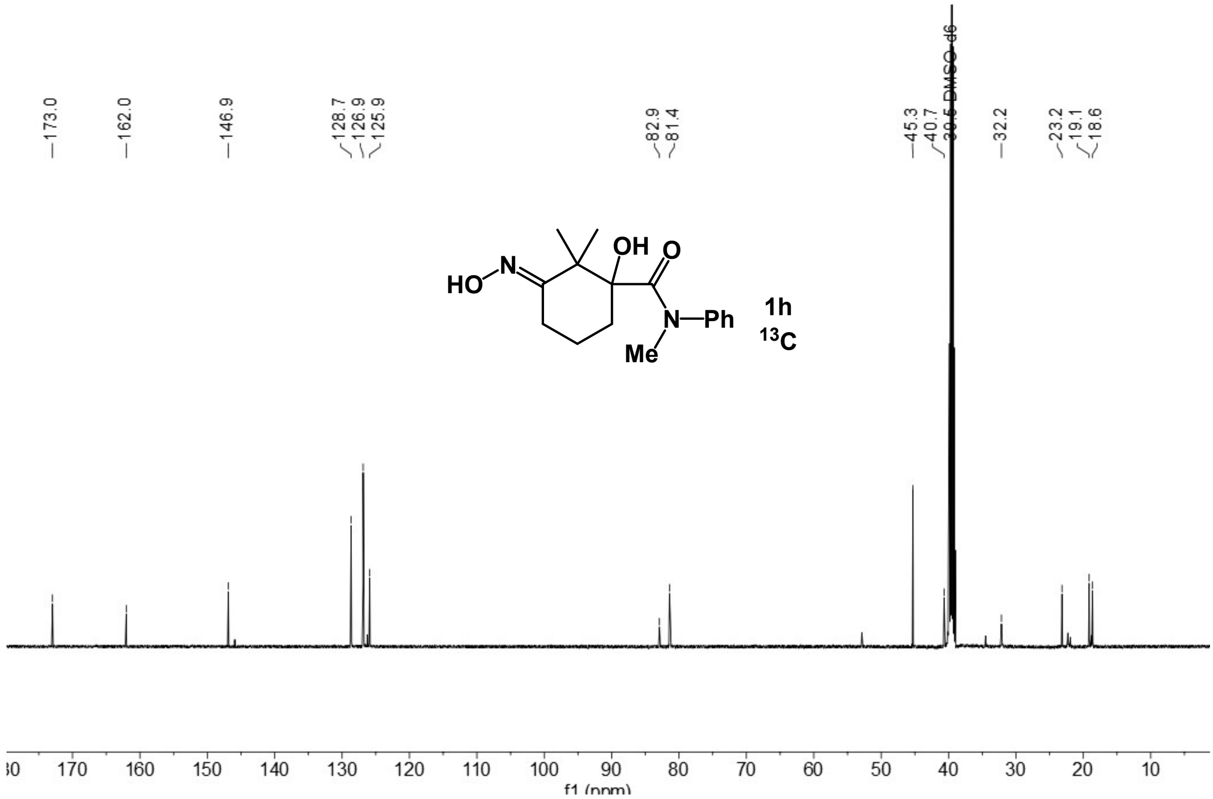
173.0
162.0
146.9
128.7 126.9 125.9
82.9 81.4
45.3 40.7
39.5 DMSO-d6
32.2
23.2 19.1 18.6
HO
N
OH
O
N—Ph
Me
1h
^{13}C
f1 (ppm)

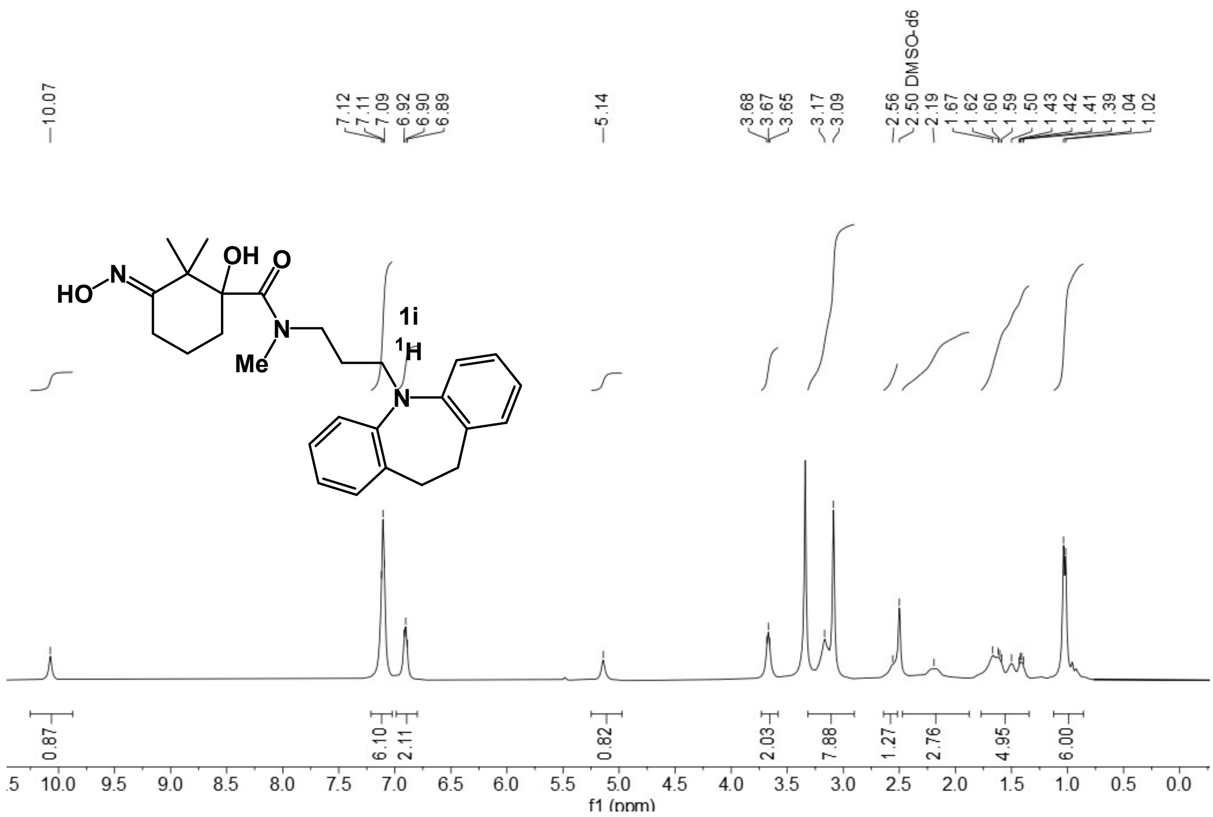
10.07
7.12 7.11 7.09 6.92 6.90 6.89
5.14
3.68 3.67 3.65 3.17 3.09
2.56 2.50 DMSO-d6 2.19 1.67 1.62 1.60 1.59 1.50 1.43 1.42 1.41 1.39 1.04 1.02
HO N OH O N Me N
1i
1H
0.87 6.10 2.11 0.82 2.03 7.88 1.27 2.76 4.95 6.00
f1 (ppm)

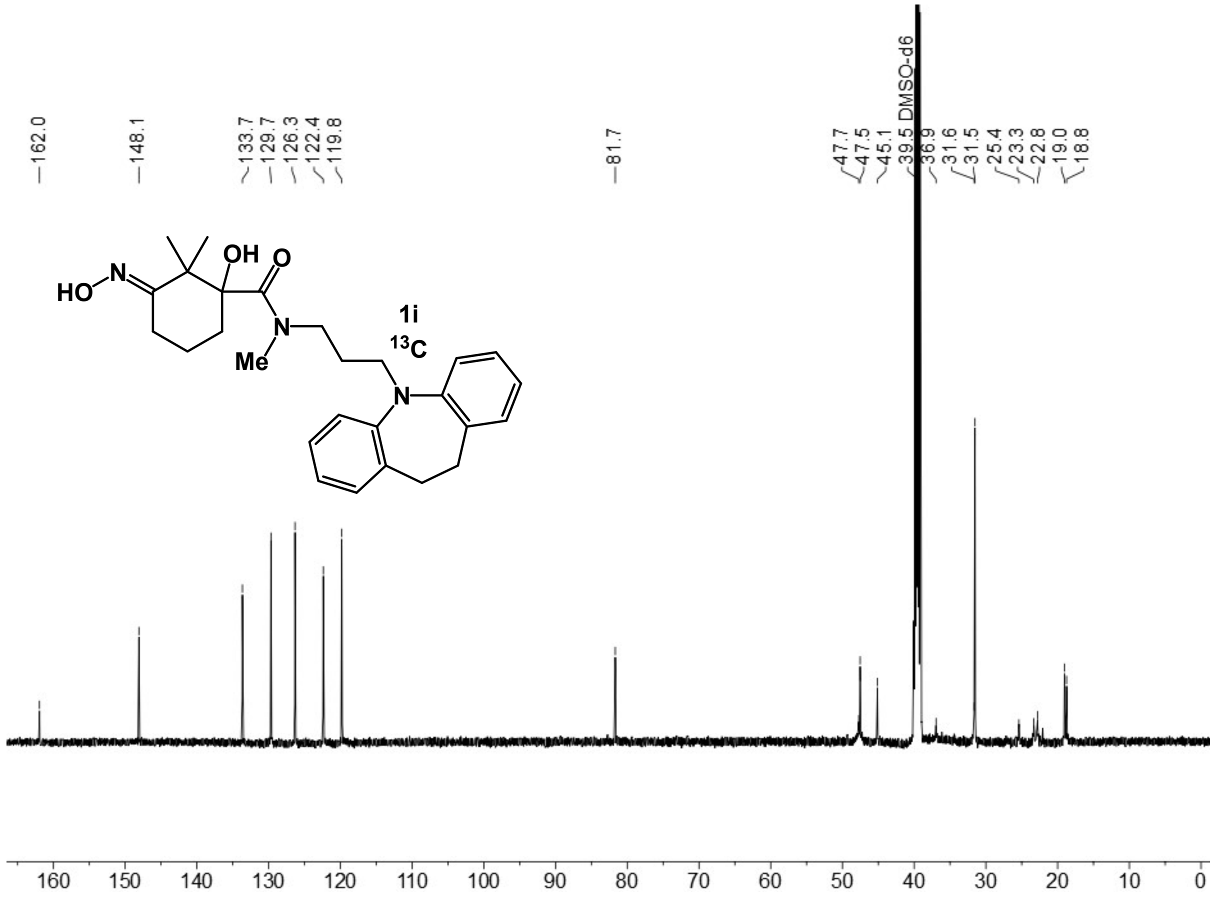
162.0
148.1
133.7 129.7 126.3 122.4 119.8
81.7
47.7 47.5 45.1 39.5 DMSO-d6 36.9 31.6 31.5 25.4 23.3 22.8 19.0 18.8
HO N OH O N Me N
1i
13C

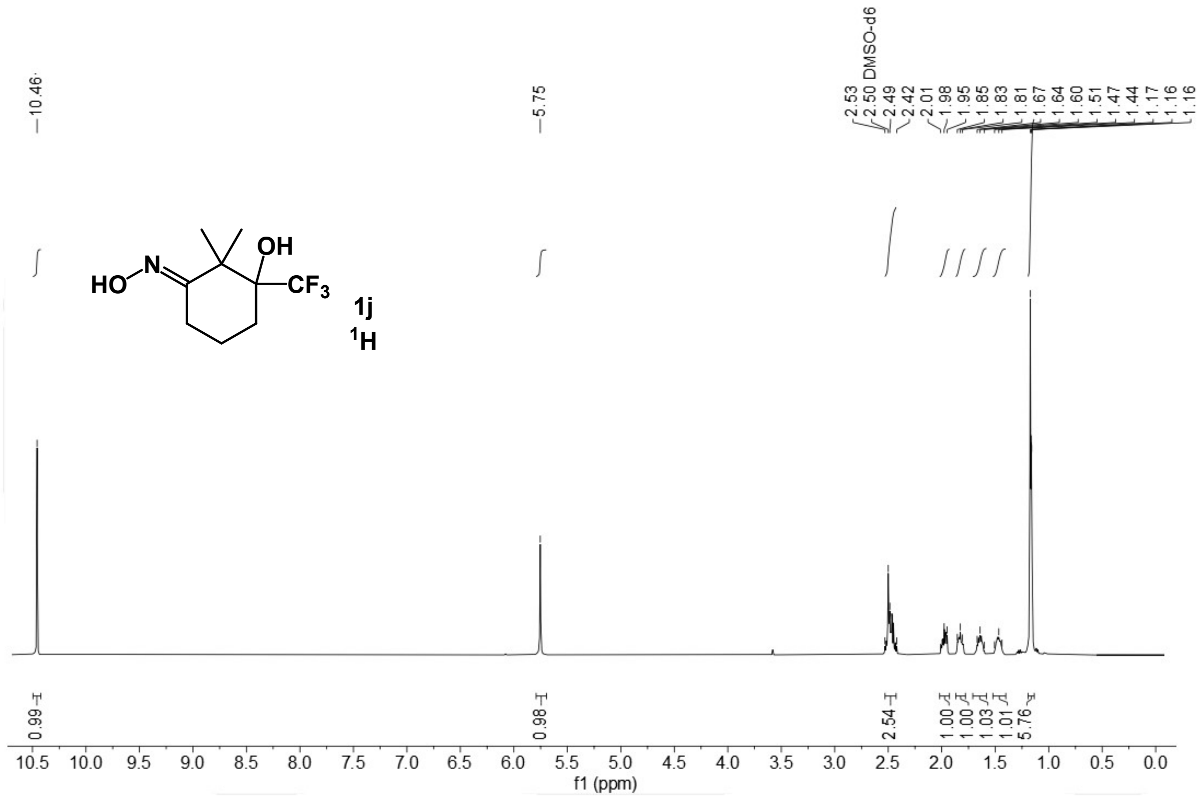
10.46
5.75
2.53
2.50 DMSO-d6
2.49
2.42
2.01
1.98
1.95
1.85
1.83
1.81
1.67
1.64
1.60
1.51
1.47
1.44
1.17
1.16
1.16
OH
HO
N
CF3
1j
1H
0.99
0.98
2.54
1.00
1.00
1.03
1.01
5.76
f1 (ppm)

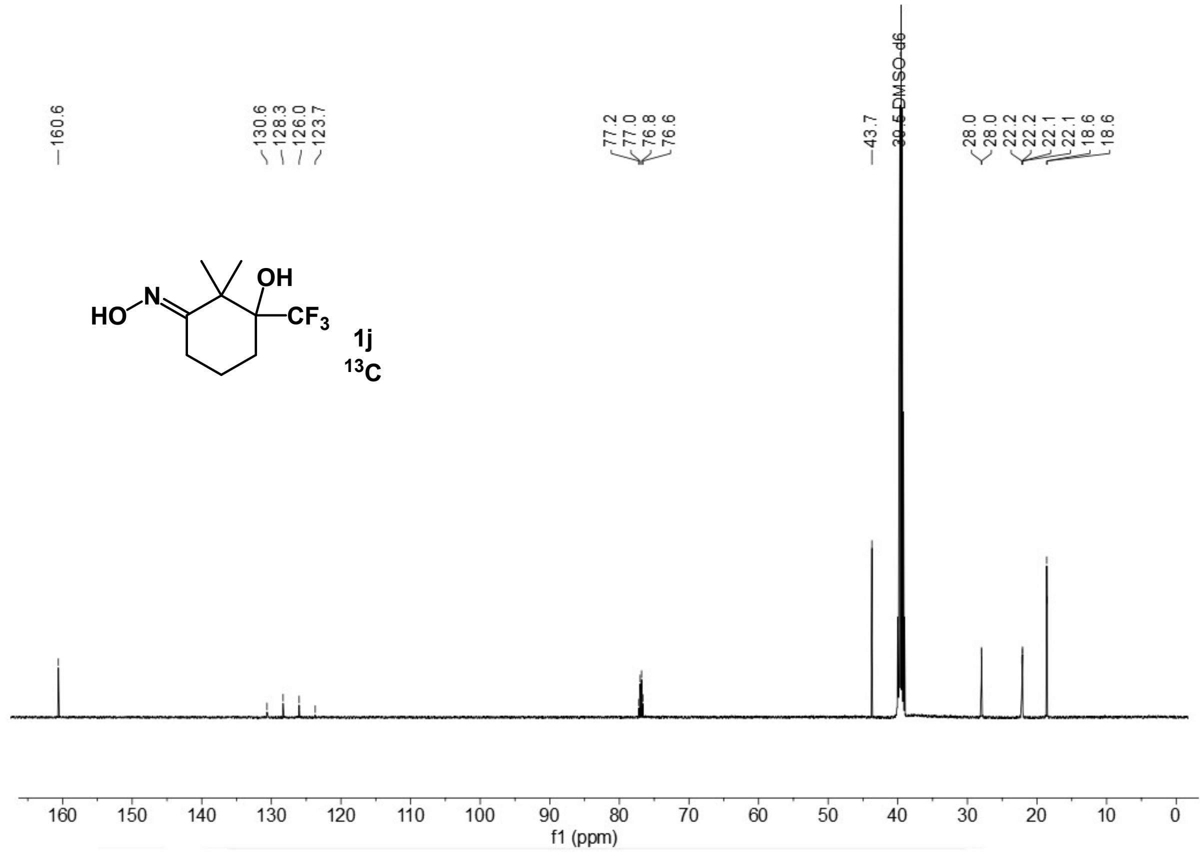
160.6
130.6
128.3
126.0
123.7
77.2
77.0
76.8
76.6
43.7
39.5 DMSO-d6
28.0
28.0
22.2
22.1
22.1
18.6
18.6
OH
HO
N
CF3
1j
13C
f1 (ppm)

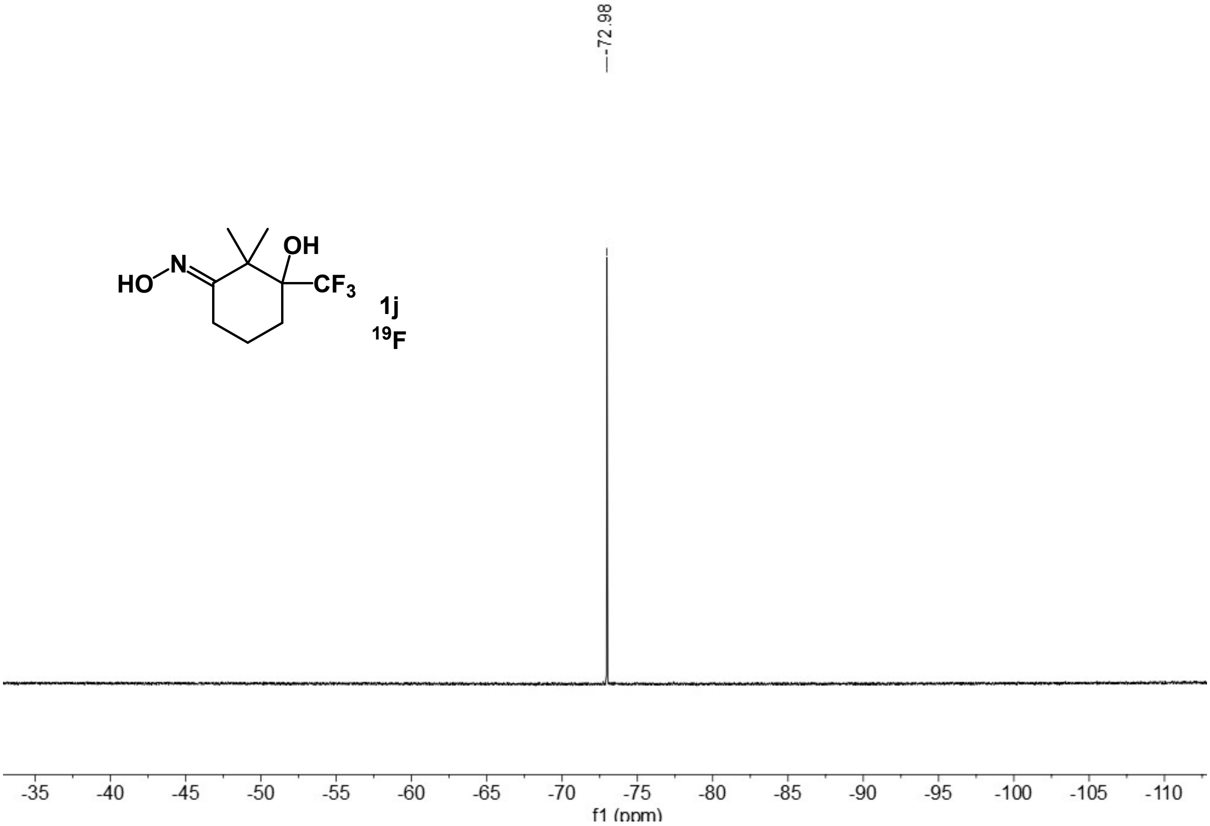
HO
N
OH
CF3
1j
19F
-72.98
-35
-40
-45
-50
-55
-60
-65
-70
-75
-80
-85
-90
-95
-100
-105
-110
f1 (ppm)

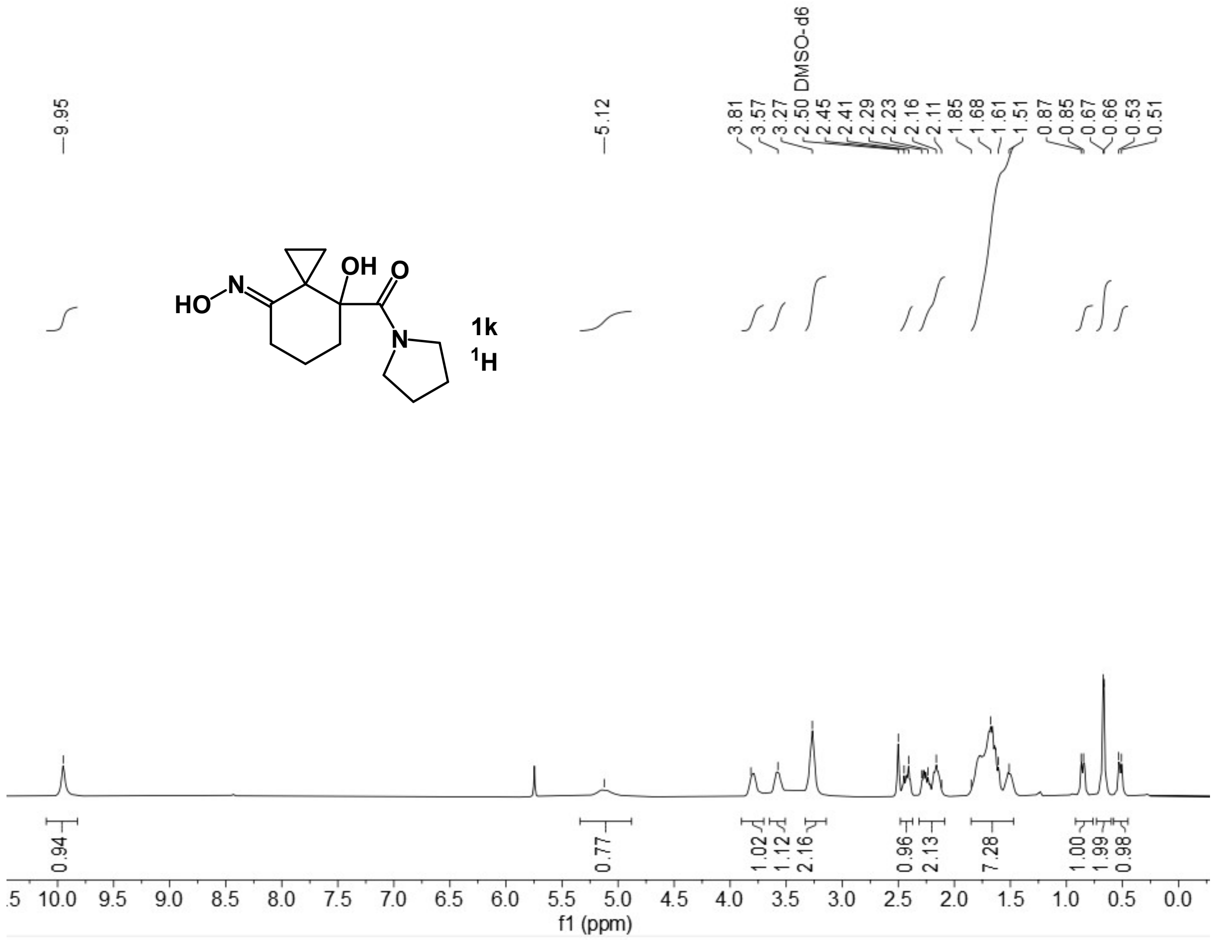
1k
^{1}H
f1 (ppm)

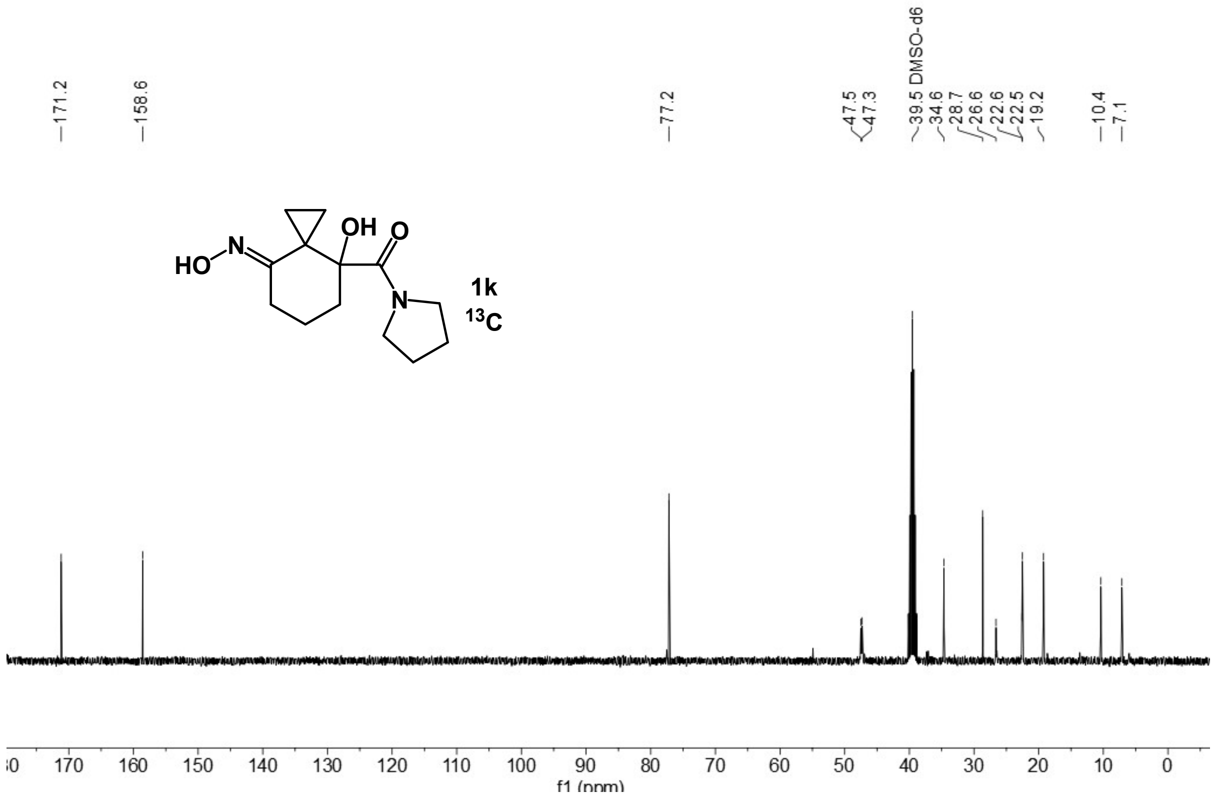
1k
^{13}C
f1 (ppm)

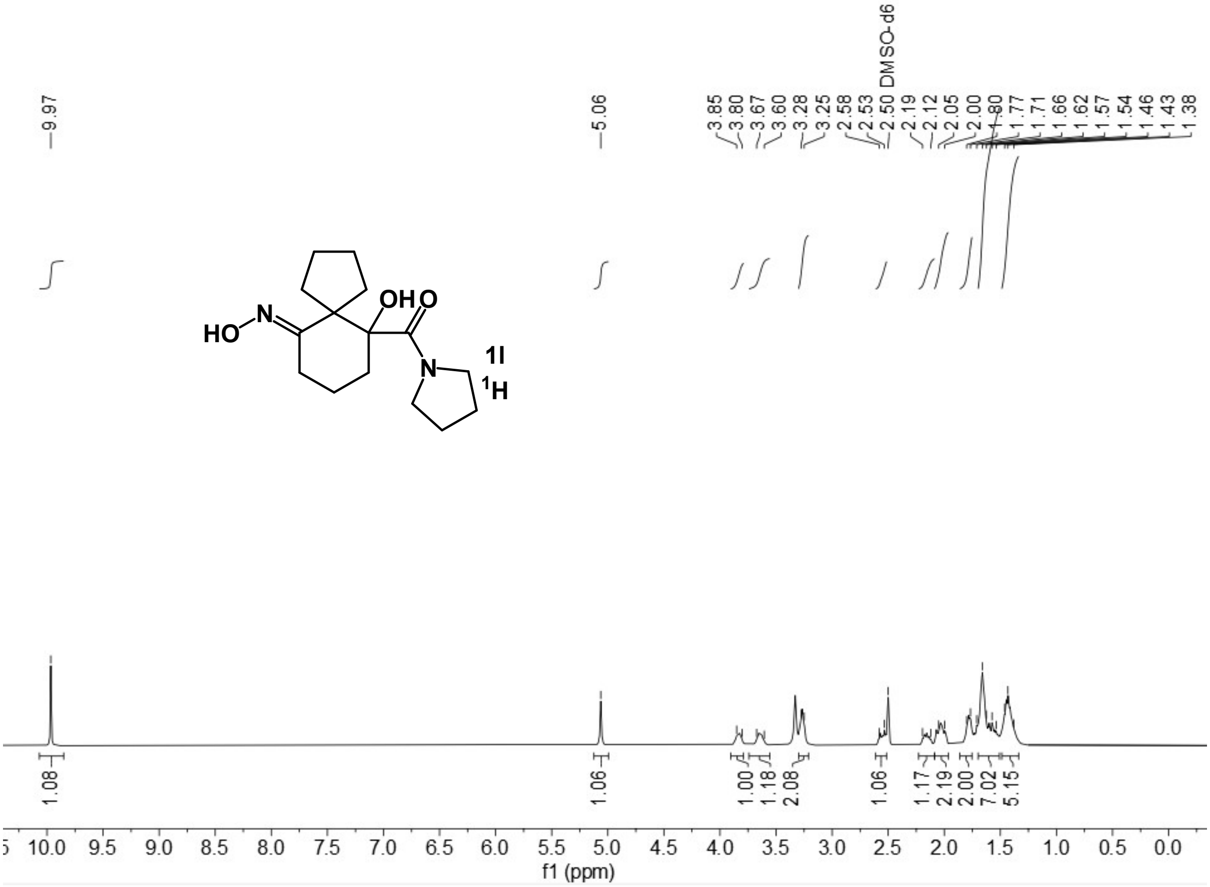
9.97
5.06
3.85
3.80
3.67
3.60
3.28
3.25
2.58
2.53
2.50 DMSO-d6
2.19
2.12
2.05
2.00
1.80
1.77
1.71
1.66
1.62
1.57
1.54
1.46
1.43
1.38
HO
N
OH
O
N
1l
1H
1.08
1.06
1.00
1.18
2.08
1.06
1.17
2.19
2.00
7.02
5.15
f1 (ppm)

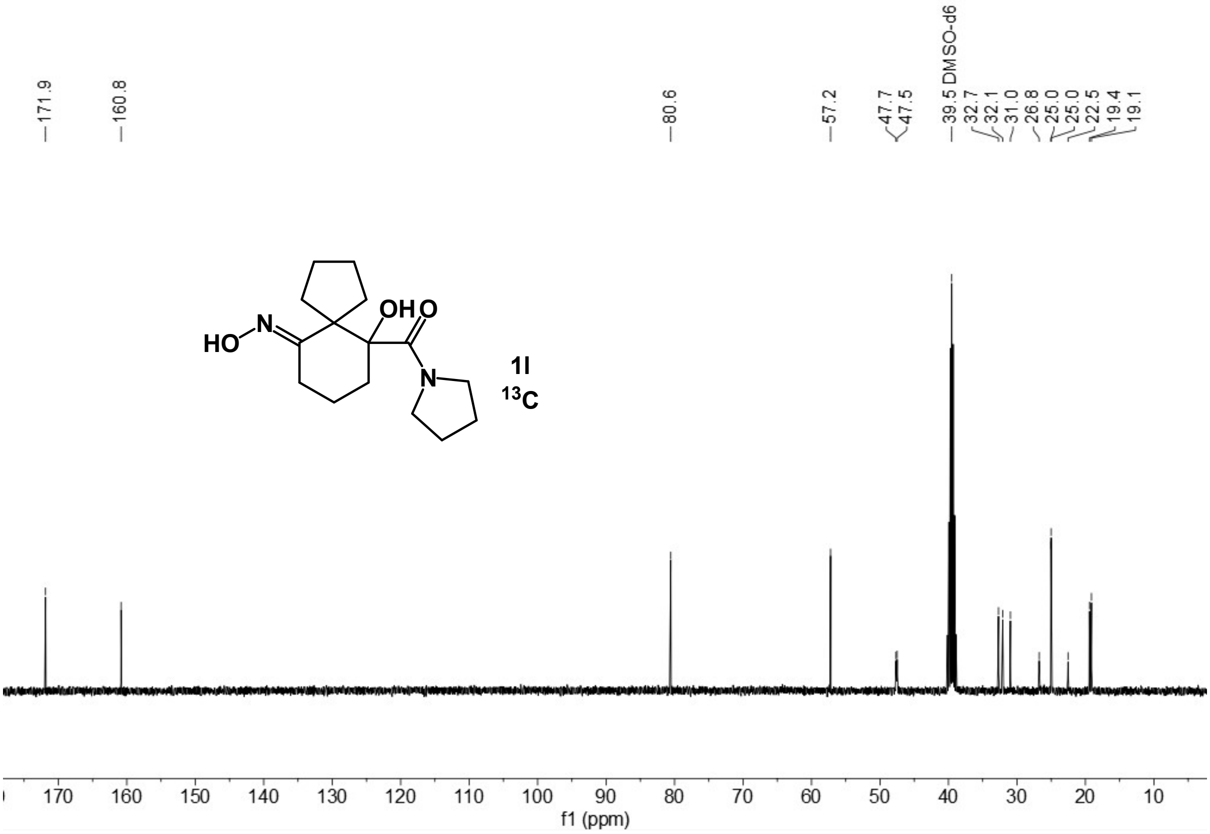
171.9
160.8
80.6
57.2
47.7
47.5
39.5 DMSO-d6
32.7
32.1
31.0
26.8
25.0
25.0
22.5
19.4
19.1
HO
N
OH
O
N
1l
13C
f1 (ppm)

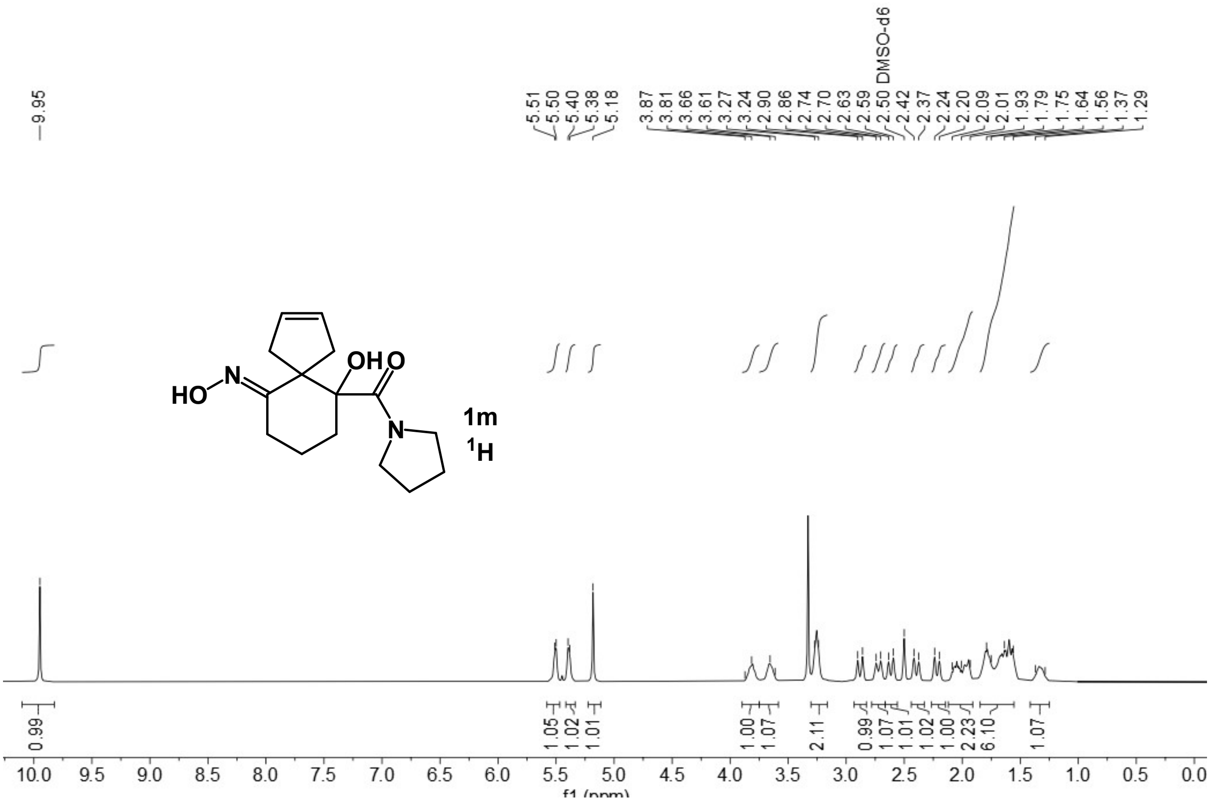
9.95
5.51
5.50
5.40
5.38
5.18
3.87
3.81
3.66
3.61
3.27
3.24
2.90
2.86
2.74
2.70
2.63
2.59
2.50 DMSO-d6
2.42
2.37
2.24
2.20
2.09
2.01
1.93
1.79
1.75
1.64
1.56
1.37
1.29
HO
N
OH
O
N
1m
1H
0.99
1.05
1.02
1.01
1.00
1.07
2.11
0.99
1.07
1.01
1.02
1.00
2.23
6.10
1.07
10.0 9.5 9.0 8.5 8.0 7.5 7.0 6.5 6.0 5.5 5.0 4.5 4.0 3.5 3.0 2.5 2.0 1.5 1.0 0.5 0.0
f1 (ppm)

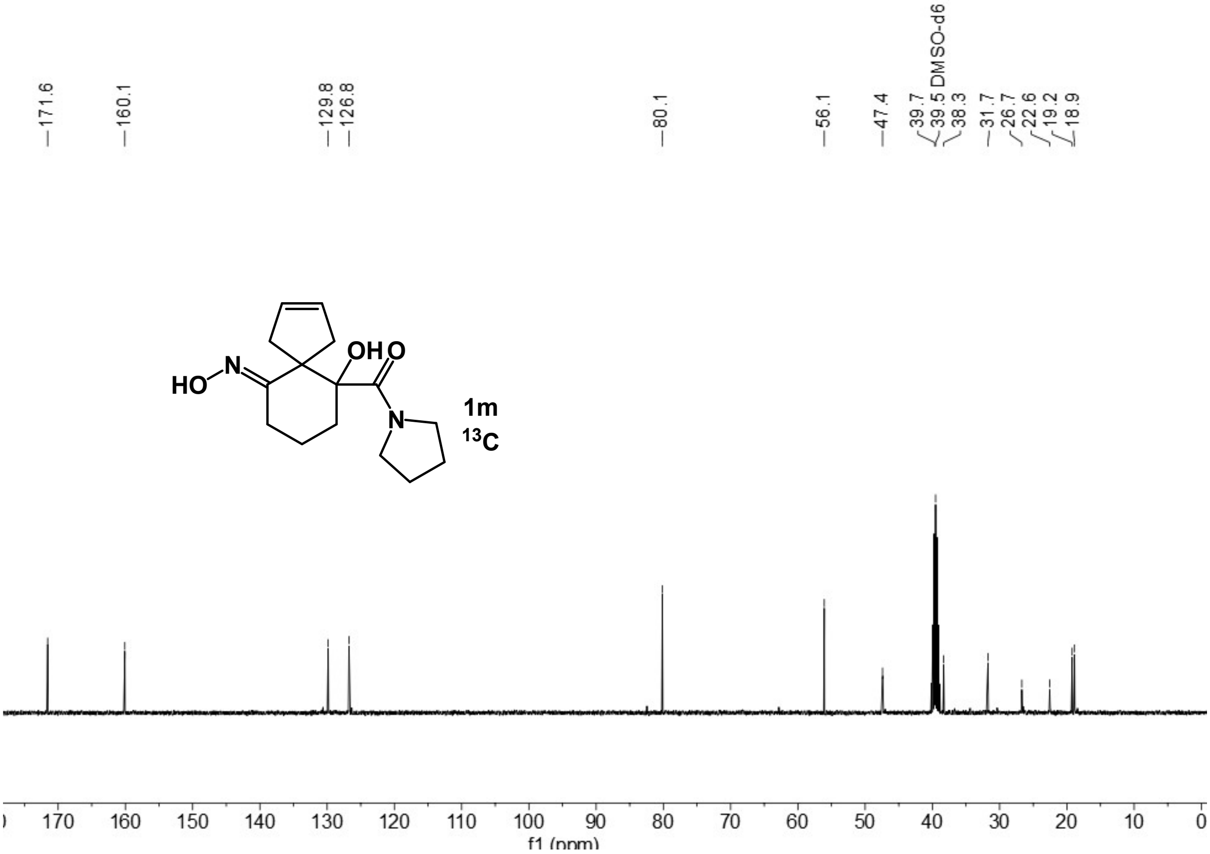
171.6
160.1
129.8
126.8
80.1
56.1
47.4
39.7
39.5 DMSO-d6
38.3
31.7
26.7
22.6
19.2
18.9
HO
N
OH
O
N
1m
13C
170 160 150 140 130 120 110 100 90 80 70 60 50 40 30 20 10 0
f1 (ppm)

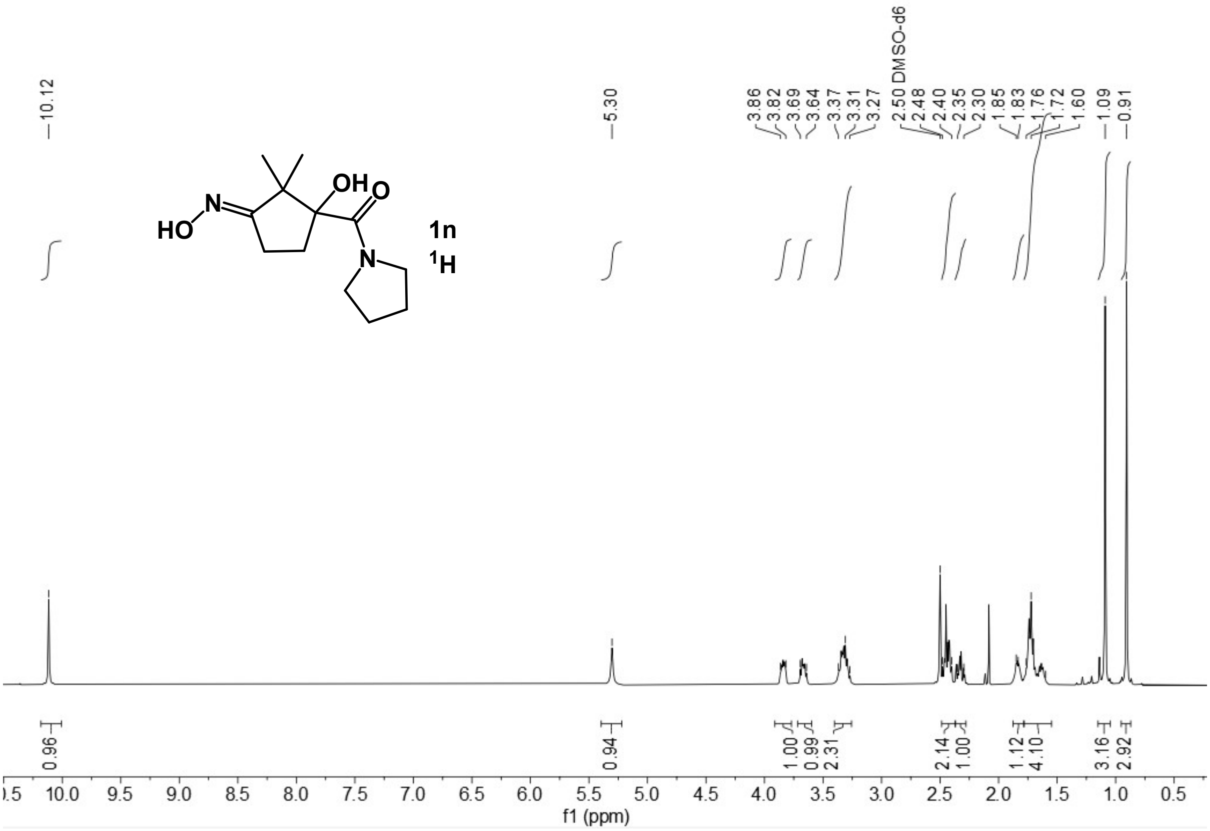
1n
1H
10.12
5.30
3.86 3.82 3.69 3.64 3.37 3.31 3.27
2.50 DMSO-d6
2.48 2.40 2.35 2.30 1.85 1.83 1.76 1.72 1.60 1.09 0.91
0.96 0.94 1.00 0.99 2.31 2.14 1.00 1.12 4.10 3.16 2.92
f1 (ppm)

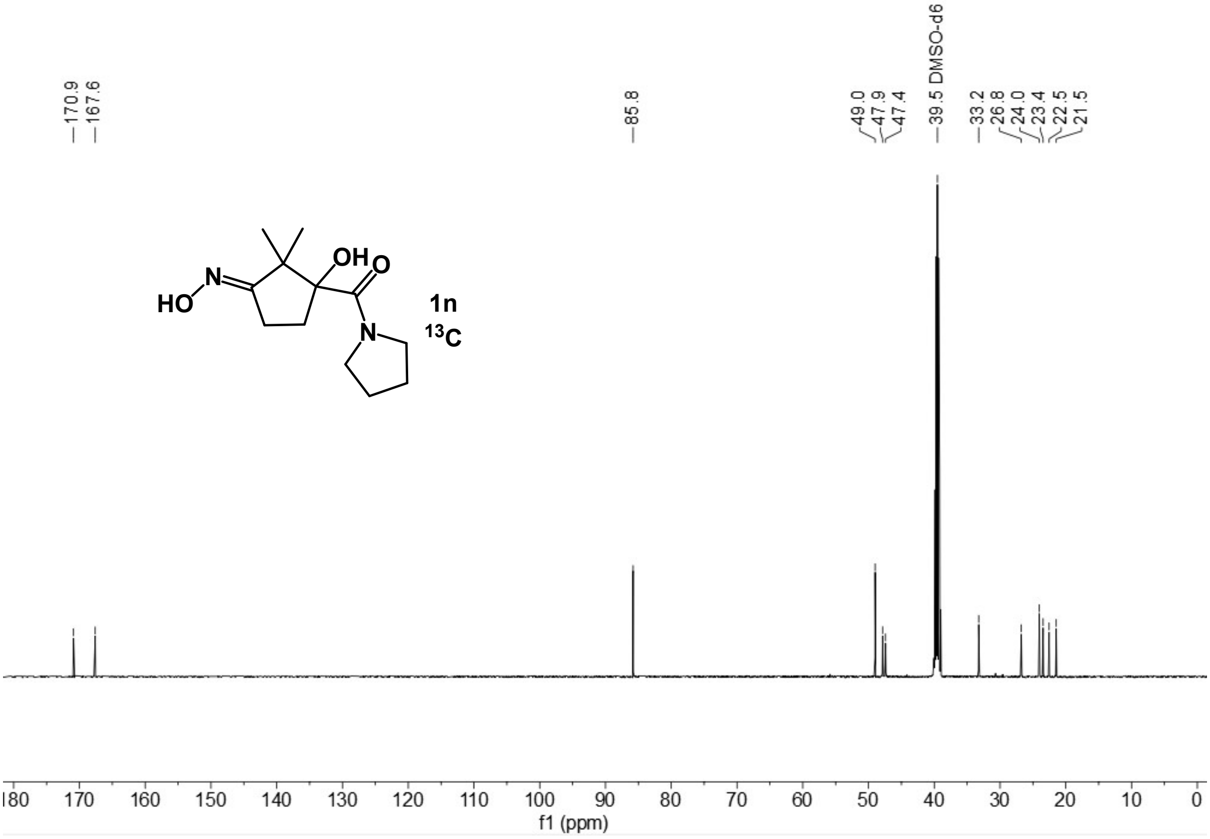
1n
13C
170.9 167.6
85.8
49.0 47.9 47.4
39.5 DMSO-d6
33.2 26.8 24.0 23.4 22.5 21.5
f1 (ppm)

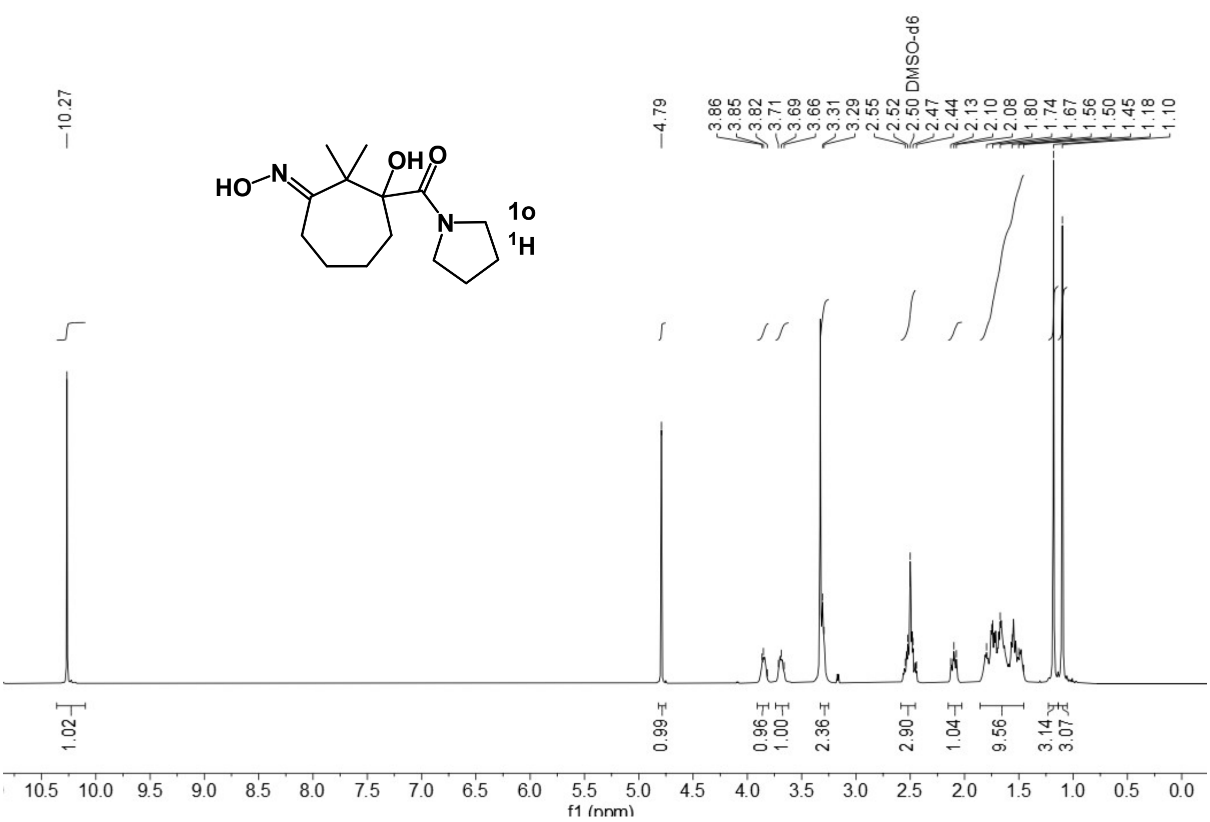
1o
^{1}H
HO
N
OH
O
N
10.27
4.79
3.86
3.85
3.82
3.71
3.69
3.66
3.31
3.29
2.55
2.52
2.50 DMSO-d6
2.47
2.44
2.13
2.10
2.08
1.80
1.74
1.67
1.56
1.50
1.45
1.18
1.10
1.02
0.99
0.96
1.00
2.36
2.90
1.04
9.56
3.14
3.07
f1 (ppm)

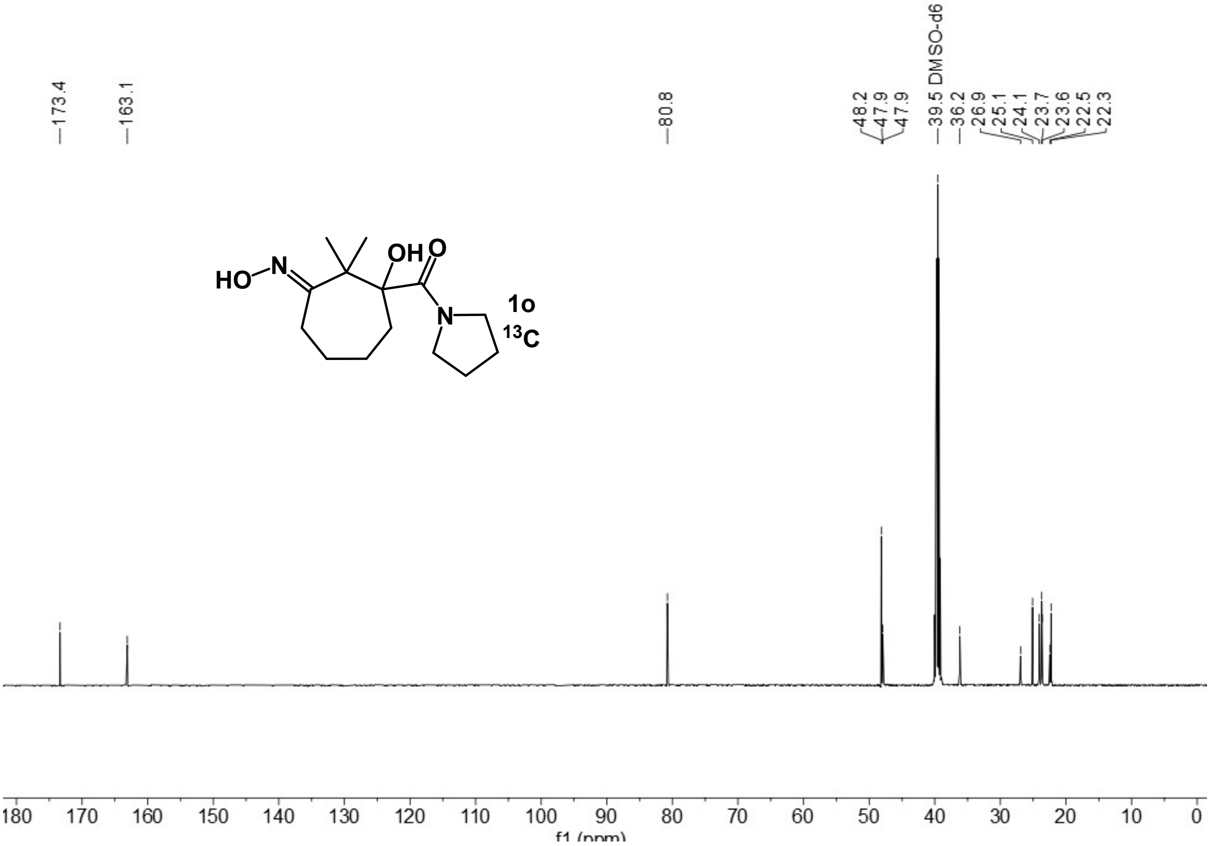
1o
^{13}C
HO
N
OH
O
N
173.4
163.1
80.8
48.2
47.9
47.9
39.5 DMSO-d6
36.2
26.9
25.1
24.1
23.7
23.6
22.5
22.3
f1 (ppm)

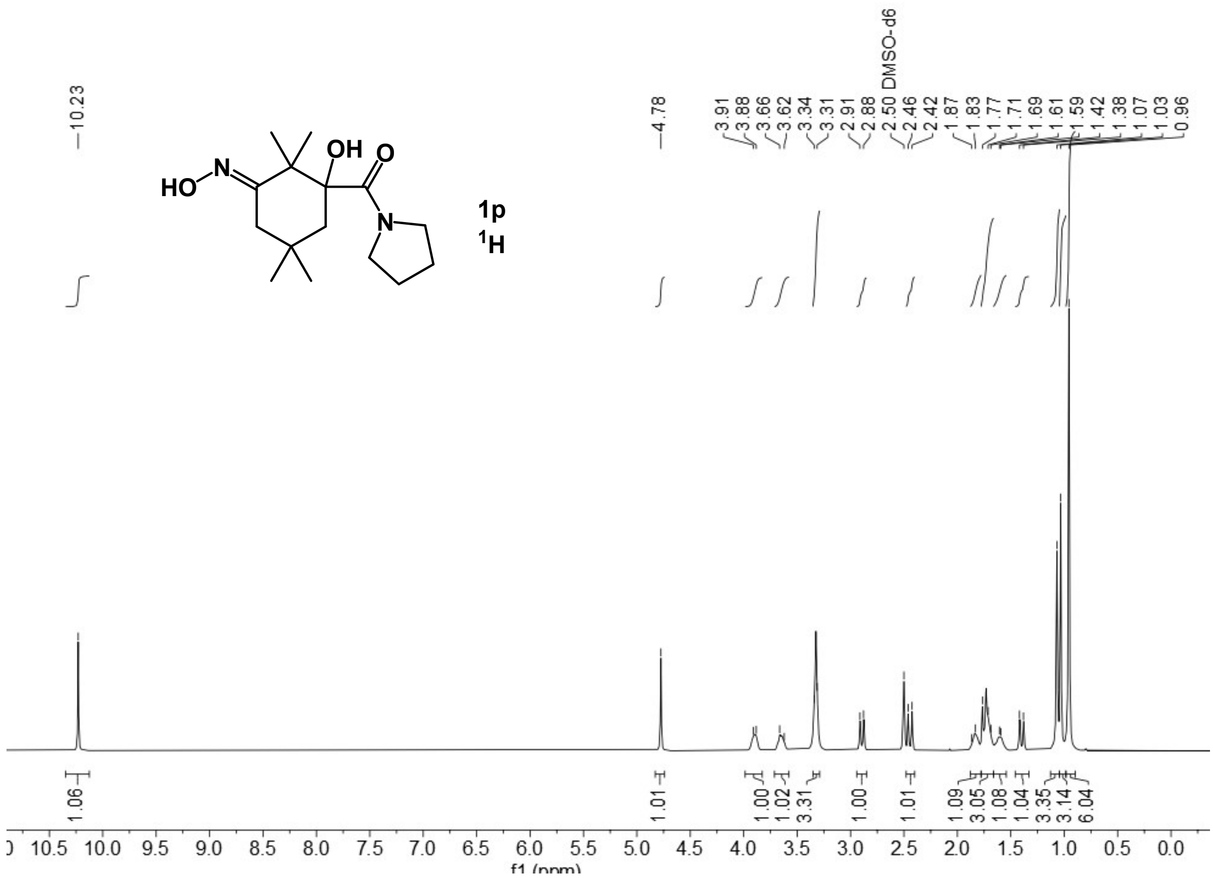

—10.23
—4.78
3.91
3.88
3.66
3.62
3.34
3.31
2.91
2.88
2.50 DMSO-d6
2.46
2.42
1.87
1.83
1.77
1.71
1.69
1.61
1.59
1.42
1.38
1.07
1.03
0.96
1p
1H
1.06
1.01
1.00
1.02
3.31
1.00
1.01
1.09
3.05
1.08
1.04
3.35
3.14
6.04
f1 (ppm)

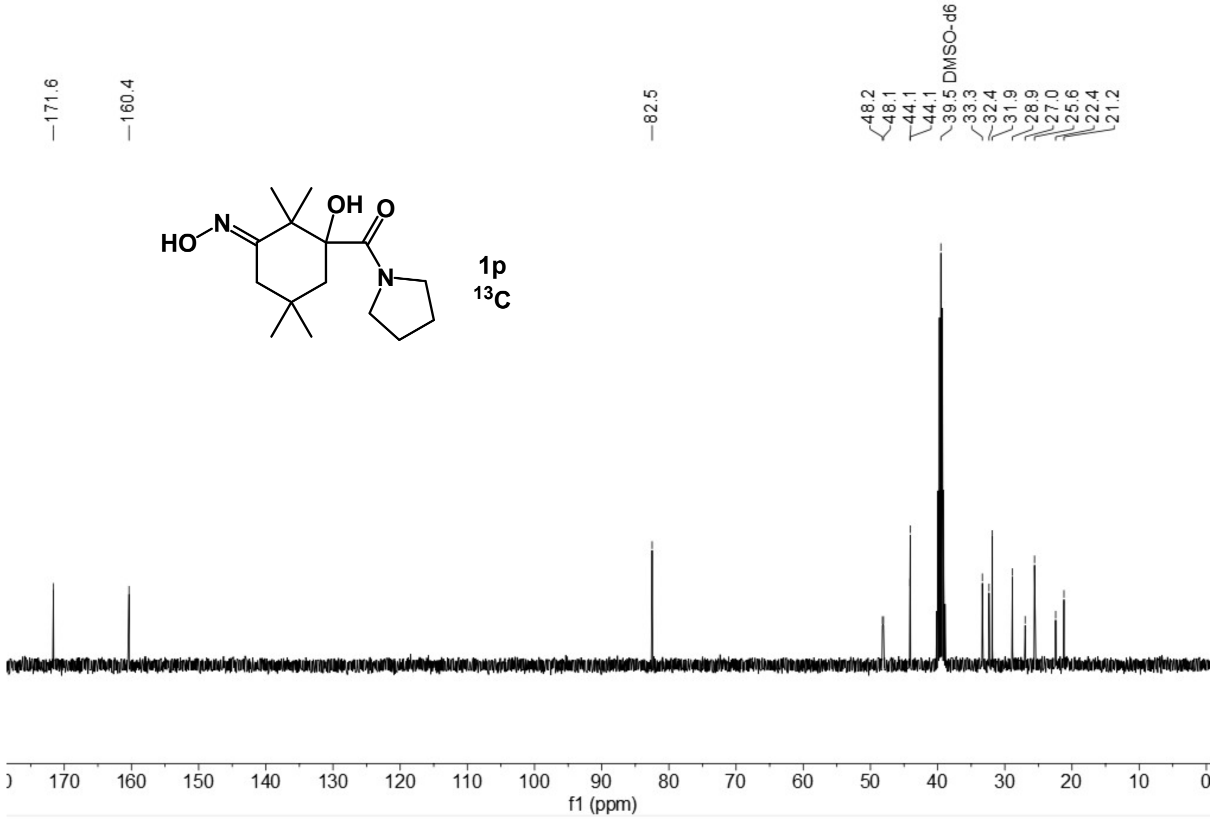

—171.6
—160.4
—82.5
48.2
48.1
44.1
44.1
39.5 DMSO-d6
33.3
32.4
31.9
28.9
27.0
25.6
22.4
21.2
1p
13C
f1 (ppm)

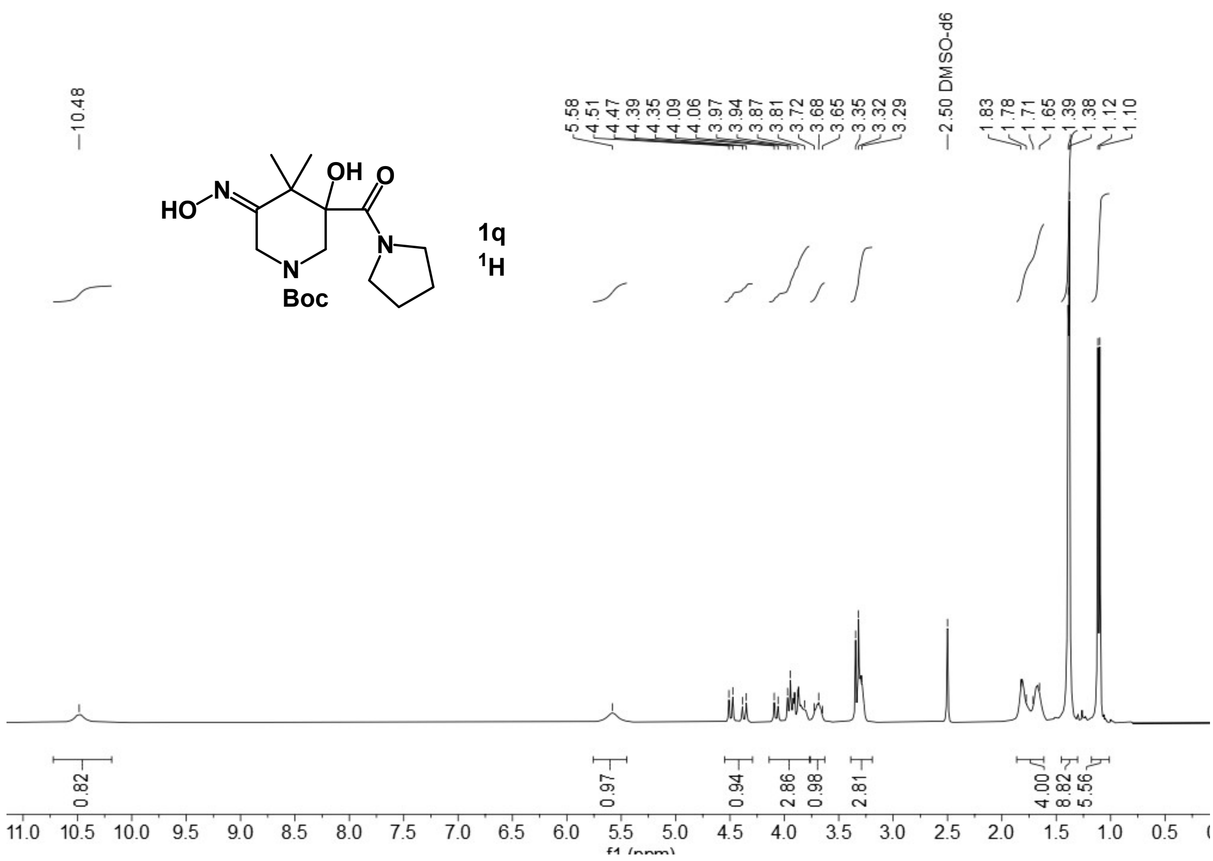
1q
1H
HO
N
OH
O
N
Boc
10.48
5.58
4.51
4.47
4.39
4.35
4.09
4.06
3.97
3.94
3.87
3.81
3.72
3.68
3.65
3.35
3.32
3.29
2.50 DMSO-d6
1.83
1.78
1.71
1.65
1.39
1.38
1.12
1.10
0.82
0.97
0.94
2.86
0.98
2.81
4.00
8.82
5.56
f1 (ppm)

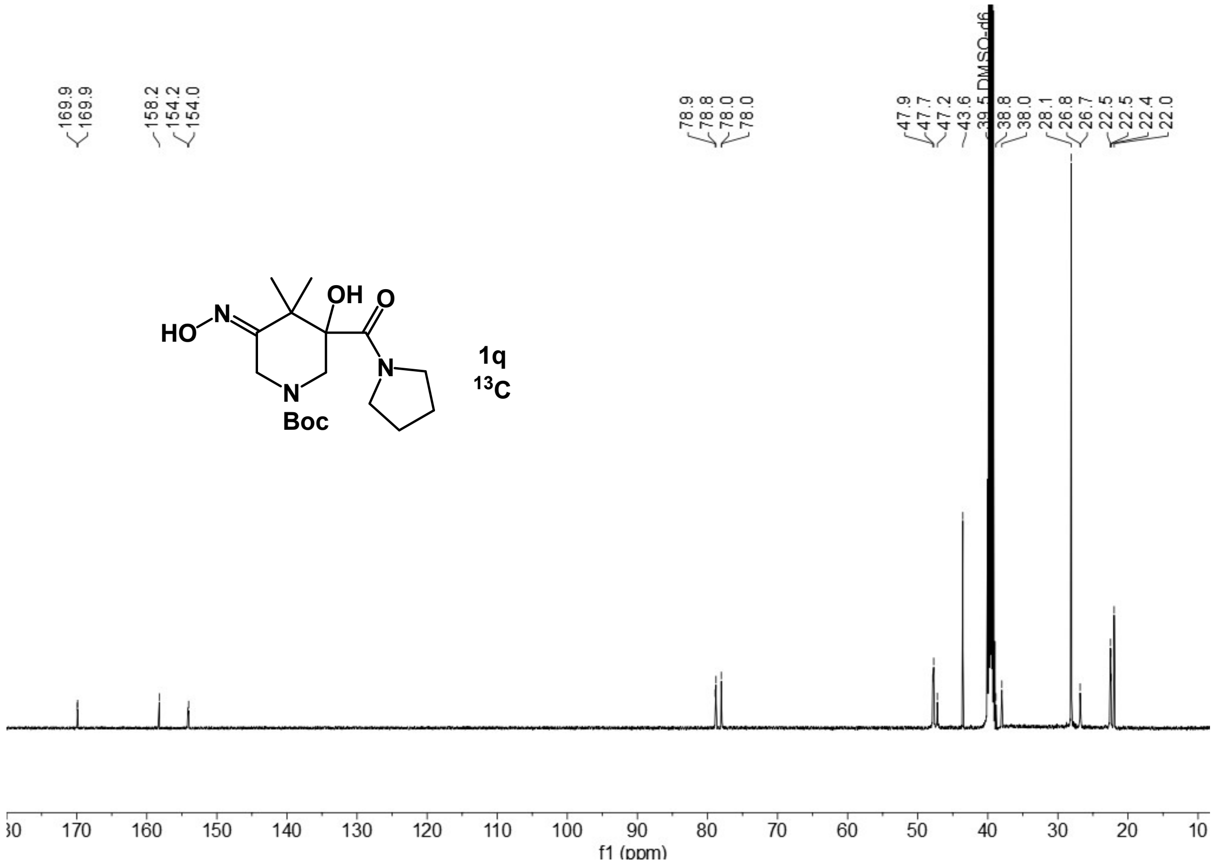
1q
13C
HO
N
OH
O
N
Boc
169.9
169.9
158.2
154.2
154.0
78.9
78.8
78.0
78.0
47.9
47.7
47.2
43.6
39.5 DMSO-d6
38.8
38.0
28.1
26.8
26.7
22.5
22.5
22.4
22.0
f1 (ppm)

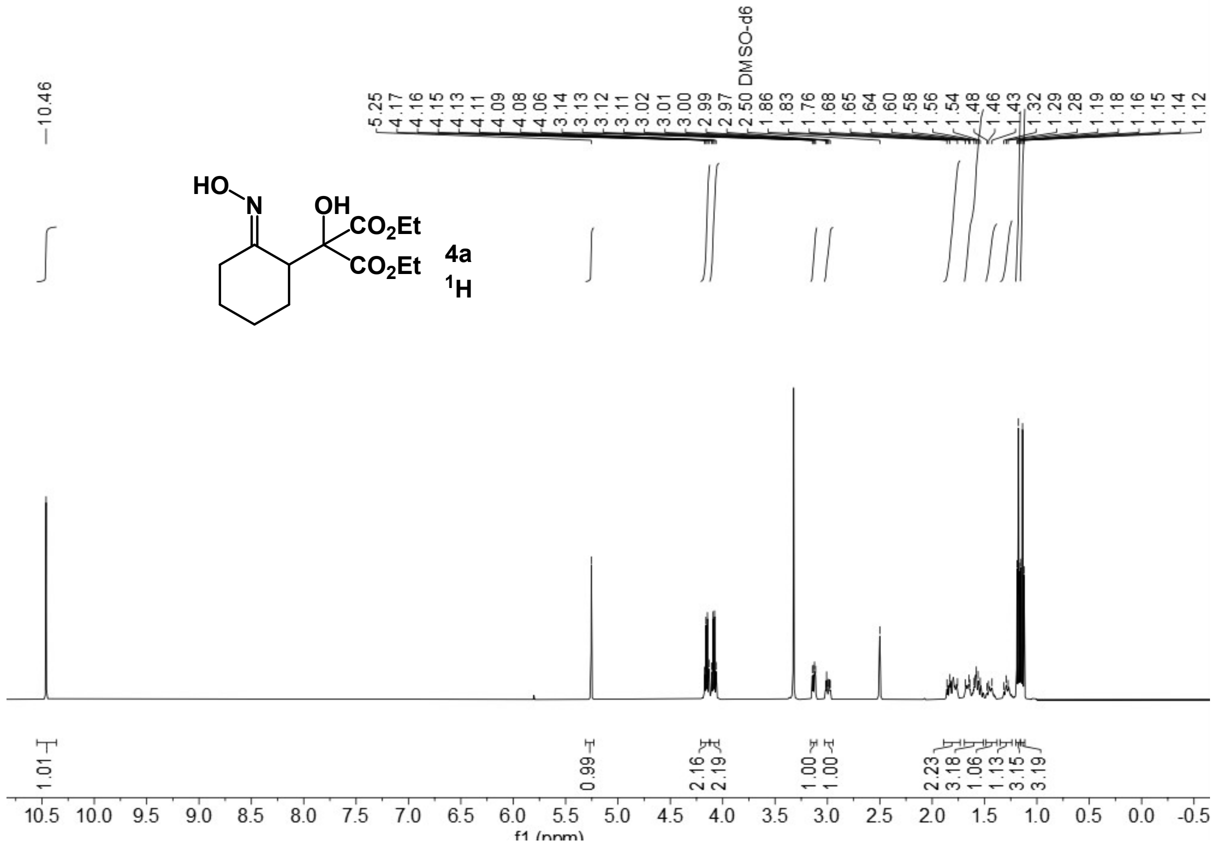

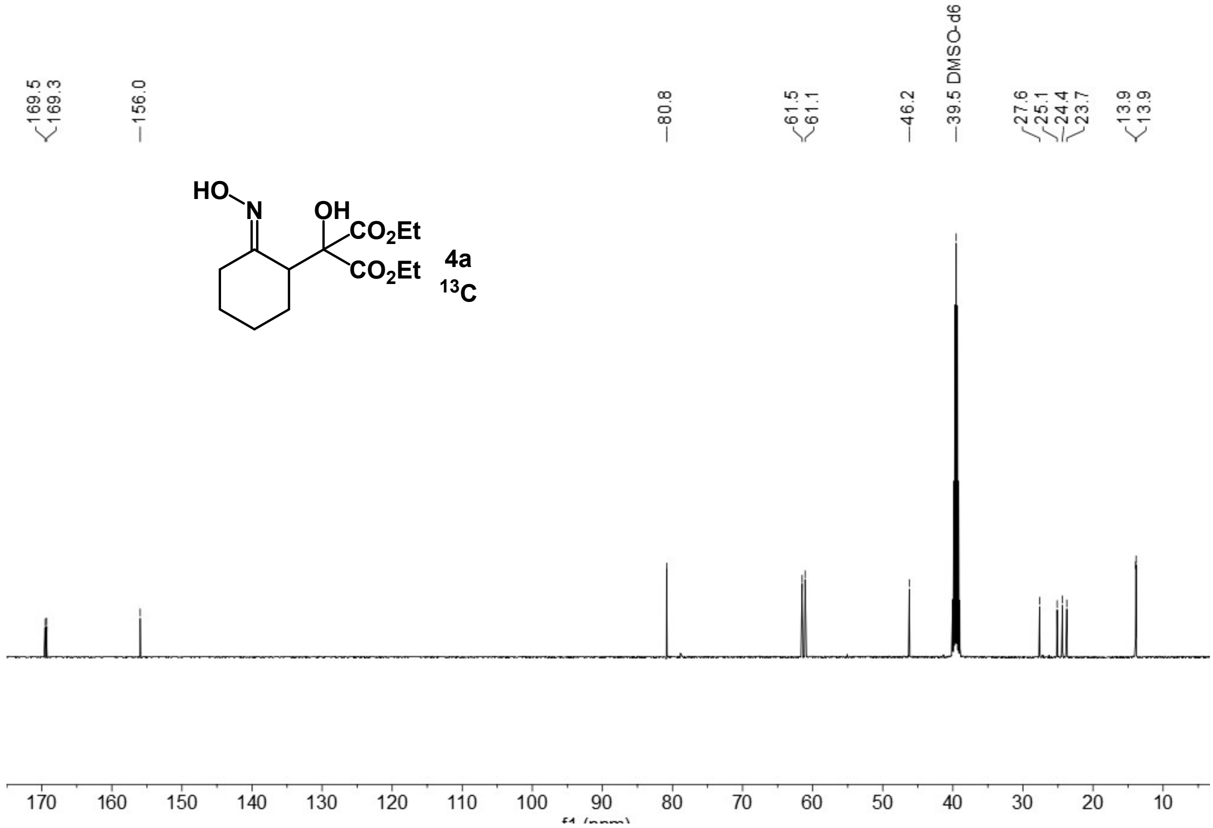

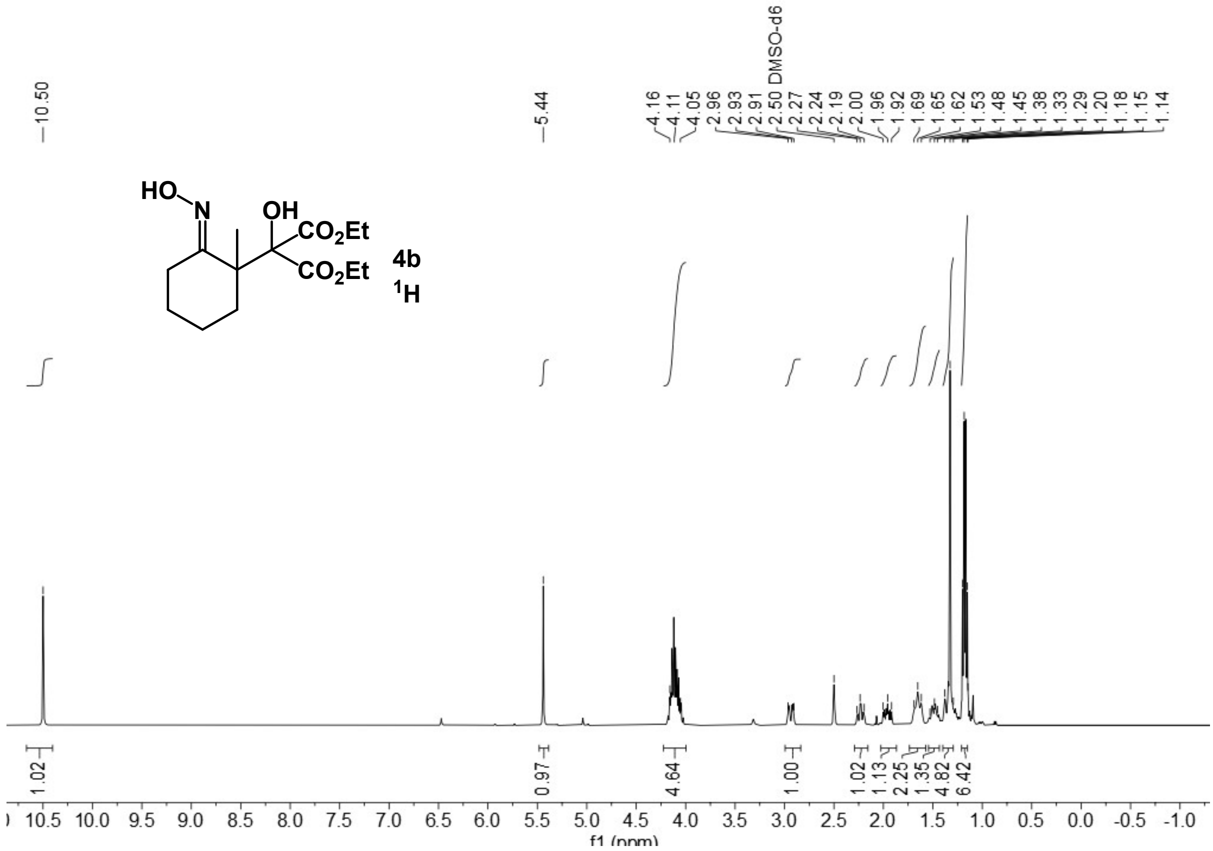
HO
N
OH
CO2Et
CO2Et
4b
1H
10.50
5.44
4.16
4.11
4.05
2.96
2.93
2.91
2.50 DMSO-d6
2.27
2.24
2.19
2.00
1.96
1.92
1.69
1.65
1.62
1.53
1.48
1.45
1.38
1.33
1.29
1.20
1.18
1.15
1.14
1.02
0.97
4.64
1.00
1.02
1.13
2.25
1.35
4.82
6.42
f1 (ppm)

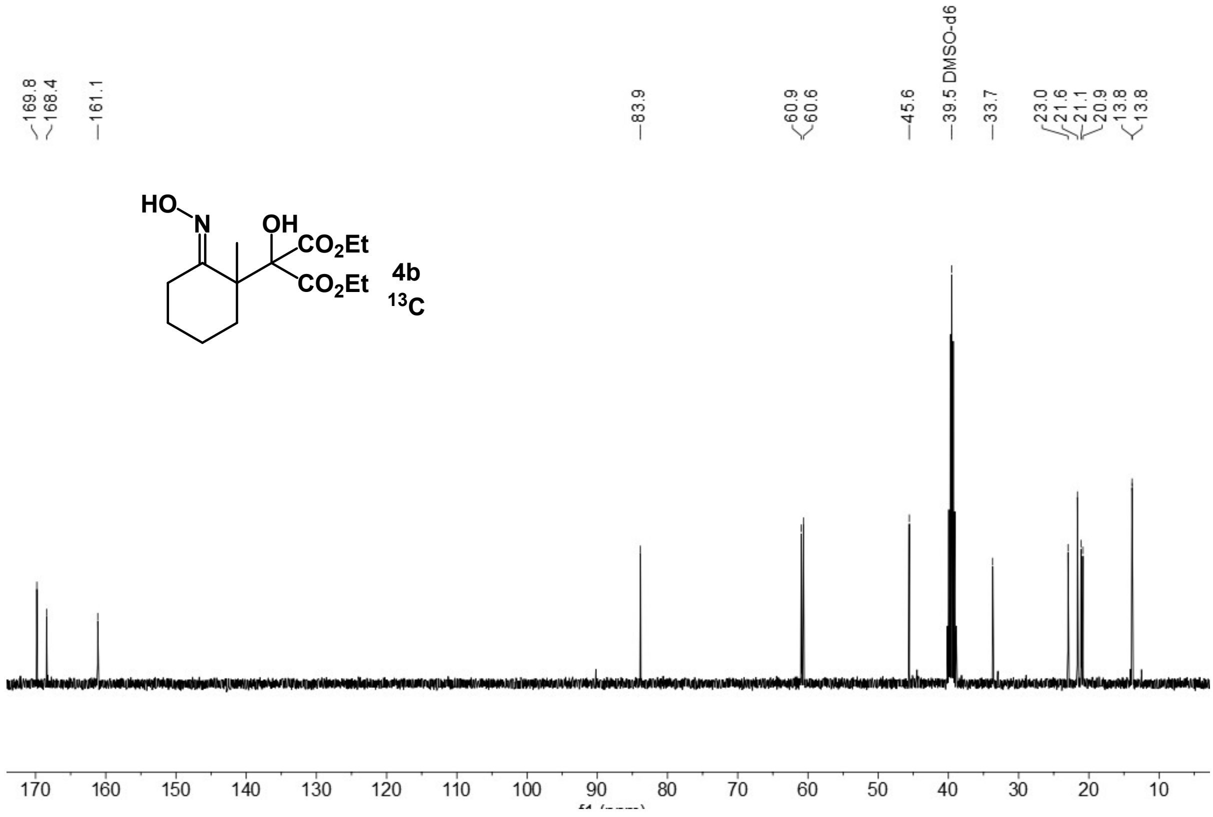
HO
N
OH
CO2Et
CO2Et
4b
13C
169.8
168.4
161.1
83.9
60.9
60.6
45.6
39.5 DMSO-d6
33.7
23.0
21.6
21.1
20.9
13.8
13.8

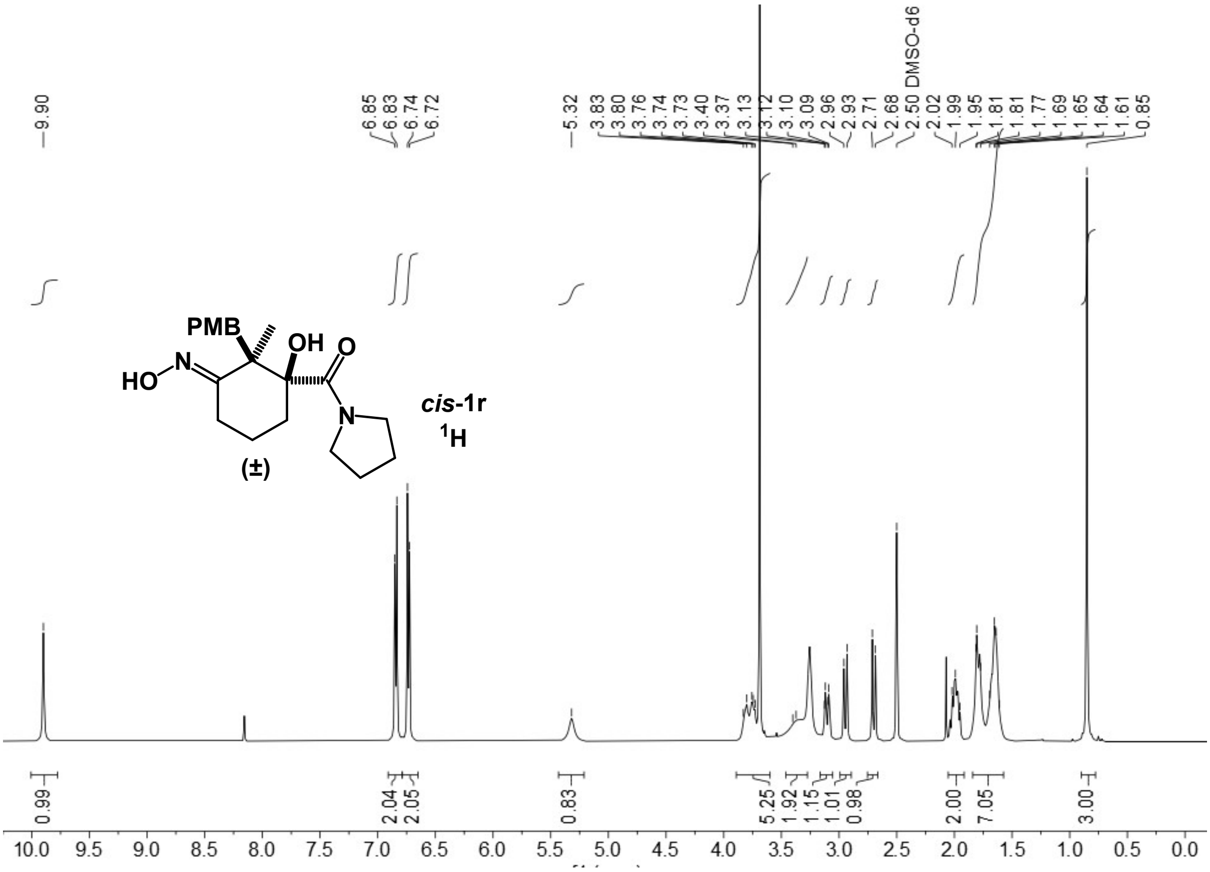
9.90
6.85
6.83
6.74
6.72
5.32
3.83
3.80
3.76
3.74
3.73
3.40
3.37
3.13
3.12
3.10
3.09
2.96
2.93
2.71
2.68
2.50 DMSO-d6
2.02
1.99
1.95
1.81
1.81
1.77
1.69
1.65
1.64
1.61
0.85
PMB
OH
O
HO
N
N
(±)
cis-1r
1H
0.99
2.04
2.05
0.83
5.25
1.92
1.15
1.01
0.98
2.00
7.05
3.00
10.0 9.5 9.0 8.5 8.0 7.5 7.0 6.5 6.0 5.5 5.0 4.5 4.0 3.5 3.0 2.5 2.0 1.5 1.0 0.5 0.0

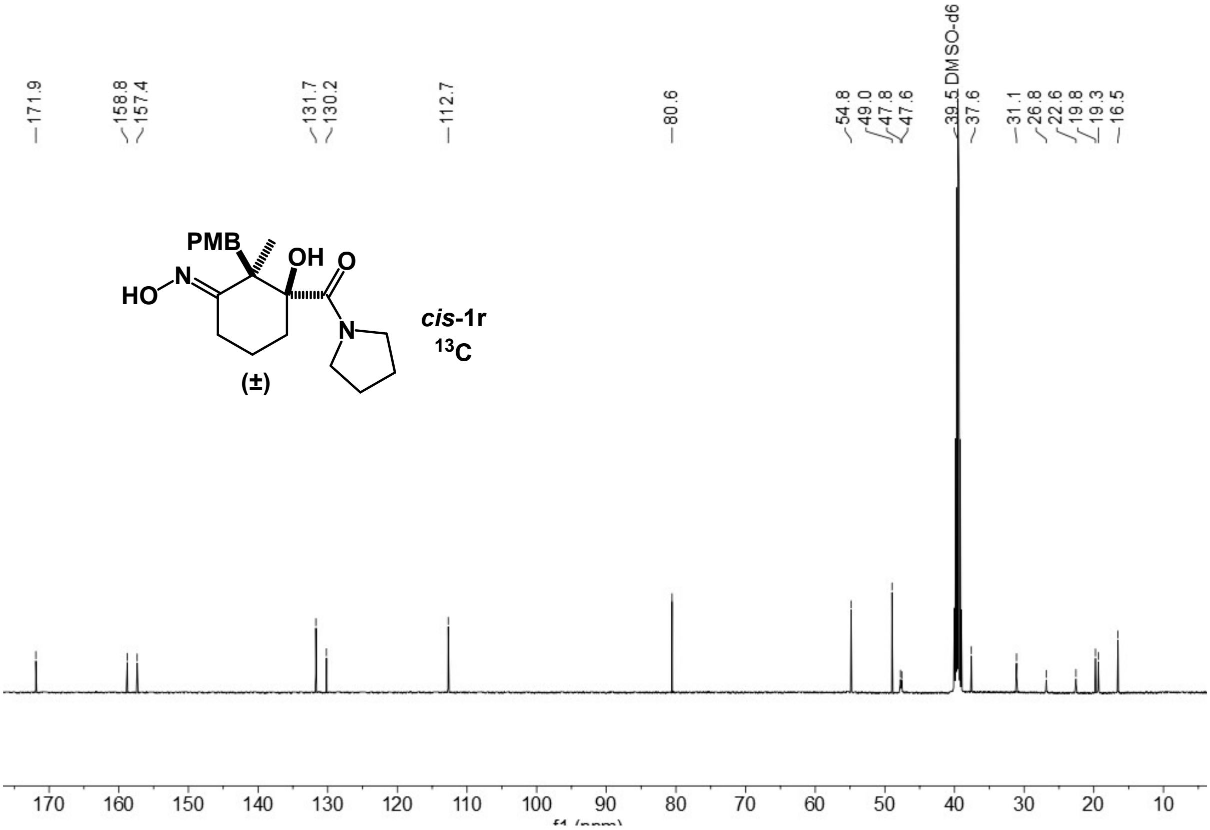
171.9
158.8
157.4
131.7
130.2
112.7
80.6
54.8
49.0
47.8
47.6
39.5 DMSO-d6
37.6
31.1
26.8
22.6
19.8
19.3
16.5
PMB
OH
O
HO
N
N
(±)
cis-1r
13C
170 160 150 140 130 120 110 100 90 80 70 60 50 40 30 20 10
f1 (ppm)

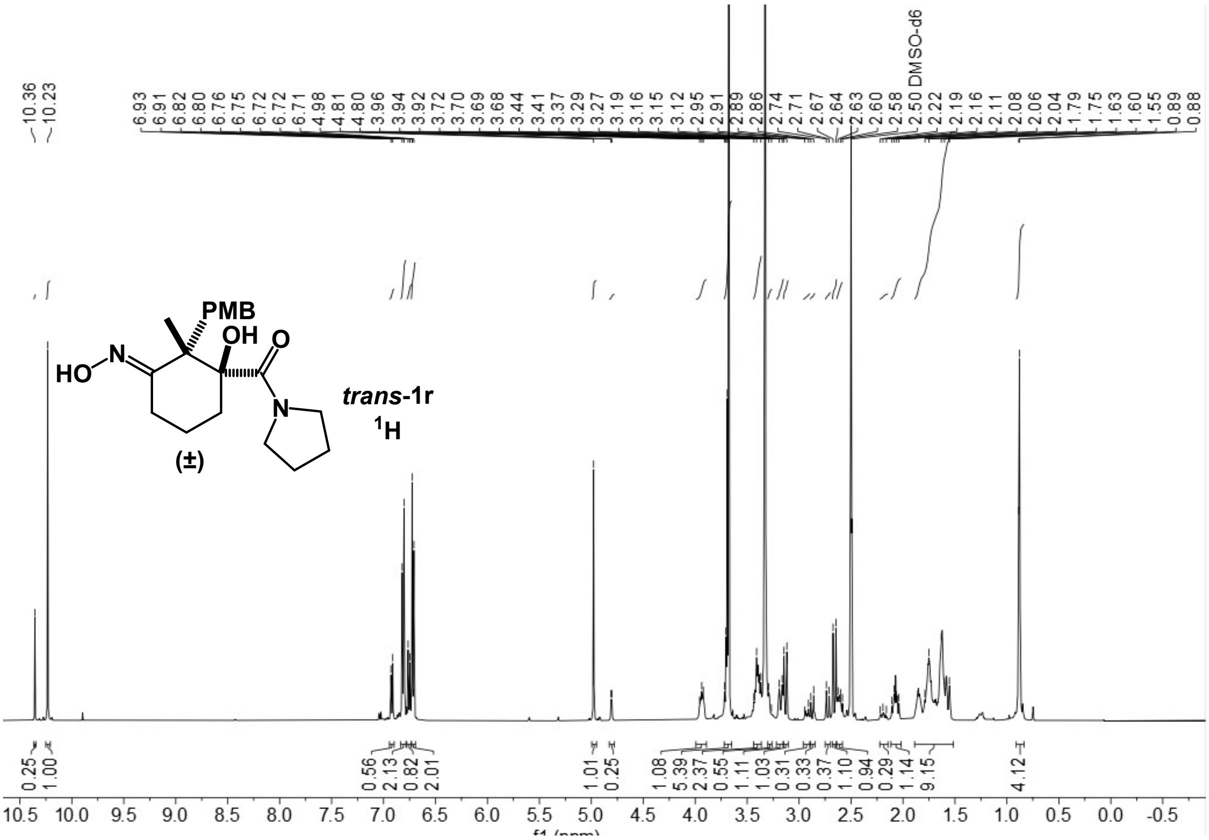
PMB
OH
O
HO
N
N
(±)
trans-1r
1H
DMSO-d6
f1 (ppm)

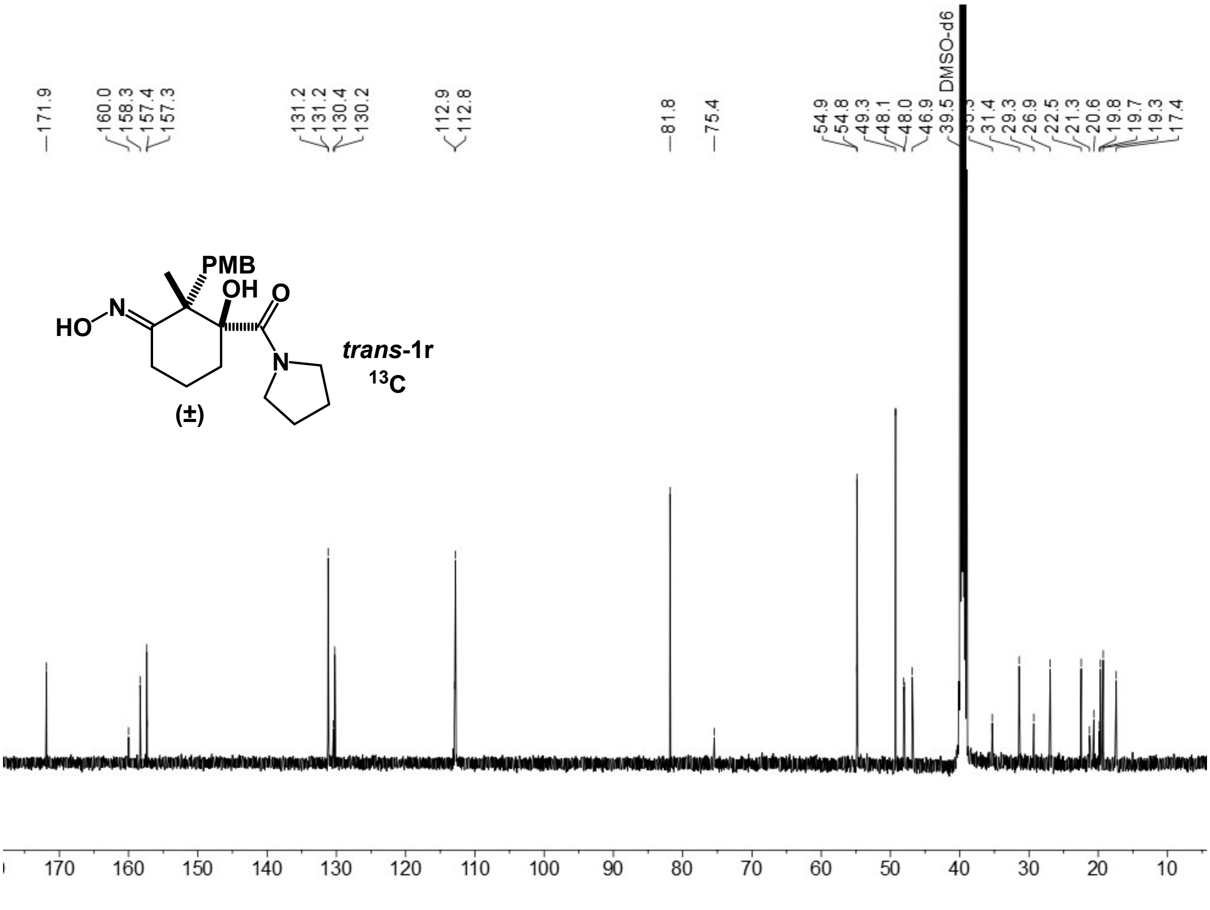
PMB
OH
O
HO
N
N
(±)
trans-1r
13C
DMSO-d6

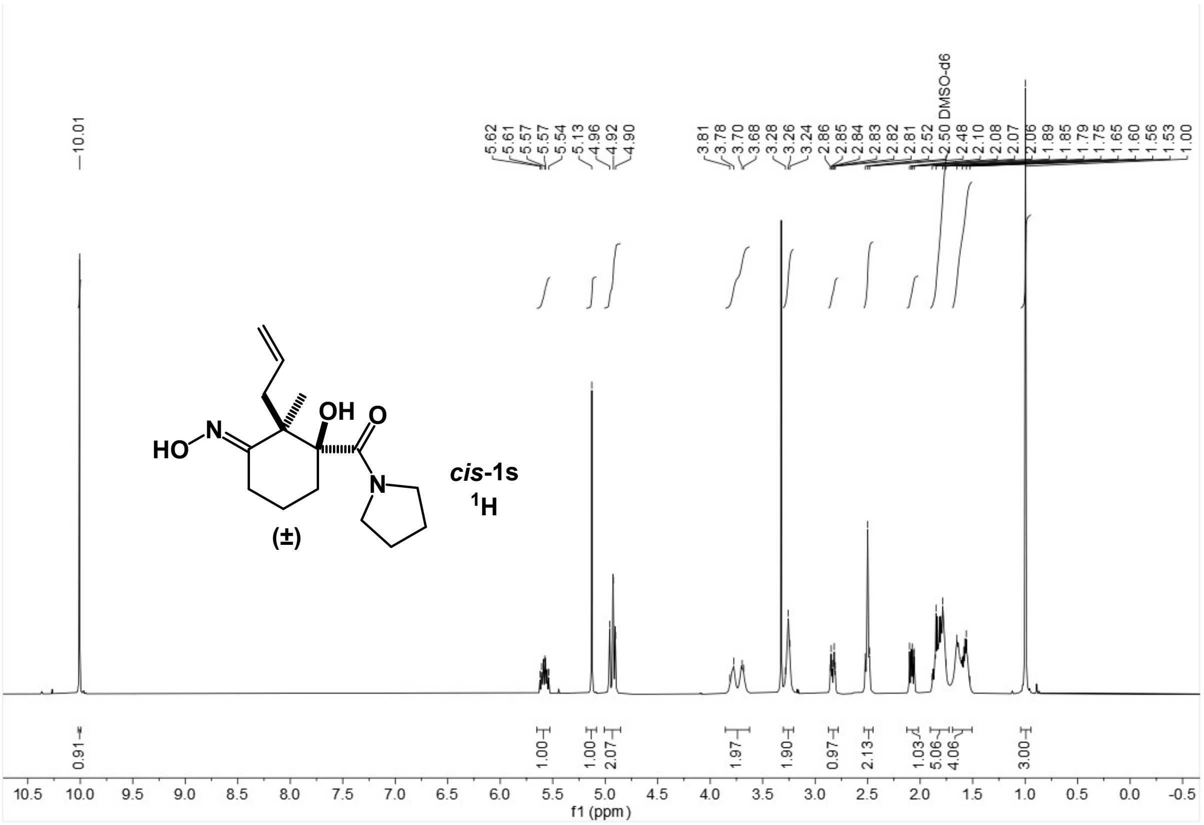

10.01
5.62 5.61 5.57 5.57 5.54 5.13 4.96 4.92 4.90
3.81 3.78 3.70 3.68 3.28 3.26 3.24 2.86 2.85 2.84 2.83 2.82 2.82 2.81 2.52 2.50 DMSO-d6 2.48 2.10 2.08 2.07 2.06 1.89 1.85 1.79 1.75 1.65 1.60 1.56 1.53 1.00
HO
N
OH
O
N
(±)
cis-1s
^{1}H
0.91 1.00 1.00 2.07 1.97 1.90 0.97 2.13 1.03 5.06 4.06 3.00
f1 (ppm)

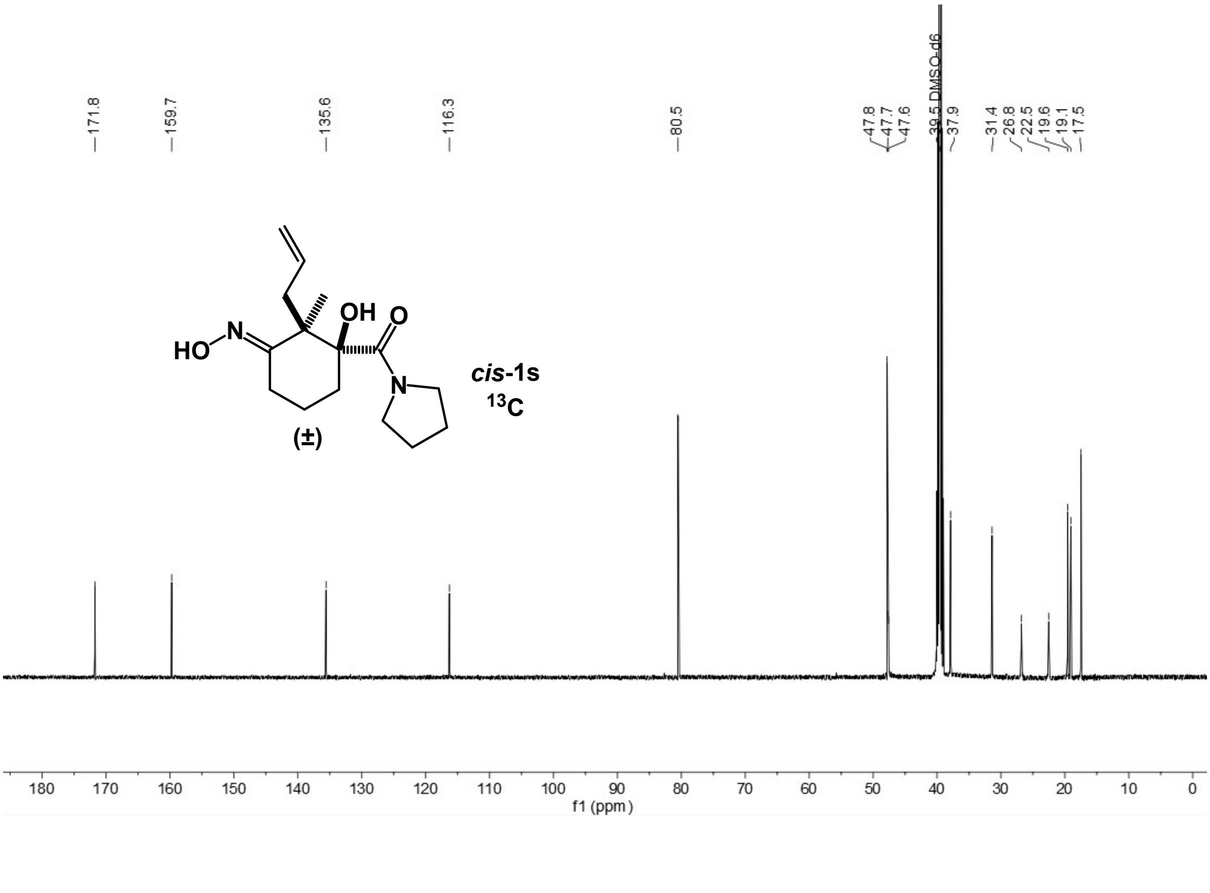

171.8
159.7
135.6
116.3
80.5
47.8 47.7 47.6
39.5 DMSO-d6
37.9
31.4 26.8 22.5 19.6 19.1 17.5
HO
N
OH
O
N
(±)
cis-1s
^{13}C
f1 (ppm)

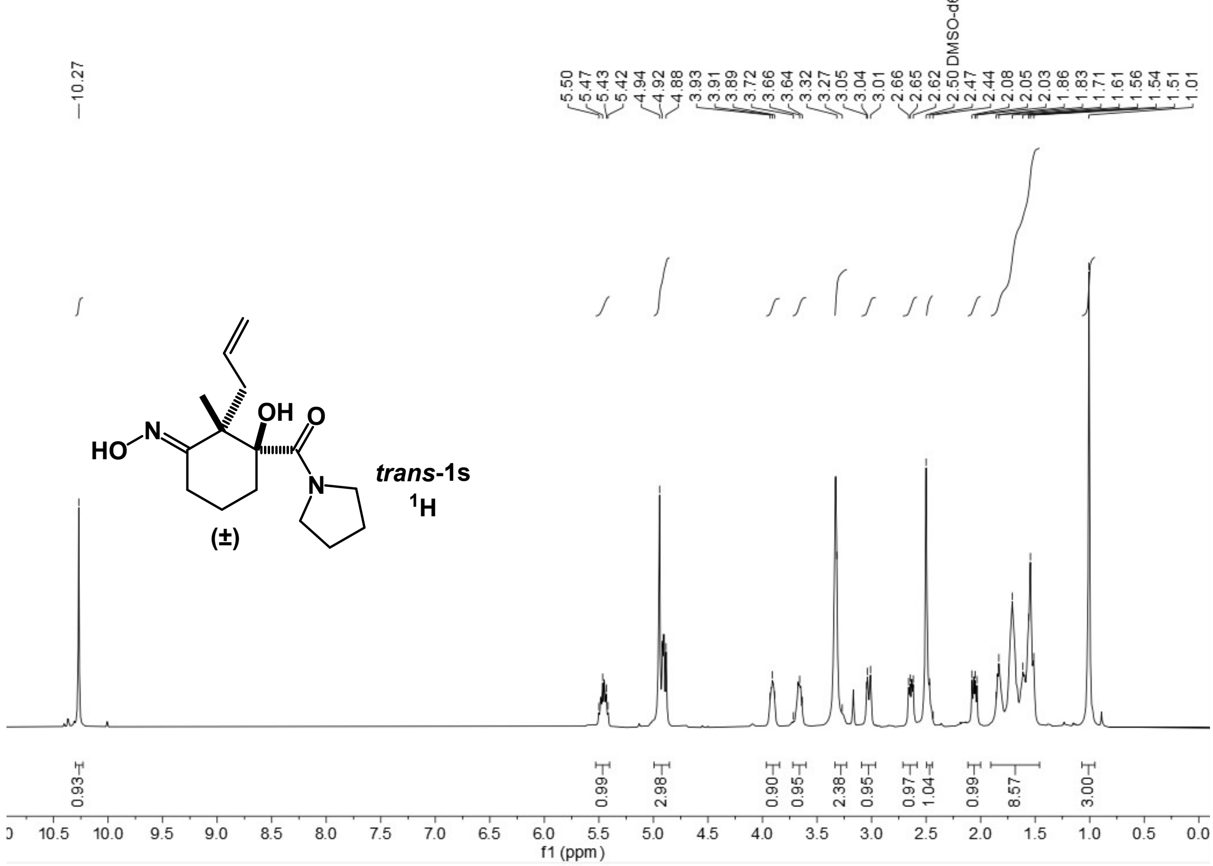

10.27
5.50
5.47
5.43
5.42
4.94
4.92
4.88
3.93
3.91
3.89
3.72
3.66
3.64
3.32
3.27
3.05
3.04
3.01
2.66
2.65
2.62
2.50 DMSO-d6
2.47
2.44
2.08
2.05
2.03
1.86
1.83
1.71
1.61
1.56
1.54
1.51
1.01
HO
N
OH
O
N
(±)
trans-1s
1H
0.93
0.99
2.98
0.90
0.95
2.38
0.95
0.97
1.04
0.99
8.57
3.00
f1 (ppm)

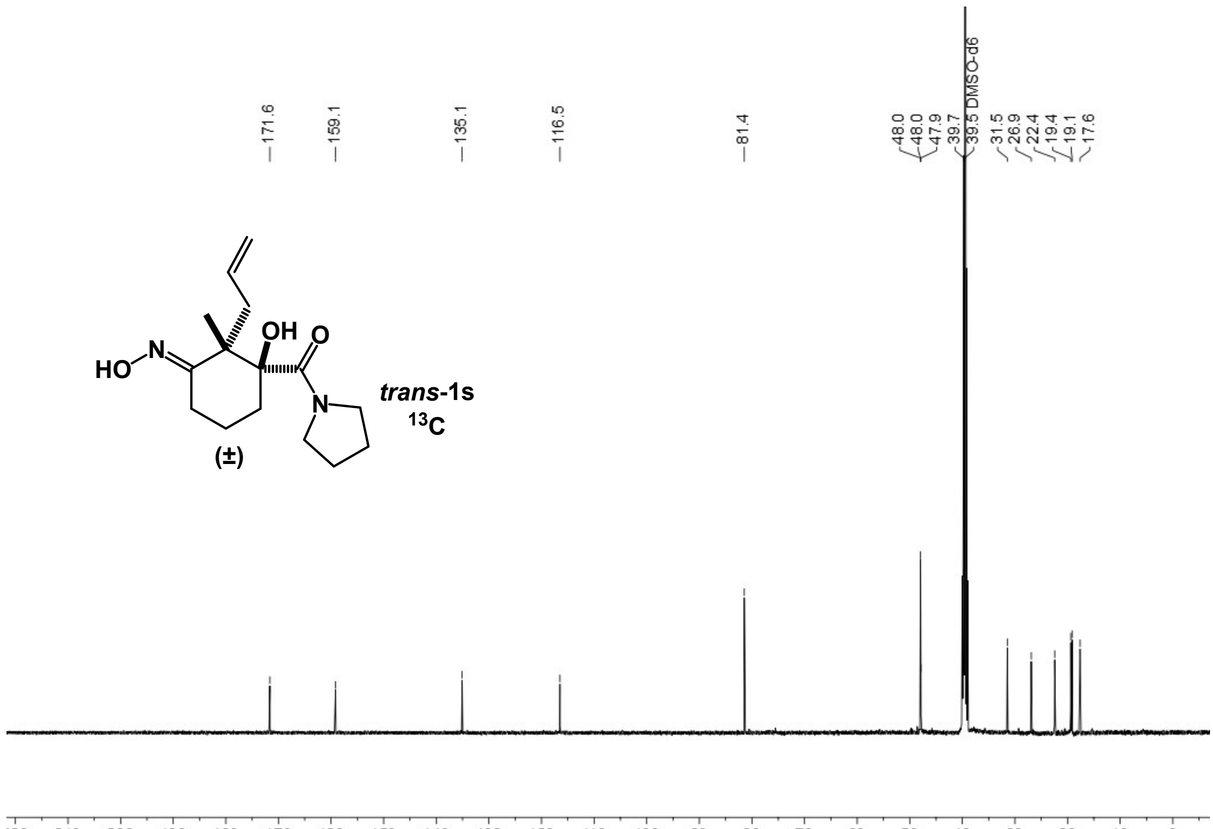

171.6
159.1
135.1
116.5
81.4
48.0
48.0
47.9
39.7
39.5 DMSO-d6
31.5
26.9
22.4
19.4
19.1
17.6
HO
N
OH
O
N
(±)
trans-1s
13C
f1 (ppm)

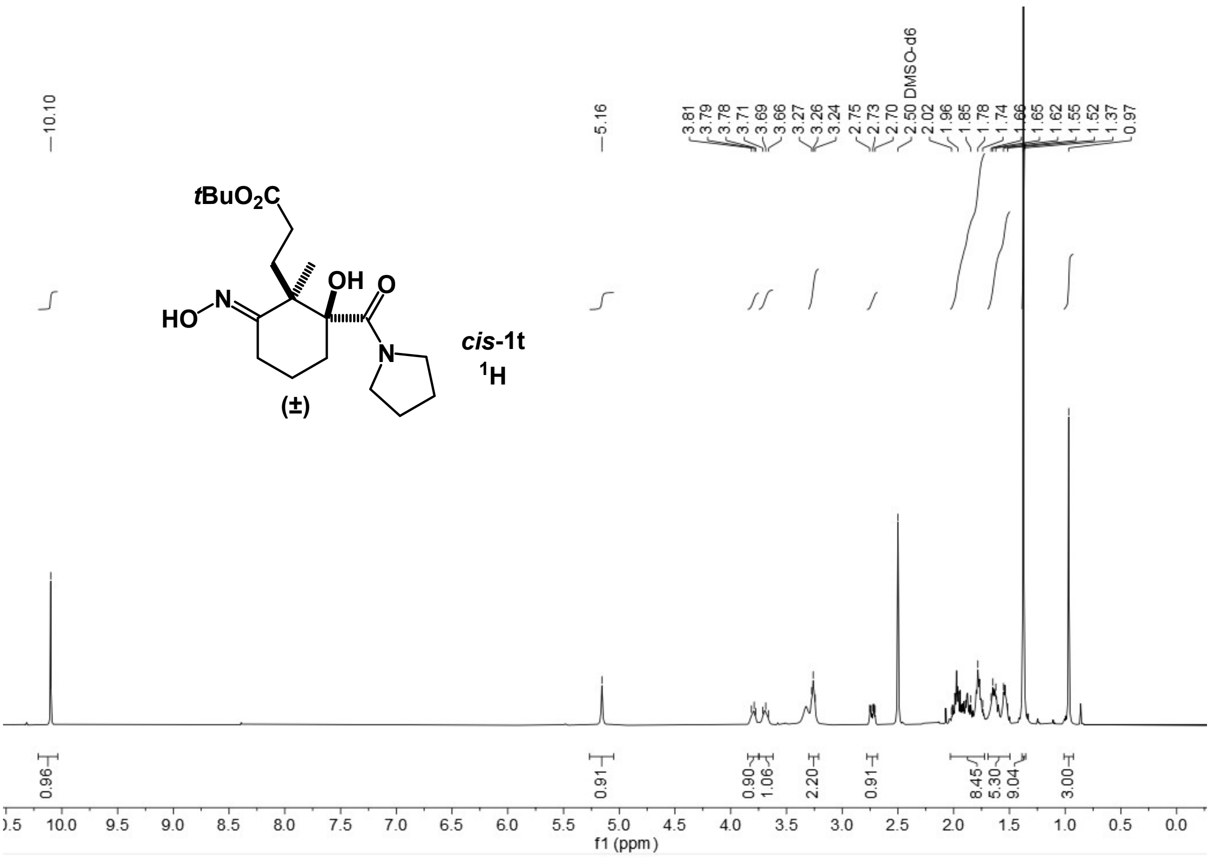
10.10
5.16
3.81
3.79
3.78
3.71
3.69
3.66
3.27
3.26
3.24
2.75
2.73
2.70
2.50 DMSO-d6
2.02
1.96
1.85
1.78
1.74
1.66
1.65
1.62
1.55
1.52
1.37
0.97
tBuO2C
OH
O
HO
N
N
(±)
cis-1t
1H
0.96
0.91
0.90
1.06
2.20
0.91
8.45
5.30
9.04
3.00
f1 (ppm)

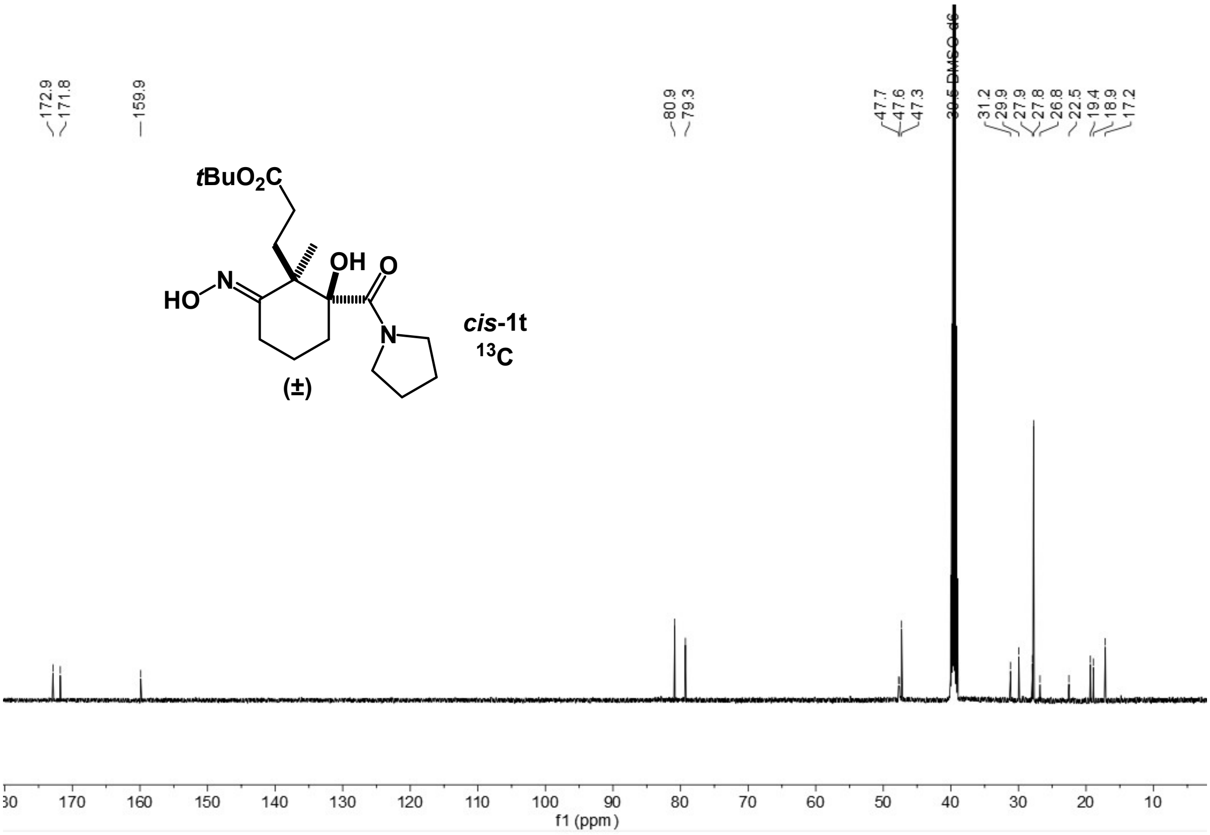
172.9
171.8
159.9
80.9
79.3
47.7
47.6
47.3
39.5 DMSO-d6
31.2
29.9
27.9
27.8
26.8
22.5
19.4
18.9
17.2
tBuO2C
OH
O
HO
N
N
(±)
cis-1t
13C
f1 (ppm)

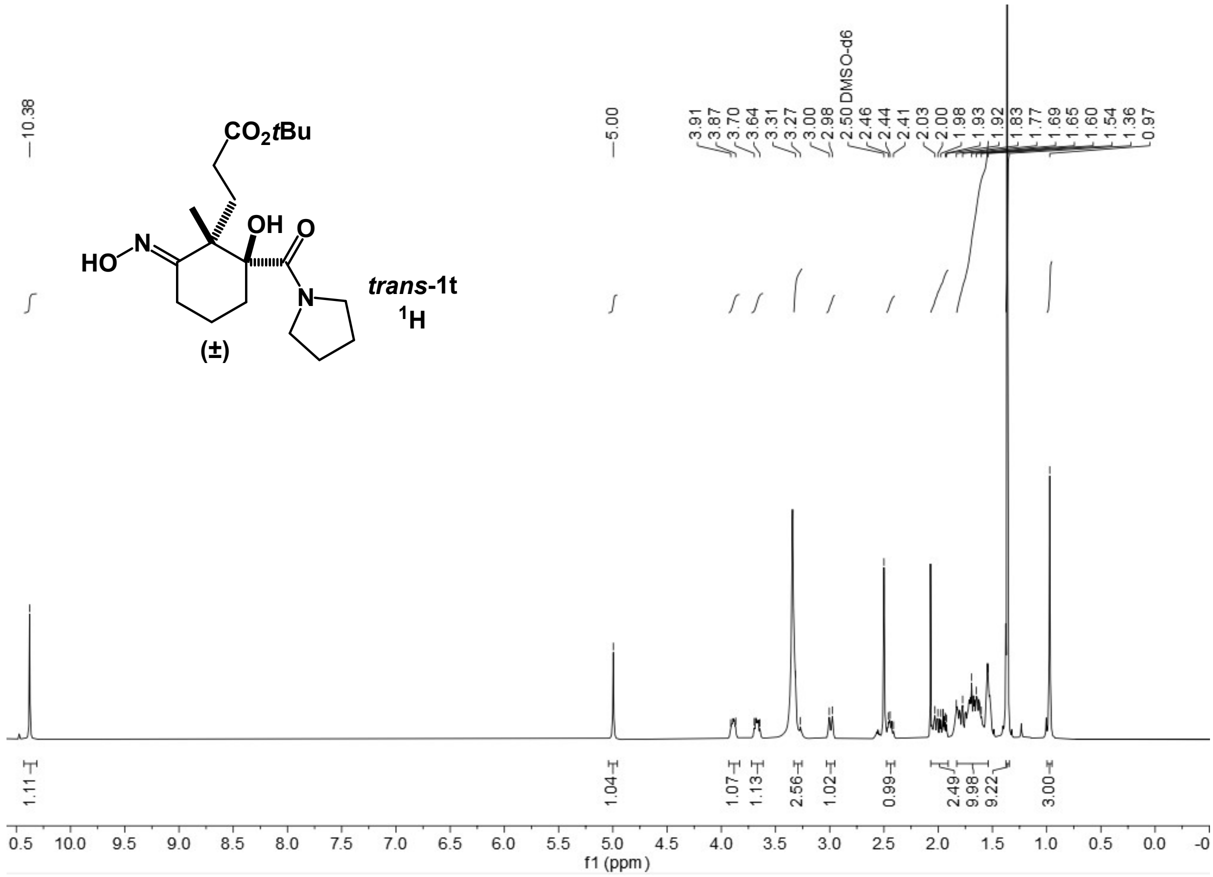

10.38
5.00
3.91
3.87
3.70
3.64
3.31
3.27
3.00
2.98
2.50 DMSO-d6
2.46
2.44
2.41
2.03
2.00
1.98
1.93
1.92
1.83
1.77
1.69
1.65
1.60
1.54
1.36
0.97
CO2tBu
OH
O
HO
N
N
(±)
trans-1t
1H
1.11
1.04
1.07
1.13
2.56
1.02
0.99
2.49
9.98
9.22
3.00
0.5 10.0 9.5 9.0 8.5 8.0 7.5 7.0 6.5 6.0 5.5 5.0 4.5 4.0 3.5 3.0 2.5 2.0 1.5 1.0 0.5 0.0 -0
f1 (ppm)

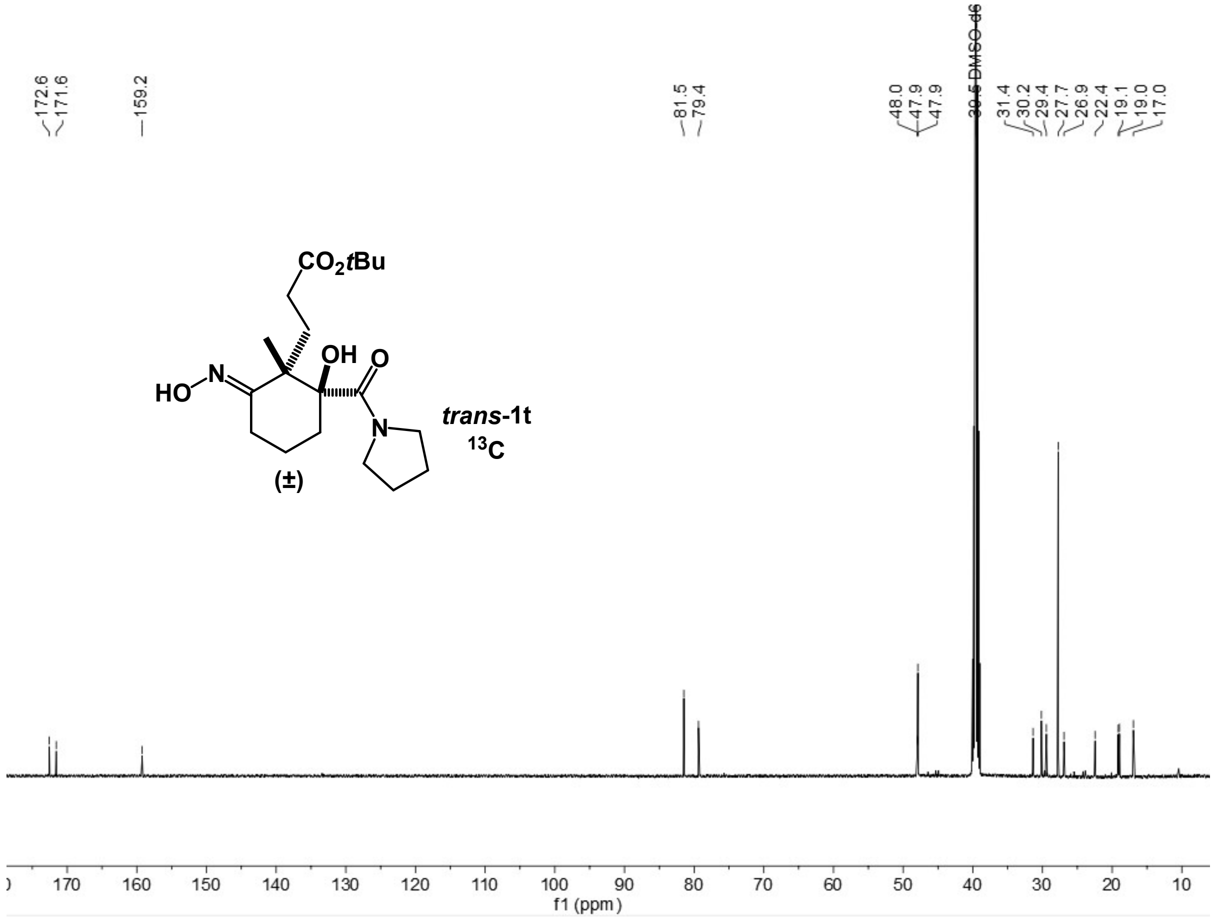

172.6
171.6
159.2
81.5
79.4
48.0
47.9
47.9
39.5 DMSO-d6
31.4
30.2
29.4
27.7
26.9
22.4
19.1
19.0
17.0
CO2tBu
OH
O
HO
N
N
(±)
trans-1t
13C
170 160 150 140 130 120 110 100 90 80 70 60 50 40 30 20 10
f1 (ppm)

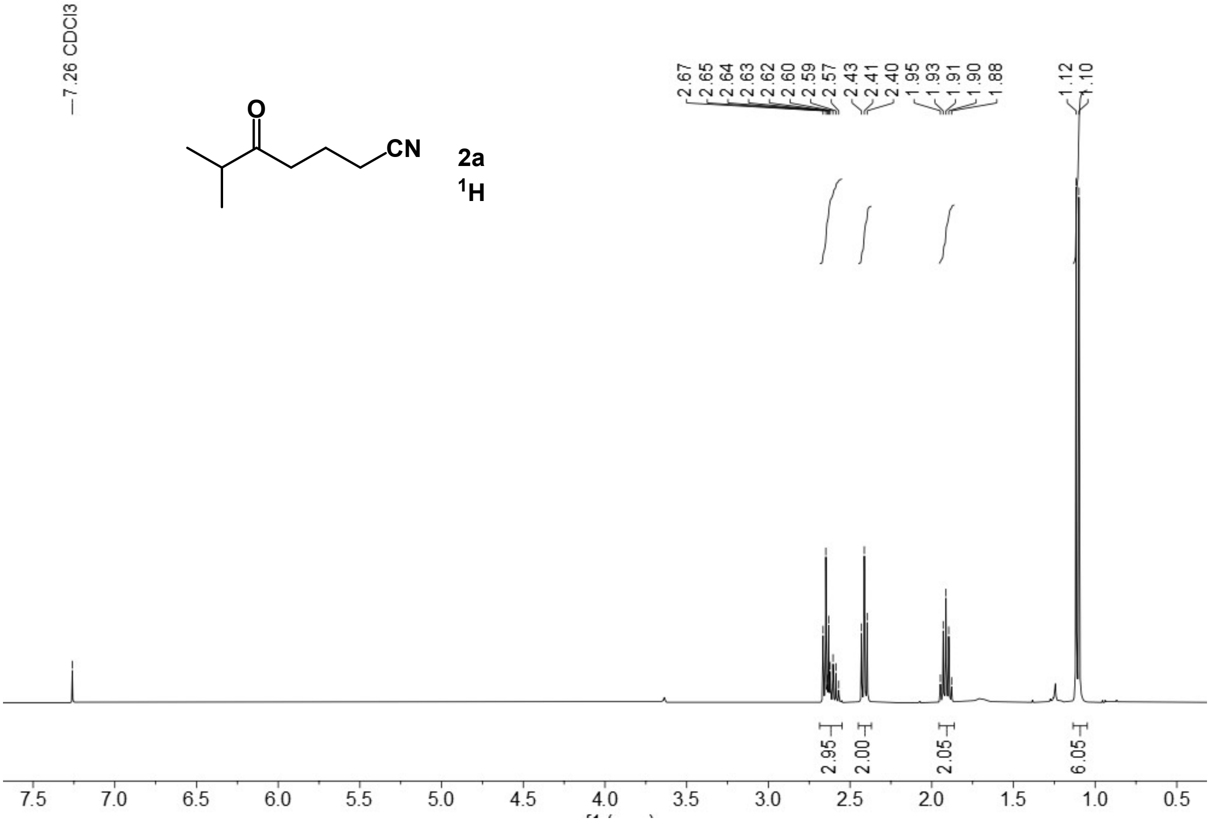
7.26 CDCl3
O
CN
2a
1H
2.67
2.65
2.64
2.63
2.62
2.60
2.59
2.57
2.43
2.41
2.40
1.95
1.93
1.91
1.90
1.88
1.12
1.10
2.95
2.00
2.05
6.05
7.5 7.0 6.5 6.0 5.5 5.0 4.5 4.0 3.5 3.0 2.5 2.0 1.5 1.0 0.5

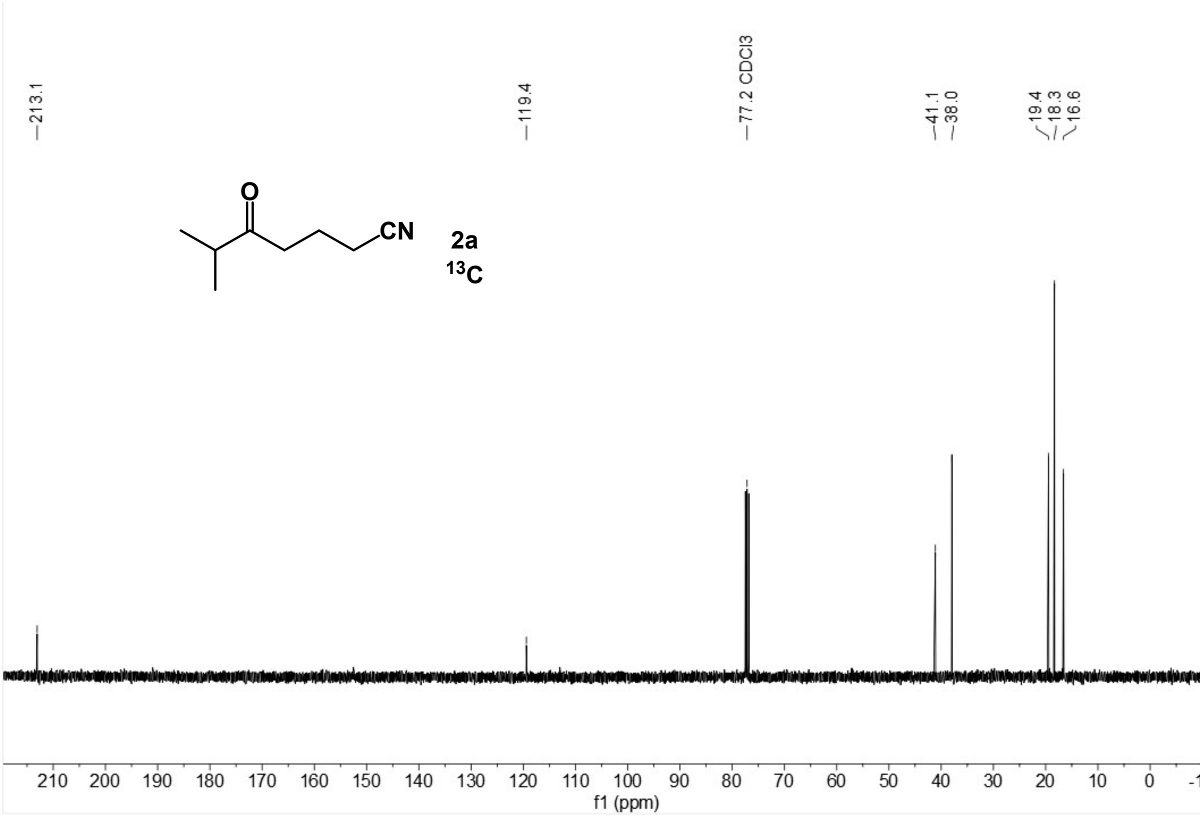
213.1
119.4
77.2 CDCl3
41.1
38.0
19.4
18.3
16.6
O
CN
2a
13C
210 200 190 180 170 160 150 140 130 120 110 100 90 80 70 60 50 40 30 20 10 0
f1 (ppm)

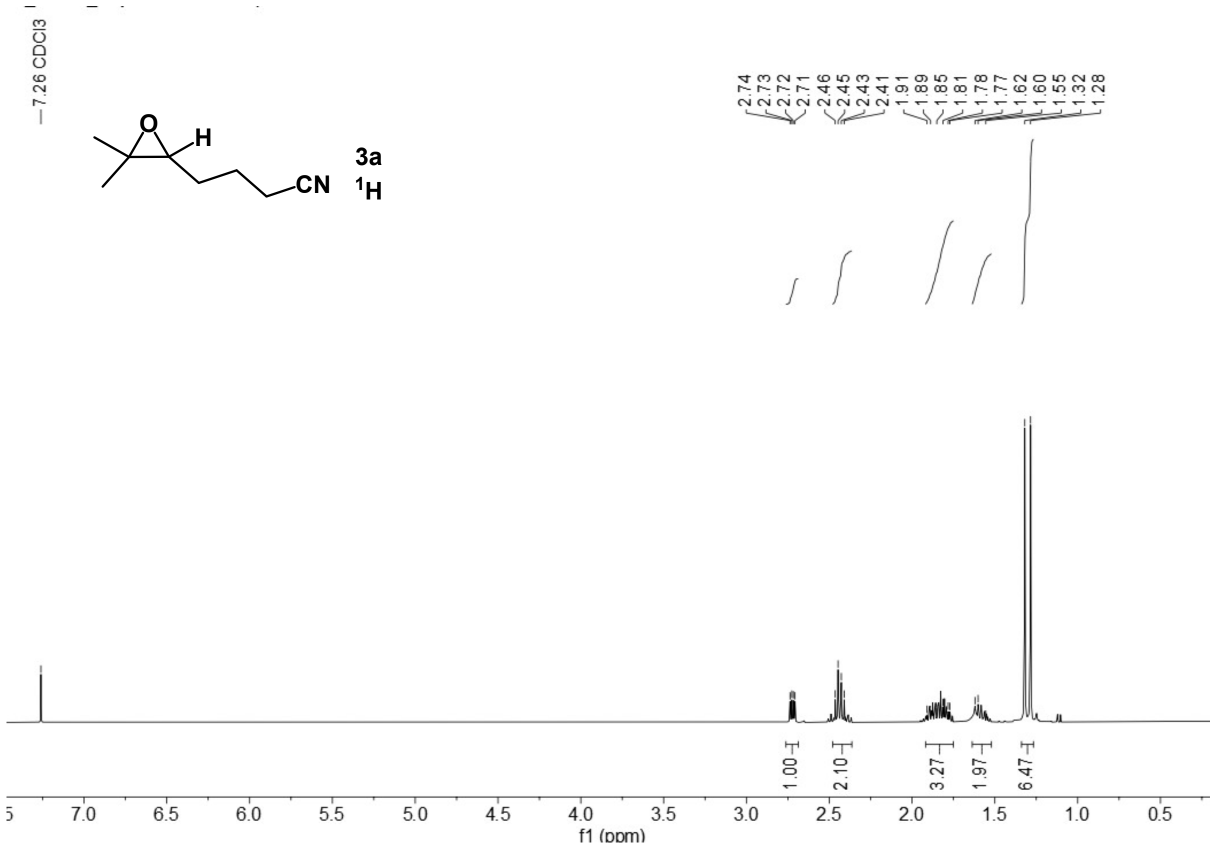
7.26 CDCl3
O
H
CN
3a
¹H
2.74 2.73 2.72 2.71 2.46 2.45 2.43 2.41 1.91 1.89 1.85 1.81 1.78 1.77 1.62 1.60 1.55 1.32 1.28
1.00
2.10
3.27
1.97
6.47
7.0 6.5 6.0 5.5 5.0 4.5 4.0 3.5 3.0 2.5 2.0 1.5 1.0 0.5
f1 (ppm)

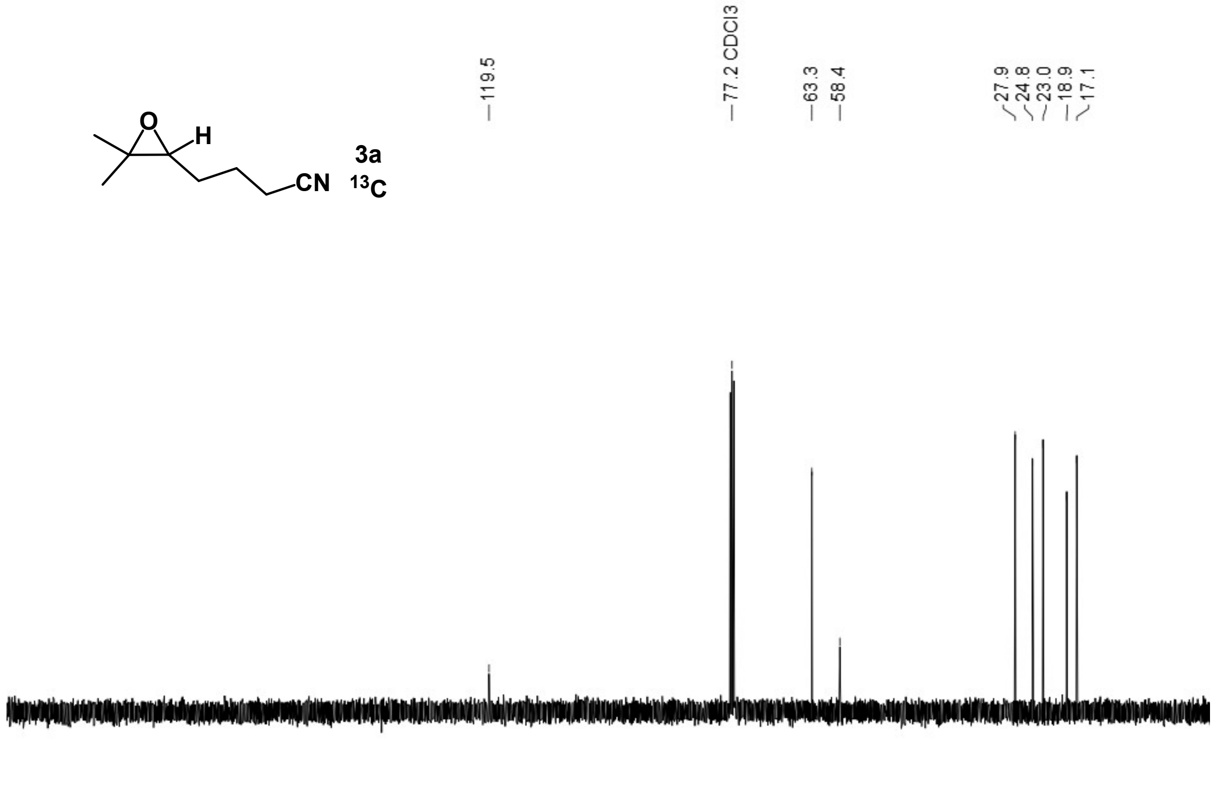
119.5
77.2 CDCl3
63.3
58.4
27.9 24.8 23.0 18.9 17.1
O
H
CN
3a
¹³C
200 190 180 170 160 150 140 130 120 110 100 90 80 70 60 50 40 30 20 10 0
f1 (ppm)

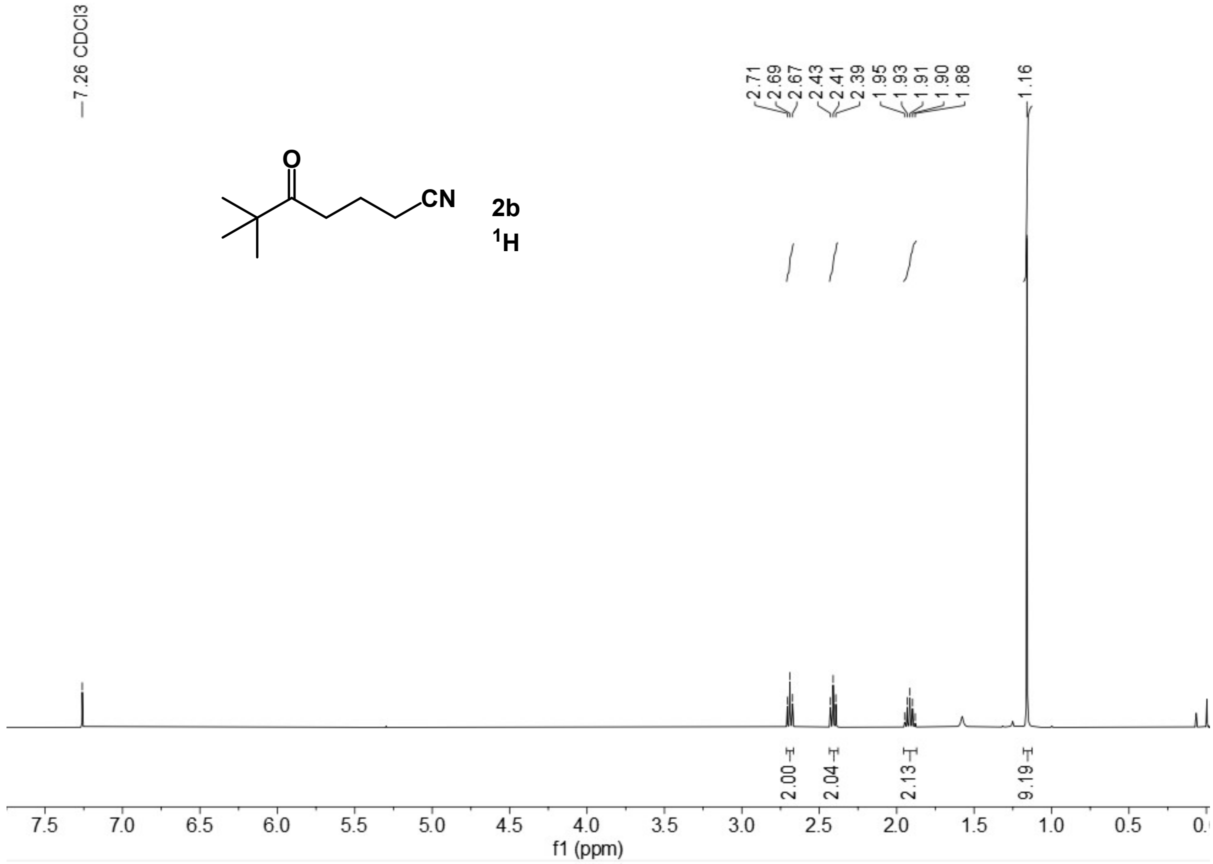

7.26 CDCl3
2.71
2.69
2.67
2.43
2.41
2.39
1.95
1.93
1.91
1.90
1.88
1.16
O
CN
2b
1H
2.00
2.04
2.13
9.19
f1 (ppm)

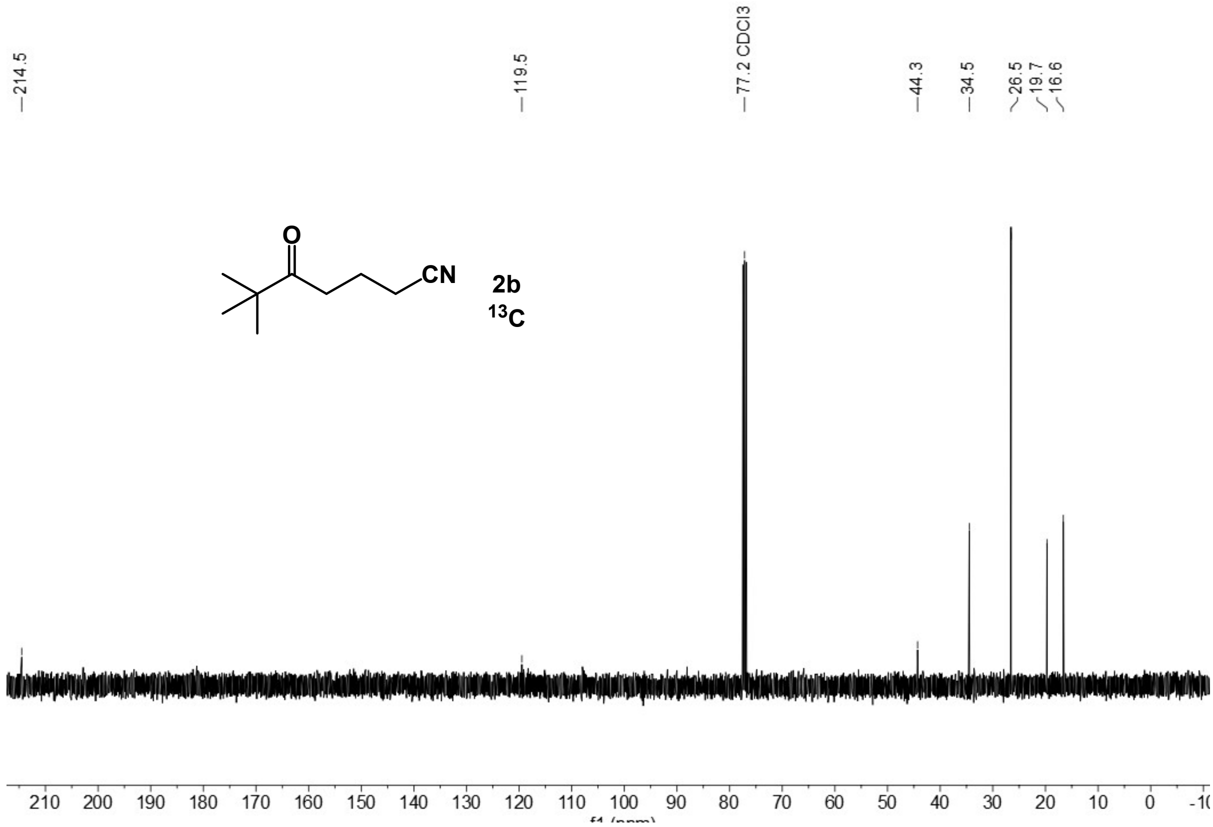

214.5
119.5
77.2 CDCl3
44.3
34.5
26.5
19.7
16.6
O
CN
2b
13C

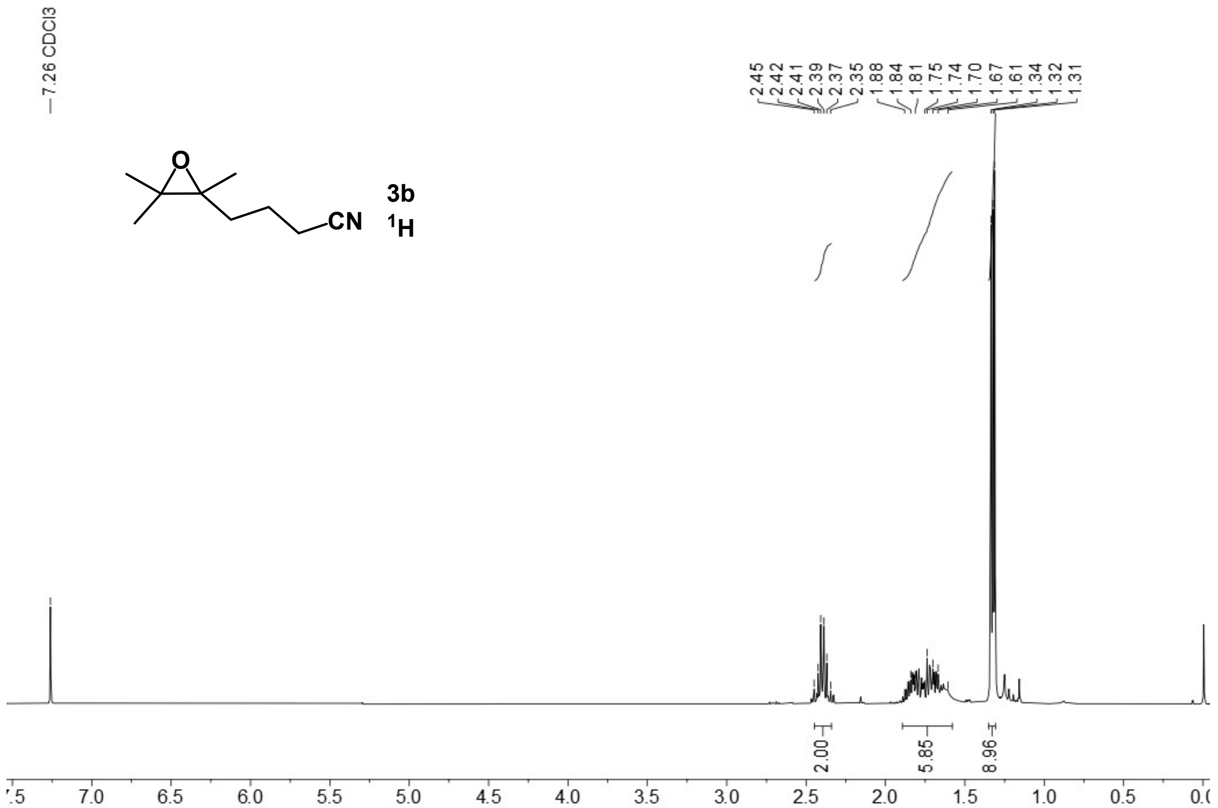
3b
1H
7.26 CDCl3
2.45
2.42
2.41
2.39
2.37
2.35
1.88
1.84
1.81
1.75
1.74
1.70
1.67
1.61
1.34
1.32
1.31
2.00
5.85
8.96

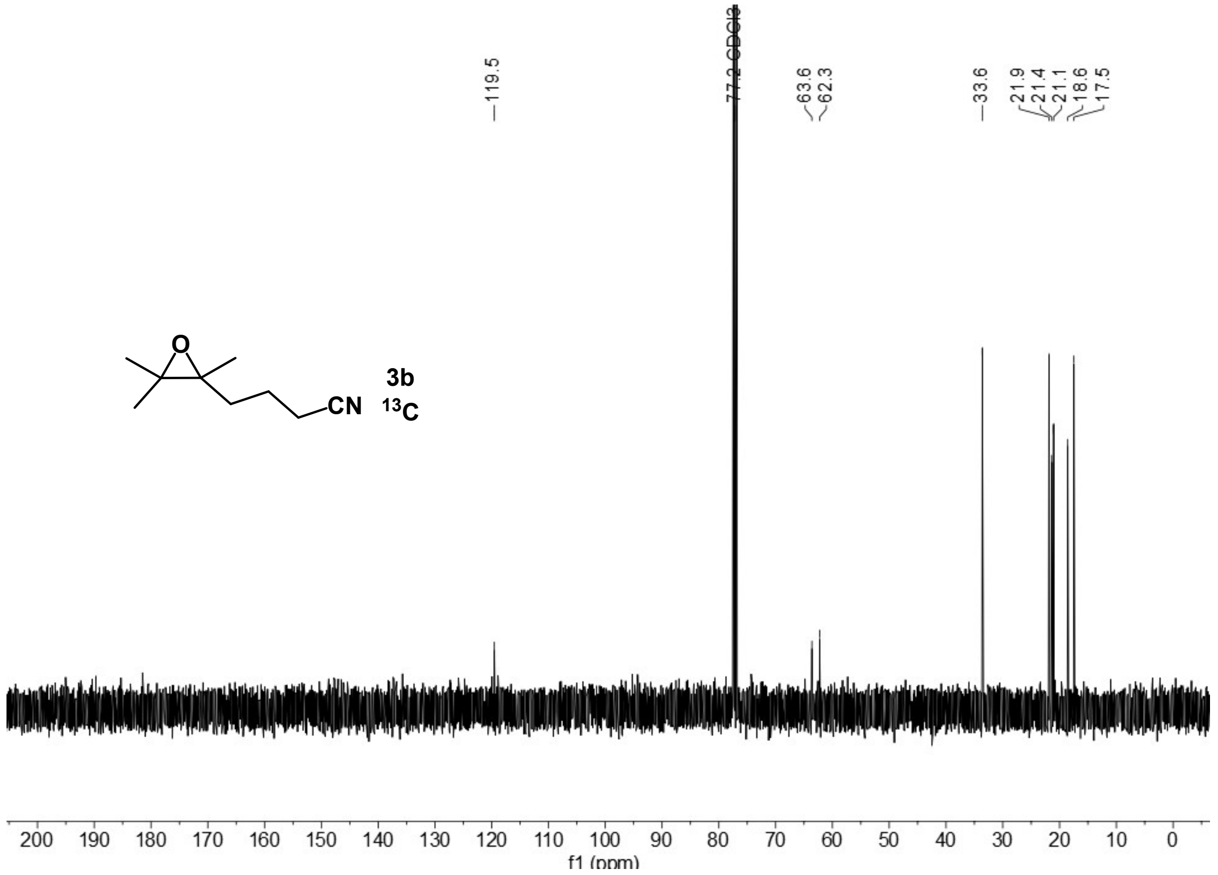
3b
13C
119.5
77.2 CDCl3
63.6
62.3
33.6
21.9
21.4
21.1
18.6
17.5
f1 (ppm)

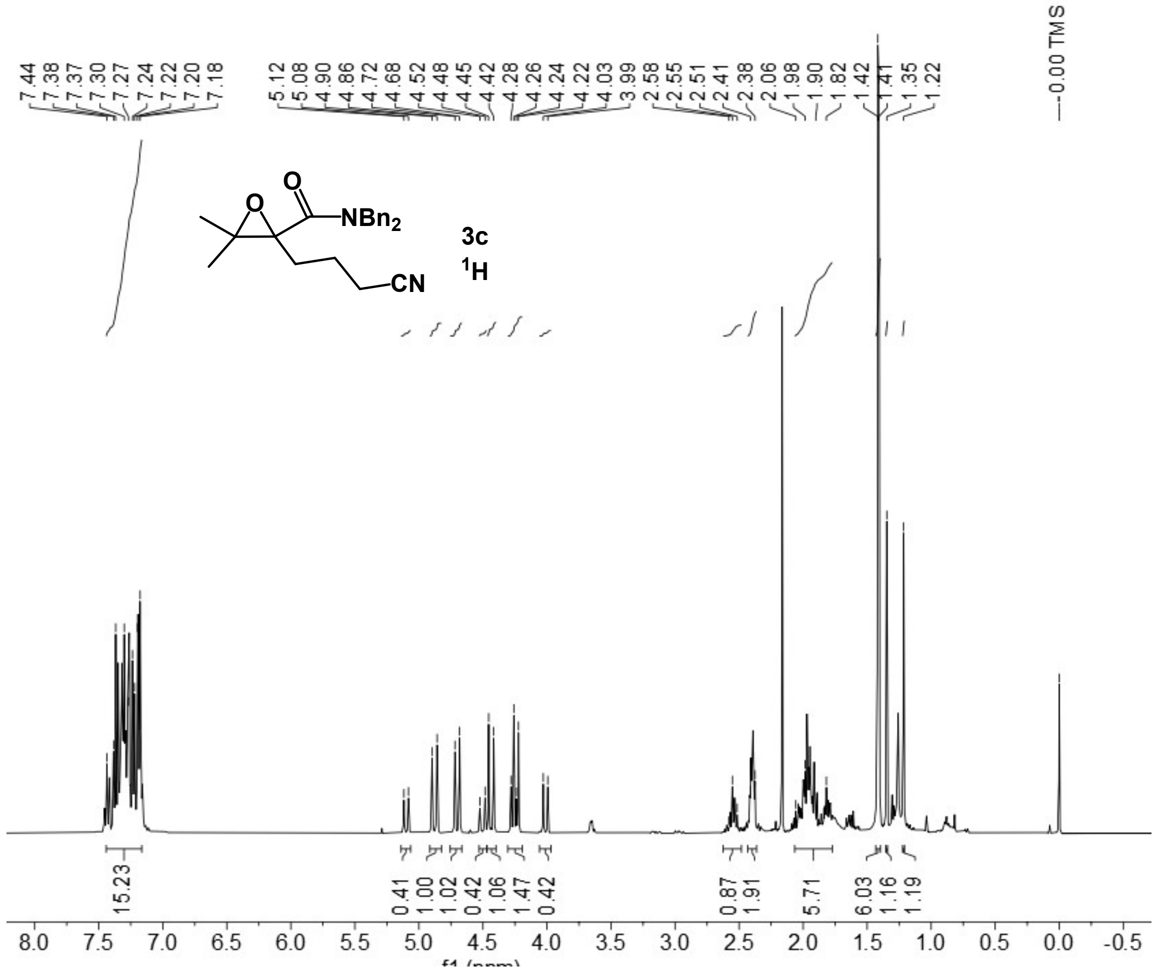
7.44 7.38 7.37 7.30 7.27 7.24 7.22 7.20 7.18
5.12 5.08 4.90 4.86 4.72 4.68 4.52 4.48 4.45 4.42 4.28 4.26 4.24 4.22 4.03 3.99
2.58 2.55 2.51 2.41 2.38 2.06 1.98 1.90 1.82 1.42 1.41 1.35 1.22
0.00 TMS
O
NBn2
CN
3c
1H
15.23
0.41 1.00 1.02 0.42 1.06 1.47 0.42
0.87 1.91 5.71 6.03 1.16 1.19
f1 (ppm)

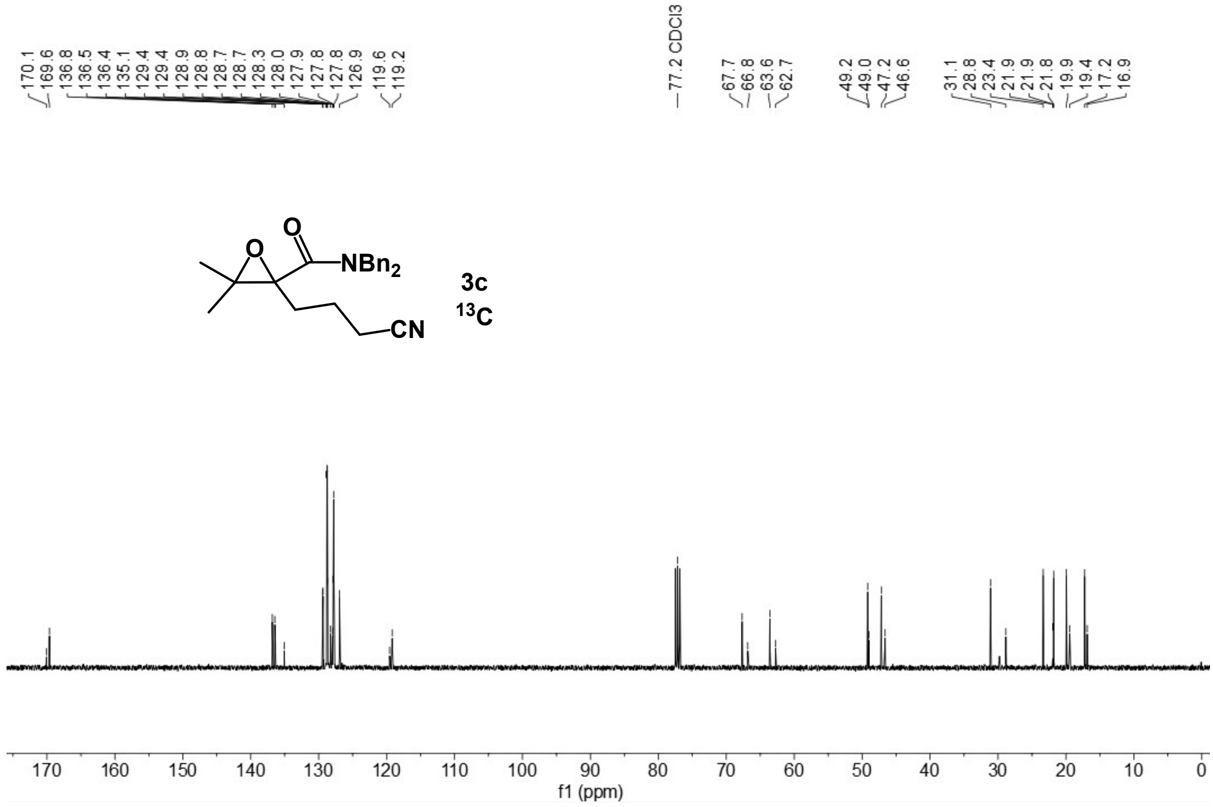
170.1 169.6 136.8 136.5 136.4 135.1 129.4 128.9 128.8 128.7 128.3 128.0 127.9 127.8 126.9 119.6 119.2
77.2 CDCl3
67.7 66.8 63.6 62.7
49.2 49.0 47.2 46.6
31.1 28.8 23.4 21.9 21.8 19.9 19.4 17.2 16.9
O
NBn2
CN
3c
13C
f1 (ppm)

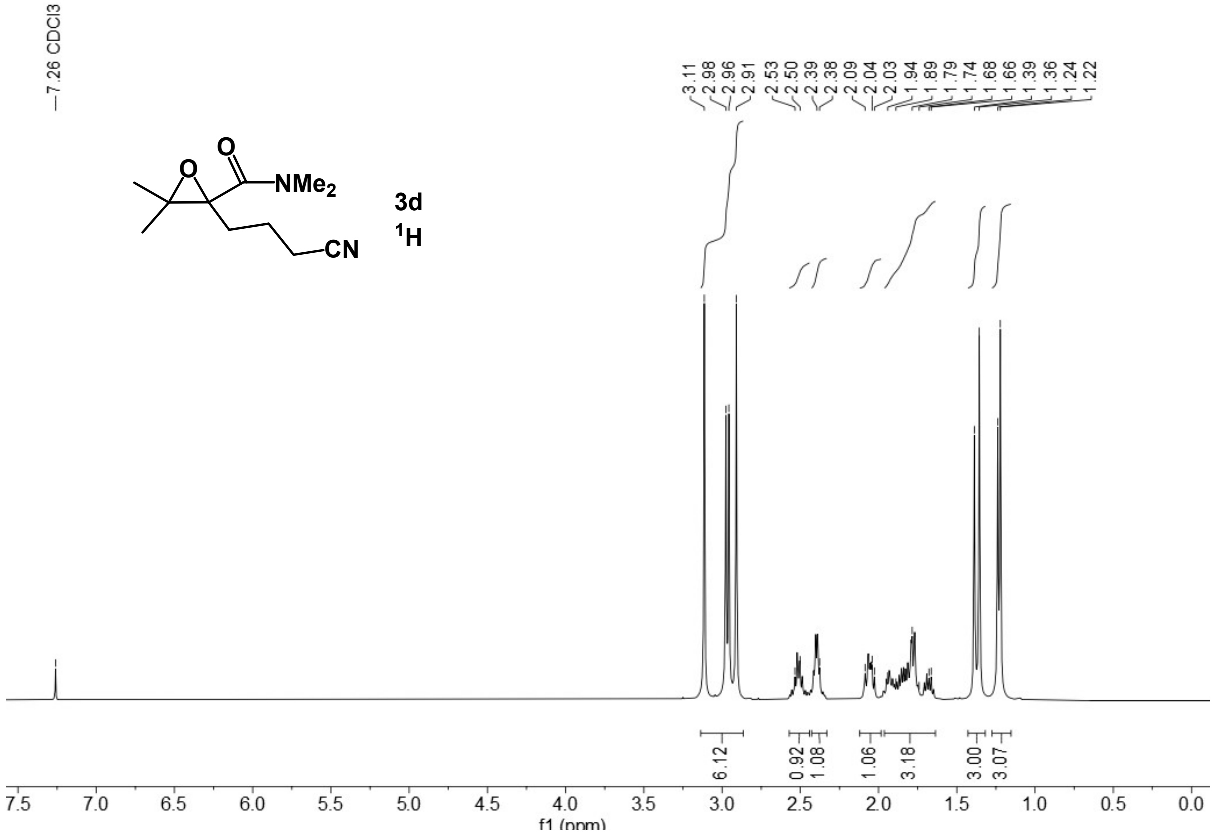
7.26 CDCl3
3.11
2.98
2.96
2.91
2.53
2.50
2.39
2.38
2.09
2.04
2.03
1.94
1.89
1.79
1.74
1.68
1.66
1.39
1.36
1.24
1.22
O
NMe2
CN
3d
1H
6.12
0.92
1.08
1.06
3.18
3.00
3.07
7.5 7.0 6.5 6.0 5.5 5.0 4.5 4.0 3.5 3.0 2.5 2.0 1.5 1.0 0.5 0.0
f1 (ppm)

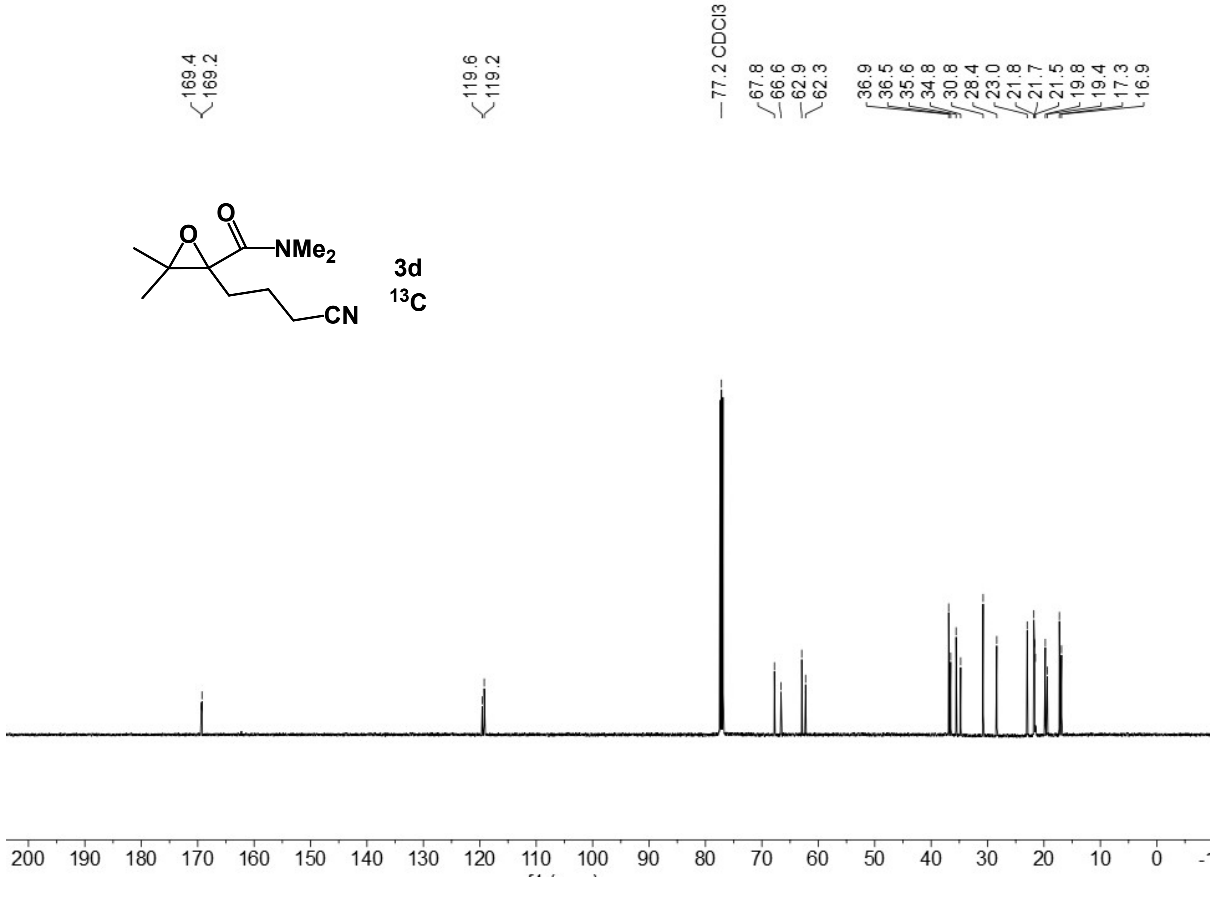
169.4
169.2
119.6
119.2
77.2 CDCl3
67.8
66.6
62.9
62.3
36.9
36.5
35.6
34.8
30.8
28.4
23.0
21.8
21.7
21.5
19.8
19.4
17.3
16.9
O
NMe2
CN
3d
13C
200 190 180 170 160 150 140 130 120 110 100 90 80 70 60 50 40 30 20 10 0

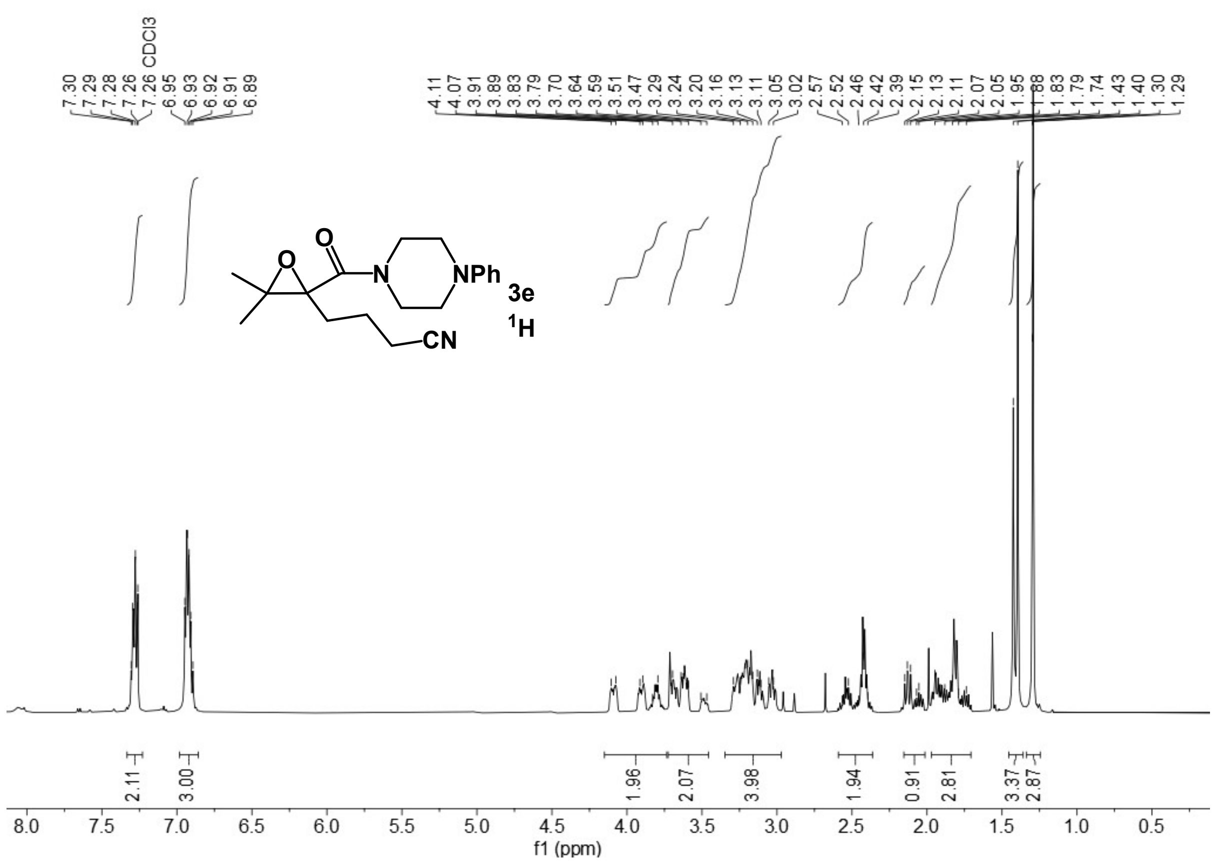
7.30 7.29 7.28 7.26 CDCl3 6.95 6.93 6.92 6.91 6.89
4.11 4.07 3.91 3.89 3.83 3.79 3.70 3.64 3.59 3.51 3.47 3.29 3.24 3.20 3.16 3.13 3.11 3.05 3.02 2.57 2.52 2.46 2.42 2.39 2.15 2.13 2.11 2.07 2.05 1.95 1.88 1.83 1.79 1.74 1.43 1.40 1.30 1.29
O
N
NPh
3e
1H
CN
2.11 3.00 1.96 2.07 3.98 1.94 0.91 2.81 3.37 2.87
8.0 7.5 7.0 6.5 6.0 5.5 5.0 4.5 4.0 3.5 3.0 2.5 2.0 1.5 1.0 0.5
f1 (ppm)

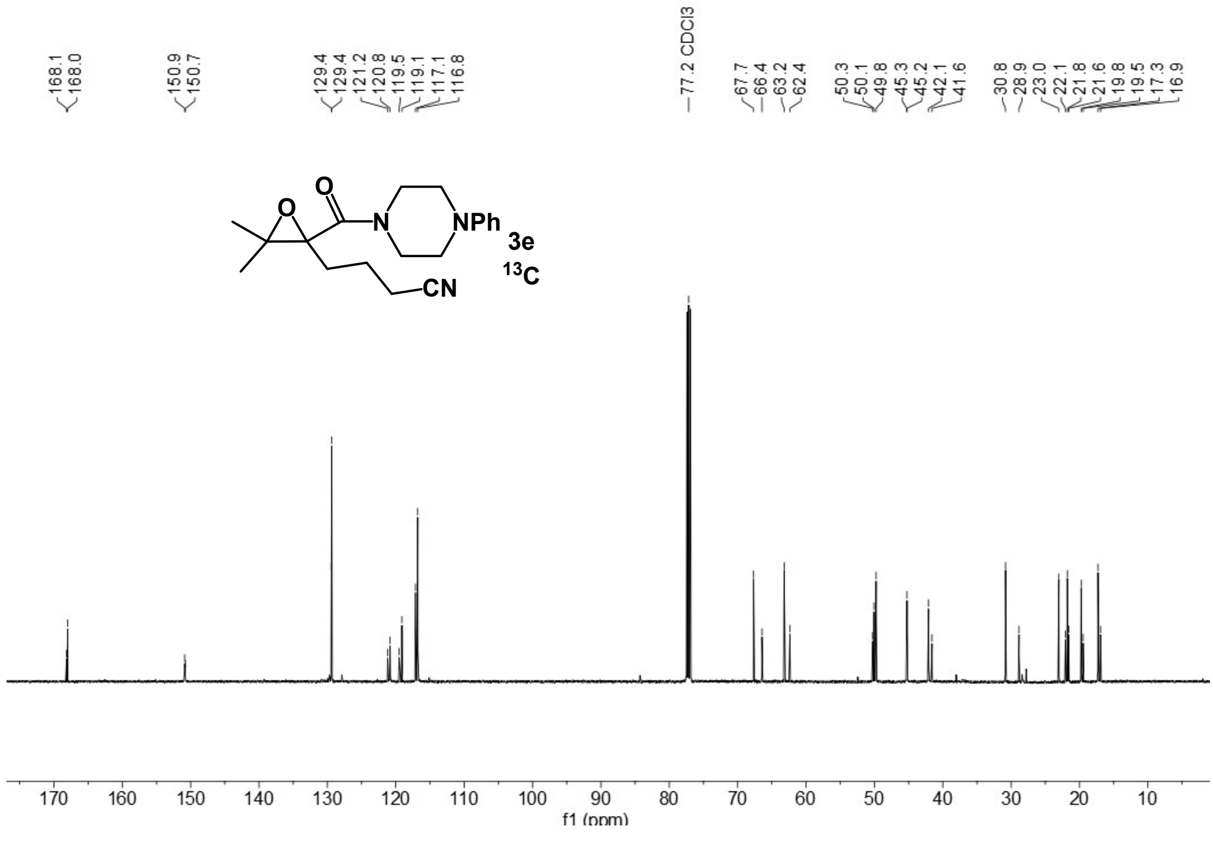
168.1 168.0 150.9 150.7 129.4 129.4 121.2 120.8 119.5 119.1 117.1 116.8 77.2 CDCl3 67.7 66.4 63.2 62.4 50.3 50.1 49.8 45.3 45.2 42.1 41.6 30.8 28.9 23.0 22.1 21.8 21.6 19.8 19.5 17.3 16.9
O
N
NPh
3e
13C
CN
170 160 150 140 130 120 110 100 90 80 70 60 50 40 30 20 10
f1 (ppm)

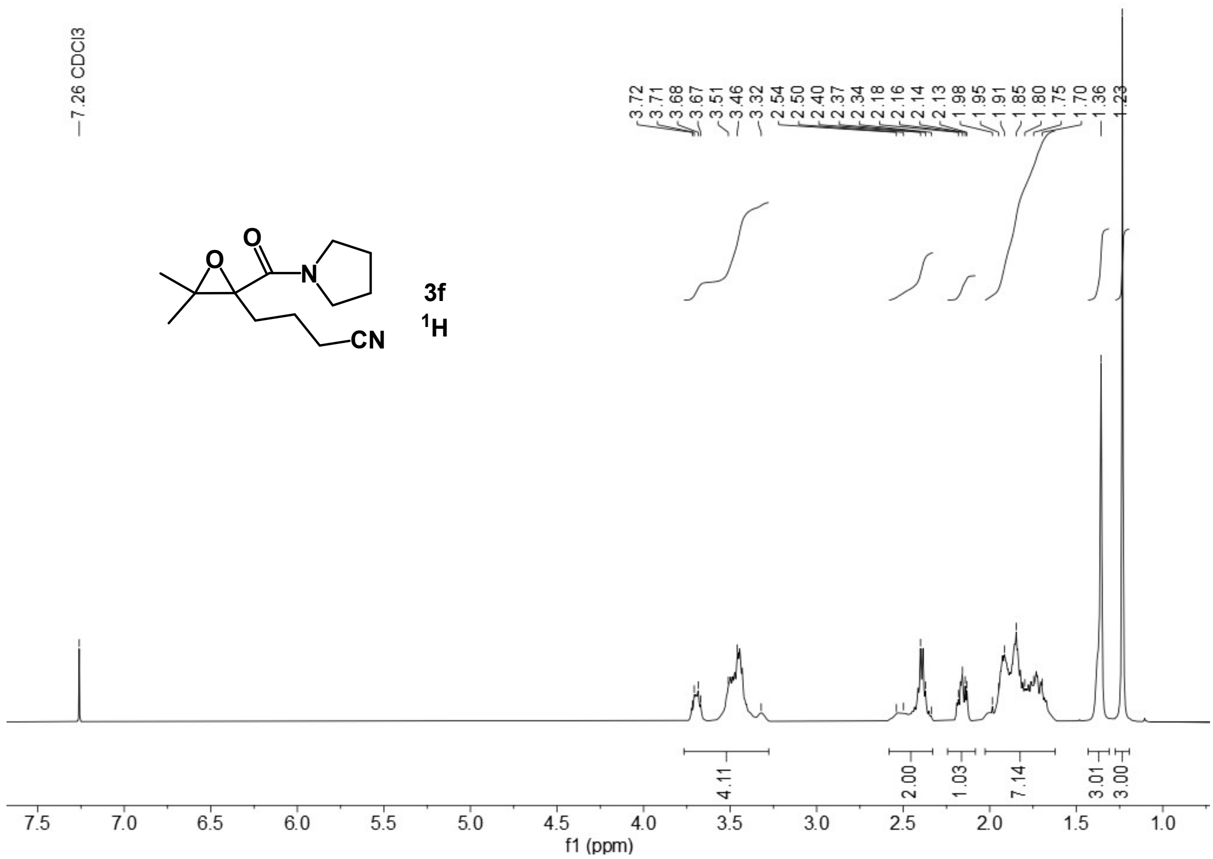

7.26 CDCl3
3.72
3.71
3.68
3.67
3.51
3.46
3.32
2.54
2.50
2.40
2.37
2.34
2.18
2.16
2.14
2.13
1.98
1.95
1.91
1.85
1.80
1.75
1.70
1.36
1.23
O
N
CN
3f
1H
4.11
2.00
1.03
7.14
3.01
3.00
f1 (ppm)

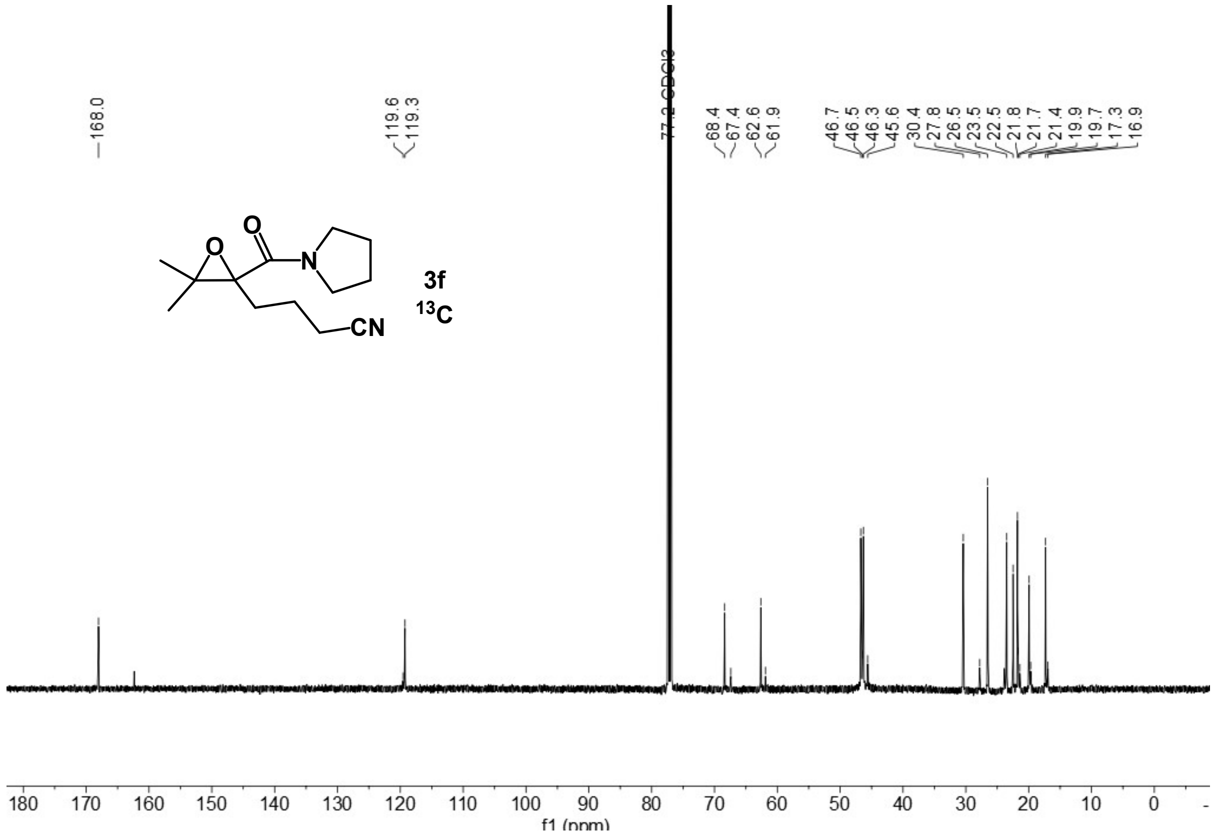

168.0
119.6
119.3
77.2 CDCl3
68.4
67.4
62.6
61.9
46.7
46.5
46.3
45.6
30.4
27.8
26.5
23.5
22.5
21.8
21.7
21.4
19.9
19.7
17.3
16.9
O
N
CN
3f
13C

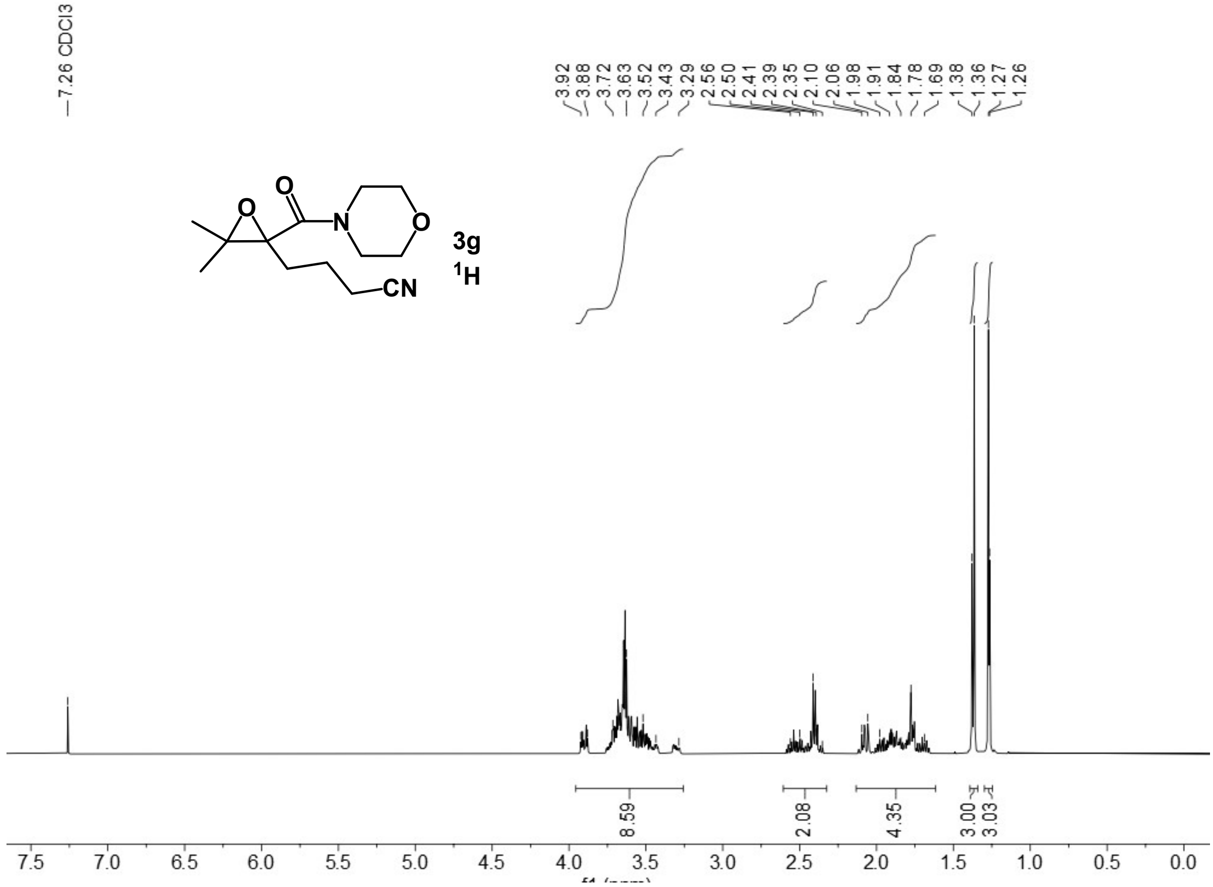
7.26 CDCl3
3.92
3.88
3.72
3.63
3.52
3.43
3.29
2.56
2.50
2.41
2.39
2.35
2.10
2.06
1.98
1.91
1.84
1.78
1.69
1.38
1.36
1.27
1.26
O
N
O
O
CN
3g
^{1}H
8.59
2.08
4.35
3.00
3.03
7.5 7.0 6.5 6.0 5.5 5.0 4.5 4.0 3.5 3.0 2.5 2.0 1.5 1.0 0.5 0.0

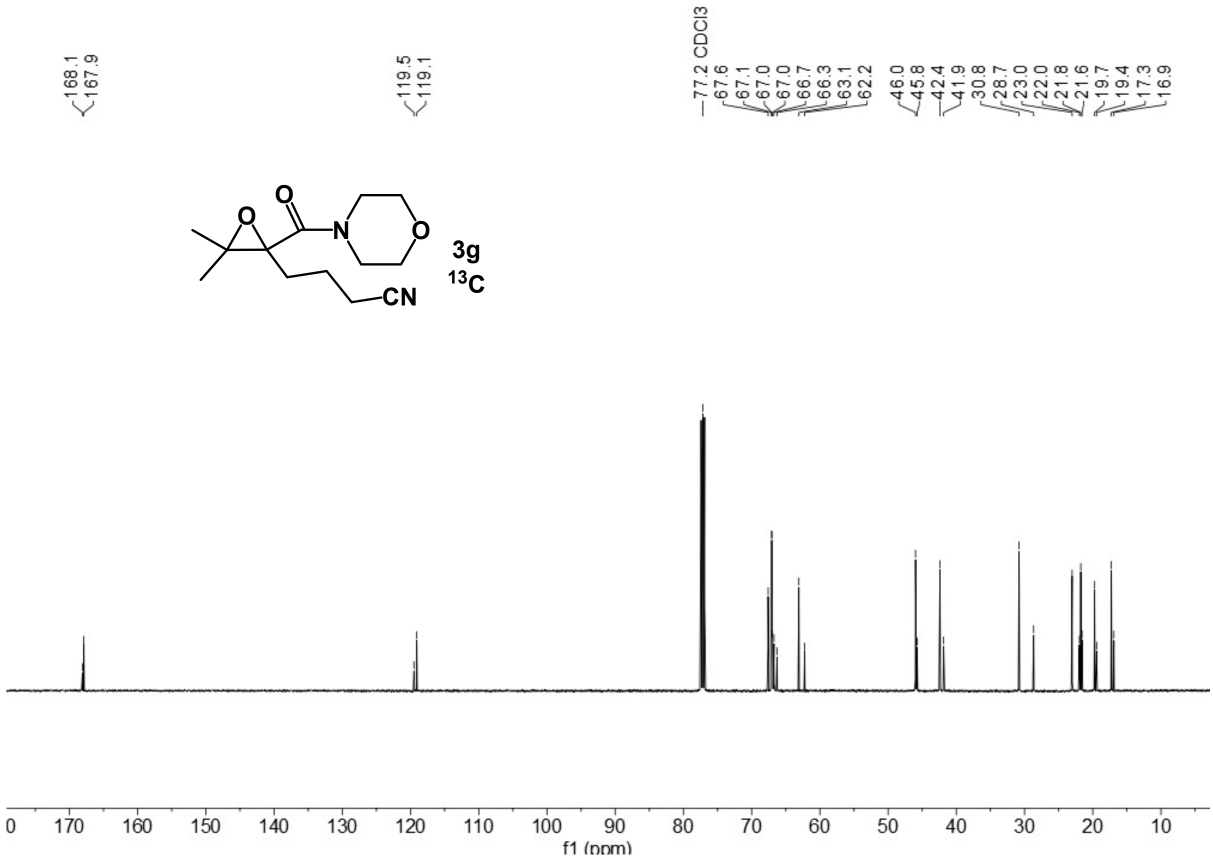
168.1
167.9
119.5
119.1
77.2 CDCl3
67.6
67.1
67.0
67.0
66.7
66.3
63.1
62.2
46.0
45.8
42.4
41.9
30.8
28.7
23.0
22.0
21.8
21.6
19.7
19.4
17.3
16.9
O
N
O
O
CN
3g
^{13}C
170 160 150 140 130 120 110 100 90 80 70 60 50 40 30 20 10
f1 (ppm)

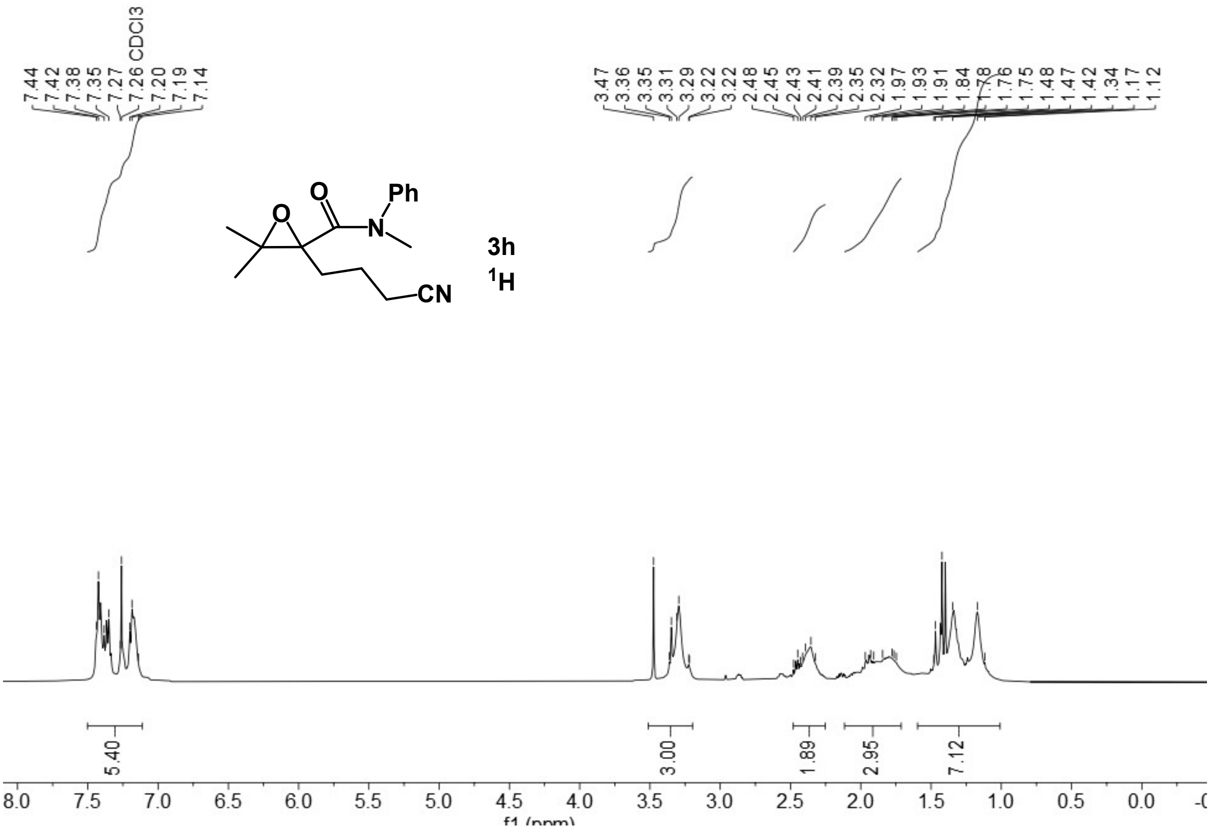
7.44 7.42 7.38 7.35 7.27 7.26 CDCl3 7.20 7.19 7.14
3.47 3.36 3.35 3.31 3.29 3.22 3.22 2.48 2.45 2.43 2.41 2.39 2.35 2.32 1.97 1.93 1.91 1.84 1.78 1.76 1.75 1.48 1.47 1.42 1.34 1.17 1.12
O
N
Ph
CN
3h
1H
5.40
3.00
1.89
2.95
7.12
f1 (ppm)

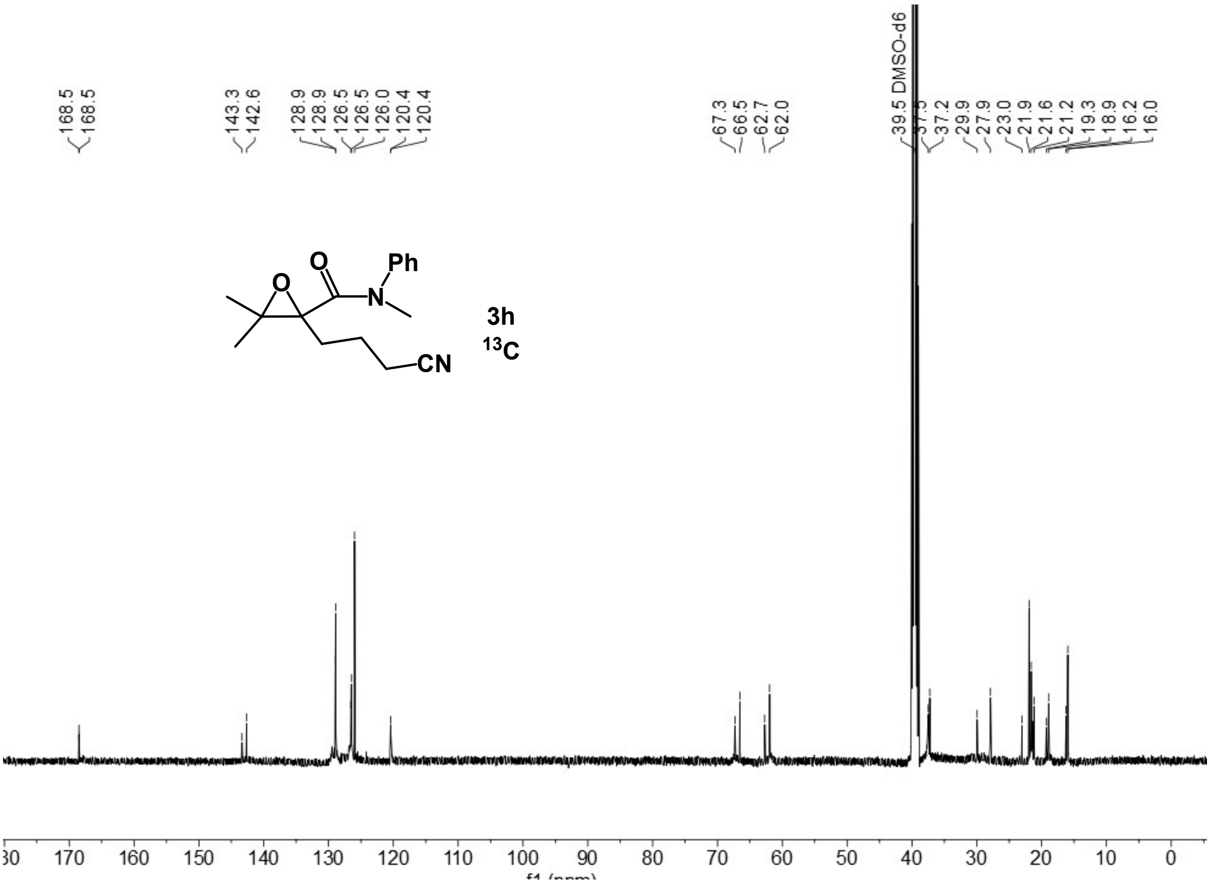
168.5 168.5
143.3 142.6
128.9 128.9 126.5 126.5 126.0 120.4 120.4
67.3 66.5 62.7 62.0
39.5 DMSO-d6
37.2 29.9 27.9 23.0 21.9 21.6 21.2 19.3 18.9 16.2 16.0
O
N
Ph
CN
3h
13C
f1 (ppm)

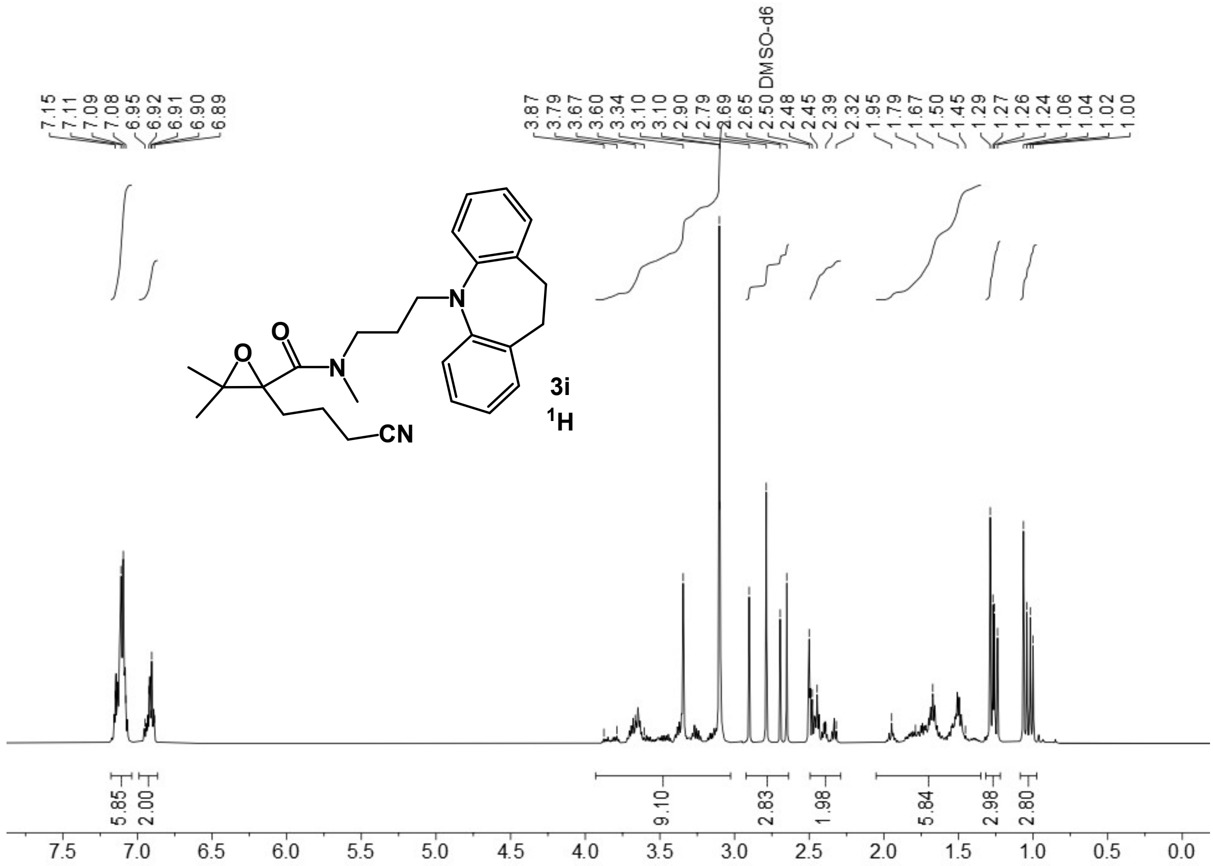
3i
1H
f1 (ppm)
DMSO-d6

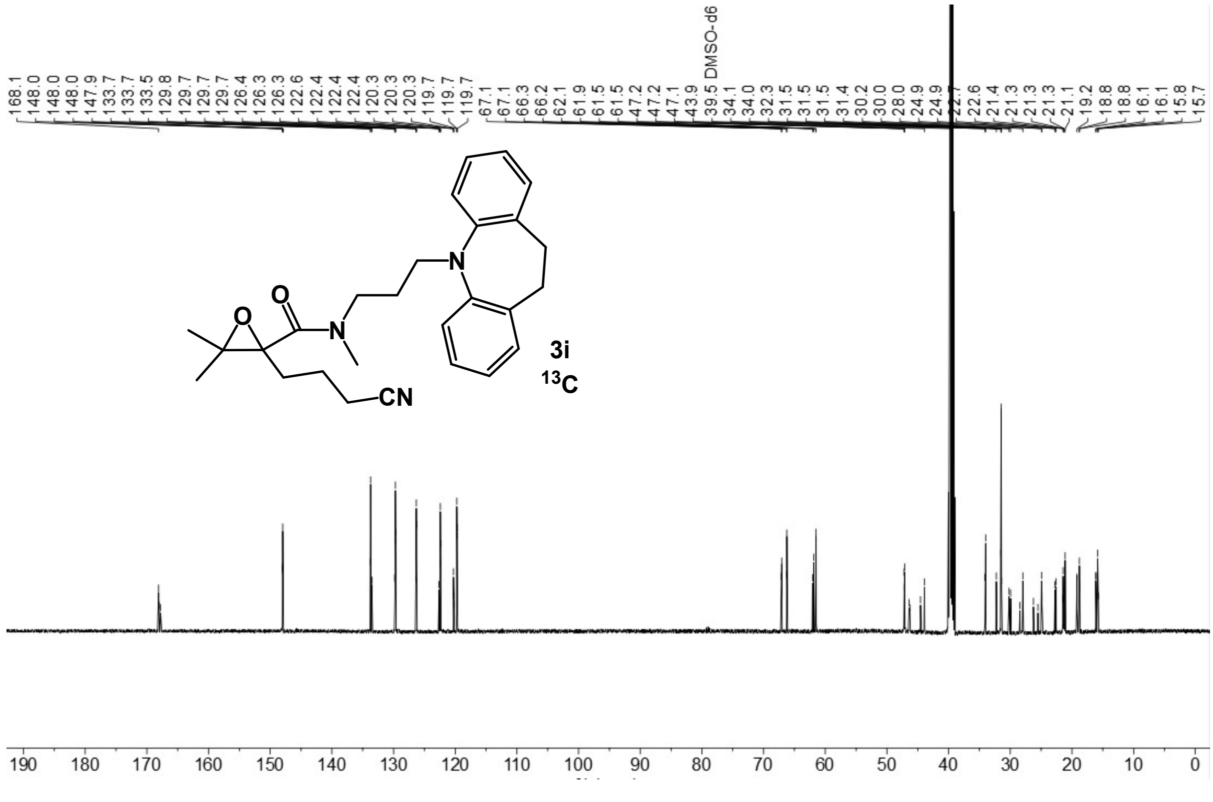
3i
13C
DMSO-d6

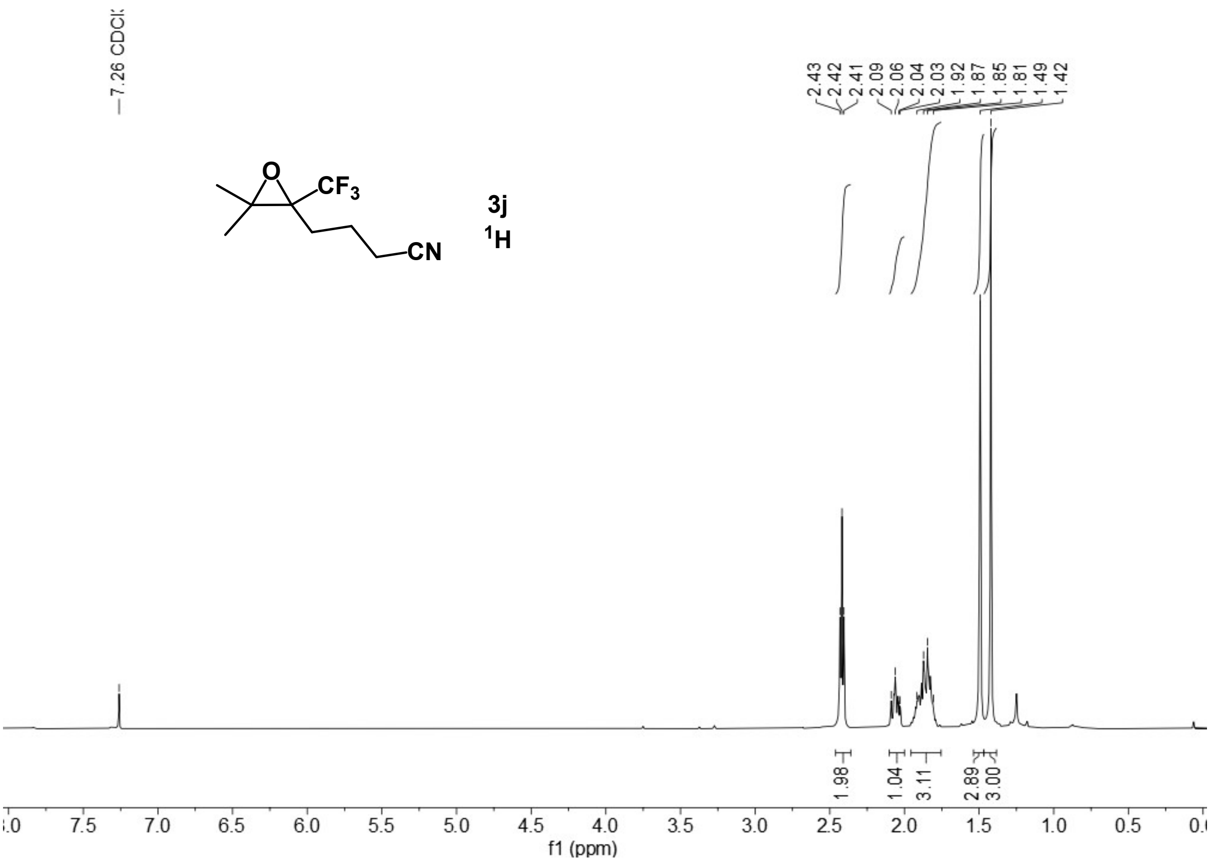
7.26 CDCl3
2.43
2.42
2.41
2.09
2.06
2.04
2.03
1.92
1.87
1.85
1.81
1.49
1.42
CF3
O
CN
3j
1H
1.98
1.04
3.11
2.89
3.00
7.5
7.0
6.5
6.0
5.5
5.0
4.5
4.0
3.5
3.0
2.5
2.0
1.5
1.0
0.5
f1 (ppm)

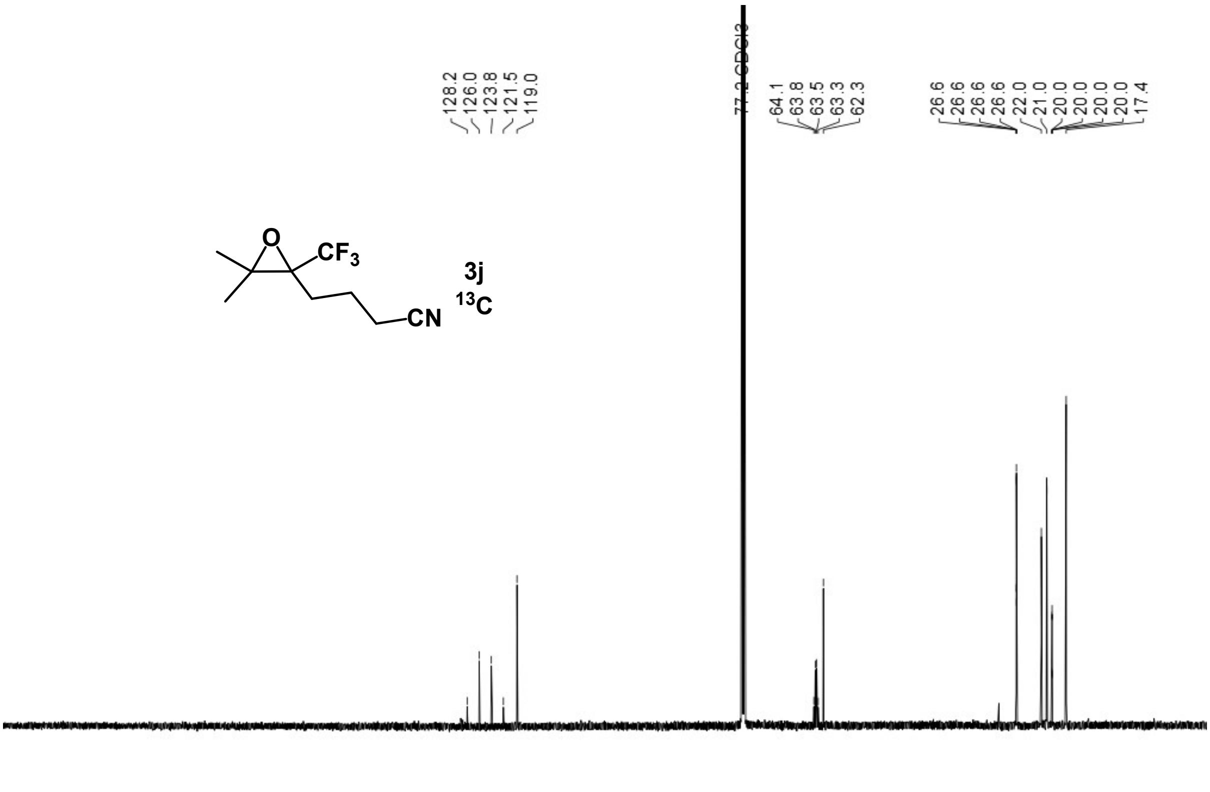
128.2
126.0
123.8
121.5
119.0
77.2 CDCl3
64.1
63.8
63.5
63.3
62.3
26.6
26.6
26.6
26.6
22.0
21.0
20.0
20.0
20.0
20.0
17.4
O
CF3
CN
3j
13C
210
200
190
180
170
160
150
140
130
120
110
100
90
80
70
60
50
40
30
20
10
0
f1 (ppm)

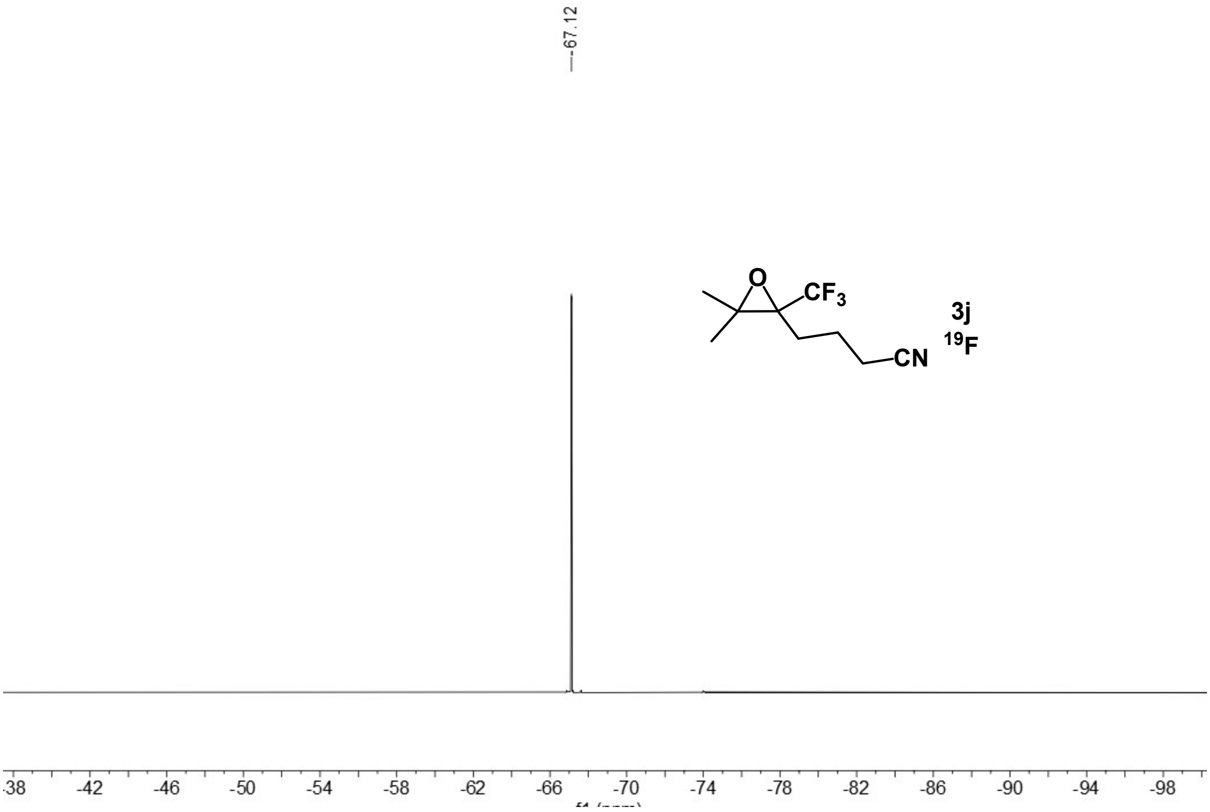
-67.12
3j
^{19}F
-38
-42
-46
-50
-54
-58
-62
-66
-70
-74
-78
-82
-86
-90
-94
-98

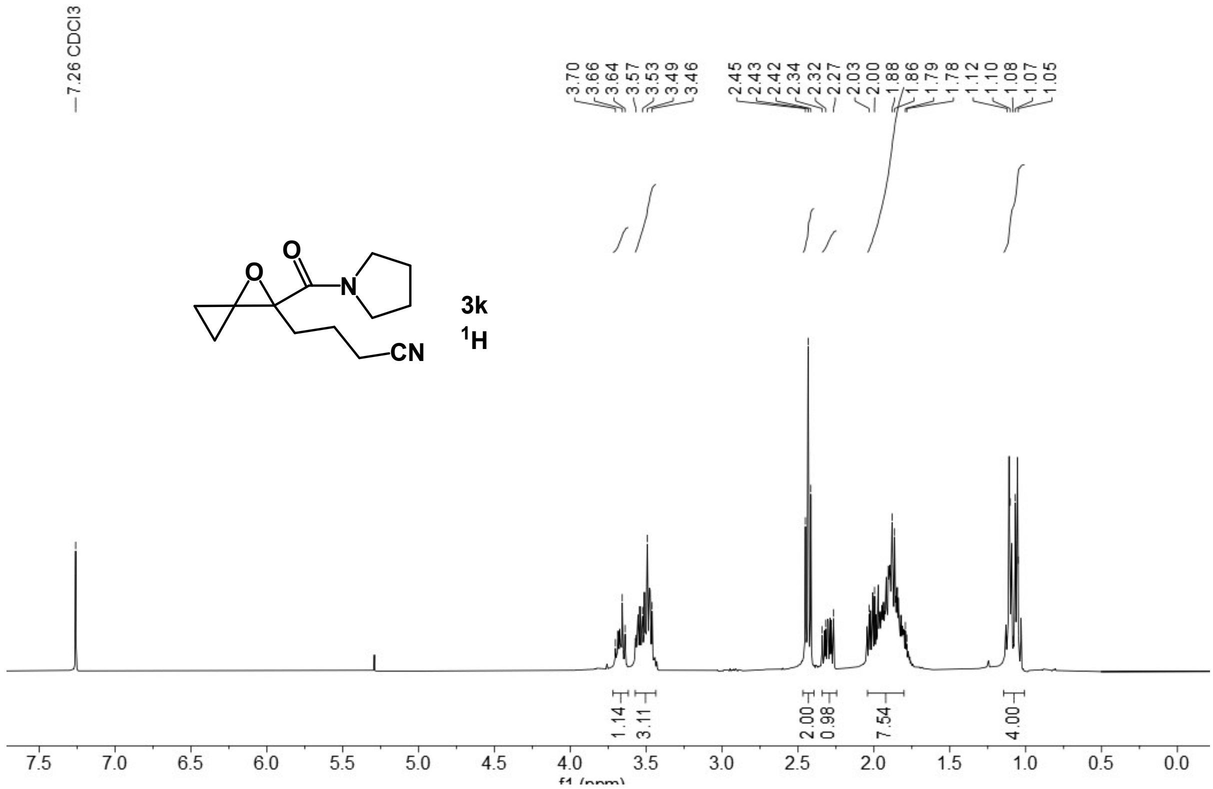
3k
1H
7.26 CDCl3
3.70 3.66 3.64 3.57 3.53 3.49 3.46
2.45 2.43 2.42 2.34 2.32 2.27 2.03 2.00 1.88 1.86 1.79 1.78 1.12 1.10 1.08 1.07 1.05
1.14 3.11 2.00 0.98 7.54 4.00
f1 (ppm)

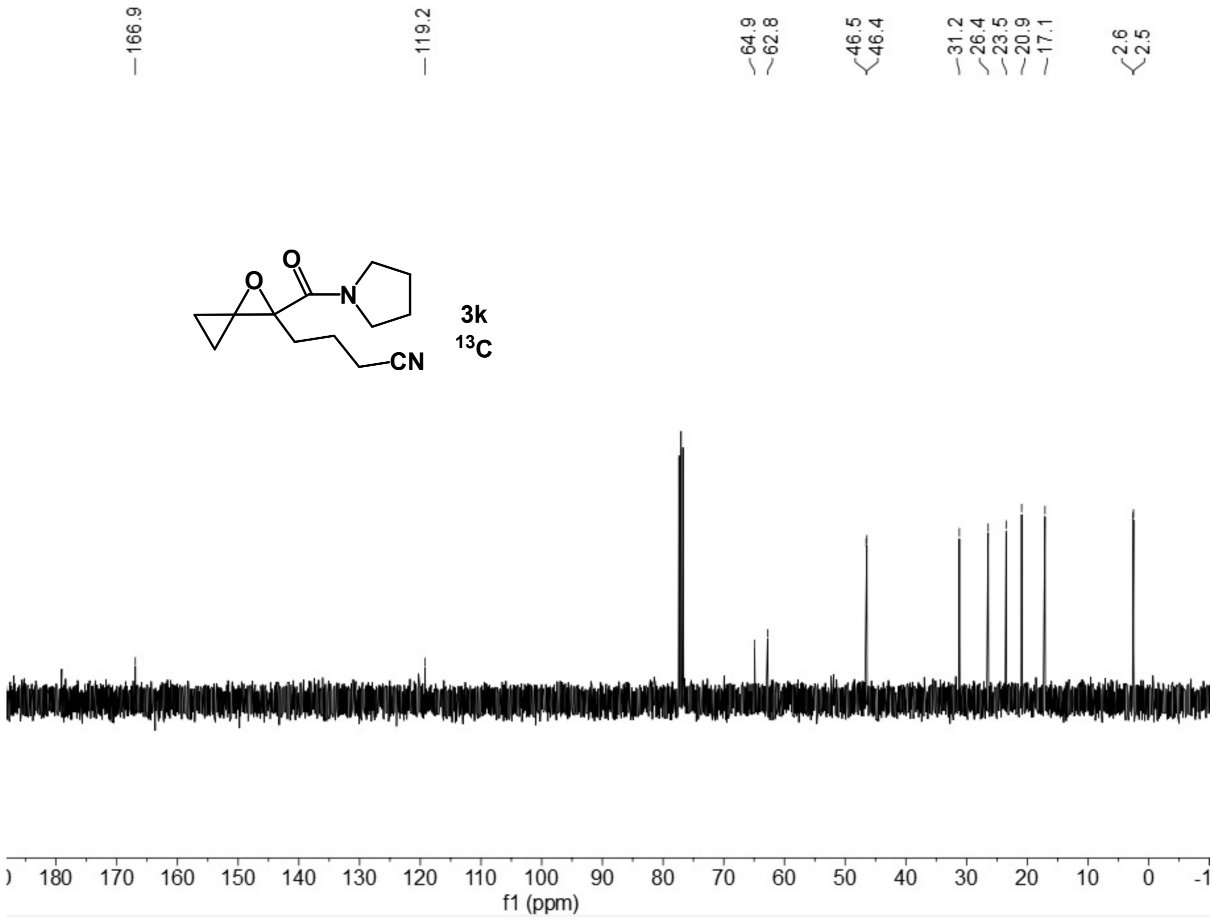
3k
13C
166.9
119.2
64.9 62.8
46.5 46.4
31.2 26.4 23.5 20.9 17.1
2.6 2.5
f1 (ppm)

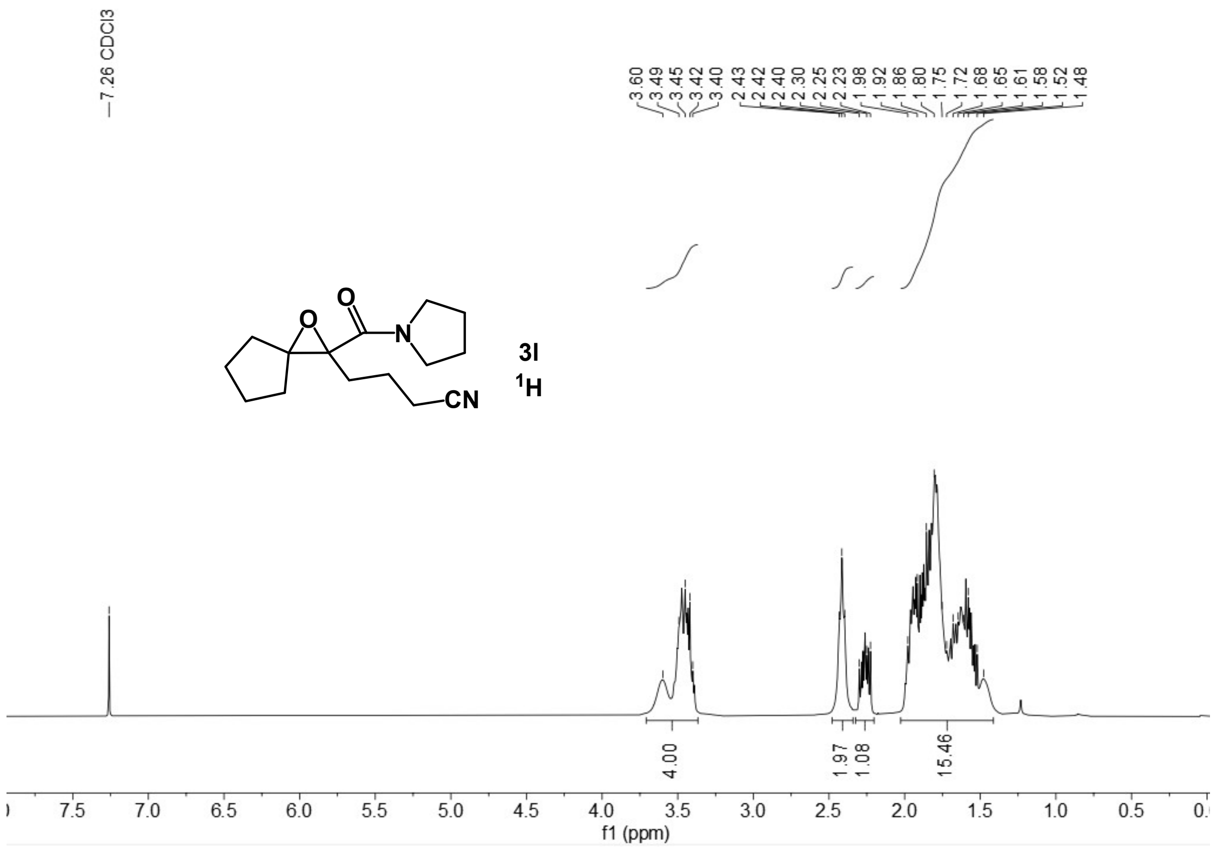

3l
1H
CN
O
N
7.26 CDCl3
3.60 3.49 3.45 3.42 3.40 2.43 2.42 2.40 2.30 2.25 2.23 1.98 1.92 1.86 1.80 1.75 1.72 1.68 1.65 1.61 1.58 1.52 1.48
4.00
1.97
1.08
15.46
f1 (ppm)

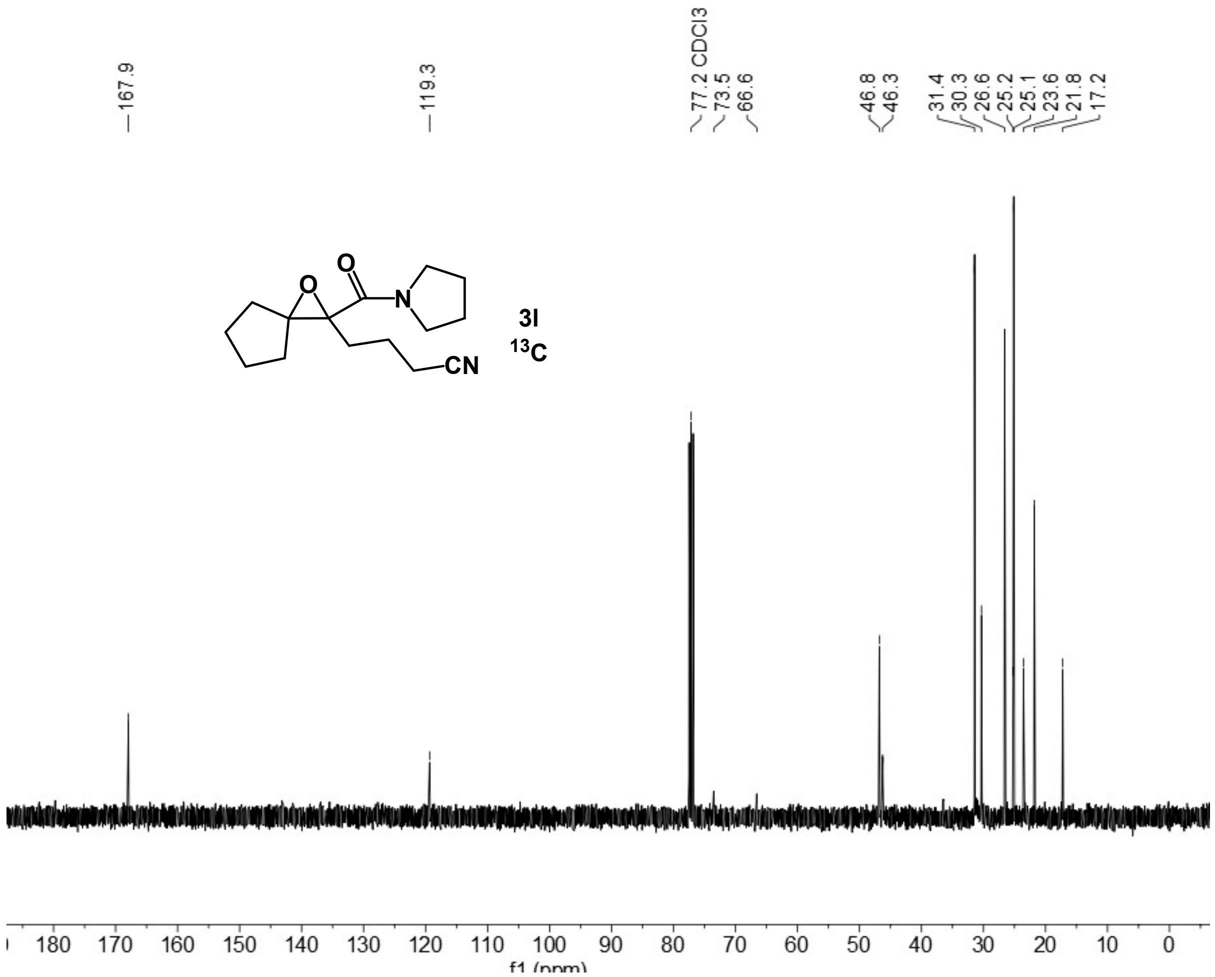

3l
13C
CN
O
N
167.9
119.3
77.2 CDCl3 73.5 66.6
46.8 46.3
31.4 30.3 26.6 25.2 25.1 23.6 21.8 17.2
f1 (ppm)

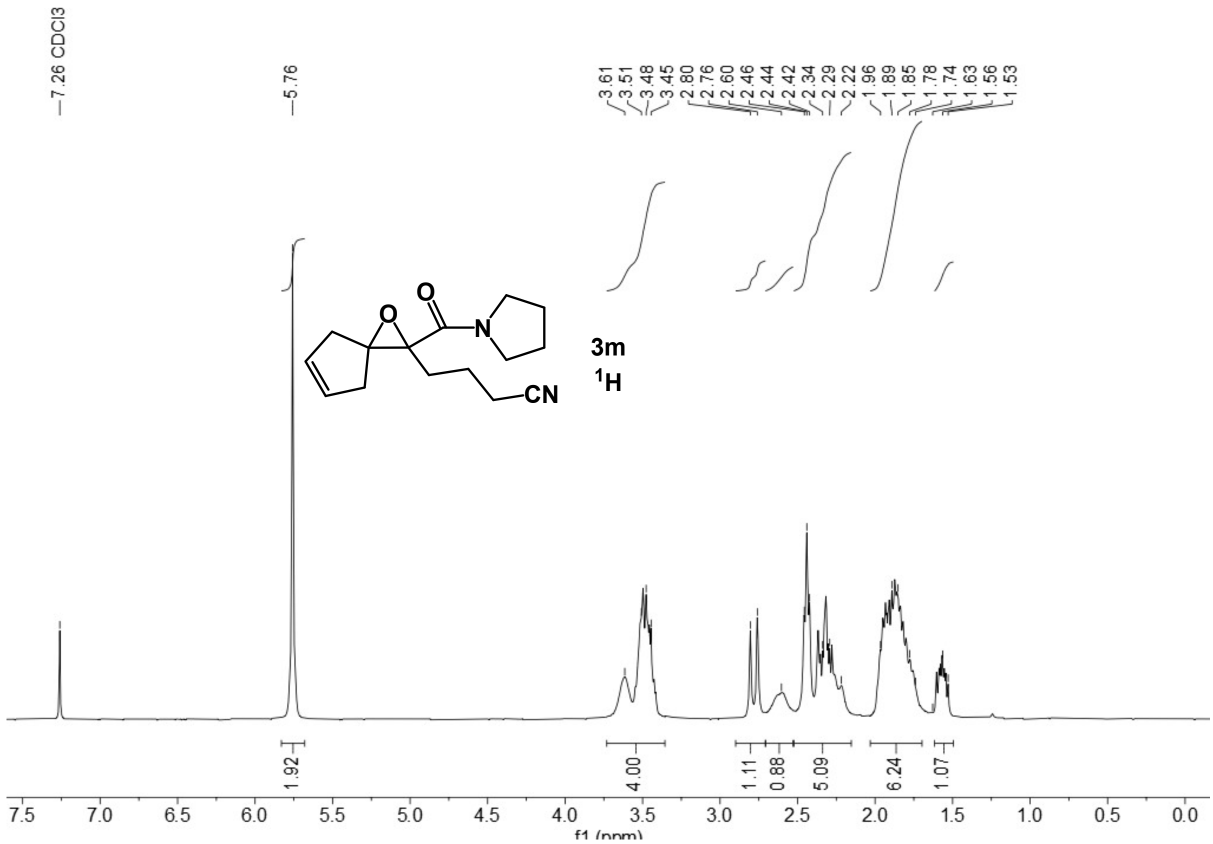
7.26 CDCl3
5.76
3.61
3.51
3.48
3.45
2.80
2.76
2.60
2.46
2.44
2.42
2.34
2.29
2.22
1.96
1.89
1.85
1.78
1.74
1.63
1.56
1.53
O
O
N
CN
3m
1H
1.92
4.00
1.11
0.88
5.09
6.24
1.07
f1 (ppm)

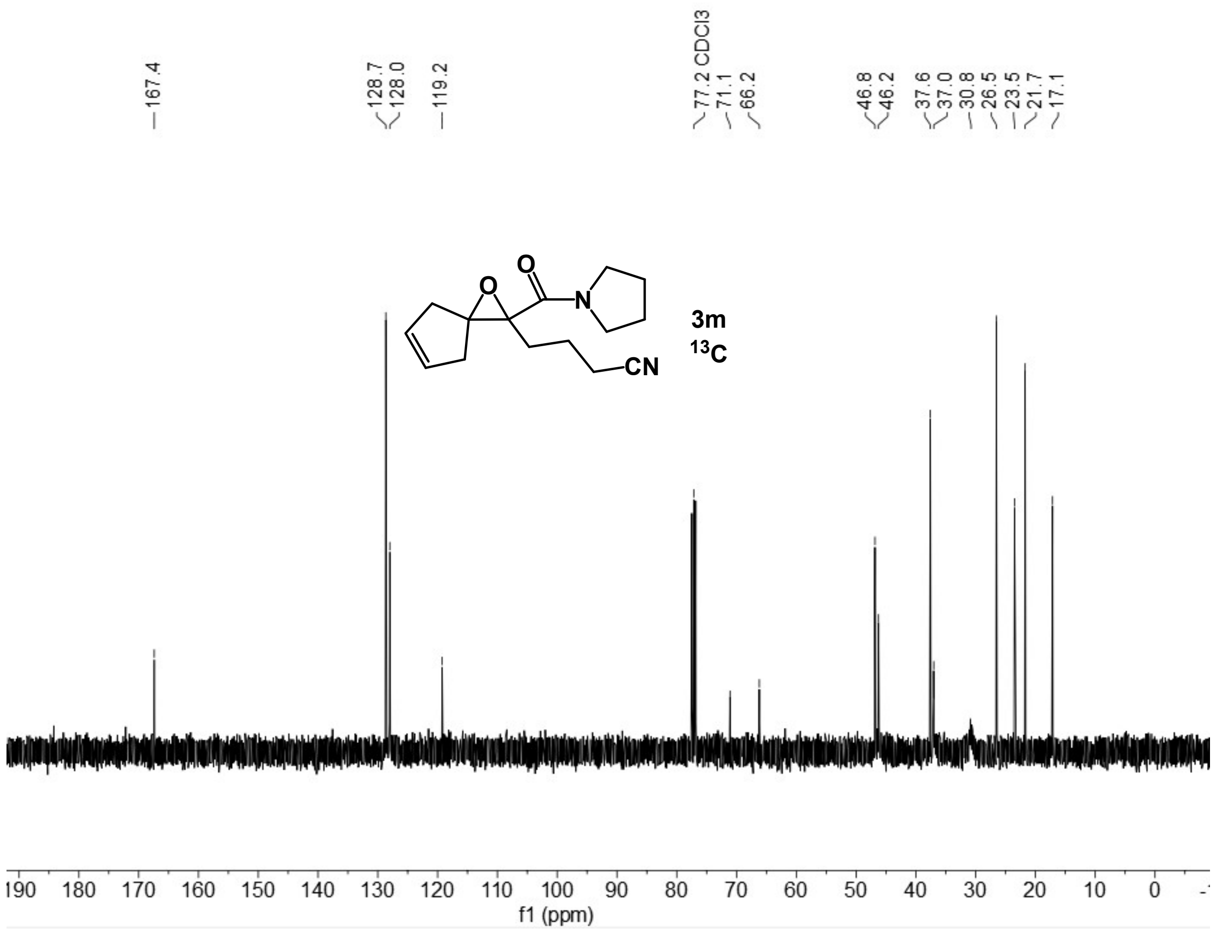
167.4
128.7
128.0
119.2
77.2 CDCl3
71.1
66.2
46.8
46.2
37.6
37.0
30.8
26.5
23.5
21.7
17.1
O
O
N
CN
3m
13C
f1 (ppm)

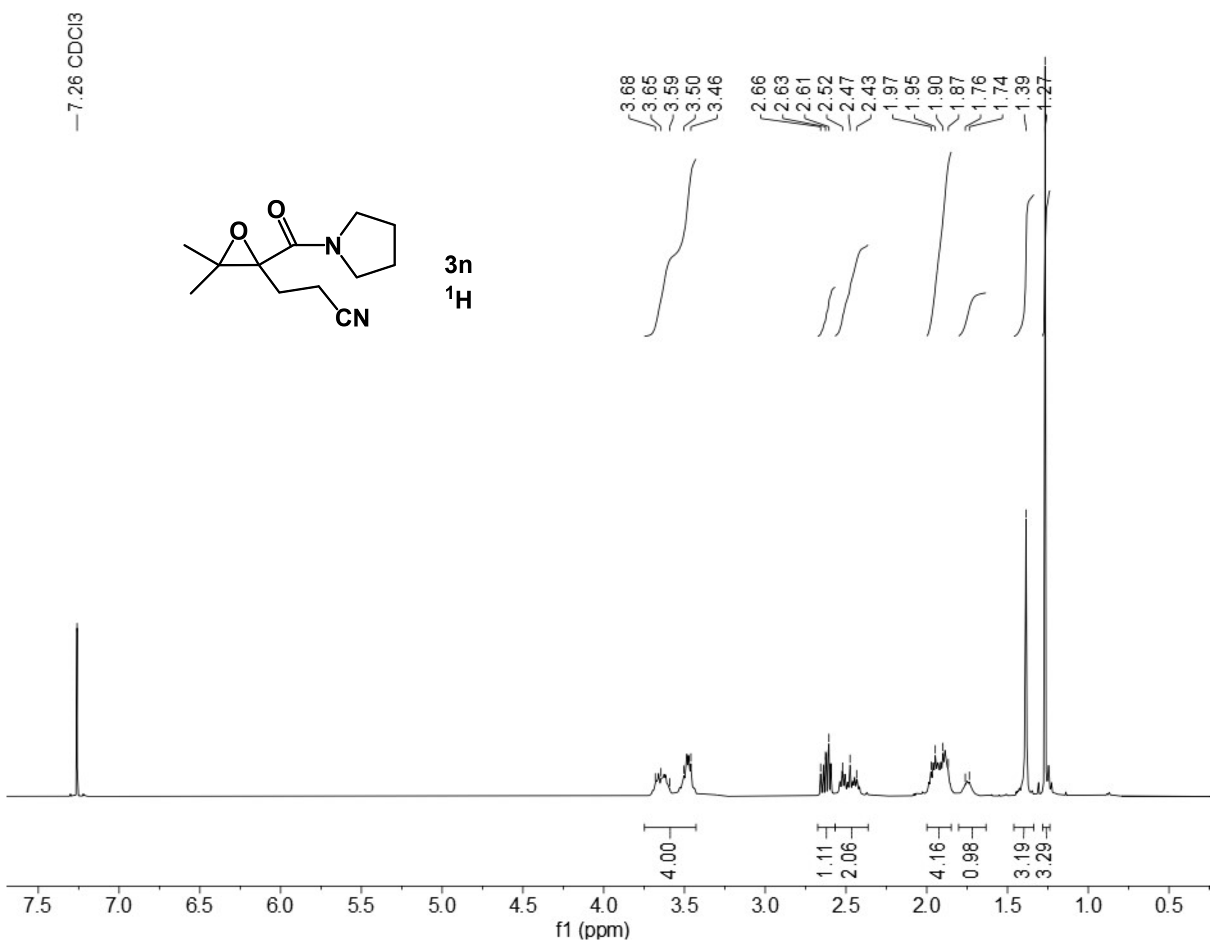

7.26 CDCl3
3.68
3.65
3.59
3.50
3.46
2.66
2.63
2.61
2.52
2.47
2.43
1.97
1.95
1.90
1.87
1.76
1.74
1.39
1.27
O
N
CN
3n
1H
4.00
1.11
2.06
4.16
0.98
3.19
3.29
7.5 7.0 6.5 6.0 5.5 5.0 4.5 4.0 3.5 3.0 2.5 2.0 1.5 1.0 0.5
f1 (ppm)

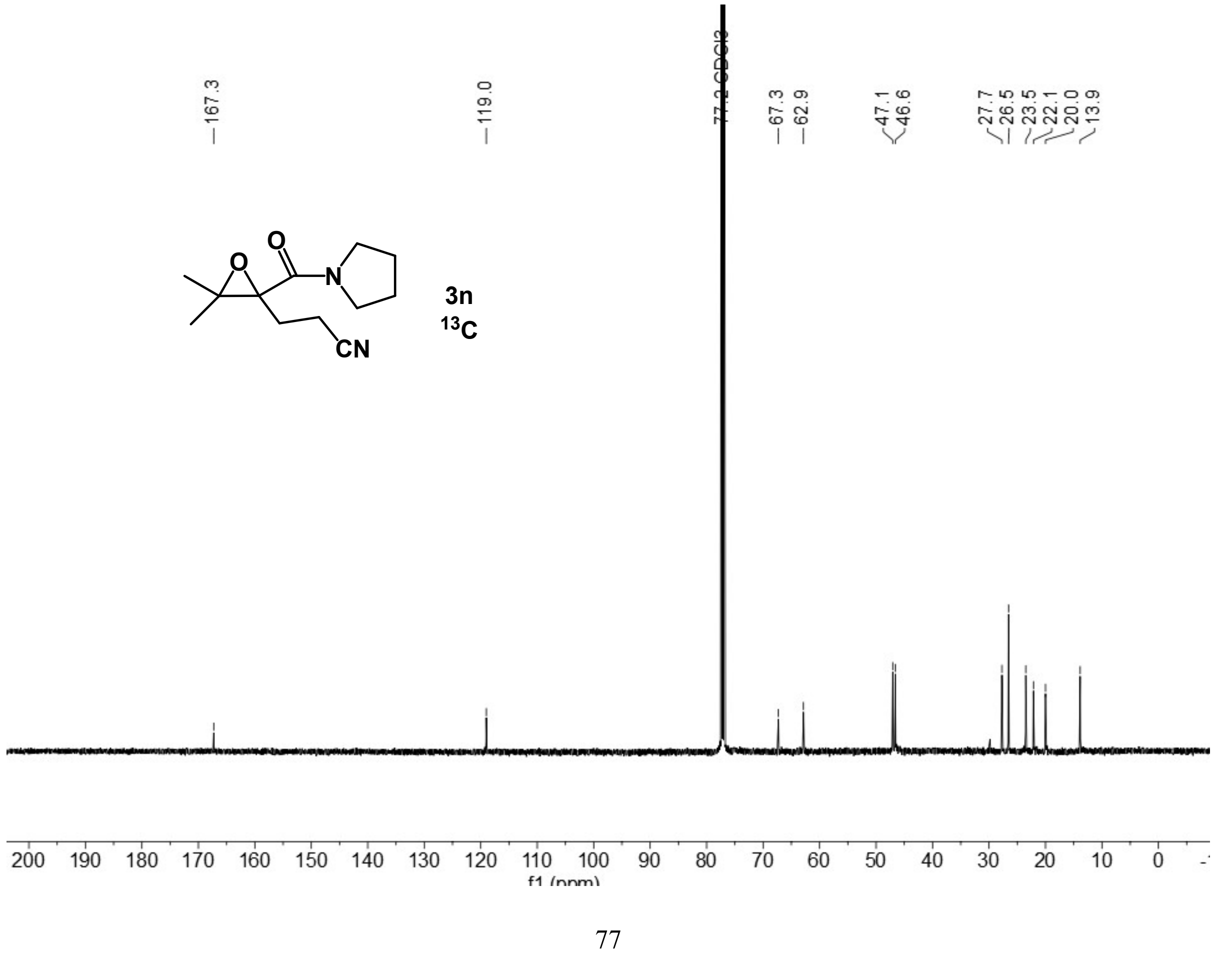

167.3
119.0
77.2 CDCl3
67.3
62.9
47.1
46.6
27.7
26.5
23.5
22.1
20.0
13.9
O
N
CN
3n
13C
200 190 180 170 160 150 140 130 120 110 100 90 80 70 60 50 40 30 20 10 0
f1 (ppm)

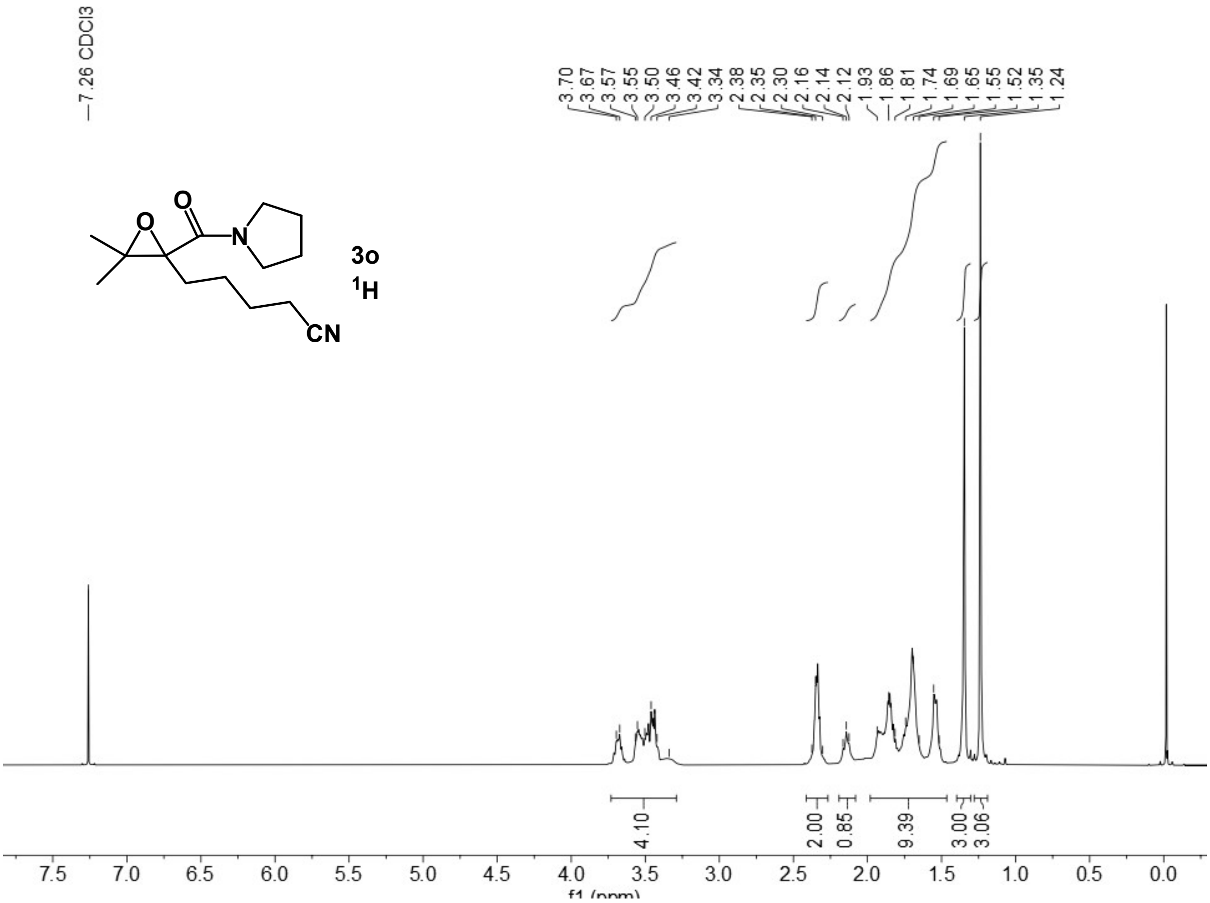
7.26 CDCl3
3.70 3.67 3.57 3.55 3.50 3.46 3.42 3.34 2.38 2.35 2.30 2.16 2.14 2.12 1.93 1.86 1.81 1.74 1.69 1.65 1.55 1.52 1.35 1.24
O
N
CN
3o
1H
4.10
2.00
0.85
9.39
3.00
3.06
7.5 7.0 6.5 6.0 5.5 5.0 4.5 4.0 3.5 3.0 2.5 2.0 1.5 1.0 0.5 0.0

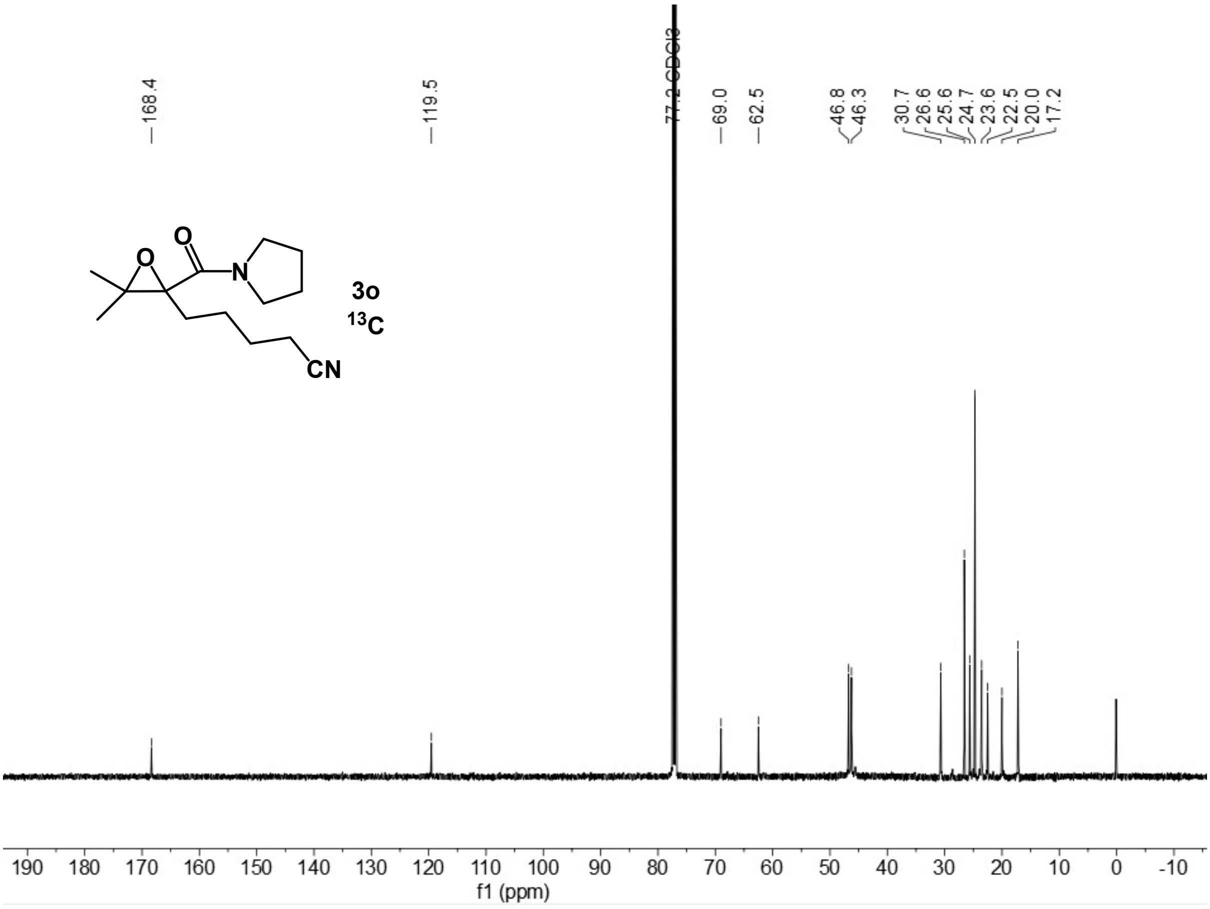
168.4
119.5
77.2 CDCl3
69.0
62.5
46.8 46.3
30.7 26.6 25.6 24.7 23.6 22.5 20.0 17.2
O
N
CN
3o
13C
190 180 170 160 150 140 130 120 110 100 90 80 70 60 50 40 30 20 10 0 -10
f1 (ppm)

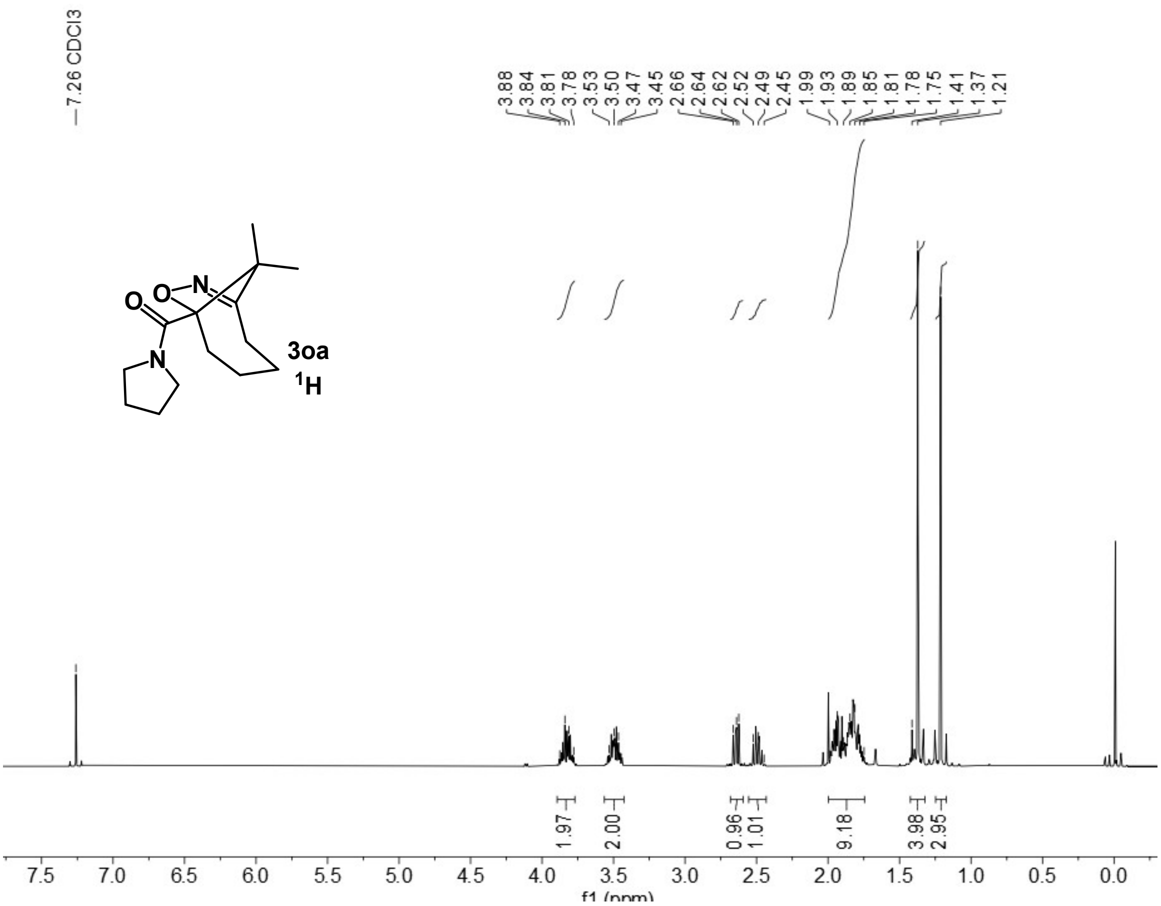
7.26 CDCl3
3.88
3.84
3.81
3.78
3.53
3.50
3.47
3.45
2.66
2.64
2.62
2.52
2.49
2.45
1.99
1.93
1.89
1.85
1.81
1.78
1.75
1.41
1.37
1.21
3oa
1H
1.97
2.00
0.96
1.01
9.18
3.98
2.95
7.5 7.0 6.5 6.0 5.5 5.0 4.5 4.0 3.5 3.0 2.5 2.0 1.5 1.0 0.5 0.0
f1 (ppm)

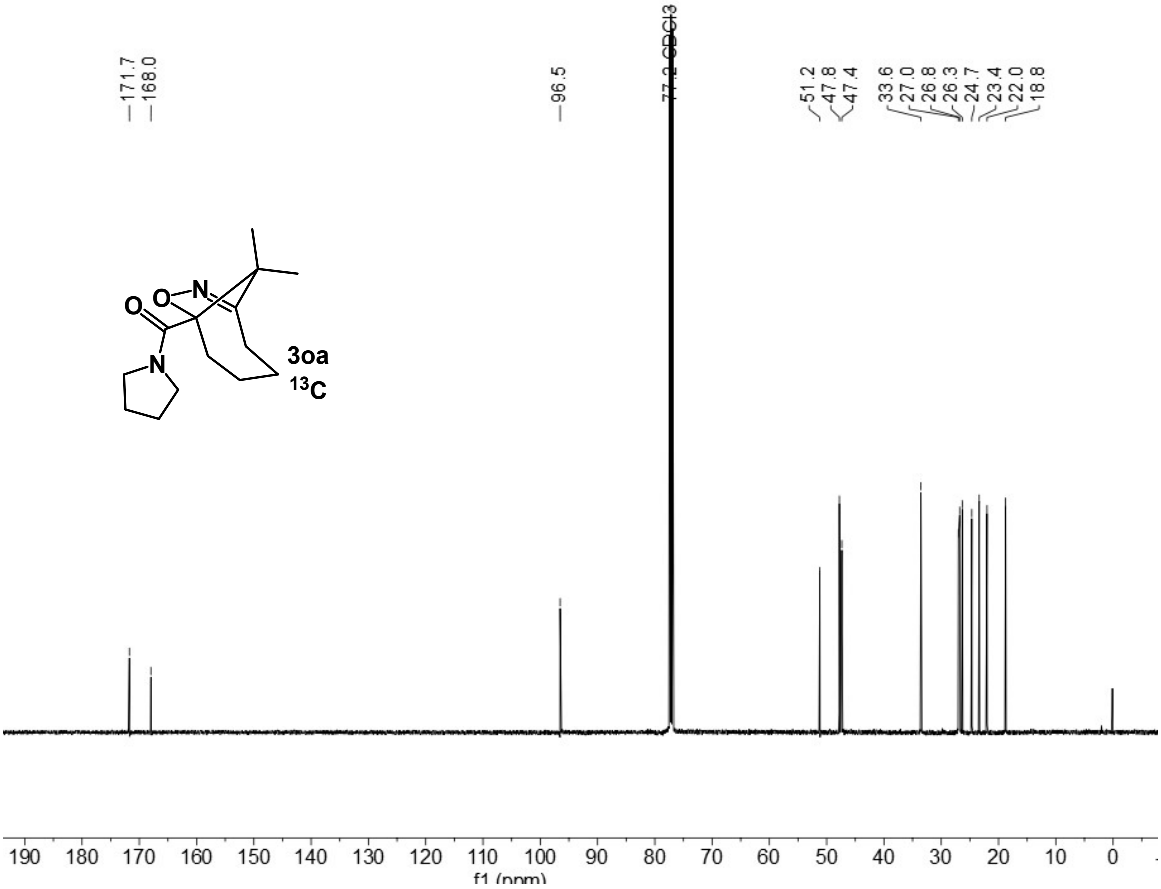
171.7
168.0
96.5
77.2 CDCl3
51.2
47.8
47.4
33.6
27.0
26.8
26.3
24.7
23.4
22.0
18.8
3oa
13C
190 180 170 160 150 140 130 120 110 100 90 80 70 60 50 40 30 20 10 0
f1 (ppm)

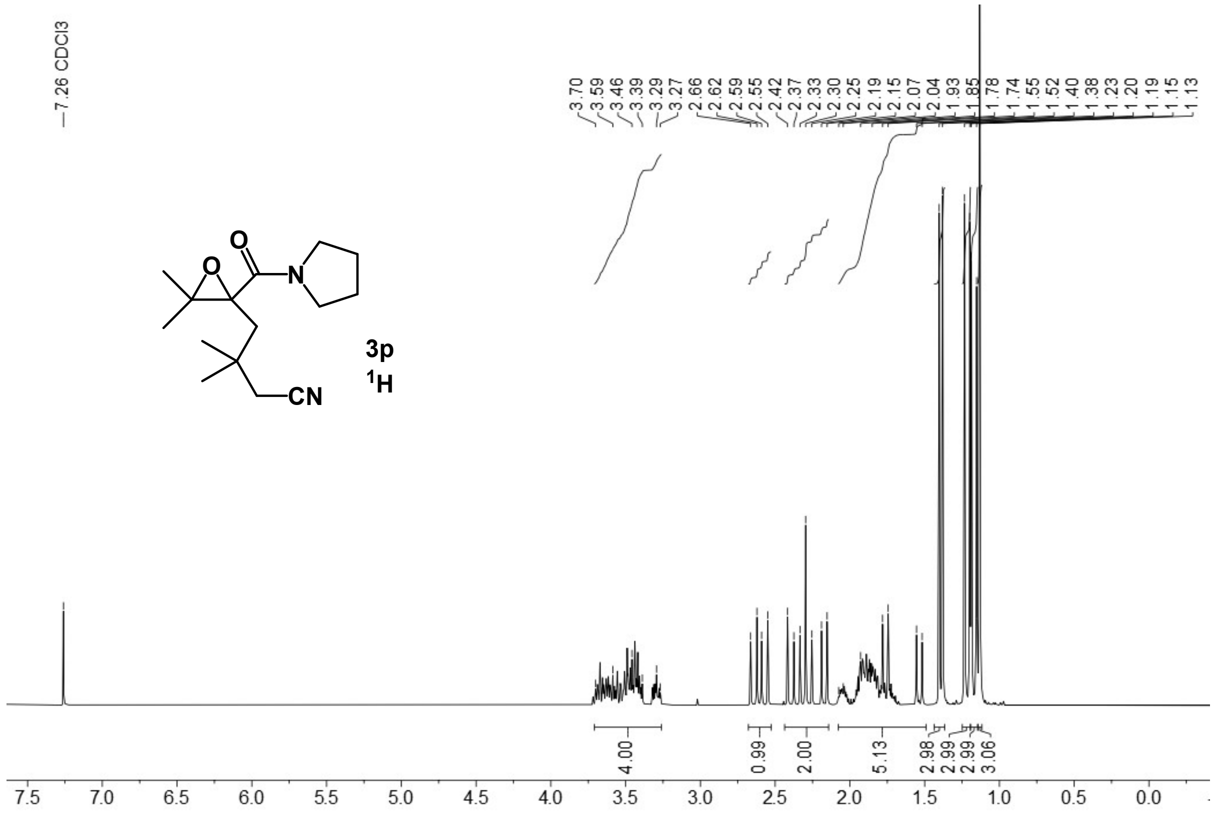
7.26 CDCl3
O
N
CN
3p
1H
4.00
0.99
2.00
5.13
2.98
2.99
2.99
3.06

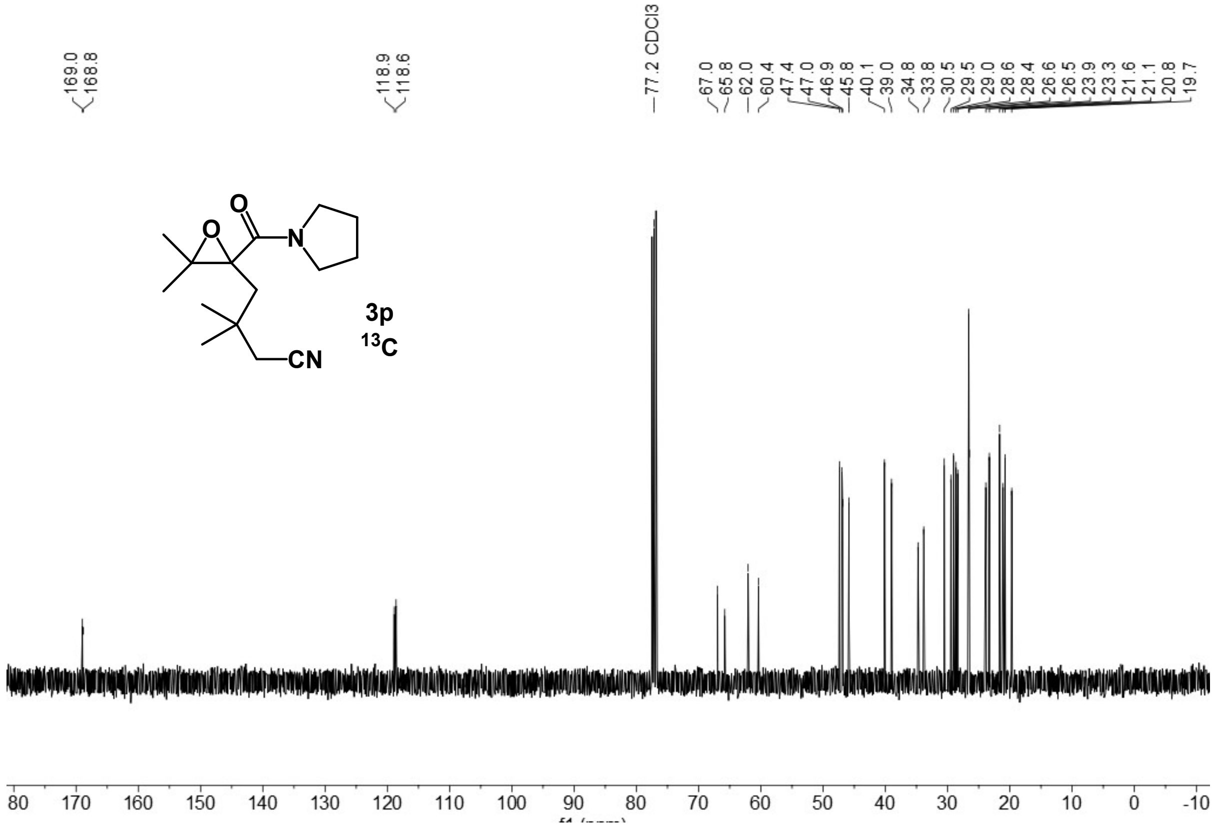
169.0
168.8
118.9
118.6
77.2 CDCl3
O
N
CN
3p
13C

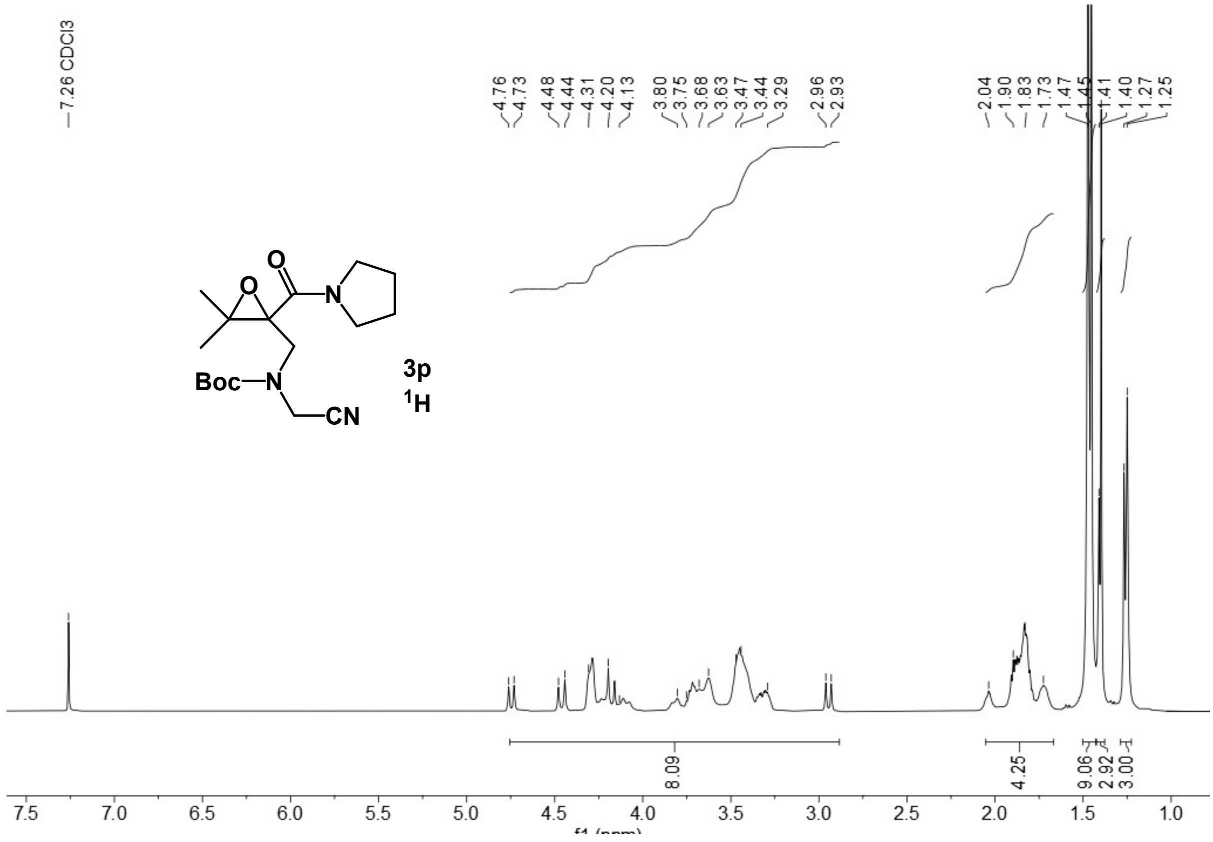

7.26 CDCl3
4.76
4.73
4.48
4.44
4.31
4.20
4.13
3.80
3.75
3.68
3.63
3.47
3.44
3.29
2.96
2.93
2.04
1.90
1.83
1.73
1.47
1.45
1.41
1.40
1.27
1.25
O
O
N
Boc
N
CN
3p
1H
8.09
4.25
9.06
2.92
3.00
f1 (ppm)

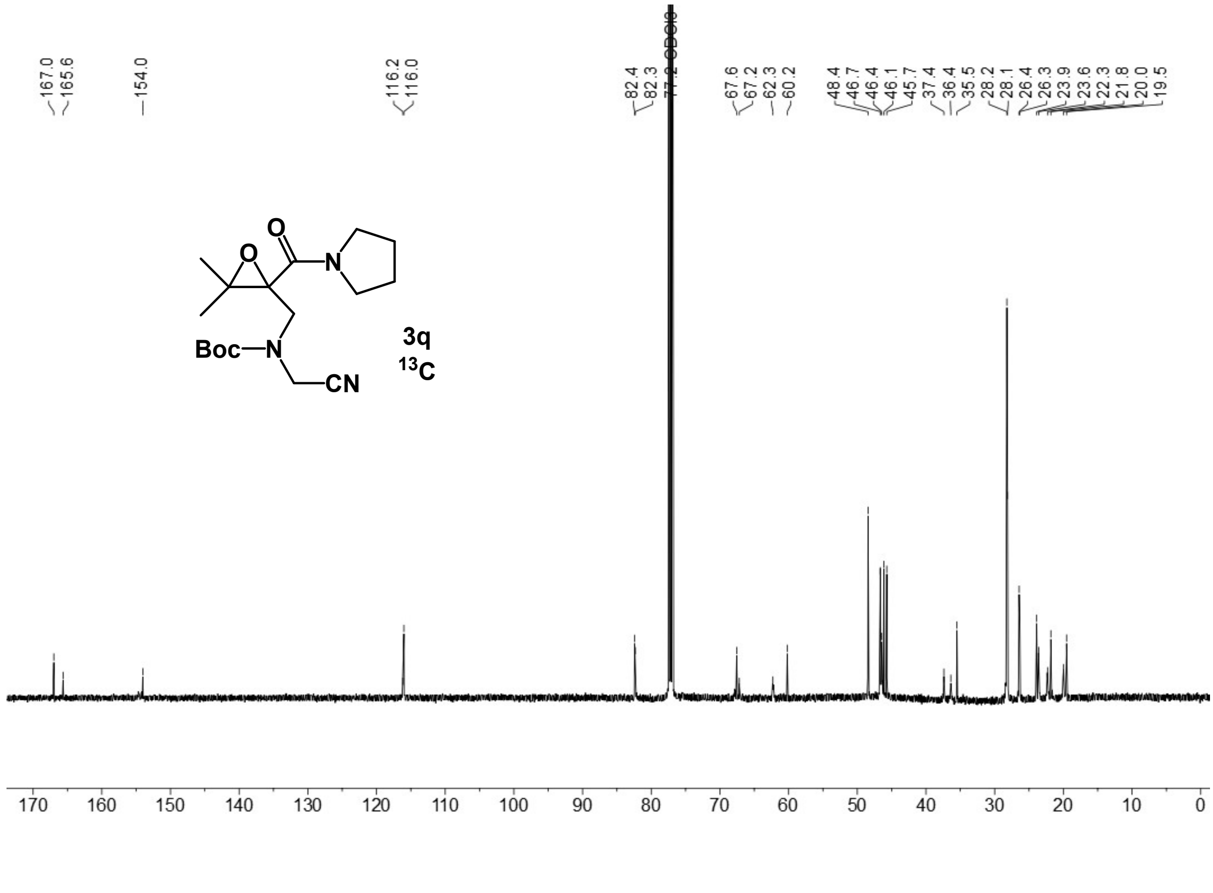

167.0
165.6
154.0
116.2
116.0
82.4
82.3
77.2 CDCl3
67.6
67.2
62.3
60.2
48.4
46.7
46.4
46.1
45.7
37.4
36.4
35.5
28.2
28.1
26.4
26.3
23.9
23.6
22.3
21.8
20.0
19.5
O
O
N
Boc
N
CN
3q
13C

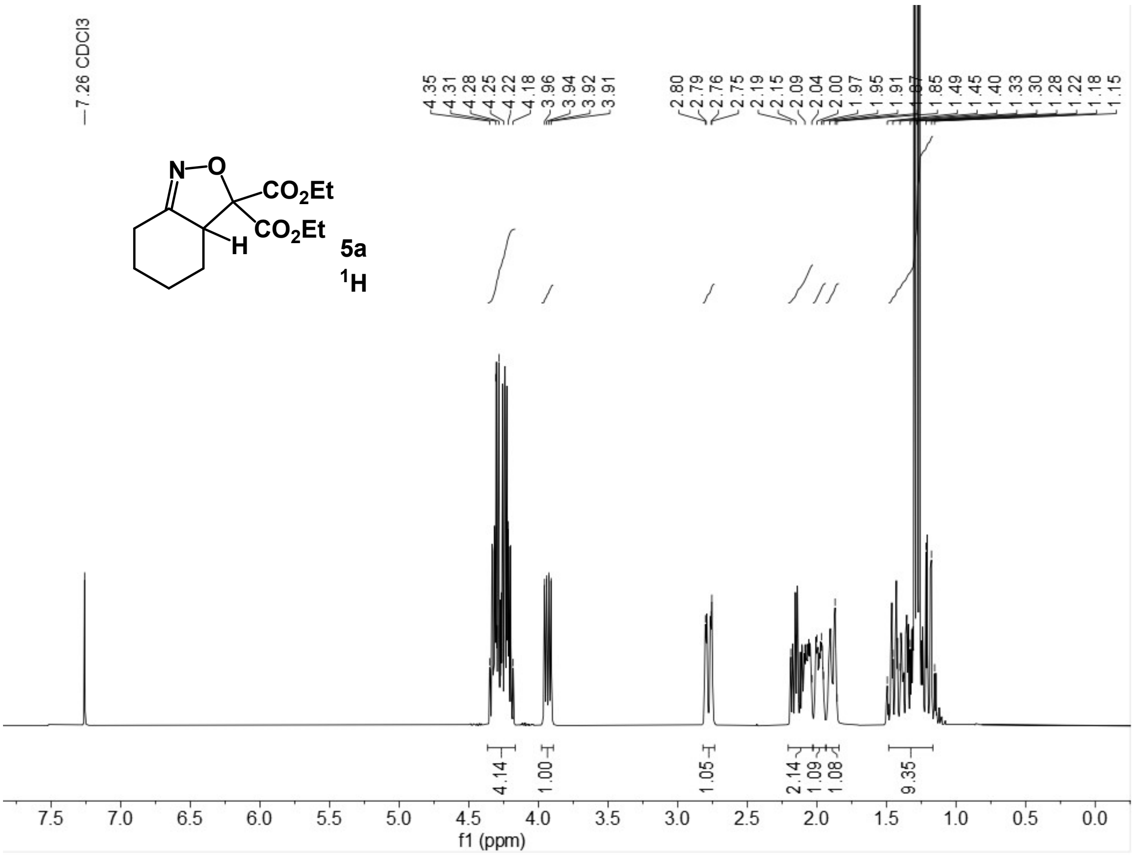
7.26 CDCl3
4.35 4.31 4.28 4.25 4.22 4.18 3.96 3.94 3.92 3.91
2.80 2.79 2.76 2.75 2.19 2.15 2.09 2.04 2.00 1.97 1.95 1.91 1.87 1.85 1.49 1.45 1.40 1.33 1.30 1.28 1.22 1.18 1.15
N–O
CO_2Et
CO_2Et
H
5a
^1H
4.14
1.00
1.05
2.14
1.09
1.08
9.35
7.5 7.0 6.5 6.0 5.5 5.0 4.5 4.0 3.5 3.0 2.5 2.0 1.5 1.0 0.5 0.0
f1 (ppm)

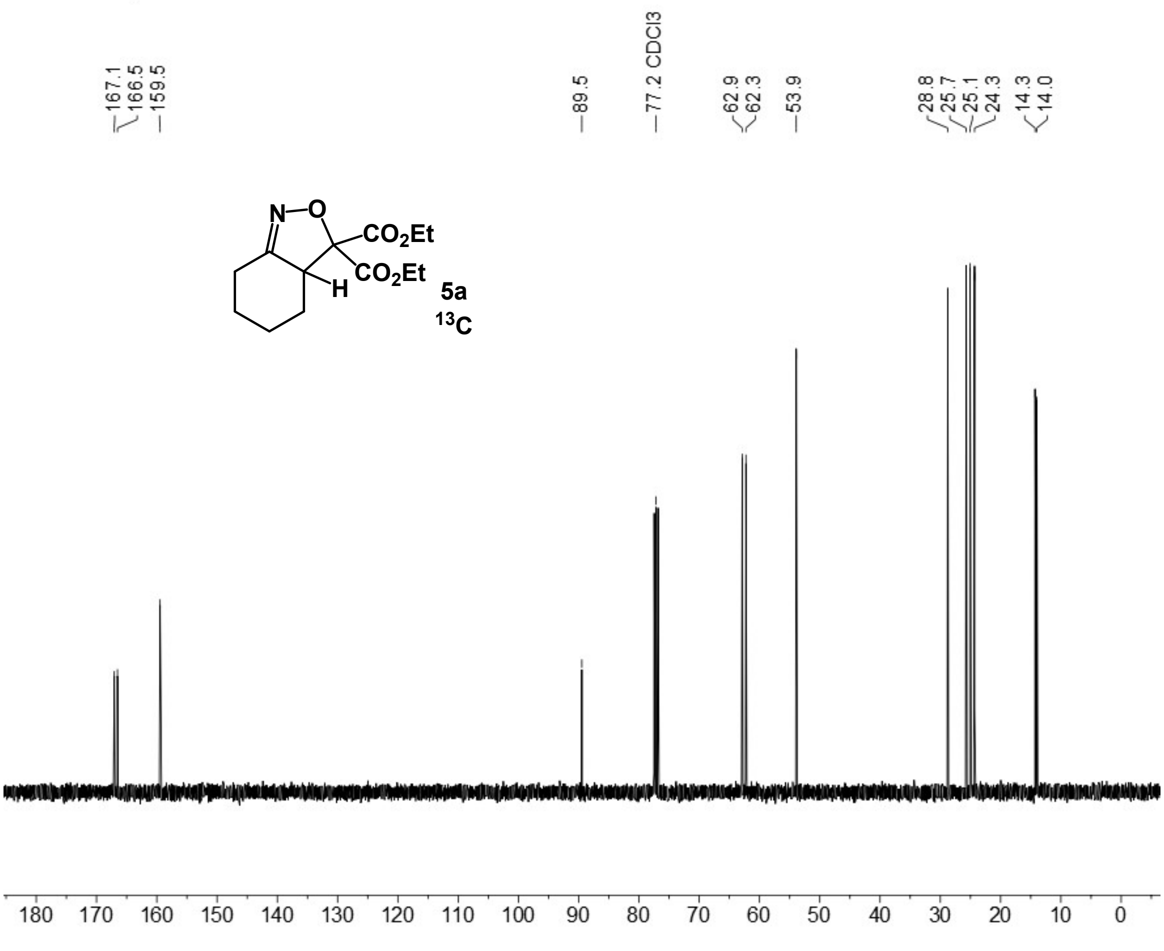
167.1 166.5 159.5
89.5
77.2 CDCl3
62.9 62.3
53.9
28.8 25.7 25.1 24.3
14.3 14.0
N–O
CO_2Et
CO_2Et
H
5a
^{13}C
180 170 160 150 140 130 120 110 100 90 80 70 60 50 40 30 20 10 0

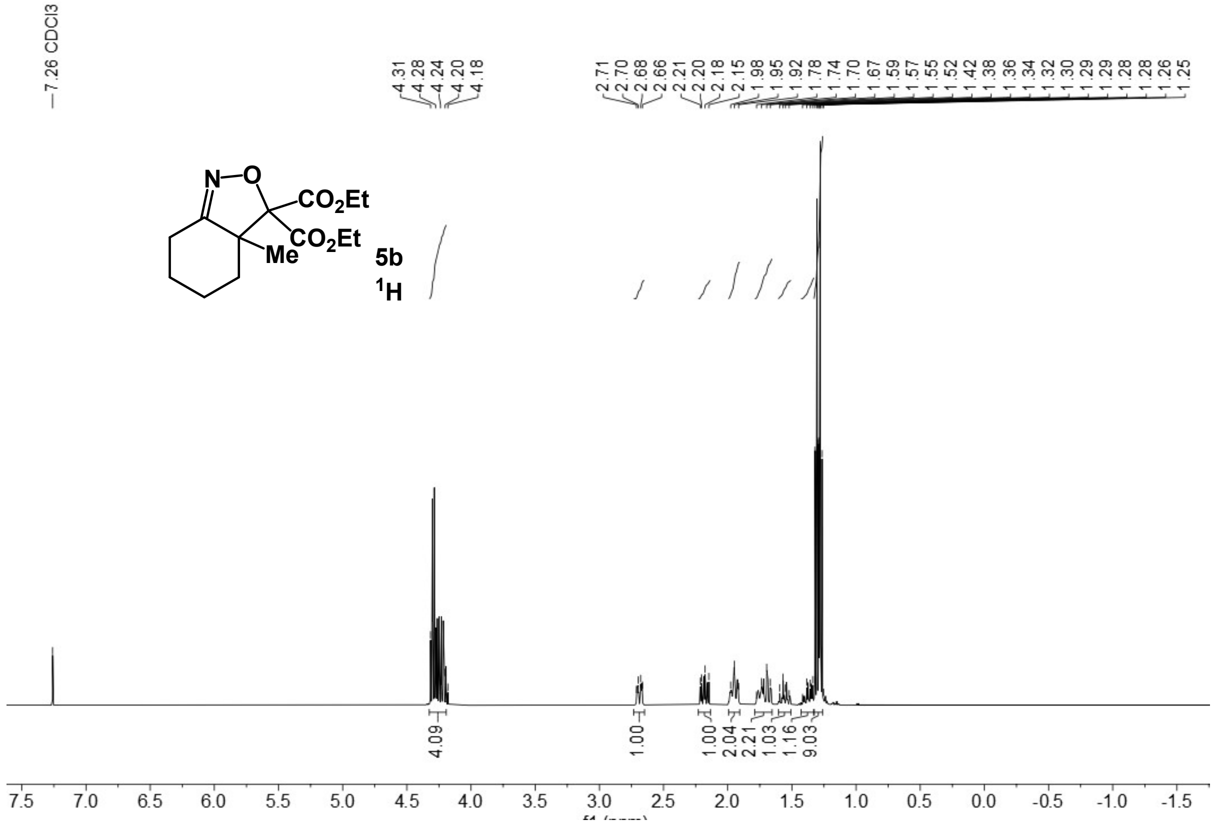
7.26 CDCl3
4.31 4.28 4.24 4.20 4.18
2.71 2.70 2.68 2.66 2.21 2.20 2.18 2.15 1.98 1.95 1.92 1.78 1.74 1.70 1.67 1.59 1.57 1.55 1.52 1.42 1.38 1.36 1.34 1.32 1.30 1.29 1.29 1.28 1.28 1.26 1.25
N–O
CO2Et
CO2Et
Me
5b
1H
4.09
1.00
1.00 2.04 2.21 1.03 1.16 9.03
7.5 7.0 6.5 6.0 5.5 5.0 4.5 4.0 3.5 3.0 2.5 2.0 1.5 1.0 0.5 0.0 -0.5 -1.0 -1.5

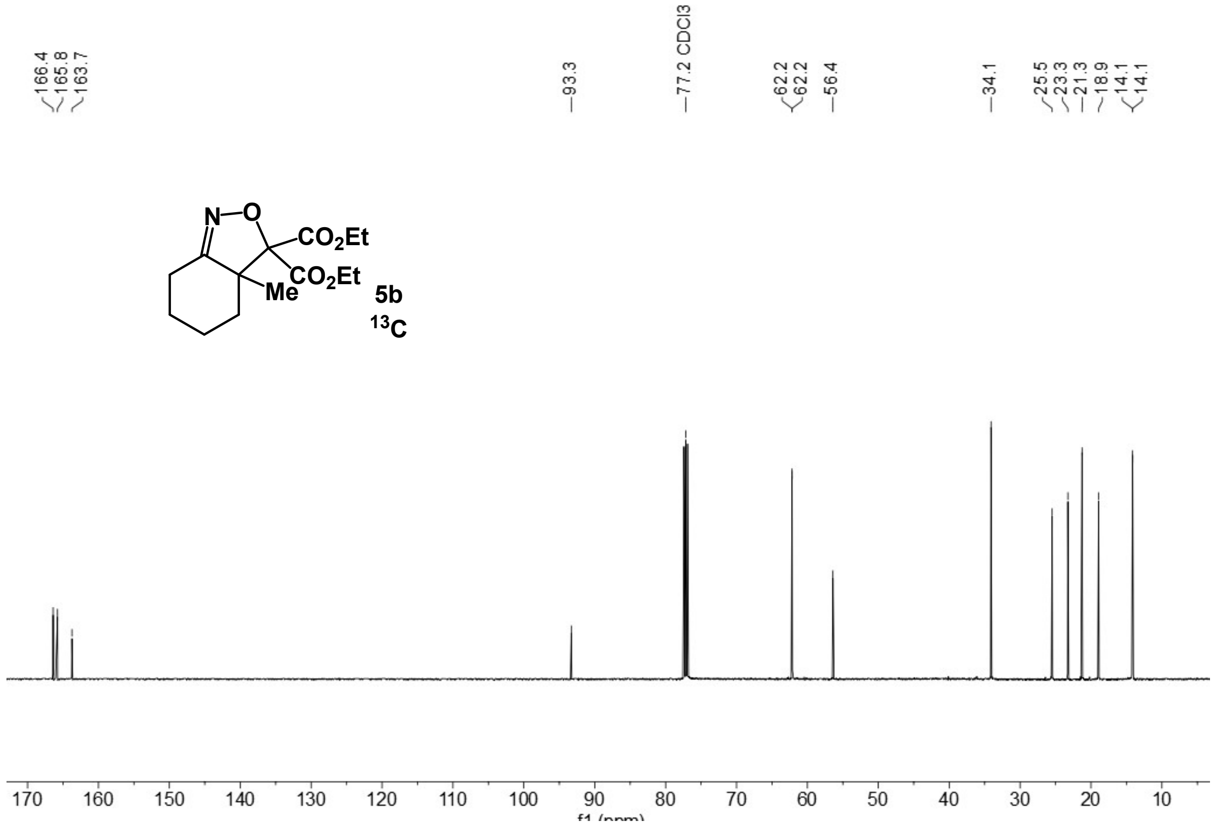
166.4 165.8 163.7
93.3
77.2 CDCl3
62.2 62.2 56.4
34.1
25.5 23.3 21.3 18.9 14.1 14.1
N–O
CO2Et
CO2Et
Me
5b
13C
170 160 150 140 130 120 110 100 90 80 70 60 50 40 30 20 10

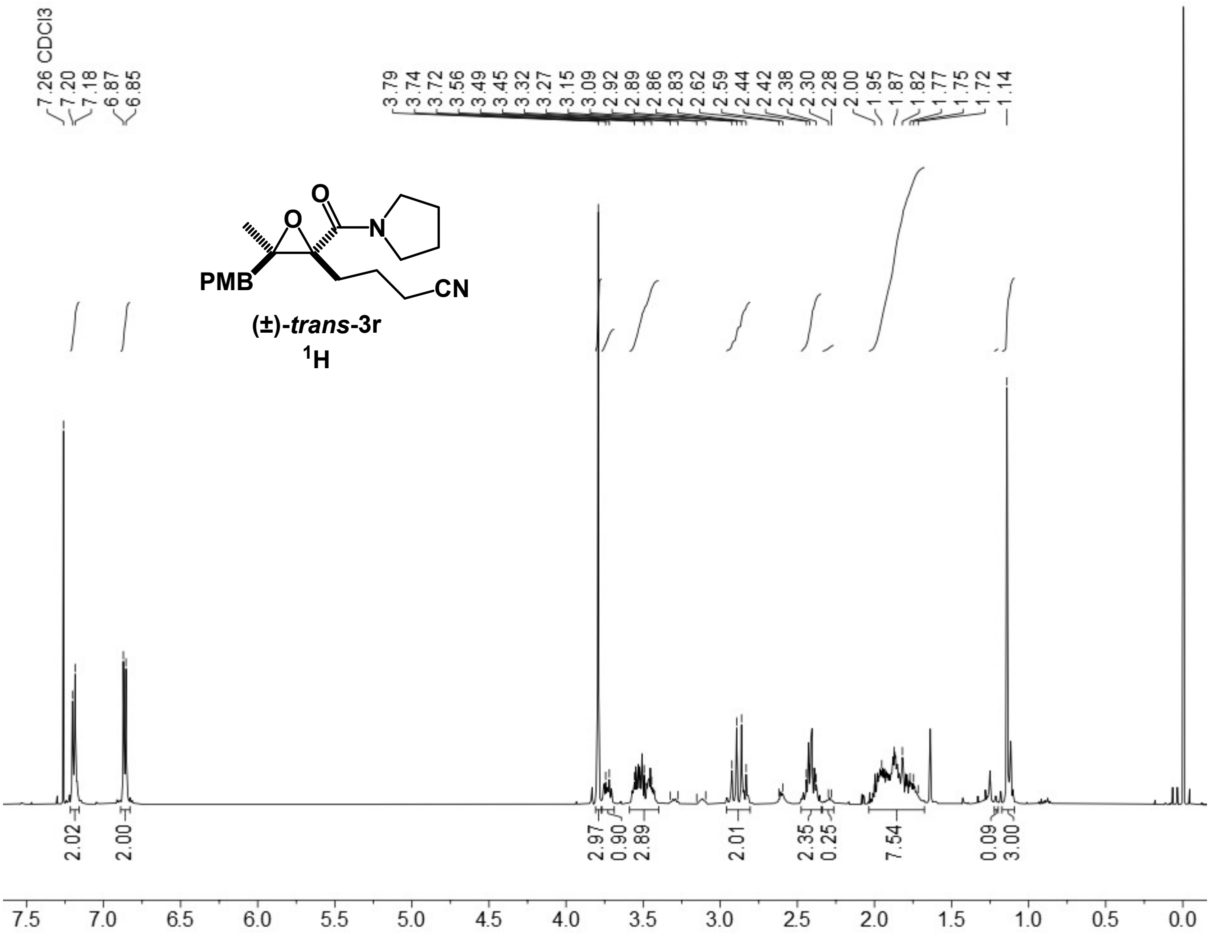

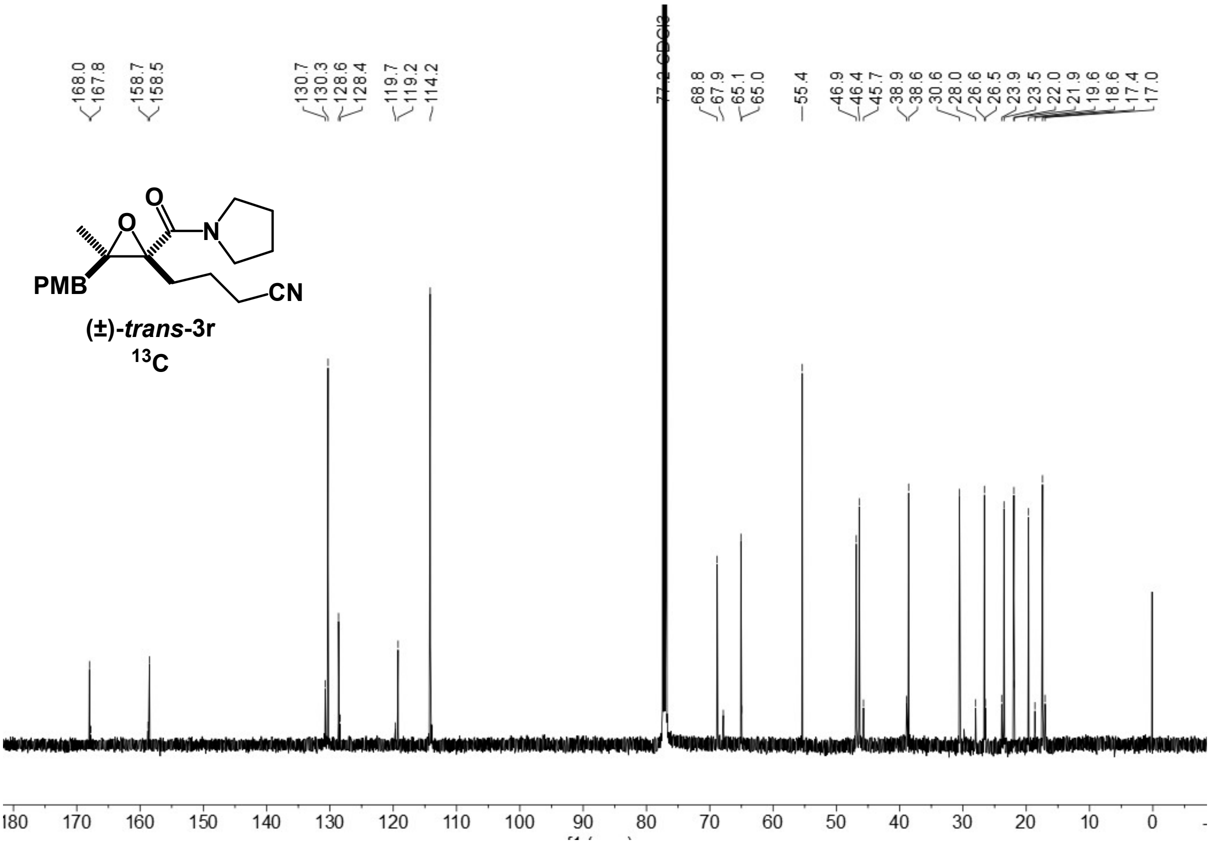

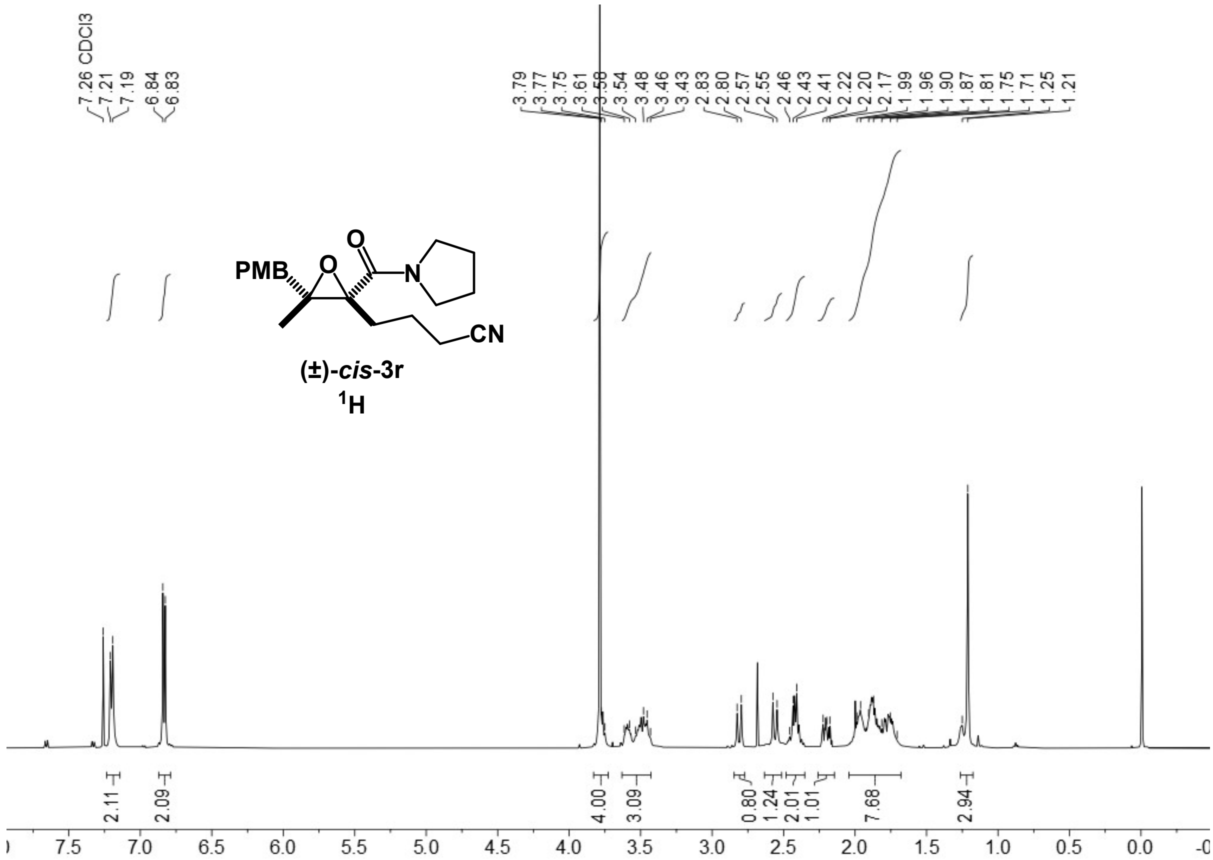
7.26 CDCl3
7.21
7.19
6.84
6.83
3.79
3.77
3.75
3.61
3.58
3.54
3.48
3.46
3.43
2.83
2.80
2.57
2.55
2.46
2.43
2.41
2.22
2.20
2.17
1.99
1.96
1.90
1.87
1.81
1.75
1.71
1.25
1.21
PMB
O
O
N
CN
(±)-cis-3r
1H
2.11
2.09
4.00
3.09
0.80
1.24
2.01
1.01
7.68
2.94

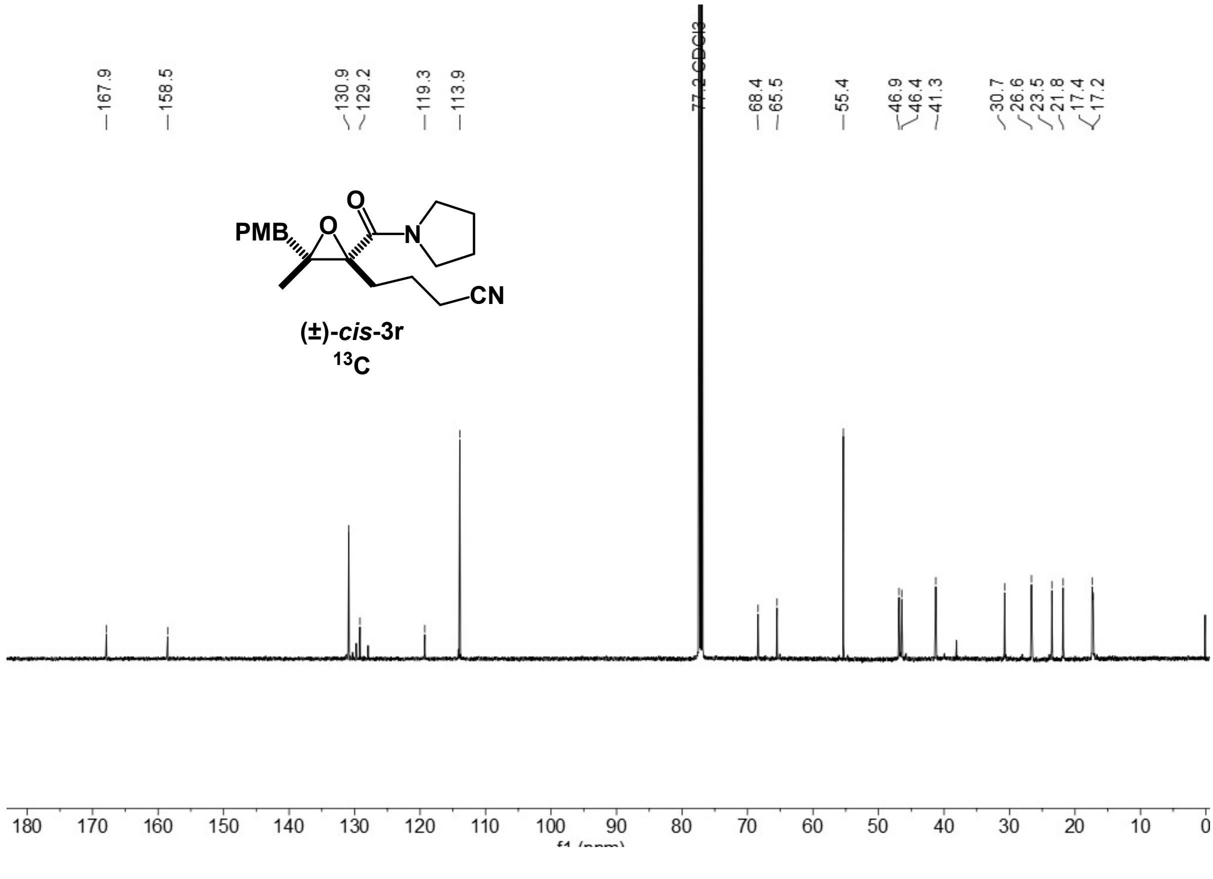
167.9
158.5
130.9
129.2
119.3
113.9
77.2 CDCl3
68.4
65.5
55.4
46.9
46.4
41.3
30.7
26.6
23.5
21.8
17.4
17.2
PMB
O
O
N
CN
(±)-cis-3r
13C
f1 (ppm)

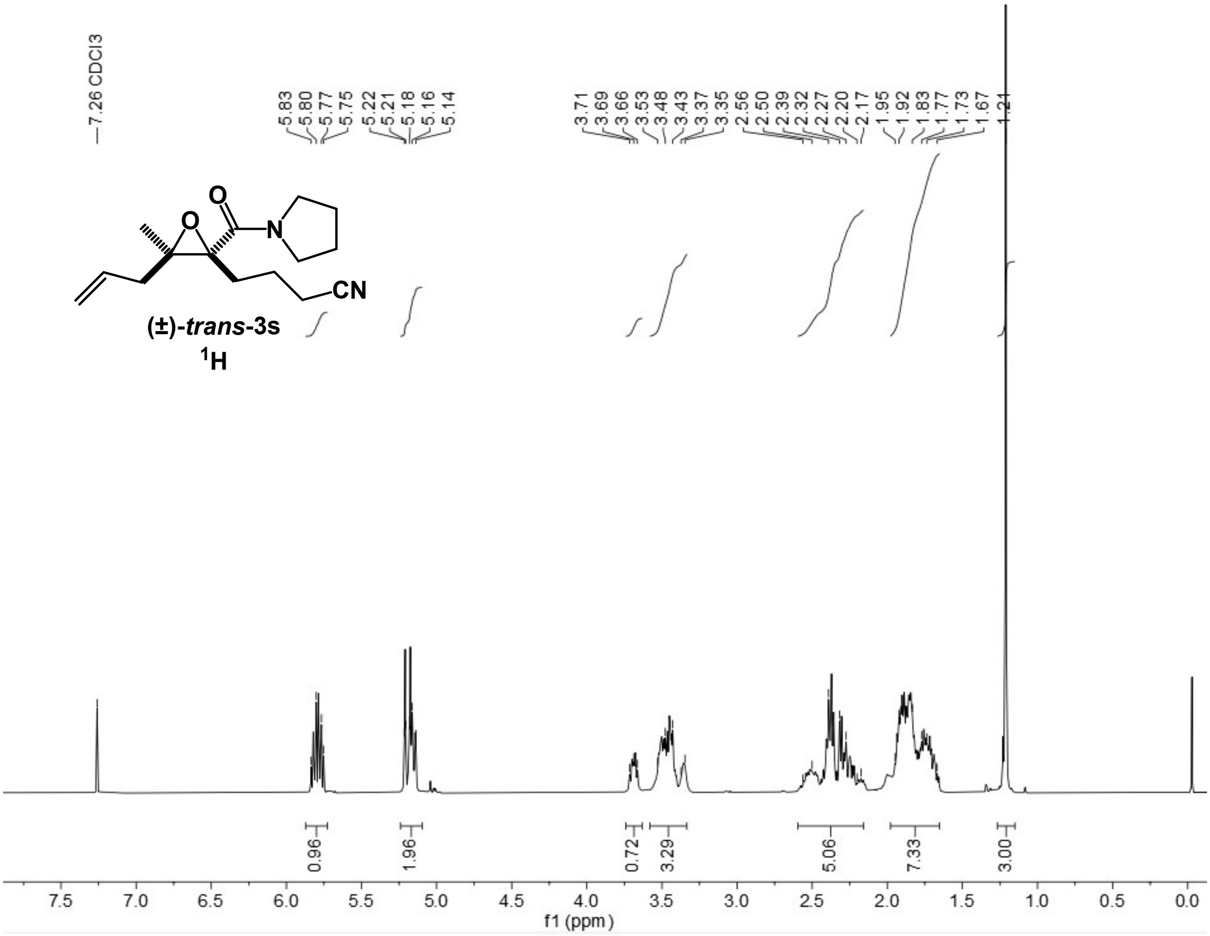
7.26 CDCl3
5.83
5.80
5.77
5.75
5.22
5.21
5.18
5.16
5.14
3.71
3.69
3.66
3.53
3.48
3.43
3.37
3.35
2.56
2.50
2.39
2.32
2.27
2.20
2.17
1.95
1.92
1.83
1.77
1.73
1.67
1.21
O
N
CN
(±)-trans-3s
1H
0.96
1.96
0.72
3.29
5.06
7.33
3.00
f1 (ppm)

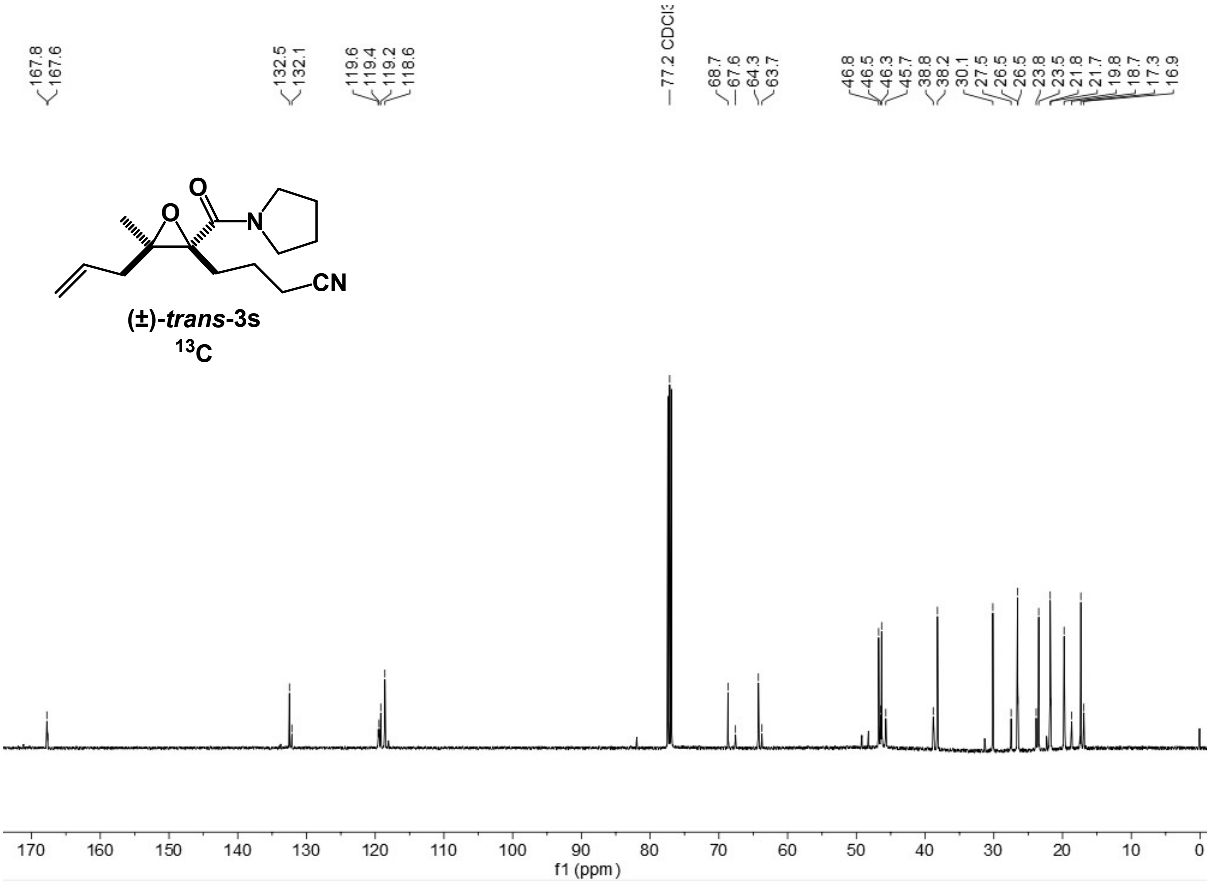
167.8
167.6
132.5
132.1
119.6
119.4
119.2
118.6
77.2 CDCl3
68.7
67.6
64.3
63.7
46.8
46.5
46.3
45.7
38.8
38.2
30.1
27.5
26.5
26.5
23.8
23.5
21.8
21.7
19.8
18.7
17.3
16.9
O
N
CN
(±)-trans-3s
13C
f1 (ppm)

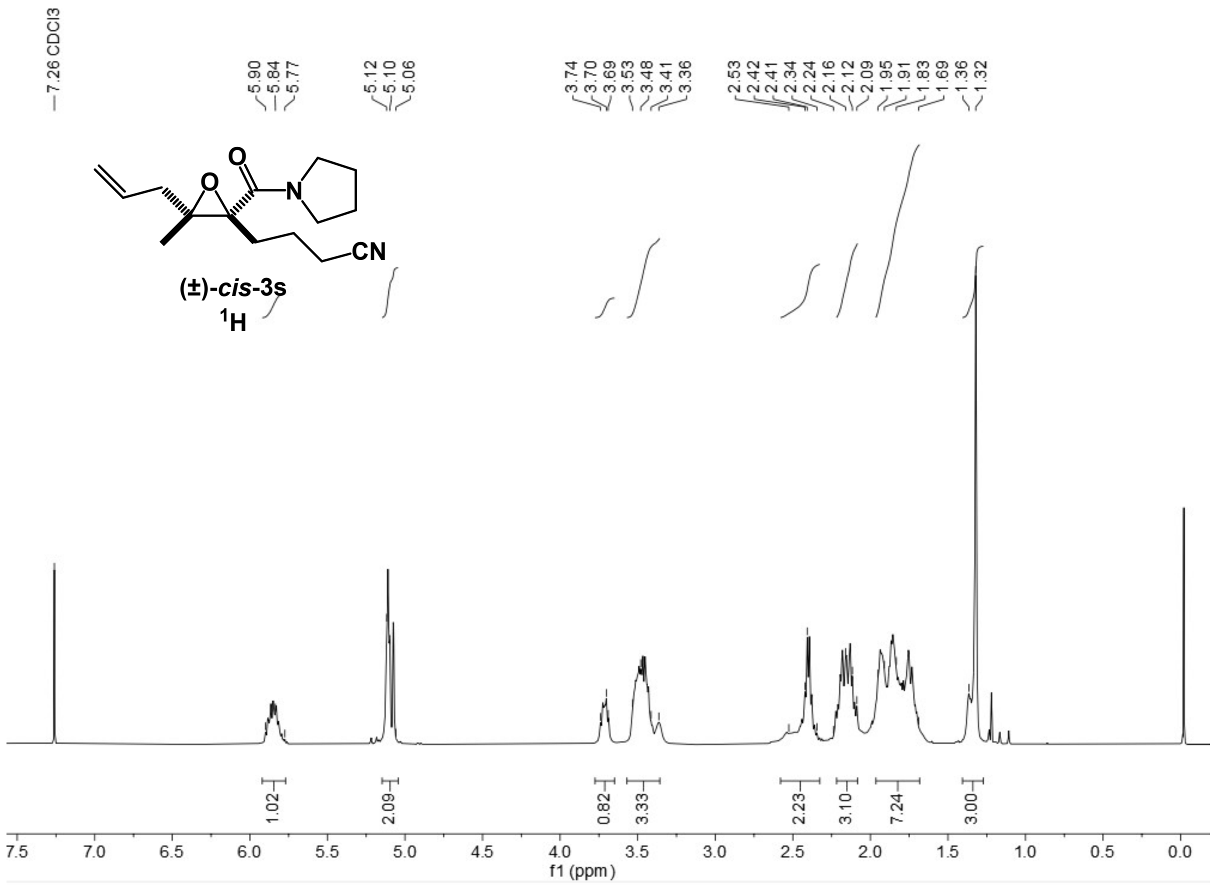

7.26 CDCl3
5.90
5.84
5.77
5.12
5.10
5.06
3.74
3.70
3.69
3.53
3.48
3.41
3.36
2.53
2.42
2.41
2.34
2.24
2.16
2.12
2.09
1.95
1.91
1.83
1.69
1.36
1.32
O
N
O
CN
(±)-cis-3s
1H
1.02
2.09
0.82
3.33
2.23
3.10
7.24
3.00
7.5
7.0
6.5
6.0
5.5
5.0
4.5
4.0
3.5
3.0
2.5
2.0
1.5
1.0
0.5
0.0
f1 (ppm)

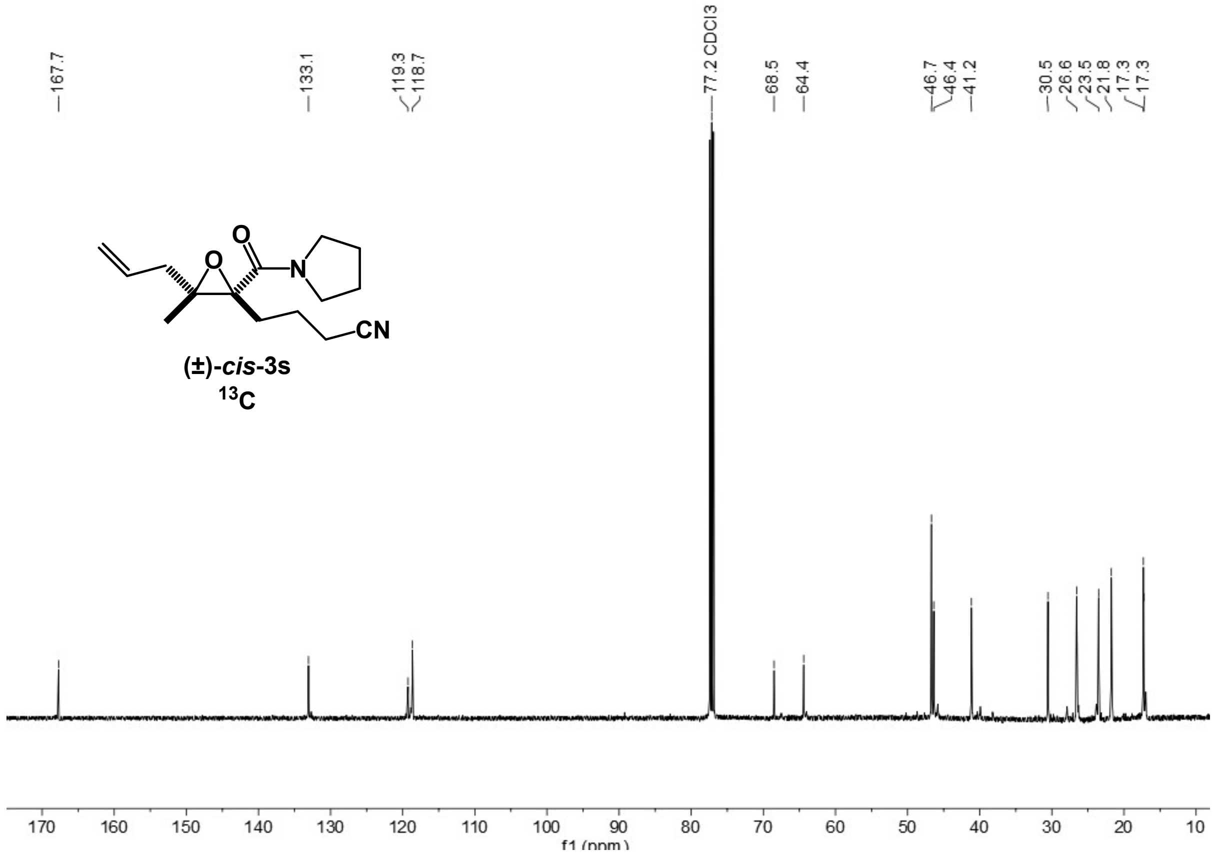

167.7
133.1
119.3
118.7
77.2 CDCl3
68.5
64.4
46.7
46.4
41.2
30.5
26.6
23.5
21.8
17.3
17.3
O
N
O
CN
(±)-cis-3s
13C
170
160
150
140
130
120
110
100
90
80
70
60
50
40
30
20
10

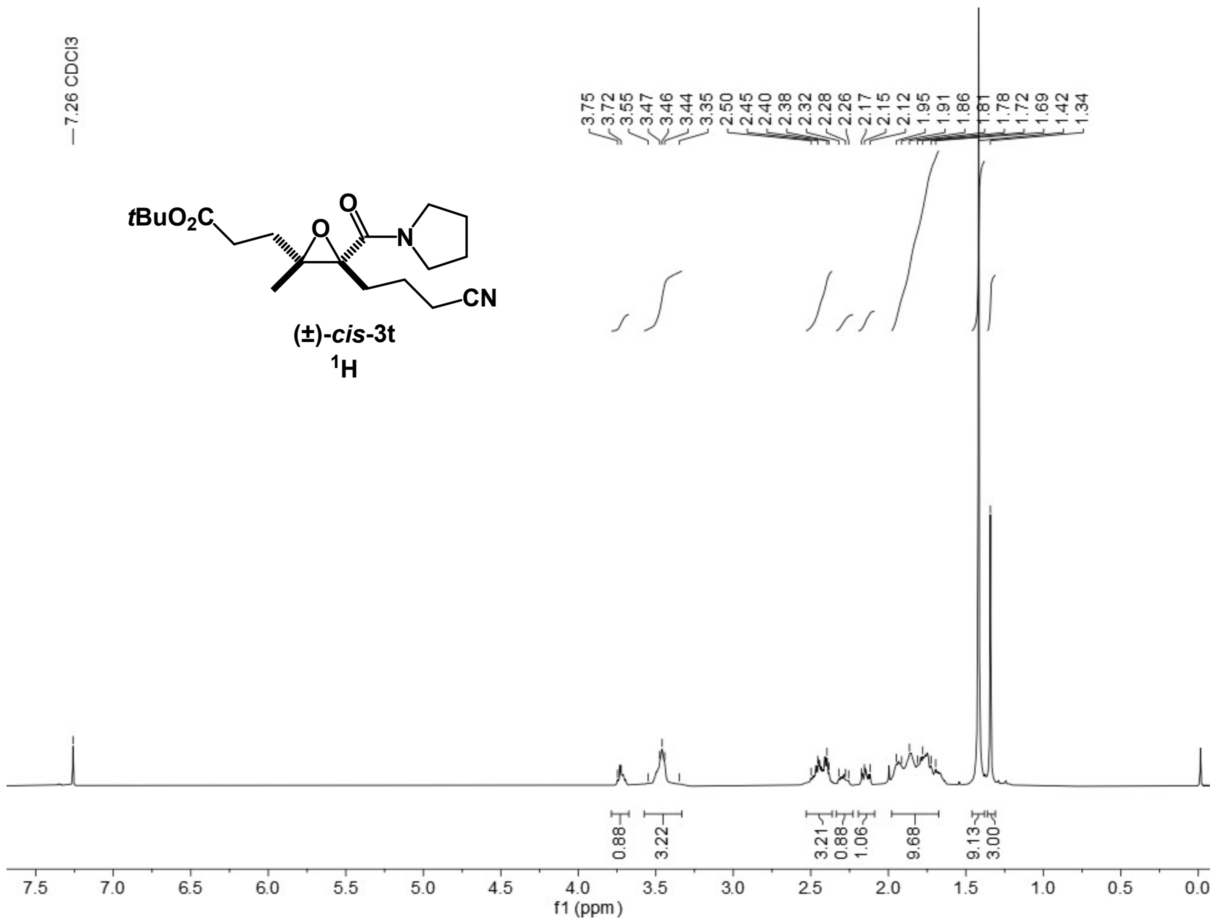
7.26 CDCl3
3.75
3.72
3.55
3.47
3.46
3.44
3.35
2.50
2.45
2.40
2.38
2.32
2.28
2.26
2.17
2.15
2.12
1.95
1.91
1.86
1.81
1.78
1.72
1.69
1.42
1.34
tBuO2C
O
O
N
CN
(±)-cis-3t
1H
0.88
3.22
3.21
0.88
1.06
9.68
9.13
3.00
f1 (ppm)

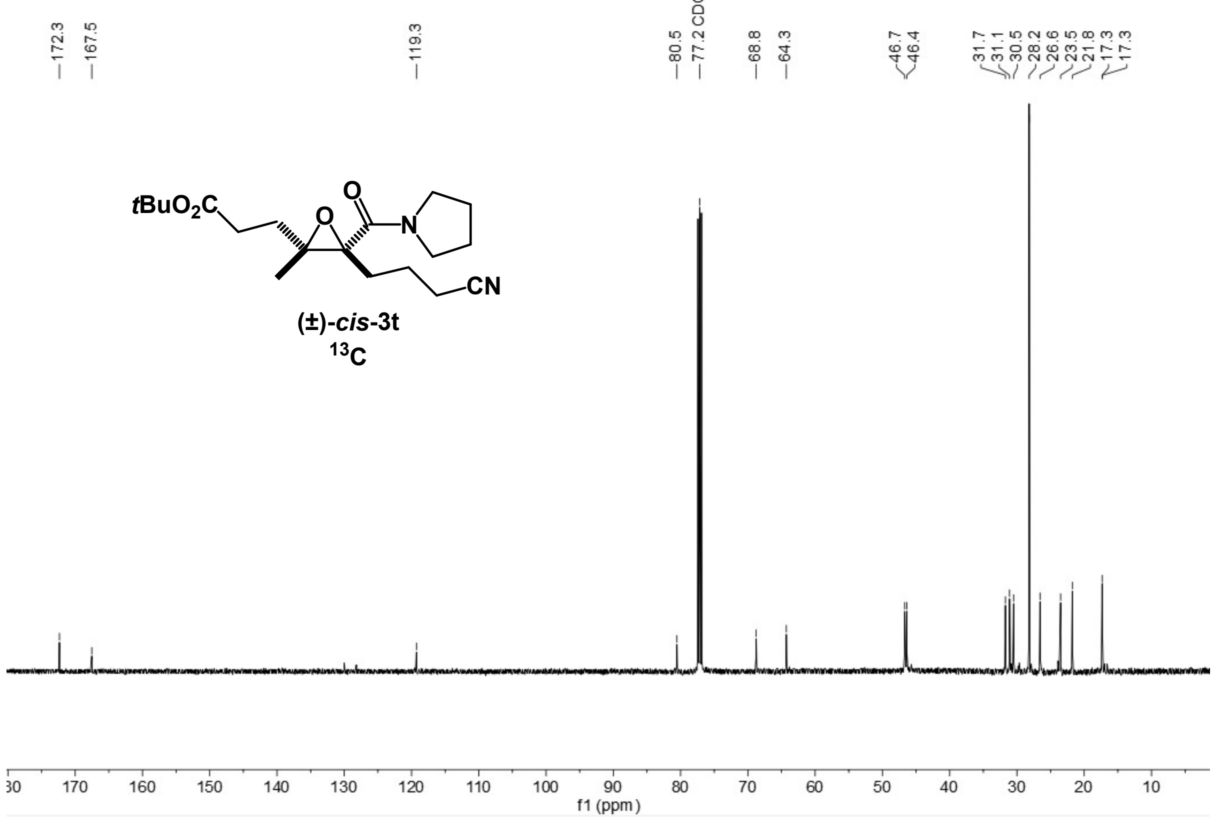
172.3
167.5
119.3
80.5
77.2 CDCl3
68.8
64.3
46.7
46.4
31.7
31.1
30.5
28.2
26.6
23.5
21.8
17.3
17.3
tBuO2C
O
O
N
CN
(±)-cis-3t
13C
f1 (ppm)

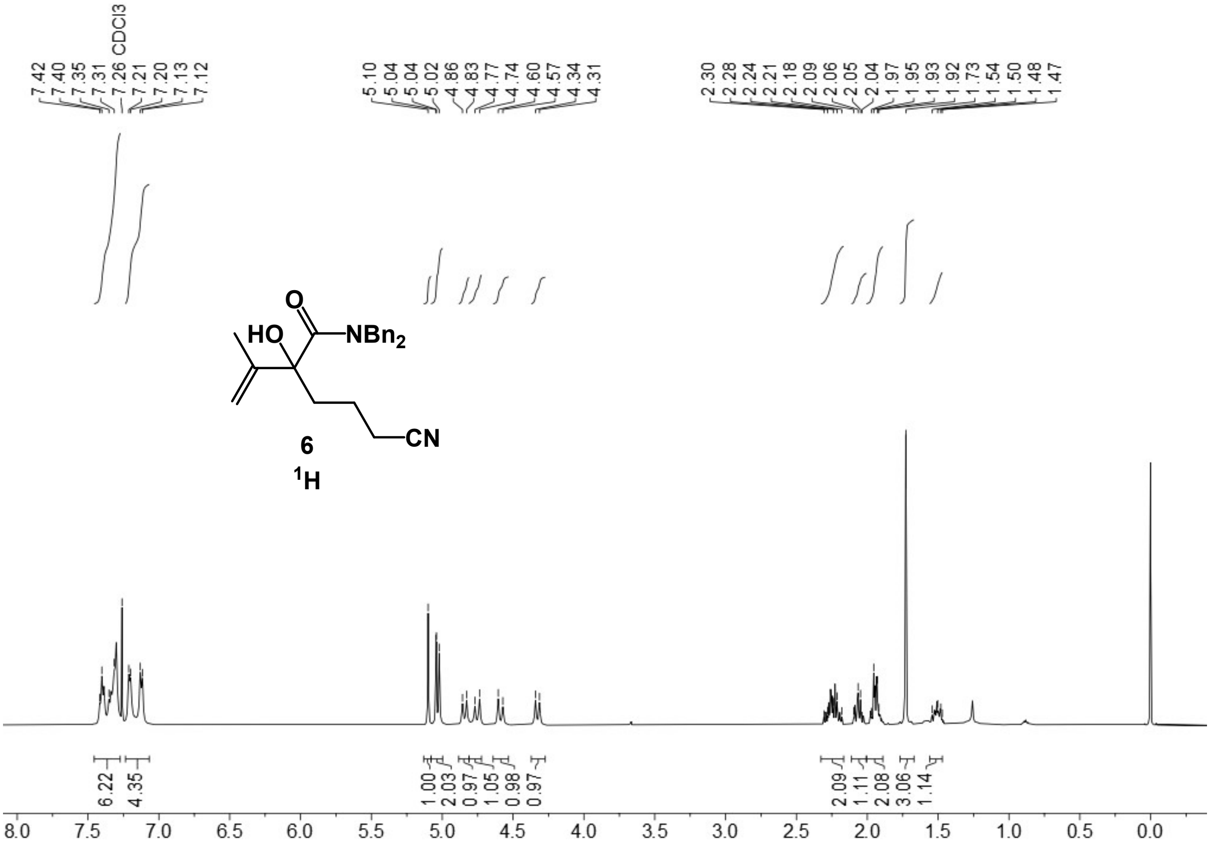
7.42
7.40
7.35
7.31
7.26 CDCl3
7.21
7.20
7.13
7.12
5.10
5.04
5.04
5.02
4.86
4.83
4.77
4.74
4.60
4.57
4.34
4.31
2.30
2.28
2.24
2.21
2.18
2.09
2.06
2.05
2.04
1.97
1.95
1.93
1.92
1.73
1.54
1.50
1.48
1.47
HO
O
NBn2
CN
6
1H
6.22
4.35
1.00
2.03
0.97
1.05
0.98
0.97
2.09
1.11
2.08
3.06
1.14
8.0
7.5
7.0
6.5
6.0
5.5
5.0
4.5
4.0
3.5
3.0
2.5
2.0
1.5
1.0
0.5
0.0

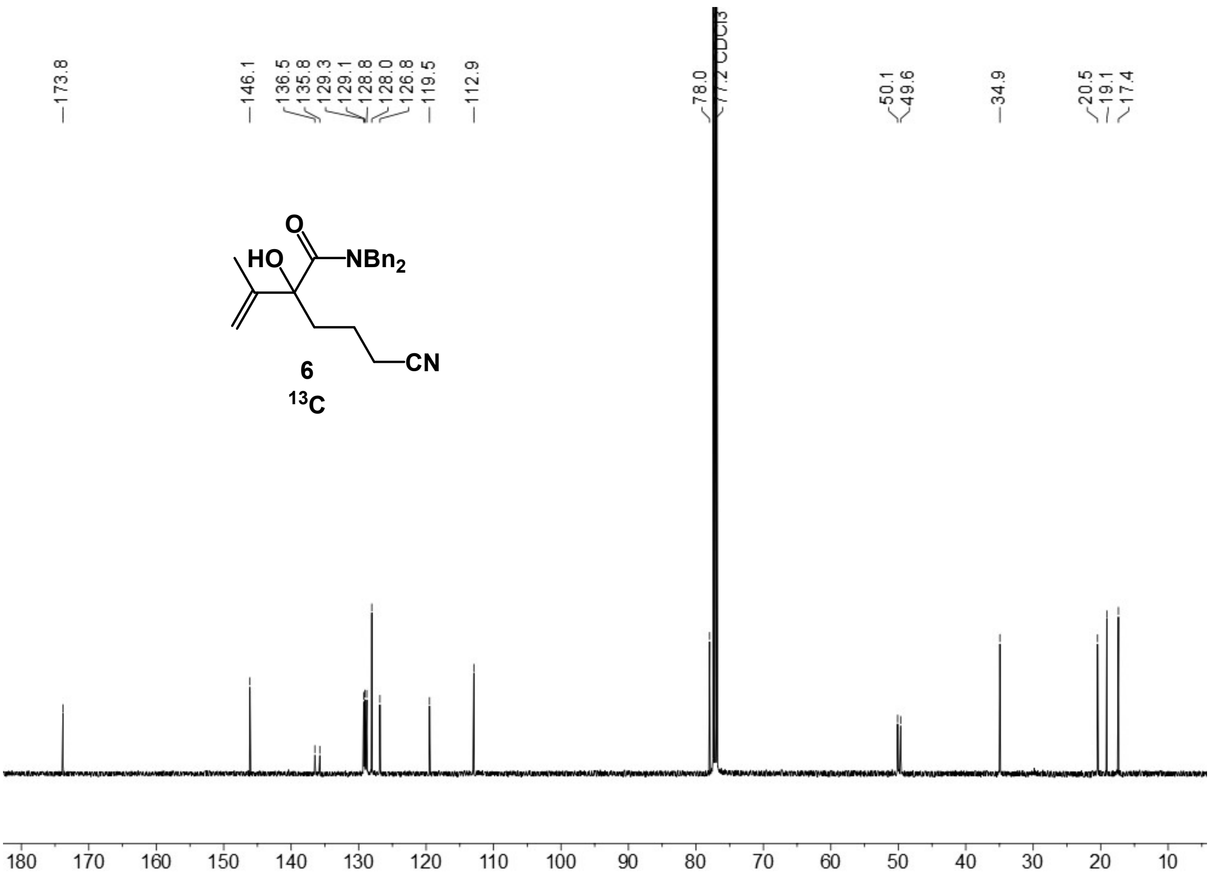
173.8
146.1
136.5
135.8
129.3
129.1
128.8
128.0
126.8
119.5
112.9
78.0
77.2 CDCl3
50.1
49.6
34.9
20.5
19.1
17.4
HO
O
NBn2
CN
6
13C
180
170
160
150
140
130
120
110
100
90
80
70
60
50
40
30
20
10

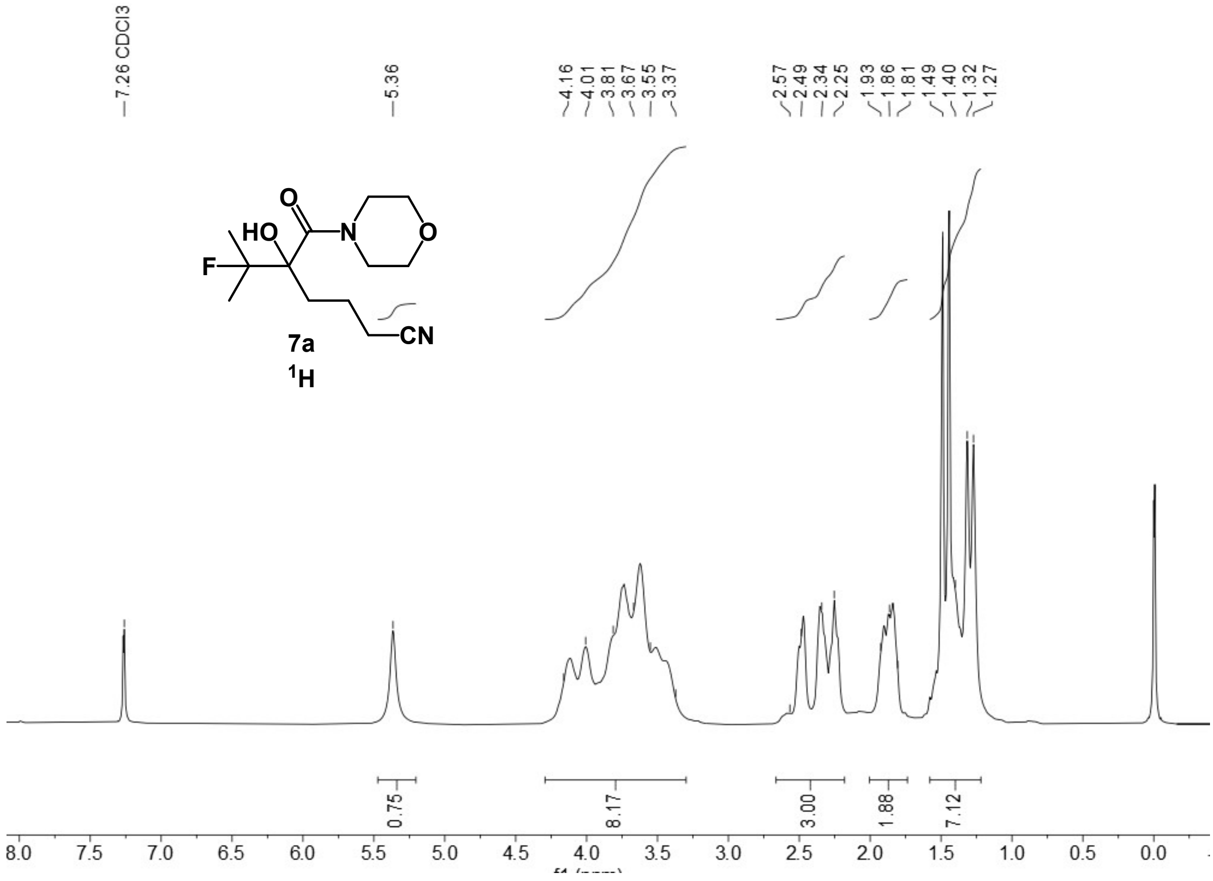
7.26 CDCl3
5.36
4.16
4.01
3.81
3.67
3.55
3.37
2.57
2.49
2.34
2.25
1.93
1.86
1.81
1.49
1.40
1.32
1.27
HO
O
N
O
F
CN
7a
^{1}H
0.75
8.17
3.00
1.88
7.12
8.0 7.5 7.0 6.5 6.0 5.5 5.0 4.5 4.0 3.5 3.0 2.5 2.0 1.5 1.0 0.5 0.0

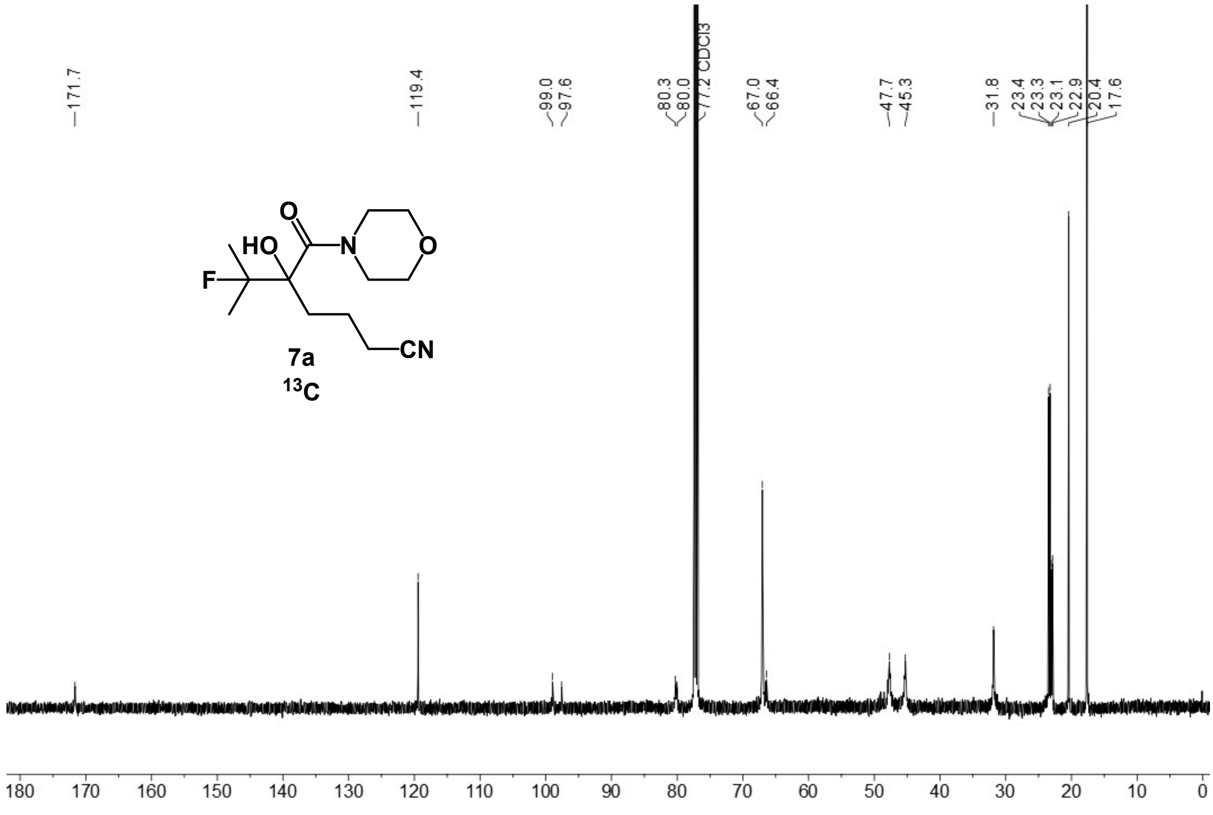
171.7
119.4
99.0
97.6
80.3
80.0
77.2 CDCl3
67.0
66.4
47.7
45.3
31.8
23.4
23.3
23.1
22.9
20.4
17.6
HO
O
N
O
F
CN
7a
^{13}C
180 170 160 150 140 130 120 110 100 90 80 70 60 50 40 30 20 10 0

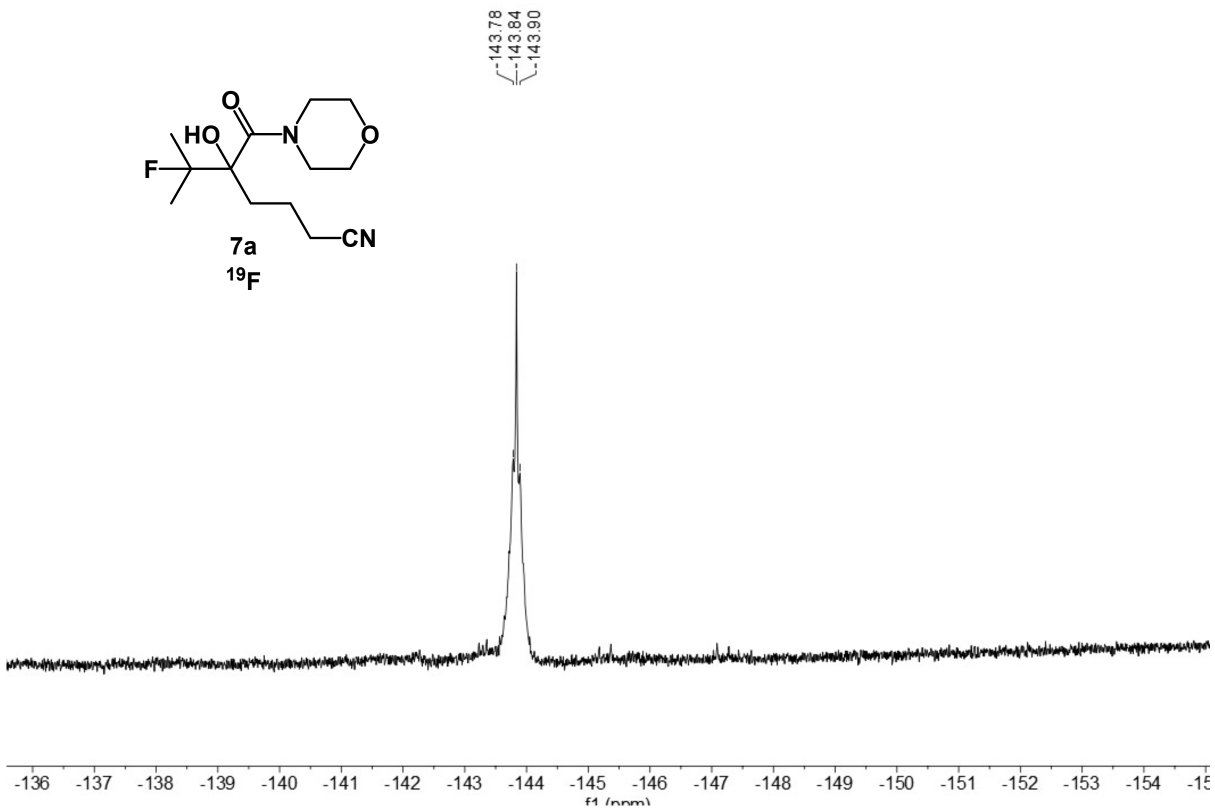

-143.78
-143.84
-143.90
O
HO
N
O
F
CN
7a
19F
-136 -137 -138 -139 -140 -141 -142 -143 -144 -145 -146 -147 -148 -149 -150 -151 -152 -153 -154
[illegible]

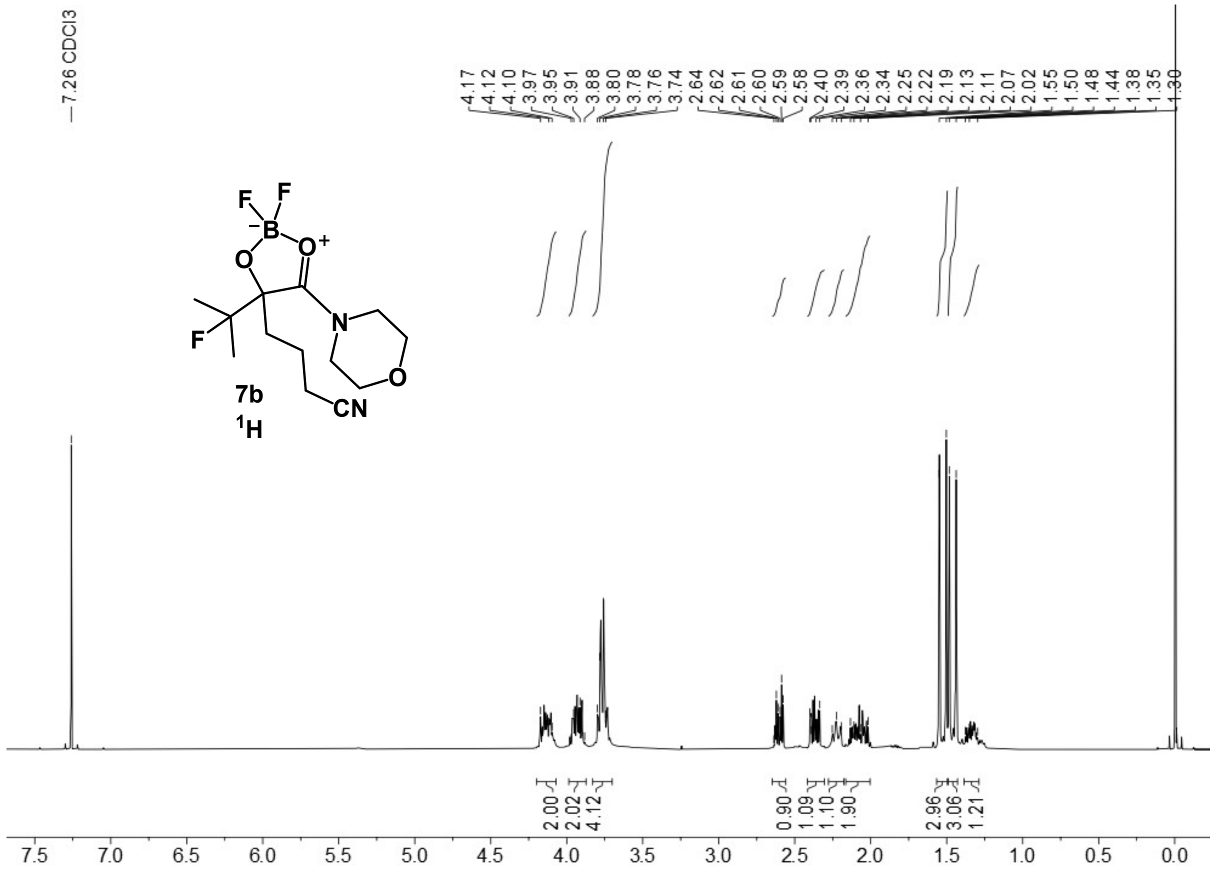
7.26 CDCl3
4.17
4.12
4.10
3.97
3.95
3.91
3.88
3.80
3.78
3.76
3.74
2.64
2.62
2.61
2.60
2.59
2.58
2.40
2.39
2.36
2.34
2.25
2.22
2.19
2.13
2.11
2.07
2.02
1.55
1.50
1.48
1.44
1.38
1.35
1.30
7b
1H
2.00
2.02
4.12
0.90
1.09
1.10
1.90
2.96
3.06
1.21

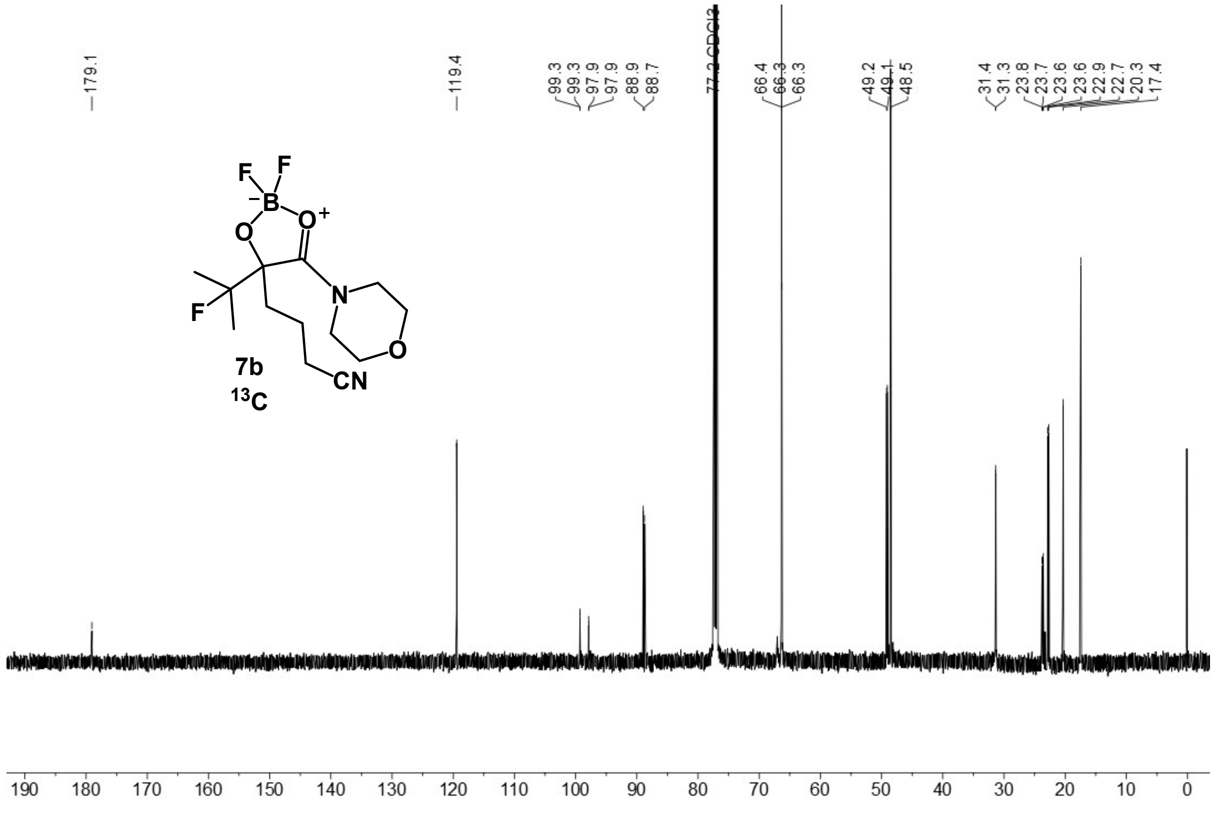
179.1
119.4
99.3
99.3
97.9
97.9
88.9
88.7
77.2 CDCl3
66.4
66.3
66.3
49.2
49.1
48.5
31.4
31.3
23.8
23.7
23.6
23.6
22.9
22.7
20.3
17.4
7b
13C

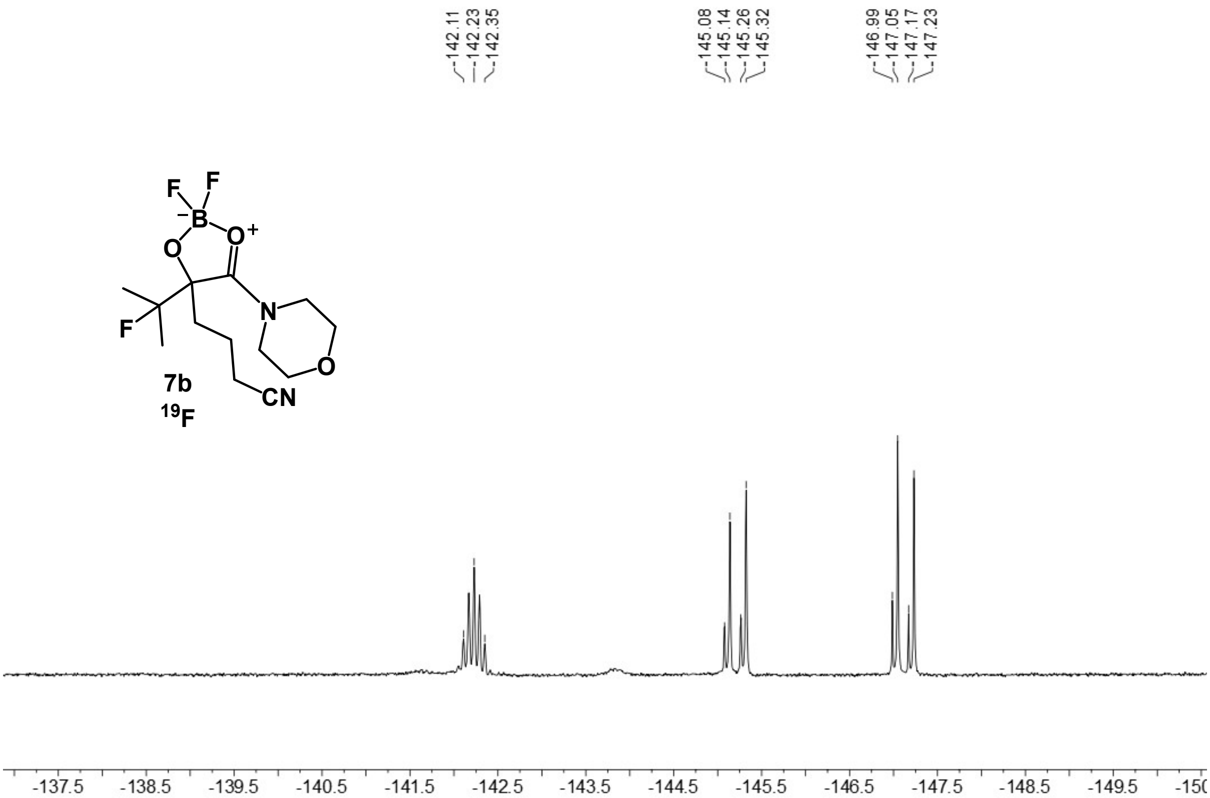

-142.11
-142.23
-142.35
-145.08
-145.14
-145.26
-145.32
-146.99
-147.05
-147.17
-147.23
F
F
B
O
O
F
N
O
CN
-137.5
-138.5
-139.5
-140.5
-141.5
-142.5
-143.5
-144.5
-145.5
-146.5
-147.5
-148.5
-149.5
-15(

7b
^{19}F

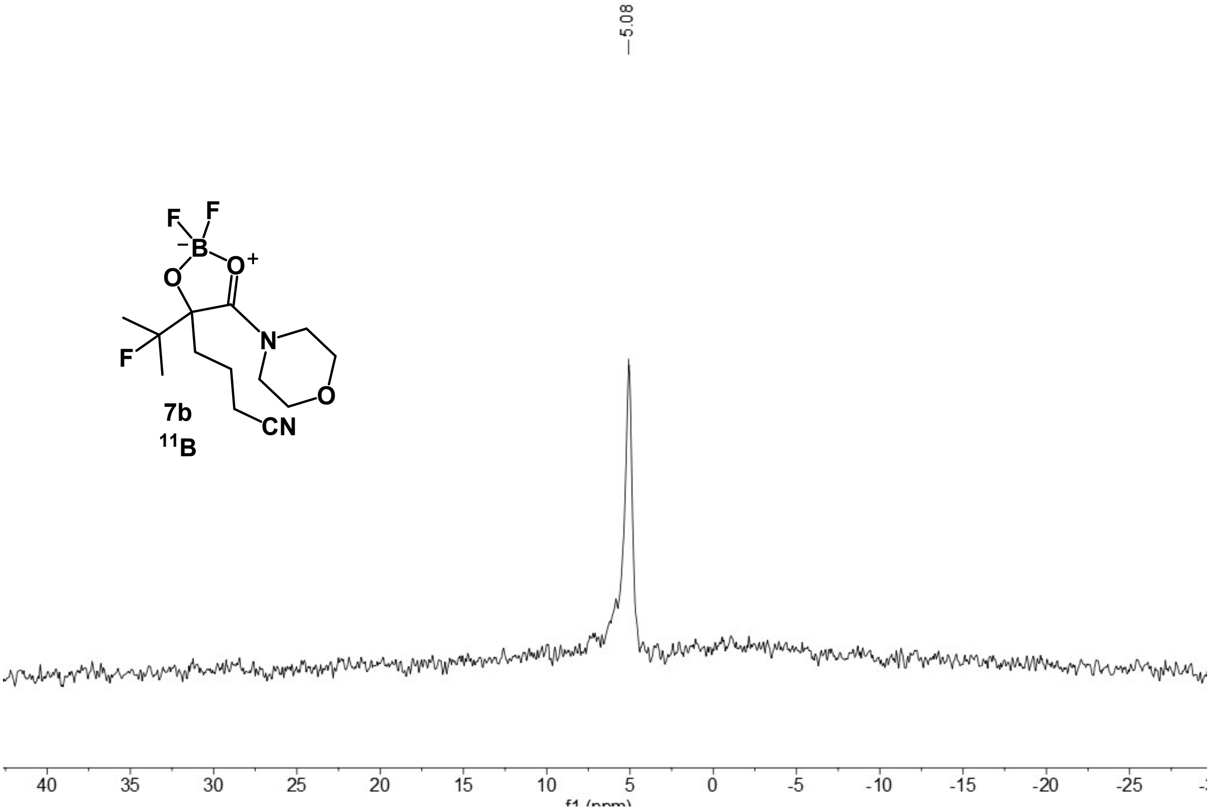

5.08
F
F
B
O
O
F
N
O
CN
40
35
30
25
20
15
10
5
0
-5
-10
-15
-20
-25
f1 (ppm)

7b
^{11}B

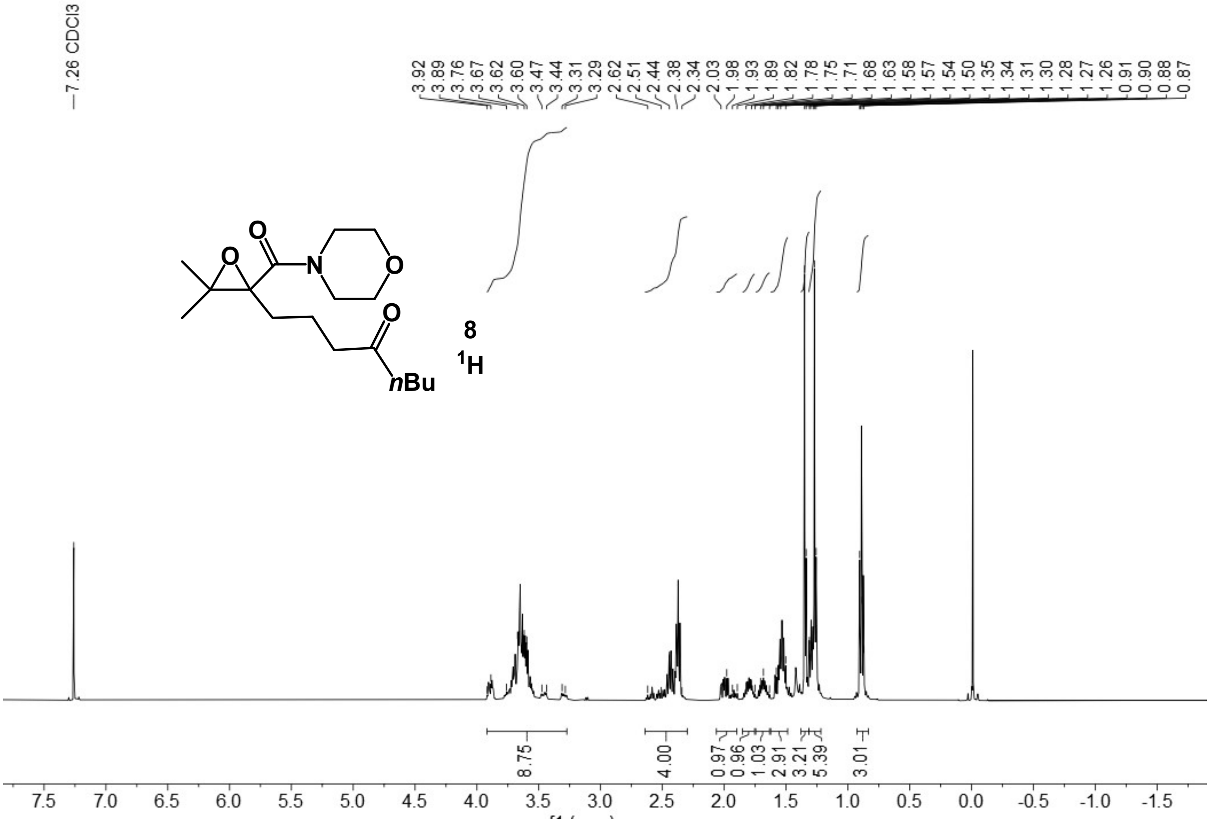
7.26 CDCl3
3.92 3.89 3.76 3.67 3.62 3.60 3.47 3.44 3.31 3.29 2.62 2.51 2.44 2.38 2.34 2.03 1.98 1.93 1.89 1.82 1.78 1.75 1.71 1.68 1.63 1.58 1.57 1.54 1.50 1.35 1.34 1.31 1.30 1.28 1.27 1.26 0.91 0.90 0.88 0.87
O
N
O
O
O
nBu
8
1H
8.75
4.00
0.97 0.96 1.03 2.91 3.21 5.39
3.01
7.5 7.0 6.5 6.0 5.5 5.0 4.5 4.0 3.5 3.0 2.5 2.0 1.5 1.0 0.5 0.0 -0.5 -1.0 -1.5

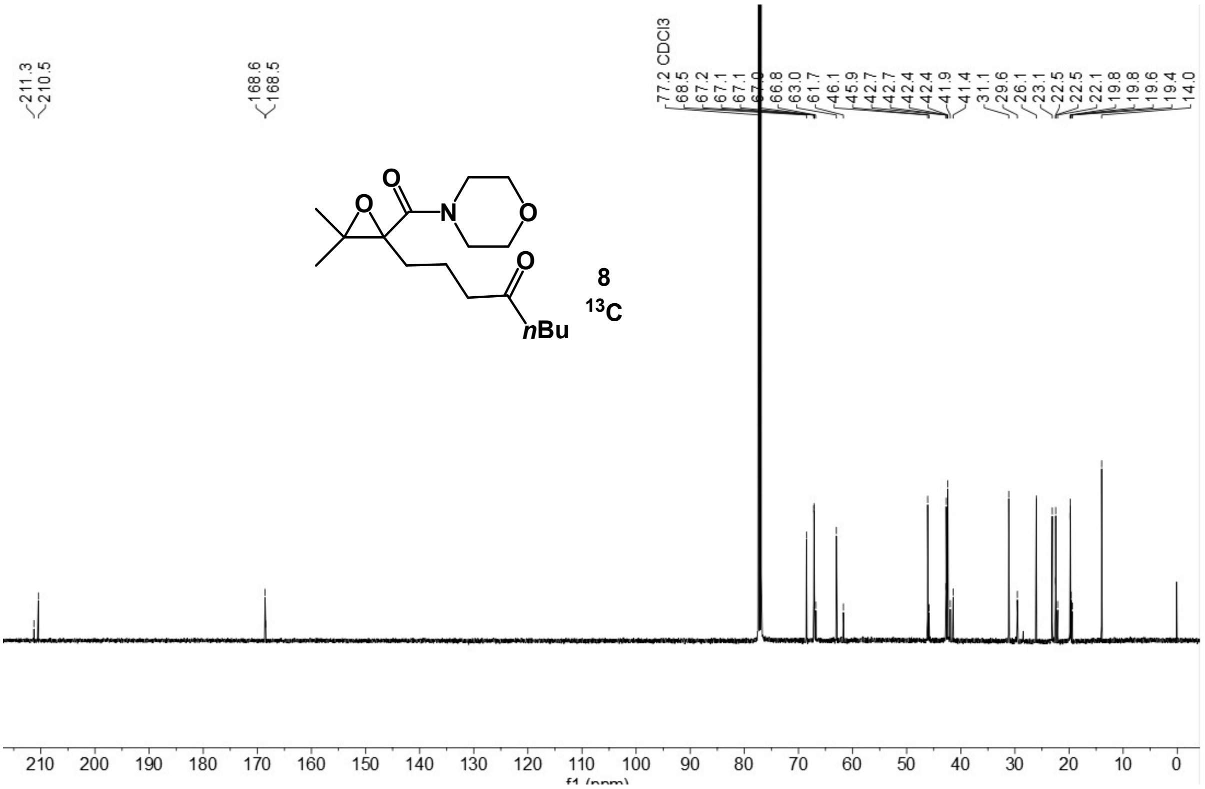
211.3 210.5
168.6 168.5
77.2 CDCl3
68.5 67.2 67.1 67.1 67.0 66.8 63.0 61.7 46.1 45.9 42.7 42.7 42.4 42.4 41.9 41.4 31.1 29.6 26.1 23.1 22.5 22.5 22.1 19.8 19.8 19.6 19.4 14.0
O
N
O
O
O
nBu
8
13C
210 200 190 180 170 160 150 140 130 120 110 100 90 80 70 60 50 40 30 20 10 0

Crystallography data for 3f, 5a, and 7b

Table S1. Crystal data and structure refinement for **3f**.

Identification code	**3f**	
Empirical formula	C13 H20 N2 O2	
Formula weight	238.11	
Temperature	150(2) K	
Wavelength	0.71073 Å	
Crystal system	Triclinic	
Space group	P-1	
Unit cell dimensions	a = 5.7302(7) Å	α= 83.662(4)°.
	b = 9.9783(12) Å	β= 89.167(5)°.
	c = 11.5048(17) Å	γ = 86.054(4)°.
Volume	652.23(15) $Å^3$	
Z	2	
Density (calculated)	1.212 Mg/m^3	
Absorption coefficient	0.083 mm^{-1}	
F(000)	258	
Crystal size	0.320 x 0.200 x 0.180 mm^3	
Theta range for data collection	1.781 to 27.514°.	
Index ranges	-7<=h<=7, -9<=k<=12, -14<=l<=14	
Reflections collected	11552	
Independent reflections	2971 [R(int) = 0.0280]	
Completeness to theta = 25.242°	98.7 %	
Absorption correction	Semi-empirical from equivalents	
Max. and min. transmission	0.7456 and 0.7144	
Refinement method	Full-matrix least-squares on F^2	
Data / restraints / parameters	2971 / 3 / 172	
Goodness-of-fit on F^2	1.045	
Final R indices [I>2sigma(I)]	R1 = 0.0367, wR2 = 0.0867	
R indices (all data)	R1 = 0.0495, wR2 = 0.0920	
Extinction coefficient	n/a	
Largest diff. peak and hole	0.299 and -0.149 e.$Å^{-3}$	

Table S2. Atomic coordinates (x 10^4) and equivalent isotropic displacement parameters ($Å^2$x 10^3) for **3f**. U(eq) is defined as one third of the trace of the orthogonalized U^{ij} tensor.

	x	y	z	U(eq)

O(1)	6662(1)	6504(1)	9009(1)	24(1)
O(2)	7763(1)	5854(1)	6779(1)	29(1)
N(1)	4657(2)	7126(1)	6005(1)	24(1)
N(2)	1402(2)	1190(1)	7771(1)	38(1)
C(1)	4872(2)	6634(1)	8127(1)	19(1)
C(2)	5398(2)	7814(1)	8727(1)	22(1)
C(3)	3817(2)	8292(1)	9677(1)	27(1)
C(4)	6879(2)	8880(1)	8126(1)	30(1)
C(5)	5895(2)	6512(1)	6911(1)	21(1)
C(6)	2435(2)	7962(1)	6035(1)	29(1)
C(7)	2236(2)	8633(1)	4777(1)	34(1)
C(8)	3381(2)	7561(1)	4063(1)	37(1)
C(9)	5470(2)	6999(1)	4798(1)	31(1)
C(10)	2764(2)	5823(1)	8443(1)	22(1)
C(11)	2993(2)	4432(1)	8009(1)	24(1)
C(12)	933(2)	3608(1)	8443(1)	24(1)
C(13)	1158(2)	2248(1)	8071(1)	26(1)
O(1W)	620(20)	5483(15)	4985(15)	86(7)

Table S3. Bond lengths [Å] and angles [°] for **3f**.

O(1)-C(1)	1.4426(12)
O(1)-C(2)	1.4562(13)
O(2)-C(5)	1.2330(13)
N(1)-C(5)	1.3366(14)
N(1)-C(9)	1.4748(14)
N(1)-C(6)	1.4754(15)
N(2)-C(13)	1.1453(15)
C(1)-C(2)	1.4797(14)
C(1)-C(10)	1.5169(14)
C(1)-C(5)	1.5230(15)
C(2)-C(3)	1.5063(15)
C(2)-C(4)	1.5077(15)
C(3)-H(3A)	0.9800
C(3)-H(3B)	0.9800
C(3)-H(3C)	0.9800

C(4)-H(4A)	0.9800
C(4)-H(4B)	0.9800
C(4)-H(4C)	0.9800
C(6)-C(7)	1.5276(17)
C(6)-H(6A)	0.9900
C(6)-H(6B)	0.9900
C(7)-C(8)	1.5262(18)
C(7)-H(7A)	0.9900
C(7)-H(7B)	0.9900
C(8)-C(9)	1.5146(18)
C(8)-H(8A)	0.9900
C(8)-H(8B)	0.9900
C(9)-H(9A)	0.9900
C(9)-H(9B)	0.9900
C(10)-C(11)	1.5232(14)
C(10)-H(10A)	0.9900
C(10)-H(10B)	0.9900
C(11)-C(12)	1.5311(14)
C(11)-H(11A)	0.9900
C(11)-H(11B)	0.9900
C(12)-C(13)	1.4639(16)
C(12)-H(12A)	0.9900
C(12)-H(12B)	0.9900
O(1W)-H(1WA)	0.841(10)
O(1W)-H(1WB)	0.840(10)

C(1)-O(1)-C(2)	61.39(6)
C(5)-N(1)-C(9)	120.26(9)
C(5)-N(1)-C(6)	127.85(9)
C(9)-N(1)-C(6)	111.88(9)
O(1)-C(1)-C(2)	59.76(6)
O(1)-C(1)-C(10)	114.90(8)
C(2)-C(1)-C(10)	122.49(9)
O(1)-C(1)-C(5)	111.97(8)
C(2)-C(1)-C(5)	118.58(8)
C(10)-C(1)-C(5)	115.44(9)
O(1)-C(2)-C(1)	58.85(6)
O(1)-C(2)-C(3)	115.77(9)

C(1)-C(2)-C(3)	121.14(9)
O(1)-C(2)-C(4)	113.85(9)
C(1)-C(2)-C(4)	120.07(9)
C(3)-C(2)-C(4)	114.51(9)
C(2)-C(3)-H(3A)	109.5
C(2)-C(3)-H(3B)	109.5
H(3A)-C(3)-H(3B)	109.5
C(2)-C(3)-H(3C)	109.5
H(3A)-C(3)-H(3C)	109.5
H(3B)-C(3)-H(3C)	109.5
C(2)-C(4)-H(4A)	109.5
C(2)-C(4)-H(4B)	109.5
H(4A)-C(4)-H(4B)	109.5
C(2)-C(4)-H(4C)	109.5
H(4A)-C(4)-H(4C)	109.5
H(4B)-C(4)-H(4C)	109.5
O(2)-C(5)-N(1)	122.08(10)
O(2)-C(5)-C(1)	120.91(9)
N(1)-C(5)-C(1)	117.00(9)
N(1)-C(6)-C(7)	102.43(9)
N(1)-C(6)-H(6A)	111.3
C(7)-C(6)-H(6A)	111.3
N(1)-C(6)-H(6B)	111.3
C(7)-C(6)-H(6B)	111.3
H(6A)-C(6)-H(6B)	109.2
C(8)-C(7)-C(6)	103.24(10)
C(8)-C(7)-H(7A)	111.1
C(6)-C(7)-H(7A)	111.1
C(8)-C(7)-H(7B)	111.1
C(6)-C(7)-H(7B)	111.1
H(7A)-C(7)-H(7B)	109.1
C(9)-C(8)-C(7)	103.07(10)
C(9)-C(8)-H(8A)	111.1
C(7)-C(8)-H(8A)	111.1
C(9)-C(8)-H(8B)	111.1
C(7)-C(8)-H(8B)	111.1
H(8A)-C(8)-H(8B)	109.1
N(1)-C(9)-C(8)	103.37(9)

N(1)-C(9)-H(9A)	111.1
C(8)-C(9)-H(9A)	111.1
N(1)-C(9)-H(9B)	111.1
C(8)-C(9)-H(9B)	111.1
H(9A)-C(9)-H(9B)	109.1
C(1)-C(10)-C(11)	112.65(9)
C(1)-C(10)-H(10A)	109.1
C(11)-C(10)-H(10A)	109.1
C(1)-C(10)-H(10B)	109.1
C(11)-C(10)-H(10B)	109.1
H(10A)-C(10)-H(10B)	107.8
C(10)-C(11)-C(12)	110.64(9)
C(10)-C(11)-H(11A)	109.5
C(12)-C(11)-H(11A)	109.5
C(10)-C(11)-H(11B)	109.5
C(12)-C(11)-H(11B)	109.5
H(11A)-C(11)-H(11B)	108.1
C(13)-C(12)-C(11)	111.62(9)
C(13)-C(12)-H(12A)	109.3
C(11)-C(12)-H(12A)	109.3
C(13)-C(12)-H(12B)	109.3
C(11)-C(12)-H(12B)	109.3
H(12A)-C(12)-H(12B)	108.0
N(2)-C(13)-C(12)	177.93(12)
H(1WA)-O(1W)-H(1WB)	113(2)

Symmetry transformations used to generate equivalent atoms:

Table S4. Anisotropic displacement parameters (Å^{2}x 10^3) for **3f**. The anisotropic displacement factor exponent takes the form: $-2\pi^2[h^2 a^{*2}U^{11} + ... + 2 h k a^* b^* U^{12}]$

	U^{11}	U^{22}	U^{33}	U^{23}	U^{13}	U^{12}
O(1)	25(1)	26(1)	22(1)	-2(1)	-5(1)	0(1)
O(2)	25(1)	34(1)	26(1)	-3(1)	3(1)	6(1)
N(1)	24(1)	29(1)	18(1)	-3(1)	2(1)	2(1)
N(2)	42(1)	27(1)	48(1)	-3(1)	-7(1)	-7(1)
C(1)	20(1)	20(1)	18(1)	-1(1)	-1(1)	-1(1)

C(2)	23(1)	21(1)	21(1)	-2(1)	-2(1)	-2(1)
C(3)	33(1)	25(1)	24(1)	-6(1)	3(1)	-4(1)
C(4)	31(1)	26(1)	34(1)	-5(1)	4(1)	-9(1)
C(5)	22(1)	20(1)	21(1)	-2(1)	1(1)	-4(1)
C(6)	26(1)	34(1)	25(1)	-4(1)	-2(1)	4(1)
C(7)	38(1)	36(1)	28(1)	-2(1)	-7(1)	4(1)
C(8)	47(1)	42(1)	22(1)	-4(1)	-5(1)	2(1)
C(9)	37(1)	38(1)	19(1)	-4(1)	4(1)	1(1)
C(10)	22(1)	22(1)	21(1)	-4(1)	3(1)	-4(1)
C(11)	27(1)	22(1)	24(1)	-4(1)	4(1)	-5(1)
C(12)	24(1)	23(1)	24(1)	-1(1)	-1(1)	-5(1)
C(13)	25(1)	26(1)	27(1)	3(1)	-5(1)	-7(1)
O(1W)	77(10)	77(10)	114(13)	-60(9)	68(9)	-25(7)

Table S5. Hydrogen coordinates (x 10^4) and isotropic displacement parameters (Å^{2}x 10 ^{3}) for **3f**.

	x	y	z	U(eq)
H(3A)	2967	7535	10044	41
H(3B)	2697	9010	9337	41
H(3C)	4757	8641	10266	41
H(4A)	7858	8489	7526	45
H(4B)	7879	9208	8702	45
H(4C)	5862	9633	7758	45
H(6A)	2505	8643	6595	34
H(6B)	1105	7398	6246	34
H(7A)	579	8849	4555	41
H(7B)	3078	9472	4671	41
H(8A)	2302	6849	3965	44
H(8B)	3878	7968	3283	44
H(9A)	5885	6043	4683	38
H(9B)	6846	7532	4603	38
H(10A)	1346	6330	8101	26
H(10B)	2563	5715	9304	26
H(11A)	4476	3948	8294	29
H(11B)	3037	4533	7144	29

H(12A)	856	3540	9308	29
H(12B)	-543	4080	8135	29
H(1WA)	600(400)	4900(200)	4480(150)	128
H(1WB)	-600(200)	5500(200)	5400(170)	128

Table S6. Torsion angles [°] for **3f**.

C(2)-O(1)-C(1)-C(10)	-114.52(10)
C(2)-O(1)-C(1)-C(5)	111.22(9)
C(1)-O(1)-C(2)-C(3)	112.28(10)
C(1)-O(1)-C(2)-C(4)	-111.90(10)
C(10)-C(1)-C(2)-O(1)	101.92(10)
C(5)-C(1)-C(2)-O(1)	-100.11(10)
O(1)-C(1)-C(2)-C(3)	-103.20(11)
C(10)-C(1)-C(2)-C(3)	-1.28(15)
C(5)-C(1)-C(2)-C(3)	156.69(10)
O(1)-C(1)-C(2)-C(4)	101.31(11)
C(10)-C(1)-C(2)-C(4)	-156.78(10)
C(5)-C(1)-C(2)-C(4)	1.19(15)
C(9)-N(1)-C(5)-O(2)	1.83(16)
C(6)-N(1)-C(5)-O(2)	-178.69(10)
C(9)-N(1)-C(5)-C(1)	-176.96(9)
C(6)-N(1)-C(5)-C(1)	2.52(16)
O(1)-C(1)-C(5)-O(2)	27.39(13)
C(2)-C(1)-C(5)-O(2)	93.89(12)
C(10)-C(1)-C(5)-O(2)	-106.61(12)
O(1)-C(1)-C(5)-N(1)	-153.81(9)
C(2)-C(1)-C(5)-N(1)	-87.30(12)
C(10)-C(1)-C(5)-N(1)	72.19(12)
C(5)-N(1)-C(6)-C(7)	166.93(11)
C(9)-N(1)-C(6)-C(7)	-13.55(12)
N(1)-C(6)-C(7)-C(8)	32.83(12)
C(6)-C(7)-C(8)-C(9)	-40.50(13)
C(5)-N(1)-C(9)-C(8)	168.18(10)
C(6)-N(1)-C(9)-C(8)	-11.38(13)
C(7)-C(8)-C(9)-N(1)	31.62(13)
O(1)-C(1)-C(10)-C(11)	-92.80(11)

C(2)-C(1)-C(10)-C(11)	-161.54(9)
C(5)-C(1)-C(10)-C(11)	39.85(13)
C(1)-C(10)-C(11)-C(12)	174.87(9)
C(10)-C(11)-C(12)-C(13)	-177.97(9)

Symmetry transformations used to generate equivalent atoms:

Table S7. Crystal data and structure refinement for **5a**.

Identification code	**5a**	
Empirical formula	C13 H19 N O5	
Formula weight	269.29	
Temperature	150(2) K	
Wavelength	1.54178 Å	
Crystal system	Monoclinic	
Space group	$P2_1/c$	
Unit cell dimensions	a = 11.2115(8) Å	α= 90°.
	b = 11.5329(9) Å	β= 104.981(4)°.
	c = 11.1450(8) Å	γ = 90°.
Volume	1392.08(18) $Å^3$	
Z	4	
Density (calculated)	1.285 Mg/m^3	
Absorption coefficient	0.826 mm^{-1}	
F(000)	576	
Crystal size	0.370 x 0.340 x 0.250 mm^3	
Theta range for data collection	4.082 to 66.215°.	
Index ranges	-13<=h<=13, -13<=k<=13, -13<=l<=13	
Reflections collected	34811	
Independent reflections	2373 [R(int) = 0.0409]	
Completeness to theta = 66.215°	97.4 %	
Absorption correction	Semi-empirical from equivalents	
Max. and min. transmission	0.7528 and 0.6980	
Refinement method	Full-matrix least-squares on F^2	
Data / restraints / parameters	2373 / 0 / 174	
Goodness-of-fit on F^2	1.101	
Final R indices [I>2sigma(I)]	R1 = 0.0343, wR2 = 0.0829	
R indices (all data)	R1 = 0.0381, wR2 = 0.0853	
Extinction coefficient	n/a	
Largest diff. peak and hole	0.242 and -0.181 e.$Å^{-3}$	

Table S8. Atomic coordinates (x 10^4) and equivalent isotropic displacement parameters ($Å^2$x 10^3) for **5a**. U(eq) is defined as one third of the trace of the orthogonalized U^{ij} tensor.

	x	y	z	U(eq)
O(1)	7586(1)	4515(1)	7896(1)	28(1)
O(2)	9391(1)	3112(1)	7498(1)	33(1)
O(3)	8074(1)	2346(1)	5805(1)	26(1)
O(4)	5210(1)	3455(1)	5777(1)	39(1)
O(5)	6275(1)	2471(1)	7450(1)	34(1)
N(1)	7754(1)	5733(1)	7726(1)	27(1)
C(1)	7355(1)	3934(1)	6711(1)	22(1)
C(2)	7264(1)	4898(1)	5739(1)	22(1)
C(3)	8119(1)	4853(1)	4859(1)	27(1)
C(4)	8089(1)	6024(1)	4207(1)	29(1)
C(5)	8496(1)	7013(1)	5126(1)	31(1)
C(6)	7695(1)	7102(1)	6048(1)	30(1)
C(7)	7568(1)	5934(1)	6571(1)	23(1)
C(8)	8412(1)	3084(1)	6749(1)	22(1)
C(9)	8973(1)	1457(1)	5681(1)	28(1)
C(10)	8644(2)	337(1)	6164(2)	57(1)
C(11)	6144(1)	3266(1)	6563(1)	26(1)
C(12)	5199(1)	1738(1)	7424(2)	39(1)
C(13)	5173(2)	730(2)	6585(2)	51(1)

Table S9. Bond lengths [Å] and angles [°] for **5a**.

O(1)-N(1)	1.4358(15)
O(1)-C(1)	1.4436(15)
O(2)-C(8)	1.1962(15)
O(3)-C(8)	1.3297(15)
O(3)-C(9)	1.4687(16)
O(4)-C(11)	1.1991(16)
O(5)-C(11)	1.3281(16)
O(5)-C(12)	1.4672(17)
N(1)-C(7)	1.2709(17)

C(1)-C(8)	1.5296(18)
C(1)-C(11)	1.5328(18)
C(1)-C(2)	1.5378(17)
C(2)-C(7)	1.4967(18)
C(2)-C(3)	1.5394(18)
C(2)-H(2A)	1.0000
C(3)-C(4)	1.5302(18)
C(3)-H(3A)	0.9900
C(3)-H(3B)	0.9900
C(4)-C(5)	1.5214(19)
C(4)-H(4A)	0.9900
C(4)-H(4B)	0.9900
C(5)-C(6)	1.5328(19)
C(5)-H(5A)	0.9900
C(5)-H(5B)	0.9900
C(6)-C(7)	1.4892(19)
C(6)-H(6A)	0.9900
C(6)-H(6B)	0.9900
C(9)-C(10)	1.483(2)
C(9)-H(9A)	0.9900
C(9)-H(9B)	0.9900
C(10)-H(10A)	0.9800
C(10)-H(10B)	0.9800
C(10)-H(10C)	0.9800
C(12)-C(13)	1.487(2)
C(12)-H(12A)	0.9900
C(12)-H(12B)	0.9900
C(13)-H(13A)	0.9800
C(13)-H(13B)	0.9800
C(13)-H(13C)	0.9800

N(1)-O(1)-C(1)	109.44(9)
C(8)-O(3)-C(9)	117.35(10)
C(11)-O(5)-C(12)	116.77(11)
C(7)-N(1)-O(1)	108.60(10)
O(1)-C(1)-C(8)	108.54(10)
O(1)-C(1)-C(11)	106.04(10)
C(8)-C(1)-C(11)	109.82(10)

O(1)-C(1)-C(2)	105.76(10)
C(8)-C(1)-C(2)	113.15(10)
C(11)-C(1)-C(2)	113.10(10)
C(7)-C(2)-C(1)	100.24(10)
C(7)-C(2)-C(3)	110.02(10)
C(1)-C(2)-C(3)	119.01(10)
C(7)-C(2)-H(2A)	109.0
C(1)-C(2)-H(2A)	109.0
C(3)-C(2)-H(2A)	109.0
C(4)-C(3)-C(2)	109.34(11)
C(4)-C(3)-H(3A)	109.8
C(2)-C(3)-H(3A)	109.8
C(4)-C(3)-H(3B)	109.8
C(2)-C(3)-H(3B)	109.8
H(3A)-C(3)-H(3B)	108.3
C(5)-C(4)-C(3)	112.12(11)
C(5)-C(4)-H(4A)	109.2
C(3)-C(4)-H(4A)	109.2
C(5)-C(4)-H(4B)	109.2
C(3)-C(4)-H(4B)	109.2
H(4A)-C(4)-H(4B)	107.9
C(4)-C(5)-C(6)	112.05(11)
C(4)-C(5)-H(5A)	109.2
C(6)-C(5)-H(5A)	109.2
C(4)-C(5)-H(5B)	109.2
C(6)-C(5)-H(5B)	109.2
H(5A)-C(5)-H(5B)	107.9
C(7)-C(6)-C(5)	109.52(11)
C(7)-C(6)-H(6A)	109.8
C(5)-C(6)-H(6A)	109.8
C(7)-C(6)-H(6B)	109.8
C(5)-C(6)-H(6B)	109.8
H(6A)-C(6)-H(6B)	108.2
N(1)-C(7)-C(6)	123.70(12)
N(1)-C(7)-C(2)	115.69(12)
C(6)-C(7)-C(2)	120.55(11)
O(2)-C(8)-O(3)	126.28(12)
O(2)-C(8)-C(1)	124.51(12)

O(3)-C(8)-C(1)	109.18(10)
O(3)-C(9)-C(10)	109.38(12)
O(3)-C(9)-H(9A)	109.8
C(10)-C(9)-H(9A)	109.8
O(3)-C(9)-H(9B)	109.8
C(10)-C(9)-H(9B)	109.8
H(9A)-C(9)-H(9B)	108.2
C(9)-C(10)-H(10A)	109.5
C(9)-C(10)-H(10B)	109.5
H(10A)-C(10)-H(10B)	109.5
C(9)-C(10)-H(10C)	109.5
H(10A)-C(10)-H(10C)	109.5
H(10B)-C(10)-H(10C)	109.5
O(4)-C(11)-O(5)	125.27(12)
O(4)-C(11)-C(1)	124.68(12)
O(5)-C(11)-C(1)	110.03(11)
O(5)-C(12)-C(13)	110.17(12)
O(5)-C(12)-H(12A)	109.6
C(13)-C(12)-H(12A)	109.6
O(5)-C(12)-H(12B)	109.6
C(13)-C(12)-H(12B)	109.6
H(12A)-C(12)-H(12B)	108.1
C(12)-C(13)-H(13A)	109.5
C(12)-C(13)-H(13B)	109.5
H(13A)-C(13)-H(13B)	109.5
C(12)-C(13)-H(13C)	109.5
H(13A)-C(13)-H(13C)	109.5
H(13B)-C(13)-H(13C)	109.5

Symmetry transformations used to generate equivalent atoms:

Table S10. Anisotropic displacement parameters ($Å^2$x 10^3) for **5a**. The anisotropic displacement factor exponent takes the form: $-2\pi^2[h^2 a^{*2}U^{11} + ... + 2 h k a^* b^* U^{12}]$

	U^{11}	U^{22}	U^{33}	U^{23}	U^{13}	U^{12}
O(1)	37(1)	25(1)	21(1)	0(1)	7(1)	3(1)
O(2)	24(1)	37(1)	33(1)	-6(1)	-4(1)	5(1)

O(3)	26(1)	23(1)	27(1)	-3(1)	2(1)	3(1)
O(4)	22(1)	42(1)	48(1)	17(1)	0(1)	-2(1)
O(5)	28(1)	38(1)	35(1)	14(1)	7(1)	-4(1)
N(1)	28(1)	24(1)	30(1)	-3(1)	8(1)	0(1)
C(1)	23(1)	23(1)	19(1)	0(1)	4(1)	1(1)
C(2)	20(1)	20(1)	23(1)	1(1)	2(1)	1(1)
C(3)	32(1)	25(1)	23(1)	1(1)	9(1)	2(1)
C(4)	33(1)	28(1)	26(1)	4(1)	8(1)	0(1)
C(5)	30(1)	28(1)	32(1)	4(1)	7(1)	-6(1)
C(6)	34(1)	22(1)	33(1)	-3(1)	8(1)	-4(1)
C(7)	20(1)	24(1)	26(1)	-3(1)	6(1)	0(1)
C(8)	24(1)	21(1)	22(1)	3(1)	5(1)	-1(1)
C(9)	29(1)	26(1)	30(1)	-2(1)	10(1)	5(1)
C(10)	67(1)	31(1)	86(1)	18(1)	42(1)	17(1)
C(11)	24(1)	25(1)	28(1)	4(1)	8(1)	3(1)
C(12)	30(1)	42(1)	48(1)	15(1)	16(1)	-5(1)
C(13)	42(1)	41(1)	75(1)	4(1)	25(1)	-10(1)

Table S11. Hydrogen coordinates (x 10^4) and isotropic displacement parameters (Å^{2}x 10 ^{3}) for **5a**.

	x	y	z	U(eq)
H(2A)	6388	4961	5233	26
H(3A)	8973	4674	5338	32
H(3B)	7842	4233	4234	32
H(4A)	8639	5992	3641	35
H(4B)	7240	6177	3699	35
H(5A)	8448	7752	4663	37
H(5B)	9367	6891	5590	37
H(6A)	8081	7643	6726	36
H(6B)	6869	7407	5620	36
H(9A)	9812	1694	6153	34
H(9B)	8972	1372	4796	34
H(10A)	9239	-258	6075	85
H(10B)	7813	107	5694	85
H(10C)	8662	422	7044	85

H(12A)	4433	2198	7128	47
H(12B)	5237	1459	8273	47
H(13A)	4461	238	6588	77
H(13B)	5935	280	6875	77
H(13C)	5107	1008	5739	77

Table S12. Torsion angles [°] for **5a**.

C(1)-O(1)-N(1)-C(7)	4.12(13)
N(1)-O(1)-C(1)-C(8)	116.41(10)
N(1)-O(1)-C(1)-C(11)	-125.65(10)
N(1)-O(1)-C(1)-C(2)	-5.29(12)
O(1)-C(1)-C(2)-C(7)	4.31(12)
C(8)-C(1)-C(2)-C(7)	-114.38(11)
C(11)-C(1)-C(2)-C(7)	119.94(11)
O(1)-C(1)-C(2)-C(3)	124.17(12)
C(8)-C(1)-C(2)-C(3)	5.48(16)
C(11)-C(1)-C(2)-C(3)	-120.20(12)
C(7)-C(2)-C(3)-C(4)	-51.64(14)
C(1)-C(2)-C(3)-C(4)	-166.37(11)
C(2)-C(3)-C(4)-C(5)	58.93(15)
C(3)-C(4)-C(5)-C(6)	-57.94(16)
C(4)-C(5)-C(6)-C(7)	48.22(15)
O(1)-N(1)-C(7)-C(6)	-178.27(11)
O(1)-N(1)-C(7)-C(2)	-1.07(15)
C(5)-C(6)-C(7)-N(1)	130.62(13)
C(5)-C(6)-C(7)-C(2)	-46.44(16)
C(1)-C(2)-C(7)-N(1)	-2.11(14)
C(3)-C(2)-C(7)-N(1)	-128.29(12)
C(1)-C(2)-C(7)-C(6)	175.18(11)
C(3)-C(2)-C(7)-C(6)	49.00(15)
C(9)-O(3)-C(8)-O(2)	1.68(19)
C(9)-O(3)-C(8)-C(1)	179.85(10)
O(1)-C(1)-C(8)-O(2)	-17.11(17)
C(11)-C(1)-C(8)-O(2)	-132.62(13)
C(2)-C(1)-C(8)-O(2)	99.96(14)
O(1)-C(1)-C(8)-O(3)	164.67(10)

C(11)-C(1)-C(8)-O(3)	49.16(13)
C(2)-C(1)-C(8)-O(3)	-78.26(13)
C(8)-O(3)-C(9)-C(10)	101.31(15)
C(12)-O(5)-C(11)-O(4)	2.7(2)
C(12)-O(5)-C(11)-C(1)	-178.83(11)
O(1)-C(1)-C(11)-O(4)	115.72(14)
C(8)-C(1)-C(11)-O(4)	-127.19(14)
C(2)-C(1)-C(11)-O(4)	0.26(19)
O(1)-C(1)-C(11)-O(5)	-62.73(13)
C(8)-C(1)-C(11)-O(5)	54.36(14)
C(2)-C(1)-C(11)-O(5)	-178.20(10)
C(11)-O(5)-C(12)-C(13)	85.64(16)

Symmetry transformations used to generate equivalent atoms:

Table S13. Crystal data and structure refinement for **7b**.

Identification code	**7b**	
Empirical formula	C13 H20 B F3 N2 O3	
Formula weight	320.12	
Temperature	150(2) K	
Wavelength	1.54178 Å	
Crystal system	Monoclinic	
Space group	C2/c	
Unit cell dimensions	a = 31.0762(14) Å	α= 90°.
	b = 9.4685(4) Å	β= 91.156(2)°.
	c = 12.1756(5) Å	γ = 90°.
Volume	3581.9(3) Å^3	
Z	8	
Density (calculated)	1.187 Mg/m^3	
Absorption coefficient	0.888 mm^{-1}	
F(000)	1344	
Crystal size	0.260 x 0.130 x 0.020 mm^3	
Theta range for data collection	2.844 to 65.997°.	
Index ranges	-36<=h<=36, -11<=k<=11, -14<=l<=14	
Reflections collected	32058	
Independent reflections	3076 [R(int) = 0.0364]	
Completeness to theta = 65.997°	98.3 %	
Absorption correction	Semi-empirical from equivalents	

Max. and min. transmission	0.7527 and 0.5999
Refinement method	Full-matrix least-squares on F^2
Data / restraints / parameters	3076 / 0 / 201
Goodness-of-fit on F^2	1.051
Final R indices [I>2sigma(I)]	R1 = 0.0451, wR2 = 0.1210
R indices (all data)	R1 = 0.0504, wR2 = 0.1240
Extinction coefficient	n/a
Largest diff. peak and hole	0.238 and -0.234 e.Å^{-3}

Table S14. Atomic coordinates (x 10^4) and equivalent isotropic displacement parameters (Å^{2}x 10^3) for **7b**. U(eq) is defined as one third of the trace of the orthogonalized U^{ij} tensor.

	x	y	z	U(eq)
F(1)	6104(1)	3129(1)	6933(1)	43(1)
F(2)	6800(1)	3114(1)	6453(1)	45(1)
F(3)	7021(1)	8202(1)	7746(1)	60(1)
O(1)	6585(1)	4685(1)	7838(1)	31(1)
O(2)	6375(1)	5129(1)	6028(1)	33(1)
O(3)	6311(1)	9896(2)	4627(1)	71(1)
N(1)	6289(1)	7469(2)	5976(1)	33(1)
N(2)	4727(1)	6402(3)	9103(2)	63(1)
C(1)	6570(1)	6171(2)	7709(1)	29(1)
C(2)	6395(1)	6333(2)	6518(2)	29(1)
C(3)	6238(1)	8891(2)	6443(2)	47(1)
C(4)	6462(1)	9951(3)	5739(2)	70(1)
C(5)	6164(1)	7400(2)	4797(2)	38(1)
C(6)	6398(1)	8534(2)	4185(2)	53(1)
C(7)	7045(1)	6715(2)	7806(2)	39(1)
C(8)	7245(1)	6326(3)	8906(2)	59(1)
C(9)	7324(1)	6243(3)	6871(2)	49(1)
C(10)	6280(1)	6778(2)	8598(1)	30(1)
C(11)	5806(1)	6408(2)	8416(2)	33(1)
C(12)	5551(1)	6804(3)	9429(2)	41(1)
C(13)	5088(1)	6583(3)	9255(2)	46(1)
B(1)	6473(1)	3942(2)	6850(2)	33(1)

Table S15. Bond lengths [Å] and angles [°] for **7b**.

F(1)-B(1)	1.386(3)
F(2)-B(1)	1.378(3)
F(3)-C(7)	1.411(3)
O(1)-C(1)	1.417(2)
O(1)-B(1)	1.430(2)
O(2)-C(2)	1.288(2)
O(2)-B(1)	1.531(2)
O(3)-C(6)	1.425(3)
O(3)-C(4)	1.426(3)
N(1)-C(2)	1.301(2)
N(1)-C(3)	1.472(2)
N(1)-C(5)	1.481(3)
N(2)-C(13)	1.145(3)
C(1)-C(10)	1.532(2)
C(1)-C(2)	1.545(2)
C(1)-C(7)	1.566(3)
C(3)-C(4)	1.501(3)
C(3)-H(3A)	0.9900
C(3)-H(3B)	0.9900
C(4)-H(4A)	0.9900
C(4)-H(4B)	0.9900
C(5)-C(6)	1.504(3)
C(5)-H(5A)	0.9900
C(5)-H(5B)	0.9900
C(6)-H(6A)	0.9900
C(6)-H(6B)	0.9900
C(7)-C(8)	1.512(3)
C(7)-C(9)	1.512(3)
C(8)-H(8A)	0.9800
C(8)-H(8B)	0.9800
C(8)-H(8C)	0.9800
C(9)-H(9A)	0.9800
C(9)-H(9B)	0.9800
C(9)-H(9C)	0.9800
C(10)-C(11)	1.527(3)
C(10)-H(10A)	0.9900

C(10)-H(10B)	0.9900
C(11)-C(12)	1.527(3)
C(11)-H(11A)	0.9900
C(11)-H(11B)	0.9900
C(12)-C(13)	1.465(3)
C(12)-H(12A)	0.9900
C(12)-H(12B)	0.9900

C(1)-O(1)-B(1)	112.87(14)
C(2)-O(2)-B(1)	109.86(14)
C(6)-O(3)-C(4)	109.2(2)
C(2)-N(1)-C(3)	126.03(16)
C(2)-N(1)-C(5)	120.95(16)
C(3)-N(1)-C(5)	112.76(16)
O(1)-C(1)-C(10)	108.22(14)
O(1)-C(1)-C(2)	102.24(13)
C(10)-C(1)-C(2)	115.21(15)
O(1)-C(1)-C(7)	106.85(15)
C(10)-C(1)-C(7)	112.91(15)
C(2)-C(1)-C(7)	110.50(15)
O(2)-C(2)-N(1)	119.15(16)
O(2)-C(2)-C(1)	111.12(15)
N(1)-C(2)-C(1)	129.68(16)
N(1)-C(3)-C(4)	109.63(19)
N(1)-C(3)-H(3A)	109.7
C(4)-C(3)-H(3A)	109.7
N(1)-C(3)-H(3B)	109.7
C(4)-C(3)-H(3B)	109.7
H(3A)-C(3)-H(3B)	108.2
O(3)-C(4)-C(3)	111.7(2)
O(3)-C(4)-H(4A)	109.3
C(3)-C(4)-H(4A)	109.3
O(3)-C(4)-H(4B)	109.3
C(3)-C(4)-H(4B)	109.3
H(4A)-C(4)-H(4B)	107.9
N(1)-C(5)-C(6)	109.13(18)
N(1)-C(5)-H(5A)	109.9
C(6)-C(5)-H(5A)	109.9

N(1)-C(5)-H(5B)	109.9
C(6)-C(5)-H(5B)	109.9
H(5A)-C(5)-H(5B)	108.3
O(3)-C(6)-C(5)	111.16(19)
O(3)-C(6)-H(6A)	109.4
C(5)-C(6)-H(6A)	109.4
O(3)-C(6)-H(6B)	109.4
C(5)-C(6)-H(6B)	109.4
H(6A)-C(6)-H(6B)	108.0
F(3)-C(7)-C(8)	107.98(19)
F(3)-C(7)-C(9)	106.62(18)
C(8)-C(7)-C(9)	111.24(19)
F(3)-C(7)-C(1)	106.09(16)
C(8)-C(7)-C(1)	110.98(17)
C(9)-C(7)-C(1)	113.55(17)
C(7)-C(8)-H(8A)	109.5
C(7)-C(8)-H(8B)	109.5
H(8A)-C(8)-H(8B)	109.5
C(7)-C(8)-H(8C)	109.5
H(8A)-C(8)-H(8C)	109.5
H(8B)-C(8)-H(8C)	109.5
C(7)-C(9)-H(9A)	109.5
C(7)-C(9)-H(9B)	109.5
H(9A)-C(9)-H(9B)	109.5
C(7)-C(9)-H(9C)	109.5
H(9A)-C(9)-H(9C)	109.5
H(9B)-C(9)-H(9C)	109.5
C(11)-C(10)-C(1)	112.97(14)
C(11)-C(10)-H(10A)	109.0
C(1)-C(10)-H(10A)	109.0
C(11)-C(10)-H(10B)	109.0
C(1)-C(10)-H(10B)	109.0
H(10A)-C(10)-H(10B)	107.8
C(12)-C(11)-C(10)	110.02(15)
C(12)-C(11)-H(11A)	109.7
C(10)-C(11)-H(11A)	109.7
C(12)-C(11)-H(11B)	109.7
C(10)-C(11)-H(11B)	109.7

H(11A)-C(11)-H(11B)	108.2
C(13)-C(12)-C(11)	111.90(17)
C(13)-C(12)-H(12A)	109.2
C(11)-C(12)-H(12A)	109.2
C(13)-C(12)-H(12B)	109.2
C(11)-C(12)-H(12B)	109.2
H(12A)-C(12)-H(12B)	107.9
N(2)-C(13)-C(12)	178.9(2)
F(2)-B(1)-F(1)	109.01(17)
F(2)-B(1)-O(1)	114.02(17)
F(1)-B(1)-O(1)	113.49(16)
F(2)-B(1)-O(2)	109.09(16)
F(1)-B(1)-O(2)	107.50(16)
O(1)-B(1)-O(2)	103.32(15)

Symmetry transformations used to generate equivalent atoms:

Table S16. Anisotropic displacement parameters (Å^{2}x 10^3) for **7b**. The anisotropic displacement factor exponent takes the form: $-2\pi^2$[h^2 $a^{*2}U^{11}$ + ... + 2 h k a* b* U^{12}]

	U^{11}	U^{22}	U^{33}	U^{23}	U^{13}	U^{12}
F(1)	53(1)	35(1)	41(1)	2(1)	-9(1)	-9(1)
F(2)	61(1)	36(1)	39(1)	-7(1)	2(1)	15(1)
F(3)	51(1)	41(1)	88(1)	-15(1)	14(1)	-19(1)
O(1)	39(1)	27(1)	26(1)	-3(1)	-2(1)	1(1)
O(2)	50(1)	23(1)	25(1)	-4(1)	-2(1)	2(1)
O(3)	139(2)	28(1)	48(1)	6(1)	7(1)	-9(1)
N(1)	45(1)	25(1)	28(1)	-3(1)	5(1)	-1(1)
N(2)	36(1)	105(2)	49(1)	2(1)	0(1)	8(1)
C(1)	32(1)	26(1)	28(1)	-4(1)	1(1)	-2(1)
C(2)	33(1)	25(1)	28(1)	-4(1)	5(1)	-2(1)
C(3)	78(2)	24(1)	38(1)	-4(1)	7(1)	6(1)
C(4)	124(3)	31(1)	54(2)	-3(1)	5(2)	-20(1)
C(5)	56(1)	30(1)	29(1)	2(1)	1(1)	0(1)
C(6)	86(2)	36(1)	38(1)	3(1)	14(1)	-8(1)
C(7)	35(1)	40(1)	43(1)	-8(1)	5(1)	-9(1)
C(8)	35(1)	97(2)	46(1)	-9(1)	-5(1)	-18(1)

C(9)	36(1)	63(2)	49(1)	-2(1)	11(1)	-2(1)
C(10)	34(1)	31(1)	26(1)	-6(1)	1(1)	-2(1)
C(11)	32(1)	39(1)	30(1)	-5(1)	0(1)	1(1)
C(12)	32(1)	57(1)	34(1)	-3(1)	2(1)	3(1)
C(13)	39(1)	66(2)	34(1)	3(1)	3(1)	10(1)
B(1)	45(1)	27(1)	28(1)	-1(1)	-2(1)	3(1)

Table S17. Hydrogen coordinates (x 10^4) and isotropic displacement parameters (Å^{2}x 10 ^{3}) for **7b**.

	x	y	z	U(eq)
H(3A)	6362	8916	7198	56
H(3B)	5929	9128	6481	56
H(4A)	6414	10910	6036	84
H(4B)	6776	9764	5765	84
H(5A)	5849	7538	4709	46
H(5B)	6237	6461	4497	46
H(6A)	6712	8350	4232	64
H(6B)	6309	8511	3400	64
H(8A)	7548	6623	8929	89
H(8B)	7229	5301	9008	89
H(8C)	7090	6800	9493	89
H(9A)	7613	6639	6969	74
H(9B)	7197	6573	6173	74
H(9C)	7341	5209	6865	74
H(10A)	6378	6411	9323	36
H(10B)	6312	7818	8611	36
H(11A)	5690	6926	7767	40
H(11B)	5776	5384	8271	40
H(12A)	5652	6226	10060	49
H(12B)	5604	7809	9611	49

Table S18. Torsion angles [°] for **7b**.

B(1)-O(1)-C(1)-C(10)	126.53(16)
B(1)-O(1)-C(1)-C(2)	4.50(19)
B(1)-O(1)-C(1)-C(7)	-111.61(17)
B(1)-O(2)-C(2)-N(1)	-174.74(17)
B(1)-O(2)-C(2)-C(1)	7.6(2)
C(3)-N(1)-C(2)-O(2)	171.21(19)
C(5)-N(1)-C(2)-O(2)	-2.6(3)
C(3)-N(1)-C(2)-C(1)	-11.6(3)
C(5)-N(1)-C(2)-C(1)	174.56(18)
O(1)-C(1)-C(2)-O(2)	-7.63(19)
C(10)-C(1)-C(2)-O(2)	-124.75(17)
C(7)-C(1)-C(2)-O(2)	105.81(18)
O(1)-C(1)-C(2)-N(1)	175.03(18)
C(10)-C(1)-C(2)-N(1)	57.9(3)
C(7)-C(1)-C(2)-N(1)	-71.5(2)
C(2)-N(1)-C(3)-C(4)	134.0(2)
C(5)-N(1)-C(3)-C(4)	-51.7(3)
C(6)-O(3)-C(4)-C(3)	-61.6(3)
N(1)-C(3)-C(4)-O(3)	56.0(3)
C(2)-N(1)-C(5)-C(6)	-133.0(2)
C(3)-N(1)-C(5)-C(6)	52.4(2)
C(4)-O(3)-C(6)-C(5)	62.3(3)
N(1)-C(5)-C(6)-O(3)	-57.3(3)
O(1)-C(1)-C(7)-F(3)	-176.35(14)
C(10)-C(1)-C(7)-F(3)	-57.5(2)
C(2)-C(1)-C(7)-F(3)	73.18(19)
O(1)-C(1)-C(7)-C(8)	-59.3(2)
C(10)-C(1)-C(7)-C(8)	59.6(2)
C(2)-C(1)-C(7)-C(8)	-169.78(18)
O(1)-C(1)-C(7)-C(9)	66.9(2)
C(10)-C(1)-C(7)-C(9)	-174.26(18)
C(2)-C(1)-C(7)-C(9)	-43.6(2)
O(1)-C(1)-C(10)-C(11)	-69.65(19)
C(2)-C(1)-C(10)-C(11)	44.0(2)
C(7)-C(1)-C(10)-C(11)	172.29(16)
C(1)-C(10)-C(11)-C(12)	169.54(16)

C(10)-C(11)-C(12)-C(13)	175.01(18)
C(1)-O(1)-B(1)-F(2)	117.69(18)
C(1)-O(1)-B(1)-F(1)	-116.67(18)
C(1)-O(1)-B(1)-O(2)	-0.6(2)
C(2)-O(2)-B(1)-F(2)	-126.22(17)
C(2)-O(2)-B(1)-F(1)	115.71(17)
C(2)-O(2)-B(1)-O(1)	-4.6(2)

Symmetry transformations used to generate equivalent atoms:

Table S19. Crystal data and structure refinement for ***cis*-1r**.

Identification code	***cis*-1r**	
Empirical formula	C22 H34 N2 O5 S	
Formula weight	438.57	
Temperature	150(2) K	
Wavelength	1.54178 Å	
Crystal system	Orthorhombic	
Space group	Pbca	
Unit cell dimensions	a = 16.6170(5) Å	α= 90°.
	b = 11.8972(3) Å	β= 90°.
	c = 22.9385(7) Å	γ = 90°.
Volume	4534.8(2) $Å^3$	
Z	8	
Density (calculated)	1.285 Mg/m^3	
Absorption coefficient	1.559 mm^{-1}	
F(000)	1888	
Crystal size	0.260 x 0.120 x 0.060 mm^3	
Theta range for data collection	3.854 to 66.365°.	
Index ranges	-19<=h<=17, -12<=k<=14, -27<=l<=25	
Reflections collected	47355	
Independent reflections	3931 [R(int) = 0.0492]	
Completeness to theta = 66.365°	98.8 %	
Absorption correction	Semi-empirical from equivalents	
Max. and min. transmission	0.7528 and 0.6205	
Refinement method	Full-matrix least-squares on F^2	
Data / restraints / parameters	3931 / 0 / 281	
Goodness-of-fit on F^2	1.050	
Final R indices [I>2sigma(I)]	R1 = 0.0525, wR2 = 0.1348	

R indices (all data)	R1 = 0.0595, wR2 = 0.1402
Extinction coefficient	n/a
Largest diff. peak and hole	1.072 and -0.666 e.Å^{-3}

Table S20. Atomic coordinates (x 10^4) and equivalent isotropic displacement parameters (Å^{2}x 10^3) for ***cis*-1r**. U(eq) is defined as one third of the trace of the orthogonalized U^{ij} tensor.

	x	y	z	U(eq)
O(1)	2920(1)	8069(1)	8231(1)	25(1)
O(2)	2984(1)	5018(2)	6143(1)	36(1)
O(3)	2614(1)	5190(1)	7979(1)	27(1)
O(4)	5705(1)	8925(2)	5321(1)	37(1)
N(1)	3344(1)	5631(2)	6608(1)	26(1)
N(2)	2540(1)	6146(1)	8822(1)	21(1)
C(1)	2621(1)	7195(2)	7871(1)	21(1)
C(2)	1751(1)	7437(2)	7673(1)	25(1)
C(3)	1427(1)	6555(2)	7255(1)	28(1)
C(4)	1969(1)	6392(2)	6720(1)	28(1)
C(5)	2844(1)	6264(2)	6874(1)	22(1)
C(6)	3204(1)	7044(2)	7334(1)	20(1)
C(7)	2601(1)	6110(2)	8237(1)	21(1)
C(8)	2532(1)	5076(2)	9145(1)	23(1)
C(9)	2626(2)	5432(2)	9778(1)	32(1)
C(10)	2194(2)	6560(2)	9796(1)	32(1)
C(11)	2428(2)	7115(2)	9223(1)	28(1)
C(12)	3294(1)	8234(2)	7043(1)	25(1)
C(13)	3929(1)	8352(2)	6579(1)	23(1)
C(14)	4668(2)	8851(2)	6705(1)	29(1)
C(15)	5246(2)	9029(2)	6281(1)	31(1)
C(16)	5097(2)	8698(2)	5709(1)	27(1)
C(17)	4376(2)	8195(2)	5570(1)	30(1)
C(18)	3799(1)	8030(2)	6003(1)	28(1)
C(19)	4025(1)	6630(2)	7546(1)	24(1)
C(20)	5582(2)	8614(2)	4728(1)	41(1)
S(1)	5164(1)	4399(1)	5814(1)	38(1)
O(5)	4350(1)	4368(2)	5529(1)	52(1)
C(21)	4997(2)	3863(3)	6509(2)	69(1)

C(22)	5324(2)	5800(2)	6024(2)	48(1)

Table S21. Bond lengths [Å] and angles [°] for ***cis*-1r**.

O(1)-C(1)	1.418(3)
O(1)-H(1O)	0.79(3)
O(2)-N(1)	1.424(2)
O(2)-H(2O)	0.91(5)
O(3)-C(7)	1.244(3)
O(4)-C(16)	1.374(3)
O(4)-C(20)	1.424(3)
N(1)-C(5)	1.278(3)
N(2)-C(7)	1.345(3)
N(2)-C(8)	1.473(3)
N(2)-C(11)	1.488(3)
C(1)-C(7)	1.541(3)
C(1)-C(2)	1.543(3)
C(1)-C(6)	1.577(3)
C(2)-C(3)	1.521(3)
C(2)-H(2A)	0.9900
C(2)-H(2B)	0.9900
C(3)-C(4)	1.535(3)
C(3)-H(3A)	0.9900
C(3)-H(3B)	0.9900
C(4)-C(5)	1.503(3)
C(4)-H(4A)	0.9900
C(4)-H(4B)	0.9900
C(5)-C(6)	1.526(3)
C(6)-C(19)	1.530(3)
C(6)-C(12)	1.572(3)
C(8)-C(9)	1.521(3)
C(8)-H(8A)	0.9900
C(8)-H(8B)	0.9900
C(9)-C(10)	1.524(4)
C(9)-H(9A)	0.9900
C(9)-H(9B)	0.9900
C(10)-C(11)	1.521(3)

C(10)-H(10A)	0.9900
C(10)-H(10B)	0.9900
C(11)-H(11A)	0.9900
C(11)-H(11B)	0.9900
C(12)-C(13)	1.506(3)
C(12)-H(12A)	0.9900
C(12)-H(12B)	0.9900
C(13)-C(18)	1.391(3)
C(13)-C(14)	1.394(3)
C(14)-C(15)	1.384(3)
C(14)-H(14A)	0.9500
C(15)-C(16)	1.390(4)
C(15)-H(15A)	0.9500
C(16)-C(17)	1.378(4)
C(17)-C(18)	1.394(3)
C(17)-H(17A)	0.9500
C(18)-H(18A)	0.9500
C(19)-H(19A)	0.9800
C(19)-H(19B)	0.9800
C(19)-H(19C)	0.9800
C(20)-H(20A)	0.9800
C(20)-H(20B)	0.9800
C(20)-H(20C)	0.9800
S(1)-O(5)	1.502(2)
S(1)-C(21)	1.739(4)
S(1)-C(22)	1.755(3)
C(21)-H(21A)	0.9800
C(21)-H(21B)	0.9800
C(21)-H(21C)	0.9800
C(22)-H(22A)	0.9800
C(22)-H(22B)	0.9800
C(22)-H(22C)	0.9800

C(1)-O(1)-H(1O)	107(2)
N(1)-O(2)-H(2O)	100(3)
C(16)-O(4)-C(20)	117.6(2)
C(5)-N(1)-O(2)	112.78(19)
C(7)-N(2)-C(8)	118.40(18)

C(7)-N(2)-C(11)	130.62(18)
C(8)-N(2)-C(11)	110.87(17)
O(1)-C(1)-C(7)	107.71(17)
O(1)-C(1)-C(2)	111.24(18)
C(7)-C(1)-C(2)	107.21(18)
O(1)-C(1)-C(6)	108.94(17)
C(7)-C(1)-C(6)	110.13(17)
C(2)-C(1)-C(6)	111.53(17)
C(3)-C(2)-C(1)	112.89(18)
C(3)-C(2)-H(2A)	109.0
C(1)-C(2)-H(2A)	109.0
C(3)-C(2)-H(2B)	109.0
C(1)-C(2)-H(2B)	109.0
H(2A)-C(2)-H(2B)	107.8
C(2)-C(3)-C(4)	112.50(19)
C(2)-C(3)-H(3A)	109.1
C(4)-C(3)-H(3A)	109.1
C(2)-C(3)-H(3B)	109.1
C(4)-C(3)-H(3B)	109.1
H(3A)-C(3)-H(3B)	107.8
C(5)-C(4)-C(3)	113.10(19)
C(5)-C(4)-H(4A)	109.0
C(3)-C(4)-H(4A)	109.0
C(5)-C(4)-H(4B)	109.0
C(3)-C(4)-H(4B)	109.0
H(4A)-C(4)-H(4B)	107.8
N(1)-C(5)-C(4)	125.2(2)
N(1)-C(5)-C(6)	115.68(19)
C(4)-C(5)-C(6)	118.67(19)
C(5)-C(6)-C(19)	111.97(17)
C(5)-C(6)-C(12)	107.03(17)
C(19)-C(6)-C(12)	109.90(18)
C(5)-C(6)-C(1)	111.57(17)
C(19)-C(6)-C(1)	109.56(17)
C(12)-C(6)-C(1)	106.65(17)
O(3)-C(7)-N(2)	120.25(19)
O(3)-C(7)-C(1)	118.46(18)
N(2)-C(7)-C(1)	121.25(18)

N(2)-C(8)-C(9)	103.89(18)
N(2)-C(8)-H(8A)	111.0
C(9)-C(8)-H(8A)	111.0
N(2)-C(8)-H(8B)	111.0
C(9)-C(8)-H(8B)	111.0
H(8A)-C(8)-H(8B)	109.0
C(8)-C(9)-C(10)	102.82(19)
C(8)-C(9)-H(9A)	111.2
C(10)-C(9)-H(9A)	111.2
C(8)-C(9)-H(9B)	111.2
C(10)-C(9)-H(9B)	111.2
H(9A)-C(9)-H(9B)	109.1
C(11)-C(10)-C(9)	103.81(19)
C(11)-C(10)-H(10A)	111.0
C(9)-C(10)-H(10A)	111.0
C(11)-C(10)-H(10B)	111.0
C(9)-C(10)-H(10B)	111.0
H(10A)-C(10)-H(10B)	109.0
N(2)-C(11)-C(10)	103.35(18)
N(2)-C(11)-H(11A)	111.1
C(10)-C(11)-H(11A)	111.1
N(2)-C(11)-H(11B)	111.1
C(10)-C(11)-H(11B)	111.1
H(11A)-C(11)-H(11B)	109.1
C(13)-C(12)-C(6)	116.74(18)
C(13)-C(12)-H(12A)	108.1
C(6)-C(12)-H(12A)	108.1
C(13)-C(12)-H(12B)	108.1
C(6)-C(12)-H(12B)	108.1
H(12A)-C(12)-H(12B)	107.3
C(18)-C(13)-C(14)	116.8(2)
C(18)-C(13)-C(12)	122.5(2)
C(14)-C(13)-C(12)	120.6(2)
C(15)-C(14)-C(13)	122.1(2)
C(15)-C(14)-H(14A)	119.0
C(13)-C(14)-H(14A)	119.0
C(14)-C(15)-C(16)	119.7(2)
C(14)-C(15)-H(15A)	120.1

C(16)-C(15)-H(15A)	120.1
O(4)-C(16)-C(17)	125.1(2)
O(4)-C(16)-C(15)	115.2(2)
C(17)-C(16)-C(15)	119.7(2)
C(16)-C(17)-C(18)	119.6(2)
C(16)-C(17)-H(17A)	120.2
C(18)-C(17)-H(17A)	120.2
C(13)-C(18)-C(17)	122.1(2)
C(13)-C(18)-H(18A)	119.0
C(17)-C(18)-H(18A)	119.0
C(6)-C(19)-H(19A)	109.5
C(6)-C(19)-H(19B)	109.5
H(19A)-C(19)-H(19B)	109.5
C(6)-C(19)-H(19C)	109.5
H(19A)-C(19)-H(19C)	109.5
H(19B)-C(19)-H(19C)	109.5
O(4)-C(20)-H(20A)	109.5
O(4)-C(20)-H(20B)	109.5
H(20A)-C(20)-H(20B)	109.5
O(4)-C(20)-H(20C)	109.5
H(20A)-C(20)-H(20C)	109.5
H(20B)-C(20)-H(20C)	109.5
O(5)-S(1)-C(21)	104.29(17)
O(5)-S(1)-C(22)	106.25(14)
C(21)-S(1)-C(22)	96.89(18)
S(1)-C(21)-H(21A)	109.5
S(1)-C(21)-H(21B)	109.5
H(21A)-C(21)-H(21B)	109.5
S(1)-C(21)-H(21C)	109.5
H(21A)-C(21)-H(21C)	109.5
H(21B)-C(21)-H(21C)	109.5
S(1)-C(22)-H(22A)	109.5
S(1)-C(22)-H(22B)	109.5
H(22A)-C(22)-H(22B)	109.5
S(1)-C(22)-H(22C)	109.5
H(22A)-C(22)-H(22C)	109.5
H(22B)-C(22)-H(22C)	109.5

Symmetry transformations used to generate equivalent atoms:

Table S22. Anisotropic displacement parameters ($Å^2$x 10^3) for ***cis*-1r**. The anisotropic displacement factor exponent takes the form: $-2\pi^2[h^2 a^{*2}U^{11} + ...+ 2 h k a^* b^* U^{12}]$

	U^{11}	U^{22}	U^{33}	U^{23}	U^{13}	U^{12}
O(1)	38(1)	13(1)	25(1)	-2(1)	0(1)	0(1)
O(2)	36(1)	43(1)	29(1)	-16(1)	-4(1)	-5(1)
O(3)	41(1)	15(1)	24(1)	-2(1)	1(1)	-2(1)
O(4)	40(1)	41(1)	32(1)	-1(1)	14(1)	-6(1)
N(1)	32(1)	24(1)	21(1)	-6(1)	-3(1)	-2(1)
N(2)	27(1)	14(1)	22(1)	-1(1)	2(1)	0(1)
C(1)	26(1)	15(1)	22(1)	-1(1)	0(1)	-1(1)
C(2)	24(1)	22(1)	30(1)	4(1)	5(1)	4(1)
C(3)	21(1)	29(1)	35(1)	3(1)	-2(1)	0(1)
C(4)	24(1)	33(1)	28(1)	-1(1)	-6(1)	-1(1)
C(5)	24(1)	22(1)	20(1)	2(1)	-2(1)	-2(1)
C(6)	22(1)	16(1)	21(1)	-1(1)	0(1)	0(1)
C(7)	22(1)	16(1)	23(1)	0(1)	1(1)	1(1)
C(8)	28(1)	17(1)	24(1)	3(1)	1(1)	1(1)
C(9)	46(2)	29(1)	22(1)	2(1)	0(1)	1(1)
C(10)	44(2)	29(1)	22(1)	-4(1)	6(1)	-1(1)
C(11)	38(1)	20(1)	25(1)	-5(1)	4(1)	0(1)
C(12)	27(1)	18(1)	30(1)	3(1)	4(1)	0(1)
C(13)	25(1)	18(1)	27(1)	4(1)	1(1)	2(1)
C(14)	32(1)	28(1)	26(1)	-1(1)	2(1)	-4(1)
C(15)	28(1)	31(1)	32(1)	-1(1)	4(1)	-7(1)
C(16)	31(1)	21(1)	31(1)	5(1)	7(1)	2(1)
C(17)	36(1)	31(1)	23(1)	3(1)	-1(1)	1(1)
C(18)	26(1)	31(1)	28(1)	5(1)	-4(1)	-2(1)
C(19)	24(1)	22(1)	27(1)	-4(1)	-5(1)	2(1)
C(20)	59(2)	34(1)	31(1)	0(1)	15(1)	3(1)
S(1)	34(1)	35(1)	43(1)	-6(1)	-3(1)	5(1)
O(5)	36(1)	69(2)	50(1)	-24(1)	-8(1)	6(1)
C(21)	65(2)	54(2)	87(3)	23(2)	17(2)	-3(2)
C(22)	40(2)	38(2)	65(2)	-7(1)	7(1)	3(1)

Table S23. Hydrogen coordinates (x 10^4) and isotropic displacement parameters (Å^{2}x 10^3) for ***cis*-1r**.

	x	y	z	U(eq)
H(1O)	2711(18)	8640(30)	8129(13)	37(9)
H(2O)	3430(30)	4760(40)	5960(20)	105(17)
H(2A)	1399	7471	8020	31
H(2B)	1734	8181	7480	31
H(3A)	883	6782	7124	34
H(3B)	1375	5830	7464	34
H(4A)	1790	5716	6505	34
H(4B)	1907	7047	6458	34
H(8A)	2982	4586	9022	27
H(8B)	2018	4671	9085	27
H(9A)	3201	5514	9885	39
H(9B)	2370	4883	10045	39
H(10A)	1604	6456	9821	38
H(10B)	2376	7015	10133	38
H(11A)	1996	7620	9082	33
H(11B)	2932	7551	9265	33
H(12A)	2769	8442	6870	30
H(12B)	3411	8786	7355	30
H(14A)	4777	9076	7094	34
H(15A)	5742	9377	6379	37
H(17A)	4272	7961	5181	36
H(18A)	3302	7688	5902	34
H(19A)	4194	7077	7883	36
H(19B)	4421	6710	7233	36
H(19C)	3985	5838	7659	36
H(20A)	6041	8865	4493	62
H(20B)	5089	8967	4582	62
H(20C)	5533	7795	4700	62
H(21A)	4891	3054	6483	103
H(21B)	4533	4241	6684	103
H(21C)	5475	3991	6751	103
H(22A)	5432	6258	5678	72
H(22B)	5786	5837	6289	72

H(22C)	4844	6085	6223	72

Table S24. Torsion angles [°] for ***cis*-1r**.

O(1)-C(1)-C(2)-C(3)	176.45(18)
C(7)-C(1)-C(2)-C(3)	-66.0(2)
C(6)-C(1)-C(2)-C(3)	54.6(2)
C(1)-C(2)-C(3)-C(4)	-55.0(3)
C(2)-C(3)-C(4)-C(5)	47.8(3)
O(2)-N(1)-C(5)-C(4)	-1.3(3)
O(2)-N(1)-C(5)-C(6)	-173.41(18)
C(3)-C(4)-C(5)-N(1)	144.6(2)
C(3)-C(4)-C(5)-C(6)	-43.5(3)
N(1)-C(5)-C(6)-C(19)	-21.2(3)
C(4)-C(5)-C(6)-C(19)	166.15(19)
N(1)-C(5)-C(6)-C(12)	99.3(2)
C(4)-C(5)-C(6)-C(12)	-73.4(2)
N(1)-C(5)-C(6)-C(1)	-144.39(19)
C(4)-C(5)-C(6)-C(1)	43.0(3)
O(1)-C(1)-C(6)-C(5)	-169.98(17)
C(7)-C(1)-C(6)-C(5)	72.1(2)
C(2)-C(1)-C(6)-C(5)	-46.8(2)
O(1)-C(1)-C(6)-C(19)	65.4(2)
C(7)-C(1)-C(6)-C(19)	-52.5(2)
C(2)-C(1)-C(6)-C(19)	-171.38(17)
O(1)-C(1)-C(6)-C(12)	-53.4(2)
C(7)-C(1)-C(6)-C(12)	-171.36(17)
C(2)-C(1)-C(6)-C(12)	69.7(2)
C(8)-N(2)-C(7)-O(3)	3.0(3)
C(11)-N(2)-C(7)-O(3)	-172.8(2)
C(8)-N(2)-C(7)-C(1)	-179.43(19)
C(11)-N(2)-C(7)-C(1)	4.8(4)
O(1)-C(1)-C(7)-O(3)	-157.1(2)
C(2)-C(1)-C(7)-O(3)	83.1(2)
C(6)-C(1)-C(7)-O(3)	-38.4(3)
O(1)-C(1)-C(7)-N(2)	25.3(3)
C(2)-C(1)-C(7)-N(2)	-94.5(2)

C(6)-C(1)-C(7)-N(2)	144.0(2)
C(7)-N(2)-C(8)-C(9)	169.2(2)
C(11)-N(2)-C(8)-C(9)	-14.2(3)
N(2)-C(8)-C(9)-C(10)	32.7(2)
C(8)-C(9)-C(10)-C(11)	-39.5(3)
C(7)-N(2)-C(11)-C(10)	165.9(2)
C(8)-N(2)-C(11)-C(10)	-10.2(3)
C(9)-C(10)-C(11)-N(2)	30.5(2)
C(5)-C(6)-C(12)-C(13)	-70.3(2)
C(19)-C(6)-C(12)-C(13)	51.5(3)
C(1)-C(6)-C(12)-C(13)	170.21(19)
C(6)-C(12)-C(13)-C(18)	82.2(3)
C(6)-C(12)-C(13)-C(14)	-101.3(3)
C(18)-C(13)-C(14)-C(15)	0.4(4)
C(12)-C(13)-C(14)-C(15)	-176.3(2)
C(13)-C(14)-C(15)-C(16)	-0.4(4)
C(20)-O(4)-C(16)-C(17)	-0.2(4)
C(20)-O(4)-C(16)-C(15)	-179.4(2)
C(14)-C(15)-C(16)-O(4)	179.2(2)
C(14)-C(15)-C(16)-C(17)	0.0(4)
O(4)-C(16)-C(17)-C(18)	-178.7(2)
C(15)-C(16)-C(17)-C(18)	0.5(4)
C(14)-C(13)-C(18)-C(17)	0.1(3)
C(12)-C(13)-C(18)-C(17)	176.7(2)
C(16)-C(17)-C(18)-C(13)	-0.5(4)

Symmetry transformations used to generate equivalent atoms:

Table S25. Hydrogen bonds for ***cis*-1r** [Å and °].

D-H...A	d(D-H)	d(H...A)	d(D...A)	<(DHA)
O(1)-H(1O)...O(3)#1	0.79(3)	1.96(3)	2.736(2)	167(3)
O(2)-H(2O)...O(5)	0.91(5)	1.87(5)	2.780(3)	175(5)

Symmetry transformations used to generate equivalent atoms:

#1 -x+1/2,y+1/2,z

References

1. Yasuhiro, S.; Yoshihide, T.; Masaaki, M.; Keiji, T. Asymmetric Total Synthesis of Glycinoeclepin A: Generation of a Novel Bridgehead Anion Species. *Chem. Lett.* **2010**, *39*, 835-837.
2. Fukaya, K. et al. Synthesis of Paclitaxel. 1. Synthesis of the ABC Ring of Paclitaxel by SmI_2-Mediated Cyclization. *Org. Lett.* **2015**, *17*, 2570-2573.
3. Nambu, H.; Ono, N.; Hirota, W.; Fukumoto, M.; Yakura, T. An Efficient Method for the Synthesis of 2´,3´-Nonsubstituted Cycloalkane-1,3-dione-2-spirocyclopropanes Using (2-Bromoethyl)diphenylsulfonium Trifluoromethanesulfonate. *Chem. Lett.* **2016**, *64*, 1763-1768.
4. Soni, R.; Collinson, J.-M.; Clarkson, G. C.; Wills, M. An Unexpected Directing Effect in the Asymmetric Transfer Hydrogenation of α,α-Disubstituted Ketones. *Org. Lett.* **2011**, *13*, 4304-4307.
5. Jacques, R.; Pullin, R. D. C.; Fletcher, S. P. Desymmetrization of meso-bisphosphates using copper catalysis and alkylzirconocene nucleophiles. *Nat. Commun.* **2019**, *10*, 21.
6. Mandai, H.; Hironaka, T.; Mitsudo, K.; Suga, S. Acylative Desymmetrization of Cyclic *meso*-1,3-Diols by Chiral DMAP Derivatives. *Chem. Lett.* **2021**, *50*,471-474.
7. Qin, X.-L.; Li, A.; Han, F.-S. Desymmetric Enantioselective Reduction of Cyclic 1,3-Diketones Catalyzed by a Recyclable *P*-Chiral Phosphinamide Organocatalyst. *J. Am. Chem. Soc.* **2021**, *143*, 2994-3002.
8. Synthesized according to a modified procedure where methyl acrylate was replaced with *tert*-butyl acrylate: Brooks, D. W.; Mazdiyasni, H.; Grothaus, P. G. Asymmetric microbial reduction of prochiral 2,2-disubstituted cycloalkanediones. *J. Org. Chem.* **1987**, *52*, 3223-3232.
9. Li, J.; Huang, C.; Wen, D.; Zheng, Q.; Tu, B.; Tu, T. Nickel-Catalyzed Amination of Aryl Chlorides with Amides. *Org. Lett.* **2021**, *23*, 687-691.
10. Nayar, M. S. B.; Callery, P. S. Formylation of Secondary Amines with Dialkyl Acetals of Dimethylformamide. *Anal. Lett.* **1980**, *13*, 625-634.
11. Huang, L.; Zhang, X.; Zhang, Y. CuBr-Catalyzed Reaction of *N*,*N*-Dimethylanilines and Silyl Enol Ethers: An Alternative Route to β-Arylamino Ketones. *Org. Lett.* **2009**, *11*, 3730-3733.
12. Mycka, R. J.; Eckenhoff, W. T.; Steward, O. W.; Barefoot, N. Z.; Fleming, F. F. γ- and δ-Hydroxynitriles: diastereoselective electrophile-dependent alkylations. *Tetrahedron* **2013**, *69*, 366-376.
